# Genomic epidemiology of SARS-CoV-2 in a university outbreak setting and implications for public health planning

**DOI:** 10.1038/s41598-022-15661-1

**Published:** 2022-07-19

**Authors:** Sema Nickbakhsh, Joseph Hughes, Nicolaos Christofidis, Emily Griffiths, Sharif Shaaban, Jessica Enright, Katherine Smollett, Kyriaki Nomikou, Natasha Palmalux, Lily Tong, Stephen Carmichael, Vattipally B. Sreenu, Richard Orton, Emily J. Goldstein, Rachael M. Tomb, The COVID-19 Genomics UK (COG-UK) Consortium, Samuel C. Robson, Thomas R. Connor, Nicholas J. Loman, Tanya Golubchik, Rocio T. Martinez Nunez, David Bonsall, Andrew Rambaut, Luke B. Snell, Rich Livett, Catherine Ludden, Sally Corden, Eleni Nastouli, Gaia Nebbia, Ian Johnston, Katrina Lythgoe, M. Estee Torok, Ian G. Goodfellow, Jacqui A. Prieto, Kordo Saeed, David K. Jackson, Catherine Houlihan, Dan Frampton, William L. Hamilton, Adam A. Witney, Giselda Bucca, Cassie F. Pope, Catherine Moore, Emma C. Thomson, Ewan M. Harrison, Colin P. Smith, Fiona Rogan, Shaun M. Beckwith, Abigail Murray, Dawn Singleton, Kirstine Eastick, Liz A. Sheridan, Paul Randell, Leigh M. Jackson, Cristina V. Ariani, Sónia Gonçalves, Derek J. Fairley, Matthew W. Loose, Joanne Watkins, Samuel Moses, Sam Nicholls, Matthew Bull, Roberto Amato, Darren L. Smith, David M. Aanensen, Jeffrey C. Barrett, Dinesh Aggarwal, James G. Shepherd, Martin D. Curran, Surendra Parmar, Matthew D. Parker, Catryn Williams, Sharon Glaysher, Anthony P. Underwood, Matthew Bashton, Nicole Pacchiarini, Katie F. Loveson, Matthew Byott, Alessandro M. Carabelli, Kate E. Templeton, Thushan I. de Silva, Dennis Wang, Cordelia F. Langford, John Sillitoe, Rory N. Gunson, Simon Cottrell, Justin O’Grady, Dominic Kwiatkowski, Patrick J. Lillie, Nicholas Cortes, Nathan Moore, Claire Thomas, Phillipa J. Burns, Tabitha W. Mahungu, Steven Liggett, Angela H. Beckett, Matthew T. G. Holden, Lisa J. Levett, Husam Osman, Mohammed O. Hassan-Ibrahim, David A. Simpson, Mrera Chand, Ravi K. Gupta, Alistair C. Darby, Steve Paterson, Oliver G. Pybus, Erik M. Volz, Daniela de Angelis, David L. Robertson, Andrew J. Page, Inigo Martincorena, Louise Aigrain, Andrew R. Bassett, Nick Wong, Yusri Taha, Michelle J. Erkiert, Michael H. Spencer Chapman, Rebecca Dewar, Martin P. McHugh, Siddharth Mookerjee, Stephen Aplin, Matthew Harvey, Thea Sass, Helen Umpleby, Helen Wheeler, James P. McKenna, Ben Warne, Joshua F. Taylor, Yasmin Chaudhry, Rhys Izuagbe, Aminu S. Jahun, Gregory R. Young, Claire McMurray, Clare M. McCann, Andrew Nelson, Scott Elliott, Hannah Lowe, Anna Price, Matthew R. Crown, Sara Rey, Sunando Roy, Ben Temperton, Sharif Shaaban, Andrew R. Hesketh, Kenneth G. Laing, Irene M. Monahan, Judith Heaney, Emanuela Pelosi, Siona Silviera, Eleri Wilson-Davies, Helen Fryer, Helen Adams, Louis du Plessis, Rob Johnson, William T. Harvey, Joseph Hughes, Richard J. Orton, Lewis G. Spurgin, Yann Bourgeois, Chris Ruis, Áine O’Toole, Marina Gourtovaia, Theo Sanderson, Christophe Fraser, Jonathan Edgeworth, Judith Breuer, Stephen L. Michell, John A. Todd, Michaela John, David Buck, Kavitha Gajee, Gemma L. Kay, Sharon J. Peacock, David Heyburn, Katie Kitchman, Alan McNally, David T. Pritchard, Samir Dervisevic, Peter Muir, Esther Robinson, Barry B. Vipond, Newara A. Ramadan, Christopher Jeanes, Danni Weldon, Jana Catalan, Neil Jones, Ana da Silva Filipe, Chris Williams, Marc Fuchs, Julia Miskelly, Aaron R. Jeffries, Karen Oliver, Naomi R. Park, Amy Ash, Cherian Koshy, Magdalena Barrow, Sarah L. Buchan, Anna Mantzouratou, Gemma Clark, Christopher W. Holmes, Sharon Campbell, Thomas Davis, Ngee Keong Tan, Julianne R. Brown, Kathryn A. Harris, Stephen P. Kidd, Paul R. Grant, Li Xu-McCrae, Alison Cox, Pinglawathee Madona, Marcus Pond, Paul A. Randell, Karen T. Withell, Cheryl Williams, Clive Graham, Rebecca Denton-Smith, Emma Swindells, Robyn Turnbull, Tim J. Sloan, Andrew Bosworth, Stephanie Hutchings, Hannah M. Pymont, Anna Casey, Liz Ratcliffe, Christopher R. Jones, Bridget A. Knight, Tanzina Haque, Jennifer Hart, Dianne Irish-Tavares, Eric Witele, Craig Mower, Louisa K. Watson, Jennifer Collins, Gary Eltringham, Dorian Crudgington, Ben Macklin, Miren Iturriza-Gomara, Anita O. Lucaci, Patrick C. McClure, Matthew Carlile, Nadine Holmes, Christopher Moore, Nathaniel Storey, Stefan Rooke, Gonzalo Yebra, Noel Craine, Malorie Perry, Nabil-Fareed Alikhan, Stephen Bridgett, Kate F. Cook, Christopher Fearn, Salman Goudarzi, Ronan A. Lyons, Thomas Williams, Sam T. Haldenby, Jillian Durham, Steven Leonard, Robert M. Davies, Rahul Batra, Beth Blane, Moira J. Spyer, Perminder Smith, Mehmet Yavus, Rachel J. Williams, Adhyana I. K. Mahanama, Buddhini Samaraweera, Sophia T. Girgis, Samantha E. Hansford, Angie Green, Charlotte Beaver, Katherine L. Bellis, Matthew J. Dorman, Sally Kay, Liam Prestwood, Shavanthi Rajatileka, Joshua Quick, Radoslaw Poplawski, Nicola Reynolds, Andrew Mack, Arthur Morriss, Thomas Whalley, Bindi Patel, Iliana Georgana, Myra Hosmillo, Malte L. Pinckert, Joanne Stockton, John H. Henderson, Amy Hollis, William Stanley, Wen C. Yew, Richard Myers, Alicia Thornton, Alexander Adams, Tara Annett, Hibo Asad, Alec Birchley, Jason Coombes, Johnathan M. Evans, Laia Fina, Bree Gatica-Wilcox, Lauren Gilbert, Lee Graham, Jessica Hey, Ember Hilvers, Sophie Jones, Hannah Jones, Sara Kumziene-Summerhayes, Caoimhe McKerr, Jessica Powell, Georgia Pugh, Sarah Taylor, Alexander J. Trotter, Charlotte A. Williams, Leanne M. Kermack, Benjamin H. Foulkes, Marta Gallis, Hailey R. Hornsby, Stavroula F. Louka, Manoj Pohare, Paige Wolverson, Peijun Zhang, George MacIntyre-Cockett, Amy Trebes, Robin J. Moll, Lynne Ferguson, Emily J. Goldstein, Alasdair Maclean, Rachael Tomb, Igor Starinskij, Laura Thomson, Joel Southgate, Moritz U. G. Kraemer, Jayna Raghwani, Alex E. Zarebski, Olivia Boyd, Lily Geidelberg, Chris J. Illingworth, Chris Jackson, David Pascall, Sreenu Vattipally, Timothy M. Freeman, Sharon N. Hsu, Benjamin B. Lindsey, Keith James, Kevin Lewis, Gerry Tonkin-Hill, Jaime M. Tovar-Corona, MacGregor Cox, Khalil Abudahab, Mirko Menegazzo, Ben E. W. Taylor, Corin A. Yeats, Afrida Mukaddas, Derek W. Wright, Leonardo de Oliveira Martins, Rachel Colquhoun, Verity Hill, Ben Jackson, J. T. McCrone, Nathan Medd, Emily Scher, Jon-Paul Keatley, Tanya Curran, Sian Morgan, Patrick Maxwell, Ken Smith, Sahar Eldirdiri, Anita Kenyon, Alison H. Holmes, James R. Price, Tim Wyatt, Alison E. Mather, Timofey John A. SkvortsovHartley, Martyn Guest, Christine Kitchen, Ian Merrick, Robert Munn, Beatrice Bertolusso, Jessica Lynch, Gabrielle Vernet, Stuart Kirk, Elizabeth Wastnedge, Rachael Stanley, Giles Idle, Declan T. Bradley, Jennifer Poyner, Matilde Mori, Owen Jones, Victoria Wright, Ellena Brooks, Carol M. Churcher, Mireille Fragakis, Katerina Galai, Andrew Jermy, Sarah Judges, Georgina M. McManus, Kim S. Smith, Elaine Westwick, Stephen W. Attwood, Frances Bolt, Alisha Davies, Elen De Lacy, Fatima Downing, Sue Edwards, Lizzie Meadows, Sarah Jeremiah, Nikki Smith, Luke Foulser, Themoula Charalampous, Amita Patel, Louise Berry, Tim Boswell, Vicki M. Fleming, Hannah C. Howson-Wells, Amelia Joseph, Manjinder Khakh, Michelle M. Lister, Paul W. Bird, Karlie Fallon, Thomas Helmer, Claire L. McMurray, Mina Odedra, Jessica Shaw, Julian W. Tang, Nicholas J. Willford, Victoria Blakey, Veena Raviprakash, Nicola Sheriff, Lesley-Anne Williams, Theresa Feltwell, Luke Bedford, James S. Cargill, Warwick Hughes, Jonathan Moore, Susanne Stonehouse, Laura Atkinson, Jack C. D. Lee, Divya Shah, Adela Alcolea-Medina, Natasha Ohemeng-Kumi, John Ramble, Jasveen Sehmi, Rebecca Williams, Wendy Chatterton, Monika Pusok, William Everson, Anibolina Castigador, Emily Macnaughton, Kate El Bouzidi, Temi Lampejo, Malur Sudhanva, Cassie Breen, Graciela Sluga, Shazaad S. Y. Ahmad, Ryan P. George, Nicholas W. Machin, Debbie Binns, Victoria James, Rachel Blacow, Lindsay Coupland, Louise Smith, Edward Barton, Debra Padgett, Garren Scott, Aidan Cross, Mariyam Mirfenderesky, Jane Greenaway, Kevin Cole, Phillip Clarke, Nichola Duckworth, Sarah Walsh, Kelly Bicknell, Robert Impey, Sarah Wyllie, Richard Hopes, Chloe Bishop, Vicki Chalker, Ian Harrison, Laura Gifford, Zoltan Molnar, Cressida Auckland, Cariad Evans, Kate Johnson, David G. Partridge, Mohammad Raza, Paul Baker, Stephen Bonner, Sarah Essex, Leanne J. Murray, Andrew I. Lawton, Shirelle Burton-Fanning, Brendan A. I. Payne, Sheila Waugh, Andrea N. Gomes, Maimuna Kimuli, Darren R. Murray, Paula Ashfield, Donald Dobie, Fiona Ashford, Angus Best, Liam Crawford, Nicola Cumley, Megan Mayhew, Oliver Megram, Jeremy Mirza, Emma Moles-Garcia, Benita Percival, Megan Driscoll, Leah Ensell, Helen L. Lowe, Laurentiu Maftei, Matteo Mondani, Nicola J. Chaloner, Benjamin J. Cogger, Lisa J. Easton, Hannah Huckson, Jonathan Lewis, Sarah Lowdon, Cassandra S. Malone, Florence Munemo, Manasa Mutingwende, Roberto Nicodemi, Olga Podplomyk, Thomas Somassa, Andrew Beggs, Alex Richter, Claire Cormie, Joana Dias, Sally Forrest, Ellen E. Higginson, Mailis Maes, Jamie Young, Rose K. Davidson, Kathryn A. Jackson, Lance Turtle, Alexander J. Keeley, Jonathan Ball, Timothy Byaruhanga, Joseph G. Chappell, Jayasree Dey, Jack D. Hill, Emily J. Park, Arezou Fanaie, Rachel A. Hilson, Geraldine Yaze, Stephanie Lo, Safiah Afifi, Robert Beer, Joshua Maksimovic, Kathryn McCluggage, Karla Spellman, Catherine Bresner, William Fuller, Angela Marchbank, Trudy Workman, Ekaterina Shelest, Johnny Debebe, Fei Sang, Marina Escalera Zamudio, Sarah Francois, Bernardo Gutierrez, Tetyana I. Vasylyeva, Flavia Flaviani, Manon Ragonnet-Cronin, Katherine L. Smollett, Alice Broos, Daniel Mair, Jenna Nichols, Kyriaki Nomikou, Lily Tong, Ioulia Tsatsani, Sarah O’Brien, Steven Rushton, Roy Sanderson, Jon Perkins, Seb Cotton, Abbie Gallagher, Elias Allara, Clare Pearson, David Bibby, Gavin Dabrera, Nicholas Ellaby, Eileen Gallagher, Jonathan Hubb, Angie Lackenby, David Lee, Nikos Manesis, Tamyo Mbisa, Steven Platt, Katherine A. Twohig, Mari Morgan, Alp Aydin, David J. Baker, Ebenezer Foster-Nyarko, Sophie J. Prosolek, Steven Rudder, Chris Baxter, SÃ­lvia F. Carvalho, Deborah Lavin, Arun Mariappan, Clara Radulescu, Aditi Singh, Miao Tang, Helen Morcrette, Nadua Bayzid, Marius Cotic, Carlos E. Balcazar, Michael D. Gallagher, Daniel Maloney, Thomas D. Stanton, Kathleen A. Williamson, Robin Manley, Michelle L. Michelsen, Christine M. Sambles, David J. Studholme, Joanna Warwick-Dugdale, Richard Eccles, Matthew Gemmell, Richard Gregory, Margaret Hughes, Charlotte Nelson, Lucille Rainbow, Edith E. Vamos, Hermione J. Webster, Mark Whitehead, Claudia Wierzbicki, Adrienn Angyal, Luke R. Green, Max Whiteley, Emma Betteridge, Iraad F. Bronner, Ben W. Farr, Scott Goodwin, Stefanie V. Lensing, Shane A. McCarthy, Michael A. Quail, Diana Rajan, Nicholas M. Redshaw, Carol Scott, Lesley Shirley, Scott A. J. Thurston, Will Rowe, Amy Gaskin, Thanh Le-Viet, James Bonfield, Jennifier Liddle, Andrew Whitwham, Kate Templeton, Rory N. Gunson, Ana da Silva Filipe, Catriona Milosevic, Emma Thomson, David L. Robertson, Matthew T. G. Holden, Christopher J. R. Illingworth, Alison Smith-Palmer

**Affiliations:** 1 Public Health Scotland, Meridian Court, 5 Cadogan Street, Glasgow, G2 6QE UK; 2 Institute of Biodiversity, Animal Health & Comparative Medicine, University of Glasgow, Graham Kerr Building, Glasgow, G12 8QQ UK; 3 MRC-University of Glasgow Centre for Virus Research, 464 Bearsden Road, Glasgow, G61 1QH UK; 4 School of Computing Science, University of Glasgow, 18 Lilybank Gardens, Glasgow, G12 8RZ UK; 5 West of Scotland Specialist Virology Centre, NHS Greater Glasgow and Clyde, Glasgow Royal Infirmary, New Lister Building, Glasgow, G31 2ER UK; 6 https://www.cogconsortium.uk; 7 Royal Infirmary of Edinburgh, NHS Lothian, 51 Little France Crescent, Edinburgh, EH16 4SA UK; 8 NHS Greater Glasgow and Clyde, Gartnavel General Hospital, 1055 Great Western Road, Glasgow, G12 0XH UK; 9 School of Medicine, University of St Andrews, North Haugh, St Andrews, KY16 9TF UK; 10 Department of Applied Mathematics and Theoretical Physics, University of Cambridge, Cambridge, UK; 11 MRC Biostatistics Unit, University of Cambridge, East Forvie Building, Forvie Site, Robinson Way, Cambridge, CB2 0SR UK; 12 Barking, Havering and Redbridge University Hospitals NHS Trust, Romford, UK; 13 Betsi Cadwaladr University Health Board, North Wales, UK; 14 Blackpool Teaching Hospitals NHS Foundation Trust, Blackpool, UK; 15 Bournemouth University, Poole, UK; 16 Cambridge Stem Cell Institute, University of Cambridge, Cambridge, UK; 17 Cardiff and Vale University Health Board, Cardiff, UK; 18 Centre for Clinical Infection and Diagnostics Research, Department of Infectious Diseases, Guy’s and St Thomas’ NHS Foundation Trust, London, UK; 19 Centre for Enzyme Innovation, University of Portsmouth, Portsmouth, UK; 20 Centre for Genomic Pathogen Surveillance, University of Oxford, Oxford, UK; 21 Clinical Microbiology Department, Queens Medical Centre, Nottingham University Hospitals NHS Trust, Nottingham, UK; 22 Clinical Microbiology, University Hospitals of Leicester NHS Trust, Leicester, UK; 23 County Durham and Darlington NHS Foundation Trust, Durham, UK; 24 Deep Seq, School of Life Sciences, Queens Medical Centre, University of Nottingham, Nottingham, UK; 25 Department of Infectious Diseases and Microbiology, Cambridge University Hospitals NHS Foundation Trust, Cambridge, UK; 26 Department of Medicine, University of Cambridge, Cambridge, UK; 27 Department of Microbiology, Kettering General Hospital, Kettering, UK; 28 Department of Microbiology, South West London Pathology, London, UK; 29 Department of Zoology, University of Oxford, Oxford, UK; 30 Division of Virology, Department of Pathology, University of Cambridge, Cambridge, UK; 31 East Kent Hospitals University NHS Foundation Trust, Kent, UK; 32 East Suffolk and North Essex NHS Foundation Trust, Colchester, UK; 33 East Sussex Healthcare NHS Trust, Seaford, UK; 34 Gateshead Health NHS Foundation Trust, Gateshead, UK; 35 Great Ormond Street Hospital for Children, NHS Foundation Trust, London, UK; 36 Great Ormond Street Institute of Child Health (GOS ICH), University College London (UCL), London, UK; 37 Guy’s and St. Thomas’ Biomedical Research Centre, London, UK; 38 Guy’s and St. Thomas’ NHS Foundation Trust, London, UK; 39 Hampshire Hospitals NHS Foundation Trust, Basingstoke, UK; 40 Health Services Laboratories, London, UK; 41 Heartlands Hospital, Birmingham, UK; 42 Hub for Biotechnology in the Built Environment, Northumbria University, Newcastle upon Tyne, UK; 43 Hull University Teaching Hospitals NHS Trust, Hull, UK; 44 Imperial College Healthcare NHS Trust, London, UK; 45 Imperial College London, London, UK; 46 Infection Care Group, St George’’s University Hospitals NHS Foundation Trust, London, UK; 47 Institute for Infection and Immunity, St George’s University of London, London, UK; 48 Barts Health NHS Trust, London, UK; 49 Institute of Microbiology and Infection, University of Birmingham, Birmingham, UK; 50 Isle of Wight NHS Trust, Newport, UK; 51 King’s College Hospital NHS Foundation Trust, London, UK; 52 King’s College London, London, UK; 53 Liverpool Clinical Laboratories, Liverpool, UK; 54 Maidstone and Tunbridge Wells NHS Trust, Tunbridge Wells, UK; 55 Manchester University NHS Foundation Trust, Manchester, UK; 56 Microbiology Department, Buckinghamshire Healthcare NHS Trust, Amersham, UK; 57 Microbiology, Royal Oldham Hospital, Oldham, UK; 58 Cardiff University, Cardiff, UK; 59 Belfast Health & Social Care Trust, Belfast, UK; 60 Newcastle University, Newcastle upon Tyne, UK; 61 NHS Greater Glasgow and Clyde, Glasgow, UK; 62 NHS Lothian, Edinburgh, UK; 63 NIHR Health Protection Research Unit in HCAI and AMR, Imperial College London, London, UK; 64 Norfolk and Norwich University Hospitals NHS Foundation Trust, Norwich, UK; 65 Norfolk County Council, Norfolk, UK; 66 North Cumbria Integrated Care NHS Foundation Trust, Cumbria, UK; 67 North Middlesex University Hospital NHS Trust, London, UK; 68 North Tees and Hartlepool NHS Foundation Trust, Durham, UK; 69 North West London Pathology, London, UK; 70 Northumbria Healthcare NHS Foundation Trust, Tyneside, UK; 71 Northumbria University, Newcastle upon Tyne, UK; 72 NU-OMICS, Northumbria University, Newcastle upon Tyne, UK; 73 Path Links, Northern Lincolnshire and Goole NHS Foundation Trust, Lincolnshire, UK; 74 Portsmouth Hospitals University NHS Trust, Portsmouth, UK; 75 Public Health Agency, Belfast, Northern Ireland UK; 76 Public Health England, London, UK; 77 Public Health England, Cambridge, UK; 78 Public Health England, Colindale, UK; 79 Cambridge University Hospitals NHS Foundation Trust, Cambridge, UK; 80 Public Health Wales, Wales, UK; 81 Quadram Institute Bioscience, Norwich, UK; 82 Queen Elizabeth Hospital, Birmingham, UK; 83 Queen’s University Belfast, Belfast, UK; 84 Royal Brompton and Harefield Hospitals, Uxbridge, UK; 85 Royal Devon and Exeter NHS Foundation Trust, Exeter, UK; 86 Royal Free London NHS Foundation Trust, London, UK; 87 School of Biological Sciences, University of Portsmouth, Portsmouth, UK; 88 School of Health Sciences, University of Southampton, Southampton, UK; 89 School of Medicine, University of Southampton, Southampton, UK; 90 School of Pharmacy and Biomedical Sciences, University of Portsmouth, Portsmouth, UK; 91 Sheffield Teaching Hospitals NHS Foundation Trust, Sheffield, UK; 92 South Tees Hospitals NHS Foundation Trust, Middlesbrough, UK; 93 Southwest Pathology Services, Taunton, UK; 94 Swansea University, Swansea, UK; 95 The Newcastle Upon Tyne Hospitals NHS Foundation Trust, Newcastle upon Tyne, UK; 96 The Queen Elizabeth Hospital King’s Lynn NHS Foundation Trust, King’s Lynn, UK; 97 The Royal Marsden NHS Foundation Trust, London, UK; 98 The Royal Wolverhampton NHS Trust, Cannock, UK; 99 Turnkey Laboratory, University of Birmingham, Birmingham, UK; 100 University College London Division of Infection and Immunity, London, UK; 101 University College London Hospital Advanced Pathogen Diagnostics Unit, London, UK; 102 University College London Hospitals NHS Foundation Trust, London, UK; 103 University Hospital Southampton NHS Foundation Trust, Southampton, UK; 104 University Hospitals Dorset NHS Foundation Trust, Poole, UK; 105 University Hospitals Sussex NHS Foundation Trust, Sussex, UK; 106 University of Birmingham, Birmingham, UK; 107 University of Brighton, Brighton, UK; 108 University of Cambridge, Cambridge, UK; 109 University of East Anglia, Norwich, UK; 110 University of Edinburgh, Edinburgh, UK; 111 University of Exeter, Exeter, UK; 112 University of Kent, Kent, UK; 113 University of Liverpool, Liverpool, UK; 114 University of Oxford, Oxford, UK; 115 University of Sheffield, Sheffield, UK; 116 University of Southampton, Southampton, UK; 117 University of St Andrews, St Andrews, UK; 118 Viapath, Guy’s and St Thomas’ NHS Foundation Trust, and King’s College Hospital NHS Foundation Trust, London, UK; 119 Virology, School of Life Sciences, Queens Medical Centre, University of Nottingham, Nottingham, UK; 120 Watford General Hospital, Watford, UK; 121 Wellcome Centre for Human Genetics, Nuffield Department of Medicine, University of Oxford, Oxford, UK; 122 Wellcome Sanger Institute, Hinxton, UK; 123 Big Data Institute, Nuffield Department of Medicine, University of Oxford, Oxford, UK; 124 Whittington Health NHS Trust, London, UK

**Keywords:** Viral infection, Epidemiology, Phylogeny, Population genetics, Computational models

## Abstract

Whole genome sequencing of SARS-CoV-2 has occurred at an unprecedented scale, and can be exploited for characterising outbreak risks at the fine-scale needed to inform control strategies. One setting at continued risk of COVID-19 outbreaks are higher education institutions, associated with student movements at the start of term, close living conditions within residential halls, and high social contact rates. Here we analysed SARS-CoV-2 whole genome sequences in combination with epidemiological data to investigate a large cluster of student cases associated with University of Glasgow accommodation in autumn 2020, Scotland. We identified 519 student cases of SARS-CoV-2 infection associated with this large cluster through contact tracing data, with 30% sequencing coverage for further analysis. We estimated at least 11 independent introductions of SARS-CoV-2 into the student population, with four comprising the majority of detected cases and consistent with separate outbreaks. These four outbreaks were curtailed within a week following implementation of control measures. The impact of student infections on the local community was short-term despite an underlying increase in community infections. Our study highlights the need for context-specific information in the formation of public health policy for higher educational settings.

## Introduction

Severe acute respiratory syndrome coronavirus 2 (SARS-CoV-2), the causative agent of coronavirus disease 2019 (COVID-19), is persisting endemically in the UK with long-term implications for public health planning and preparedness. As at 2nd June 2022, 73% of the Scottish population aged 12 and over have received three doses of COVID-19 vaccine^1^. However, lower population immunity among children, adolescents and younger adults^2^, the occurrence of infection despite vaccination^3^, and the emergence of vaccine escape variants^4,5^ threaten the long-term ability of the national vaccination programme to suppress the spread of infection and prevent disease.

With the exception of the first SARS-CoV-2 epidemic wave in 2020, cases among children, adolescents, and young adults have represented a high fraction of confirmed cases in Scotland. Younger age groups may contribute to seasonal patterns of COVID-19 in the long-term, with annual outbreaks in educational settings a well-recognised feature of acute respiratory infections in temperate climates^6^. Although we note that large and frequent COVID-19 clusters are also associated with settings involving older adults such as leisure, sporting events, and occupation^7–9^.

With respect to higher education settings, the risk of respiratory infection outbreaks have been brought to the forefront by COVID-19, with the implementation of social distancing and remote learning to limit cases^10^. Universities present a unique epidemiological scenario for study, with the movement of students to and from domicile locations at the start and end of academic terms presenting a risk for introducing viruses into student populations and for spillover into local communities^11^. Theoretical models suggest that SARS-CoV-2 will more easily transmit in denser populations depending on vaccination rates, adherence to social distancing, and effectiveness of contact tracing and isolation measures^12,13^. With a potential increased likelihood of asymptomatic infection^14^ and lower vaccination coverage^1^, student populations are at risk of seeding outbreaks among their social groups as well as exposing the local community and non-term-time contacts. Reports highlighted the potential for a large number of cases in higher education settings, particularly in association with densely populated residential halls^15,16^.

Expansive SARS-CoV-2 genomic surveillance conducted in the UK, with over 300,000 whole genome sequences generated by June 2022 in Scotland alone, provides a means to characterise outbreak risks in a setting-specific manner. In combination with epidemiological data, genomic surveillance has enabled fine-grain characterisation of SARS-CoV-2 outbreaks in other semi-closed settings such as hospital wards^17,18^ and care homes^19^. To date one study in the UK has taken such an approach to understand SARS-CoV-2 outbreaks in a tertiary education setting^15^. This study identified persistent transmission among students, though with little evidence of transmission to the local community. However, the social and community contexts of universities in the UK differ, such that patterns of spread within a collegiate university, a single institution organised into self-governing colleges with associated accommodation, may not be directly applicable to universities with a more centralised organisational structure.

Here we use genomic epidemiology to investigate a large cluster of cases among University of Glasgow (UoG) students in Scotland, UK, arising in the autumn of 2020 at the start of the 2020/21 academic year^20^. At this point reported case numbers were relatively low, although with the second epidemic wave in Scotland already underway^21^. Our study provides a genomically-informed insight into epidemiological risks associated with SARS-CoV-2 outbreaks in a university accommodation setting in which students were resident in halls integrated with the local community in the city of Glasgow.

Our study contributes to a comprehensive understanding of the role that residential accommodation can play in the spread of SARS-CoV-2, and highlights the success of rapidly implemented control measures on mitigating any subsequent impact on the wider university population and the local community. Given the current lower 3rd dose vaccine uptake among young adults in Scotland (as at June 2022)^1^, and in the event of a novel SARS-CoV-2 variant of concern, there is a need for public health planning and policy formation to consider the higher educational setting, particularly in preparation for winter seasons. We propose that consideration is given to the varying accommodation, academic, and social contexts across student populations.

## Results

### Outbreak investigation

As of the 2020/21 academic year, the UoG hosts approximately 33,000 students from more than 140 countries, with 97% of undergraduate students aged 16–29 years, and with 58% of the 6100 new undergraduate students domiciled in Scotland^22^. The university provides multiple residential halls predominantly housing undergraduate students^23^.

**Figure 1:**
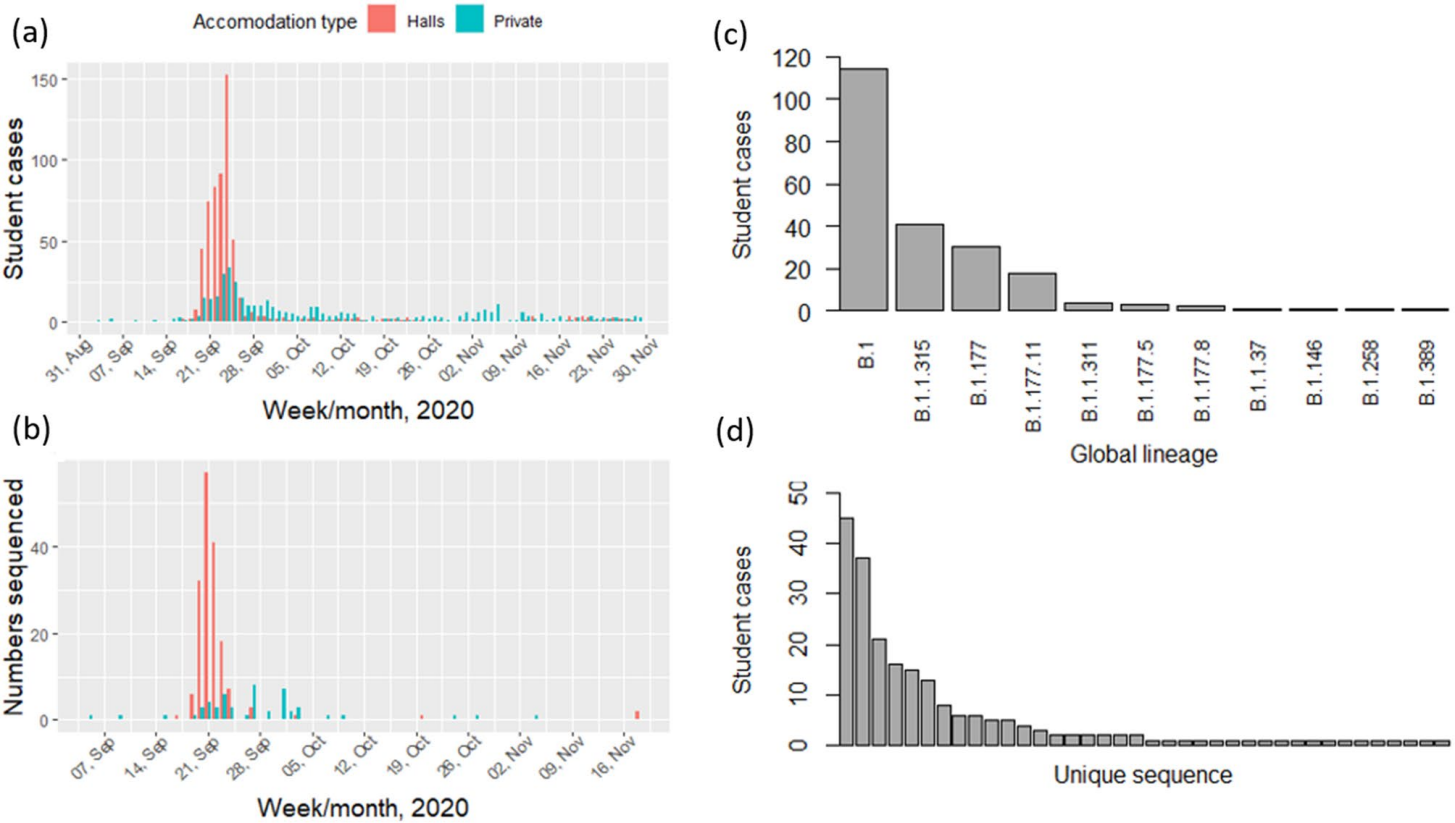
(**a**) Numbers of University of Glasgow student cases identified from contact tracing information over time, and (**b**) for the subset with sequenced samples, according to university residential halls or private accommodation. (**c**) Frequency distributions of SARS-CoV-2 lineages and (**d**) unique sequences identified among the student cases.

A cluster of COVID-19 student cases associated with two geographically distinct residential halls was identified by the UoG and the NHS Greater Glasgow and Clyde (NHSGGC) Public Health Protection Unit at the start of the 2020/21 academic year, immediately following Freshers’ week (12th to 18th September) (Fig. 1a). The outbreak peaked and was subsequently curtailed within around seven days of being identified, following the implementation of non-pharmaceutical control measures commonly in use at the time for controlling SARS-CoV-2; including infection control, contact tracing, increased availability of testing, and support for students to enable self-isolation.

A total of 1039 student SARS-CoV-2 positive cases were identified, with 616 (59.3%) resident at one of six university residential halls spanning 13 postcode locations. The remaining 423 (40.7%) students were deemed to reside in private accommodation (see “Methods”). The median case age was 18 years (IQR 18–19 years; range 16–51 years), consistent with a majority undergraduate population, and 57.2% (n = 591) were female consistent with a known gender skew among the wider student population^24^. Of the 616 student cases associated with residential halls, 519 (84.2%) had respiratory specimen dates from 19th September to 26th September 2020 spanning the residential halls cluster. Based on these data, the student cluster included around 9% of the new undergraduate population overall. SARS-CoV-2 whole genome sequences were available for 220 cases, with specimen dates spanning 5th September to 18th November 2020; 73.2% were from students residing in university residential halls with specimen dates from 19th September to 26th September 2020 (Fig. 1b). The median age among sequenced cases was 18 years (range 17–25 years, IQR 18–19 years) and 138 (62.7%) were female.

### SARS-CoV-2 genetic diversity

Among the 220 student cases, multiple global lineages (Pango nomenclature)^25,26^ were detected (Fig. 1c), the most common being B.1 (n = 114, 52.8%), B.1.1.315 (n = 41, 19.0%), B.1.177 (n = 30, 13.9%) and B.1.177.11 (n = 18, 8.3%). Seven further Pango lineages were detected at low numbers (n = 13, 5.9%). A total of thirty-nine distinct SARS-CoV-2 sequences were identified (Fig. 1d).

**Figure 2:**
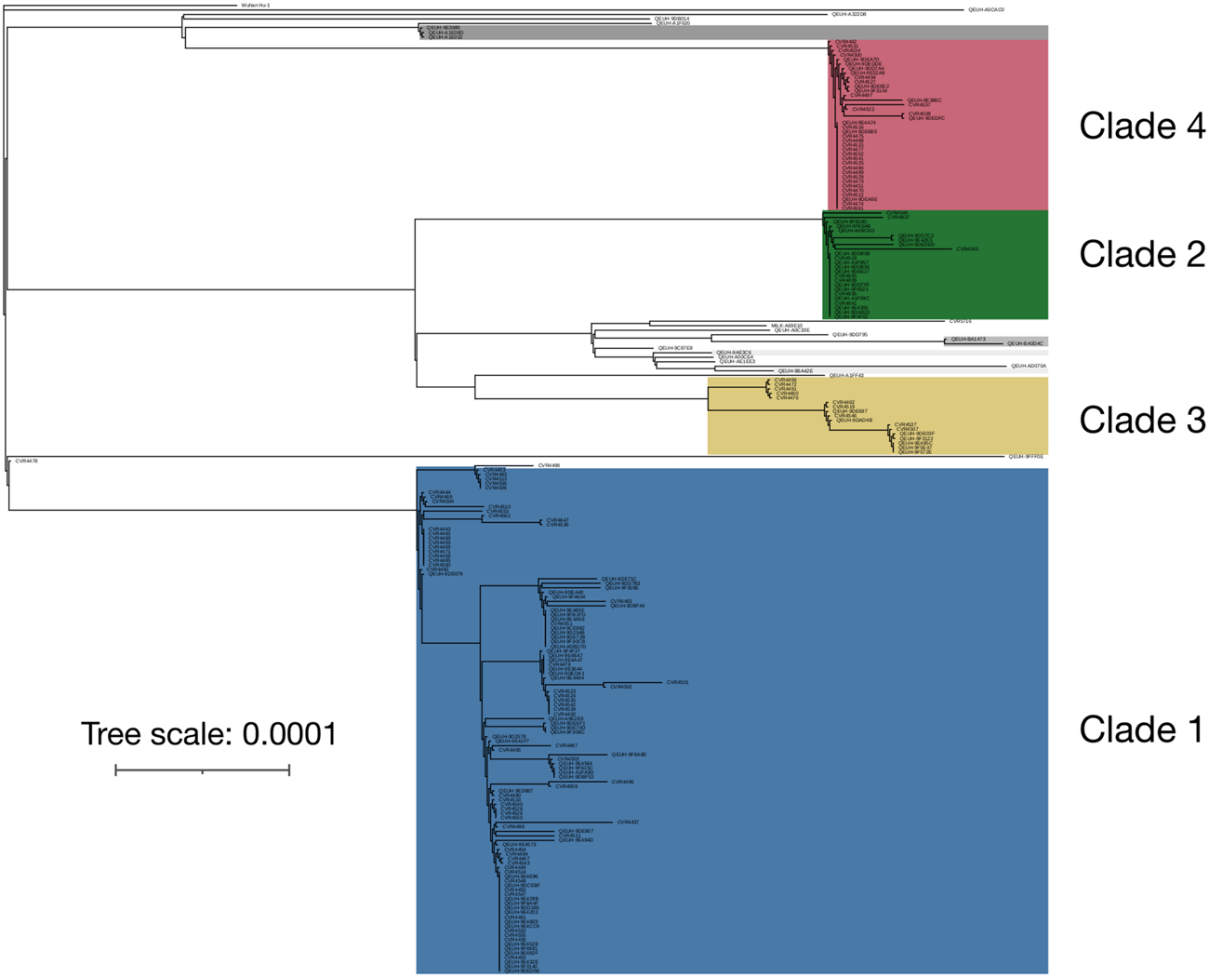
Phylogenetic tree of high quality genome sequences collected from University of Glasgow students. The reference sequence Wuhan Hu-1 (GenBank accession MN90894) was included as an outgroup. Four notable clades of sequences were identified, corresponding to cases that were putatively linked via direct transmission, as identified by the A2B-COVID software package. Shades of gray indicate three other sets of putatively linked cases. The phylogenetic analysis was conducted using iQ-Tree2^38^, while the figure was made with iTOL^44^. The tree scale indicates the fraction of sites in the genome at which substitutions were observed. The tree scale bar denotes nucleotide substitutions per site.

Putative transmission clusters were identified from 214 high quality SARS CoV-2 genome sequences (see “Methods”) using the A2B-COVID software package^17^. We use the term ‘transmission cluster’ to denote a set of potentially linked cases consistent with having arisen from a single introduction into the student population (see “Methods”; Supplementary Fig. S1, supplementary material). Of the 214 high quality sequences, 157 were obtained from students resident in halls with specimen dates spanning 19th to 26th September 2020, providing 30% coverage of the student halls COVID-19 cluster. Four large transmission clusters were identified, comprising 191 sequences; mapping these onto a phylogenetic tree identified them with the four clades (highlighted in Fig. 2). Among the remaining 23 sequences, three further putative transmission clusters were found, each comprising two or three cases, alongside 16 sequences that were not positioned closely with other transmission clusters.

While each putative transmission cluster is consistent with describing a distinct introduction into the student population, a more conservative estimate was made that at least 11 distinct introductions of SARS-Co-2 had occurred. To derive this, each transmission cluster and any sequences descended from it was counted as a single introduction. Further, the sequences intermediate to transmission clusters 2 and 3 were regarded as potentially arising from a single introduction (Fig. 2). In either case, the majority of introductions of SARS-CoV-2 into the university failed to cause substantial onward transmission among students.

### Genomic evidence is consistent with introductions from within and outside Scotland

**Figure 3:**
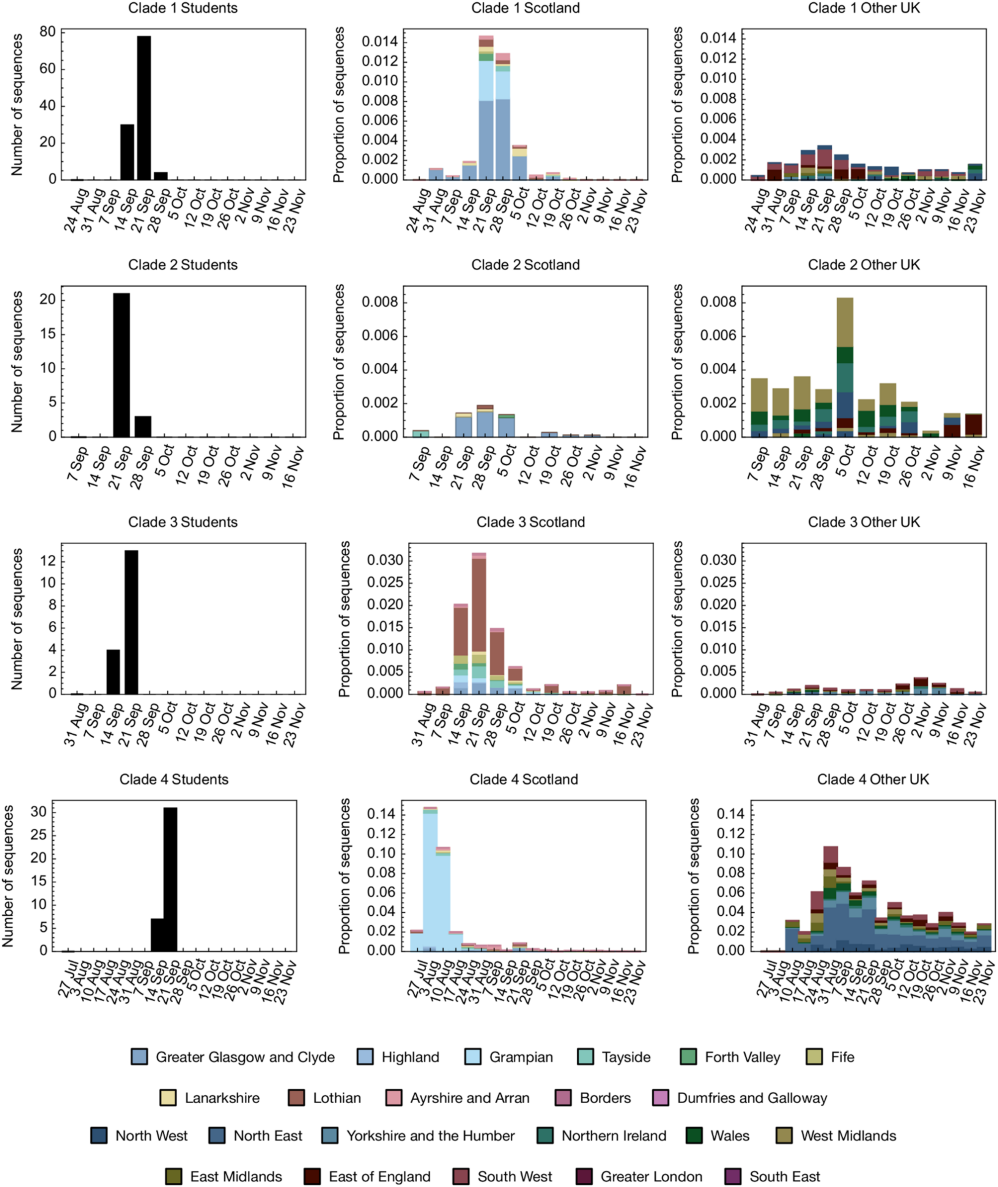
Potential origins of major SARS-CoV-2 clades identified in the University of Glasgow (UoG) student population. Numbers of UoG student sequences associated with each major clade (left column) are shown alongside data describing the associated UK-wide clades. Figures show the proportion of SARS-CoV-2 sequences collected each week in Scotland (middle column) and in the rest of the UK (right column) that were associated with each student clade, coloured by the regions in which the clade-associated sequences were collected. Total numbers of sequences informing this analysis were as follows: Clade 1 n = 412; Clade 2 n = 255; Clade 3 n = 541; Clade 4 n = 4006.

Determining the most recent common ancestor of the sequences associated with the four main transmission clusters positioned them within more expansive UK-wide phylogenetic clades circulating at that time. These clades combined both student and community sequences and revealed potentially distinct origins for the introductions to the University forming four main transmission clusters. Investigating the proportion of genome sequences falling into each of the four clades by regional location on a weekly basis, we found differences in the proportional representation of each clade when comparing Scotland with other UK regions in the period before the UoG residential halls cluster (Fig. 3).

Recent information from the Higher Education Statistics Agency estimates that around 84% of Scottish university students from within the UK are domiciled within Scotland (Supplementary Table S1, supplementary material). This compares to between 35 and 63% of students in England domiciled within the region of their higher education provider, 62% in Wales, and 93% in Northern Ireland. For UoG students specifically, 81% were domiciled in Scotland, with the percentage of students resident across the remaining UK regions ranging from 0.4 to 3% (see Supplementary Table S2, supplementary material). Employing a simple model taking into consideration the high percentage of UoG students domiciled within Scotland (Supplementary Tables S1–S2, supplementary material), we estimated a relatively high probability of Scottish relative to English origin for clades 1 and 3 (90:10 and 99:1 respectively), although these clades were not notably prevalent in Scotland in the period before the residential halls cluster (Fig. 3). Evidence was inconclusive for clades 2 and 4 (probability ratio of 60:40 in both cases) (see “Methods”).

Sequences from five residential halls (43 cases), and private accommodation (5 cases), were genetically indistinguishable from sequences identified from outbreaks associated with six other Scottish universities (Supplementary Fig. S4, supplementary material). These data may point towards acquisition of infection in Scottish domicile locations prior to the new academic term, as is the apparent case for at least two of the four clades.

The small number of UoG-outbreak-associated clades detected following the outbreak is consistent with this group of student cases having no long-term impact on the persistence of SARS-CoV-2 in the Scottish community.

### Genetic diversity across halls suggests a high degree of student mixing

**Figure 4:**
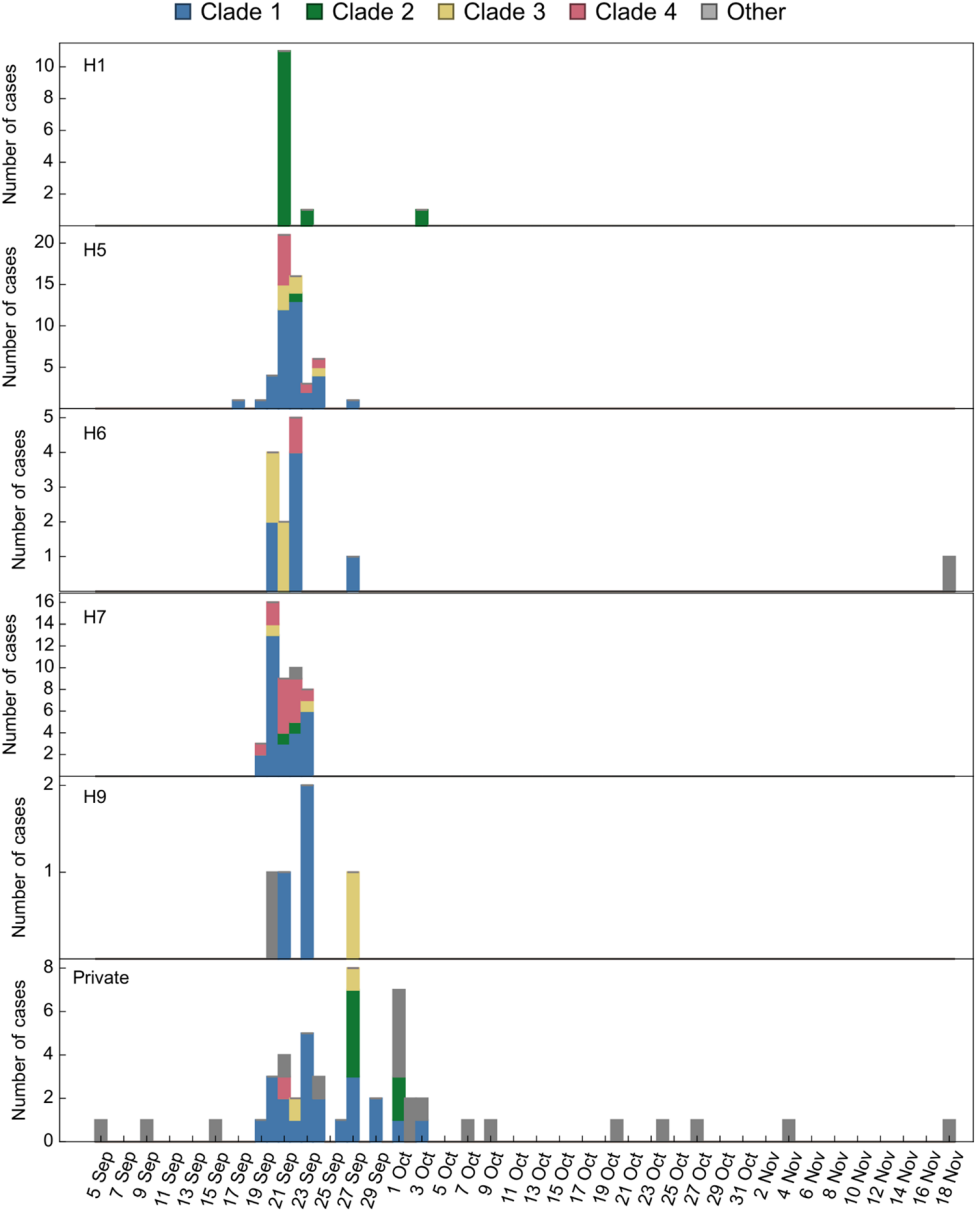
Dates of sequenced PCR-confirmed cases of SARS-CoV-2 infection identified in University of Glasgow students resident in halls or private accommodation, coloured by transmission clade. Data is shown for residential halls in which a minimum of four cases were observed. The identification of viruses from multiple transmission clusters in many of the residential halls suggests that there were multiple introductions of SARS-CoV-2 into each hall.

Sequences from each of the four major transmission clusters were found distributed across multiple residential halls and among students resident in private accommodation (Fig. 4). This picture is consistent with student-to-student transmission, either within residential halls and/or alongside common sources of exposure. The observation of mixed viral populations within most residential halls is consistent with each having had multiple SARS-CoV-2 introductions. Observations of viruses from different transmission clusters were seen in rapid succession within halls, making it difficult to identify the location of the first case in each and suggesting extensive mixing between students prior to the outbreaks. The rapid drop in cases suggests that transmission within these halls was quickly contained.

Cases infected with SARS-CoV-2 other than the four major clades were rarely associated with residential halls, with the vast majority resident in private accommodation (Fig. 4).

### Evidence suggests a short-lived impact on local community case incidence

**Figure 5:**
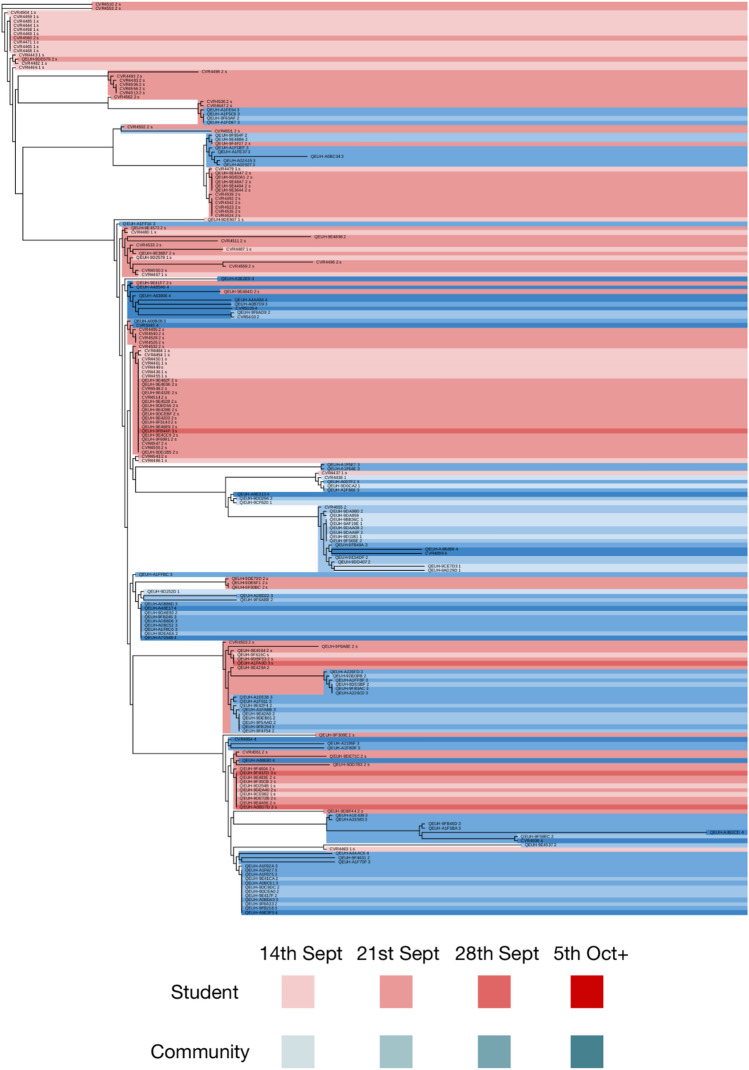
Phylogenetic relationship between student (red) and community (blue) sequences associated with transmission clade 1. Shades indicate the week in which sequences were collected, the lightest colour indicating dates before and including the week of 14th September, and the darkest colour indicating dates from the 5th October onwards. Community sequences describe those identified in NHS Greater Glasgow and Clyde sharing a common ancestor with the student sequences. Student and community cases were observed in multiple subclades of the tree, indicating ongoing transmission between the students and local community. Phylogenetic analysis was conducted using iQ-Tree2^38^, while the figure was made with iTOL^44^.

An analysis of student and community sequences showed considerable intermixing of cases, with multiple subclades of a constructed phylogenetic tree spanning both populations (Fig. 5). Sequences used for this analysis comprised the 112 student sequences from transmission cluster 1, and additionally the 104 sequences in the associated UK-wide phylogenetic clade that originated within NHSGGC. While our data are insufficient to derive accurate rates or directionality of transmission, the data are consistent with ongoing mixing of students with others in the local community, though with no long-term impact in terms of persisting chains of transmission.

**Figure 6:**
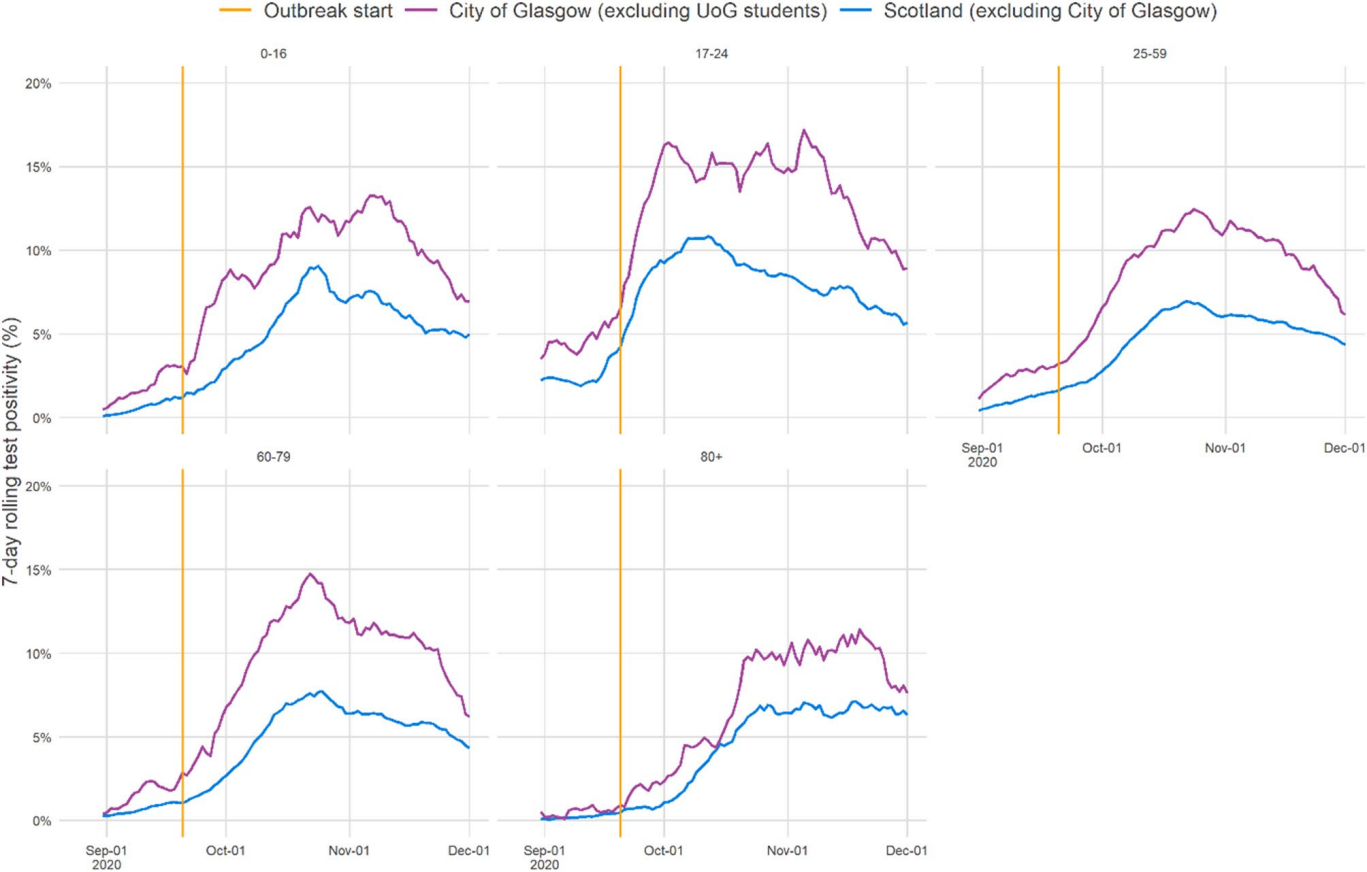
Age-stratified 7-day rolling proportions of PCR-confirmed cases of SARS-CoV-2 infection (test positivity) among all tested individuals in the City of Glasgow community [excluding University of Glasgow (UoG) students], and in comparison with the rest of Scotland, excluding the City of Glasgow. The vertical lines indicate the start of the UoG student halls cluster around 19th September 2020.

**Figure 7:**
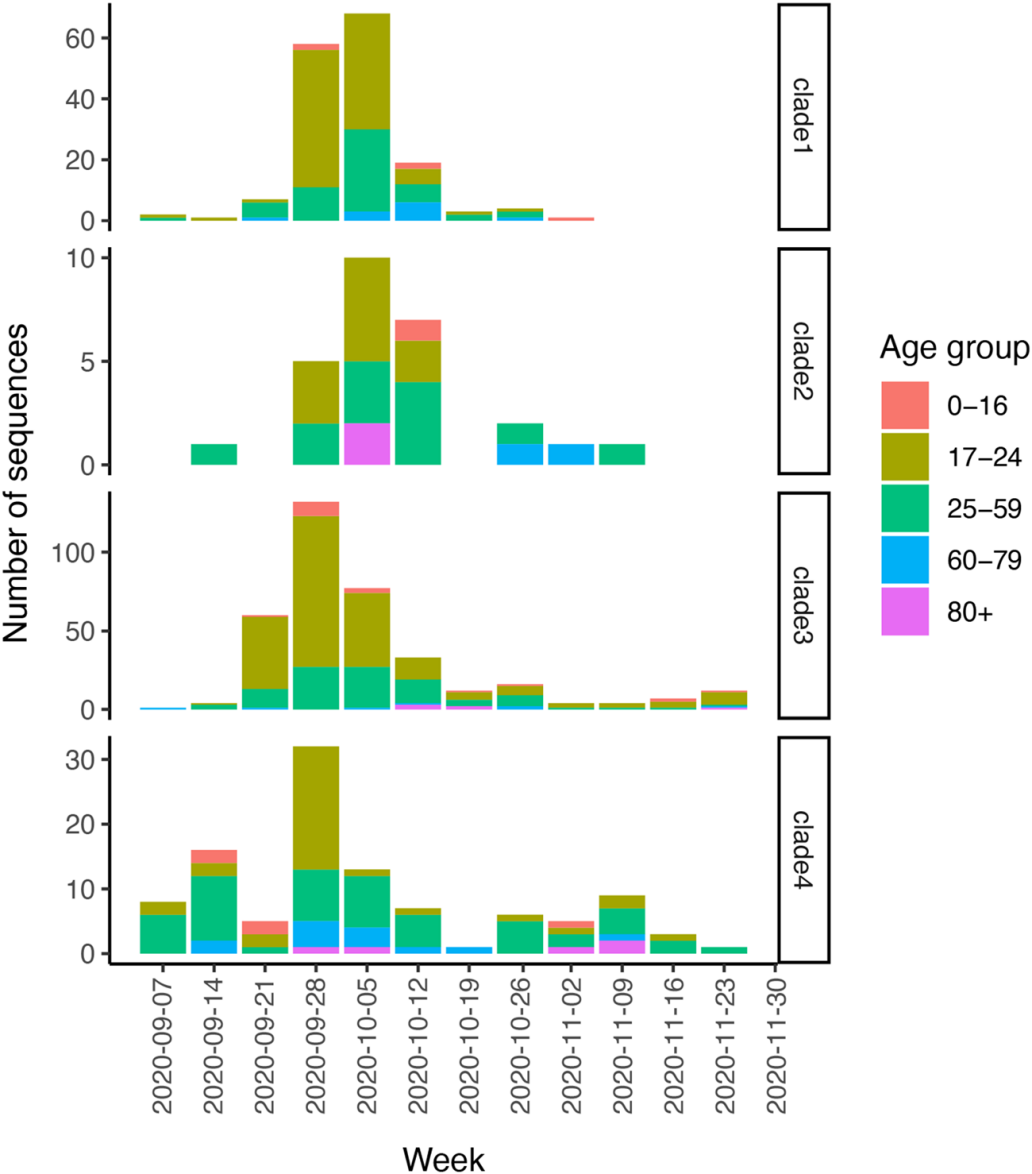
Age distribution of SARS-CoV-2 cases among residents of NHS Greater Glasgow and Clyde with sequences that phylogenetically clustered with each University of Glasgow (UoG) student transmission clade. UoG student cases were excluded. Numbers of sequences informing this analysis were as follows: Clade 1 n = 163, Clade 2 n = 27, Clade 3 n = 362, Clade 4 n = 106.

Age-stratified proportions of SARS-CoV-2 infections identified among PCR-tested individuals in the City of Glasgow (excluding UoG students) showed a rise in cases among adults aged 17–24 years coinciding with the start of the UoG residential halls cluster (Fig. 6). Furthermore, the age distribution of sequenced community cases identified in NHSGGC falling into UK-wide clades associated with the four major student transmission clusters showed a similar bias towards 17–24 year olds, consistent with mixing of students and the local community occurring predominantly among this age group (Fig. 7). The rise in the proportion of positive tests among 17–24 year olds was also seen on average across the rest of Scotland (Fig. 6), suggesting the burden of cases in this age group, with the potential impact of student populations at the start of term, was not unique to the City of Glasgow.

## Discussion

We combined genome sequence data with epidemiological information to investigate a cluster of COVID-19 cases associated with UoG student residential halls. These cases occurred prior to rollout of the UK’s COVID-19 vaccination programme and the use of lateral flow device tests for regular asymptomatic self-testing by the students. We evaluated frequency and origins of SARS-CoV-2 introductions into the student population, the likelihood of transmission among the students, and impact on the local community. The integration of genomic, evolutionary, and epidemiological approaches provides a deep insight into the nature of viral spread within a university accommodation context.

Our study highlights several important features of public health significance. Firstly, the increase in SARS-CoV-2 infections among the student population primarily involved university accommodation. The sudden rise in student cases emerged shortly after social events linked to “Freshers’ week” at the start of the university term, providing a strong indication that the outbreaks spread via induction events and/or other social activity and gatherings^27^.

Secondly, while there was evidence of at least 11 introductions of SARS-CoV-2 into the university student population, our data were consistent with only four leading to substantial onward transmission among students, each consistent with a separate outbreak. Our data suggests the presence of large variations in the numbers of individuals infected by each introduction, potentially attributable to superspreading events whereby a small number of individuals cause a disproportionate number of cases^28^. Although the sequencing of UoG student cases was incomplete, with around 30% coverage of the residential halls cluster, we can be reasonably confident that all major transmission clusters were detected.

Thirdly, the data were strongly indicative of introductions into the university from Scotland for at least two of the four clades. Our findings highlight that although the potential for virus importations into Scotland through long-distance student movements remains, the risk of new variants being introduced to Scotland at the start of the academic year would appear low in comparison to other UK regions given the high proportion of Scottish students domiciled within Scotland^29^. The risk of importing SARS-CoV-2 from countries outside of the UK was not directly addressed by our study owing to a lack of robust data at a global level.

Fourthly, our study highlights the rapid spread of SARS-CoV-2 within and between university accommodation. Halls of residence provide household-like conditions enabling direct and indirect transmission of SARS-CoV-2 via respiratory (aerosol and droplet) and fomite routes. Accommodation at the UoG comprises predominantly off-campus accommodation, representing a mixture of self-contained flats, shared kitchen spaces and bathroom facilities, and common room/study areas^23^. The identified transmission clusters were consistent with student–student transmission linked to household-like conditions and/or shared facilities within residential halls. Social gatherings may also be a conduit for the apparent transmission between households and likely contributed to the multiple viral populations within halls, with mixing of individuals returning to the university from various locations, and with compliance to social distancing expected to be poor at such events.

Despite the scale and speed at which the outbreak grew, the rapid curtailment of the outbreaks demonstrates that the swift action of the university and local Public Health Protection Unit, together with an apparent high degree of student compliance, prevented any further rise in case numbers and limited any impact on the local community. We note that a decline in proportional sequencing coverage in Scotland in the period subsequent to the UoG outbreaks (from an average daily coverage of ~ 24% in September 2020 to ~ 4% from mid-October 2020) limits the ability to detect associated variants circulating at low prevalence, and precludes a direct comparison of observed clade-specific case incidence rates between Scotland and England. However, we can be reasonably confident that variants associated with any significant outbreaks or long-lasting chains of transmission would be detectable among the community sequences available, given that sequencing was conducted daily over an extended time frame. It is likely that self-isolation by students was the predominant factor leading to the rapid end of the student halls-associated outbreaks, although our study did not formally evaluate control measure effectiveness.

At the time of the UoG outbreaks, most educational institutes in the UK had undergone emergency closure of in-person teaching in response to the COVID-19 epidemic, resulting in prolonged periods of remote learning. However, significant outbreaks arose in the university setting, albeit during a period of relatively high susceptibility and prior to rollout of the national vaccination programme. In contrast to 2020/21, numbers of university student cases were comparatively lower in Scotland at the start of the 2021/22 academic year^30^ in the absence of university closures and restriction of social activities. This period saw a decreasing case incidence nationally alongside the accumulation of immune protection via vaccination and naturally-acquired immunity, and with the introduction of self-testing by asymptomatic students using lateral flow device tests^1,21,31^. The opening of educational settings during periods of low incidence may be an important component of risk mitigation^32^, alongside promotion of safer socialisation practices and enhanced social care and support structures^33^.

The results from our study contrast with those reported for an outbreak among students at the University of Cambridge (UoC)^15^. UoC is an atypical UK higher educational institution in that in addition to education, accommodation and the social setting among students revolves around independent colleges. By contrast, UoG is far more centralised and integrated, albeit with university accommodation being more geographically intertwined with the local community. In Cambridge, the outbreak was dominated by a single large cluster that persisted until the national lockdown in November 2020. That the UoG residential halls were geographically restricted likely enabled greater control and enforcement of isolation measures through provision of student support. The outbreaks associated with UoG did however show greater interaction between students and the broader community than observed for UoC, with community and student cases in the largest clade being substantially mixed. The contrasting findings across studies provides invaluable context in understanding the role of SARS-CoV-2 spread within the university setting. Differences in university organisation and housing structure are likely to have a pronounced impact on the potential spread of respiratory viruses, and the physical location of students within a city can affect both the nature and impact of an outbreak.

Our study has some important limitations. Firstly, a comparison of infection risks associated with student social activities, or the household conditions and/or shared facilities within residential halls, was not possible. Secondly, interpretation of direct student-to-student transmission events is limited by lack of information on how sequenced cases were linked to individual households within a residential hall, and the low levels of variation in SARS-CoV-2 genomes make it difficult to conclusively define epidemiological links. Thirdly, the analysis of the origins of the university student associated clades does not consider the potential for the introduction of cases into the student population via international travel. Fourthly, it is possible that some student cases were misclassified as community cases. Fifth, we note that the presence, frequency and diversity of SARS-CoV-2 may be biased by the targeted sampling of outbreaks, particularly during periods of low sequencing coverage. Some targeted sequencing of the UoG student outbreak had occurred to assist with initial investigations in near real-time. Finally, only 30% of identified student cases were sequenced. We have aimed to provide conservative estimates throughout.

These analyses have provided valuable insight into a large outbreak in an off-campus university accommodation setting, and an evidence base to inform future policy recommendations for students returning to universities at the start of terms. The higher education setting presents a risk for contributing to the winter burden of COVID-19. With rapid identification, and implementation of non-pharmaceutical control interventions, the impact of outbreaks on local communities may be limited. However, while high rates of vaccination have moderated the impact of SARS-CoV-2 disease severity on the UK population, it is clear SARS-CoV-2 will continue to evolve in the human population such that student populations, as with all demographics, should be encouraged to be up to date with vaccinations (currently three doses in under 75 year olds as of mid-2022).

## Methods

### Epidemiological investigation

A retrospective search was performed in a database of NHS Scotland Test and Protect contact tracing interviews, known as the Case Management System. The case definition pertaining to a University of Glasgow (UoG) associated student was applied as follows: the text “Glasgow” and “university or uni” under occupation related fields, and with RT-PCR diagnostic test confirmation of a SARS-CoV-2 infection between 1st September 2020 and 30th November 2020. Free text notes were inspected and cases were excluded if determined to not be a University of Glasgow student. Residential postcodes were then matched to UoG student halls postcodes obtained from the Scottish Government Advanced Learning and Skills Analysis (ALSA), identifying cases resident in university accommodation. Further cross checking was performed: student status was corroborated by the individual’s age (with the majority of student cases ≤ 19 years) and/or an additional interrogation of free text notes. Four cases had missing age information. We note that our approach is relatively conservative, and the number of student cases identified may differ from that based on other methods (such as reported in Ref.^20^).

### Student SARS-CoV-2 whole genome sequences

In Scotland, SARS-CoV-2 genome sequencing is coordinated by the NHS Sequencing Service. Respiratory specimens collected through routine RT-PCR NHS diagnostic testing and UK Government community testing were submitted to The COVID-19 Genomics UK Consortium (COG-UK) partnership laboratories at the MRC-University of Glasgow Centre for Virus Research and Sanger Institute for sequencing. The genomic sequence data were uploaded to CLIMB (Cloud Infrastructure for Microbial Bioinformatics)-COVID^34^ and amalgamated with all Scottish sequences. The UoG student case list was linked to Scottish genomic sequence data derived from respiratory specimens collected between 1st September 2020 and 30th November 2020 using a unique identifier. Out of a total 220 identified sequences, 214 were considered high quality for further analysis, with > 80% nucleotide coverage across the viral genome. Four sequenced cases had specimen dates preceding 19th September.

### Inference of transmission clusters among UoG students

Putative transmission clusters were inferred using the A2B-COVID software package^17^. Given symptom onset dates and SARS-CoV-2 genome sequences from multiple individuals, this carries out a series of hypothesis tests, calculating whether the data from each pair of individuals A and B is statistically consistent with a model of A having directly infected B; data are assessed as being consistent with transmission, borderline, or unlikely to have arisen from a direct transmission event. Our model utilises distributions for the infectivity profile and time to symptom onset described for SARS-CoV-2 by other publications^35–37^. In the absence of symptom onset dates, respiratory specimen collection dates were used as proxy estimates. A sub-setting procedure was used to generate putative transmission clusters, placing two individuals in the same cluster if transmission between those two individuals in either direction was not unlikely.

Phylogenetic inference of UoG student sequences was then conducted using the IQ-Tree2 package^38^ in order to characterise the transmission clusters in terms of phylogenetic clades. Inferences used ModelFinder to identify the most appropriate model in each case^39^. The trees of Figs. 2 and 5 were thus inferred using a TIM2 model with empirical base frequencies and allowing for a proportion of invariable sites.

### Inference of transmission clade origins

The sampling locations of sequences in each clade were used to determine the likely origin of each transmission cluster. Viral genome sequence data were then used to estimate the probability that each of the four major clades we identified arose from an introduction into the student population either from England or Scotland. To derive an estimate of the probability that the introduction of a clade C came from England, we first estimated the fraction of genome sequences of clade C in England and Scotland in the two weeks prior to the observation of the first student case in that clade:

$${Q}_{S,C}=\frac{{g}_{S,C}}{{G}_{S}},$$

and

$${Q}_{E,C}=\frac{{g}_{E,C}}{{G}_{E}},$$

where g_S,C_ and g_E,C_ were the number of virus genome sequences in clade C collected in Scotland and England respectively, during the two weeks prior to the first observation of the clade in students at the UoG, while G_S_ and G_E_ were the total number of virus genome sequences collected in Scotland and England during the same period.

Using these values, we estimated the prevalence of each clade in Scotland and England to inform the probabilities P_S,C_ and P_E,C_ that a given individual in either Scotland or England respectively was infected with SARS-CoV-2 from clade C:

$${P}_{S,C}=\frac{{{Q}_{S,C}I}_{S}}{{N}_{S}},$$

and

$${P}_{E,C}=\frac{{{Q}_{E,C}I}_{E}}{{N}_{E}},$$

where I_S_ and I_E_ were the mean total numbers of positive tests reported per week in Scotland and England during the 2 weeks in question, as reported in UK Government statistics^40^, while N_S_ and N_E_ are ONS mid-2020 population estimates for Scotland and England^41^. Given these values, we estimated the probability that a student arriving in Glasgow was infected with SARS-CoV-2 of clade C and domiciled in either Scotland (*X*_*S,C*_) or England (*X*_*E,C*_). For *X*_*S,C*_ this is given by

$${X}_{S,C}=\frac{{P}_{S,C}{P}_{S,U}}{{P}_{S,C}{P}_{S,U}+{P}_{E,C}{P}_{E,U}},$$

where P_S,U_ and P_E,U_ are the probabilities that a given student at UoG was domiciled in Scotland or England respectively. The equivalent value *X*_*E,C*_ that the student was domiciled in England is given by *X*_*E,C*_ = 1 − *X*_*S,C*_.

In our calculation we neglected the possibility of introductions from outside of either England or Scotland due to the lack of data from other locations. Our measure is approximate, being prone to sampling bias and limited data, but was used to provide a broad indication of the geographical origins of clades.

### Phylogenetic inference for NHSGGC sequences

The COG-UK phylogeny for SARS-CoV-2 was produced using CLIMB-COVID data available on the 2020–12-31. The grapevine pipeline^42^ was then used for extracting the smallest phylogenetic clade containing the sequences in each putative transmission cluster. This was used to put the SARS-CoV-2 genome sequences from UoG students in the broader context of UK wide sequences. The clades were pruned to include only sequences from UoG students and NHSGGC and students from other Scottish Universities determined through contact tracing. For visualisation of these clades, identical sequences were collapsed and the figure was produced in The Environment for Tree Exploration^43^.

### Local epidemiological context in the City of Glasgow

All individuals with respiratory specimens collected for diagnostic testing of SARS-CoV-2 infections by RT-PCR in Scotland between 1st September 2020 and 30th November 2020 were identified from the national Electronic Communication of Surveillance in Scotland (ECOSS) database. Age-stratified 7-day rolling proportions of SARS-CoV-2 positive cases, among all RT-PCR-tested individuals, were estimated for the City of Glasgow, excluding cases with residential postcodes associated with UoG residential halls, in a conservative approach to removing the influence of UoG students from the analysis. A comparison was made with the rest of Scotland, excluding individuals resident in the City of Glasgow. The 17–24 years group represent the age range of the UoG residential halls-associated cases.

### Ethics declaration

This study was undertaken as part of public health surveillance activity within the COVID-19 programme of Public Health Scotland, in line with the necessary associated regulations and guidelines. The retention and processing of information on individuals is conducted by Public Health Scotland as part of COVID-19 surveillance in Scotland in the context of emergency data processing (https://www.informationgovernance.scot.nhs.uk/covid-19-privacy-statement/), including the Civil Contingencies Act 2004, the NHS (Scotland) Act 1978 and the Public Health (Scotland) Act 2008, and under Articles 6(1)(e), 9(2)(h), 9(2)(i), 9(2)(j) of the General Data Protection Regulation. Surveillance data was shared with NHS Scotland according to the Intra NHS Scotland Data Sharing Accord (https://www.informationgovernance.scot.nhs.uk/wp-content/uploads/2020/06/2020-06-17-Intra-NHS-Scotland-Sharing-Accord-v2.0.pdf). Ethics approval was not required for this work which was based on pre-existing routine surveillance data for the Scottish population.

## Supplementary Information

Supplementary Information 1.

Supplementary Information 2.

Supplementary Information 3.

## Data Availability

The SARS-CoV-2 genome sequences used in this study are available from GISAID with identifiers provided in supplementary material. Other healthcare data are available upon application to the NHS Scotland Public Benefit and Privacy Panel For Health and Social Care: https://www.informationgovernance.scot.nhs.uk/pbpphsc/. The A2B-COVID tool is available from https://chjackson.github.io/a2bcovid/.
