# Supplementary material for: Genomic epidemiology of SARS-CoV-2 in a university outbreak setting and implications for public health planning
Source: Sci Rep. 2022 Jul 19;12:11735. doi: 10.1038/s41598-022-15661-1 (PMC9296497; doi:10.1038/s41598-022-15661-1)
Supplement: Supplementary file 1 — Supplementary Information 1. [file 41598_2022_15661_MOESM1_ESM.docx]

**Genomic epidemiology of SARS-CoV-2 in a university outbreak setting and implications for public health planning**

Sema Nickbakhsh^1,2#*^, Joseph Hughes^1,3#^, Nicolaos Christofidis^1^, Emily Griffiths^1^, Sharif Shaaban^1^, Jessica Enright^4^, Katherine Smollett^3^, Kyriaki Nomikou^3^, Natasha Palmalux^3^, Lily Tong^3^, Stephen Carmichael^3^, Vattipally B. Sreenu^3^, Richard Orton^3^, Emily J. Goldstein^5^, Rachael M. Tomb^5^, The COVID-19 Genomics UK (COG-UK) Consortium^6§^, Kate Templeton^7^, Rory N. Gunson^5^, Ana da Silva Filipe^3^, Catriona Milosevic^8^, Emma Thomson^1,3^, David L. Robertson^1,3^, Matthew T.G. Holden^1,9,^ Christopher J.R. Illingworth^3,10,11✝*^, Alison Smith-Palmer^1✝^

Affiliations

^1^Public Health Scotland, Meridian Court, 5 Cadogan Street, Glasgow G2 6QE, United Kingdom

^2^Institute of Biodiversity, Animal Health & Comparative Medicine, University of Glasgow, Graham Kerr Building, Glasgow, G12 8QQ, United Kingdom

^3^MRC-University of Glasgow Centre for Virus Research, 464 Bearsden Road, Glasgow, G61 1QH, United Kingdom

^4^School of Computing Science, University of Glasgow, 18 Lilybank Gardens, Glasgow, G12 8RZ, United Kingdom

^5^West of Scotland Specialist Virology Centre, NHS Greater Glasgow and Clyde, Glasgow Royal Infirmary, New Lister Building, Glasgow, G31 2ER, United Kingdom

^6^https://www.cogconsortium.uk

^7^Royal Infirmary of Edinburgh, NHS Lothian, 51 Little France Crescent, Edinburgh EH16 4SA, United Kingdom

^8^NHS Greater Glasgow and Clyde, Gartnavel General Hospital, 1055 Great Western Road, Glasgow, G12 0XH, United Kingdom

^9^School of Medicine, University of St Andrews, North Haugh, St Andrews, KY16 9TF, United Kingdom

^10^Department of Applied Mathematics and Theoretical Physics, University of Cambridge, Cambridge, United Kingdom

^11^MRC Biostatistics Unit, University of Cambridge, East Forvie Building, Forvie Site, Robinson Way, Cambridge, CB2 0SR, United Kingdom

^#^Shared first authors. ^✝^Shared senior authors. ^§^Full list of consortium names and affiliations are in the appendix.

*Corresponding authors: Dr Sema Nickbakhsh: [Sema.Nickbakhsh@phs.scot](mailto:Sema.Nickbakhsh@phs.scot); Dr Christopher Illingworth: [Christopher.Illingworth@glasgow.ac.uk](mailto:Christopher.Illingworth@glasgow.ac.uk)


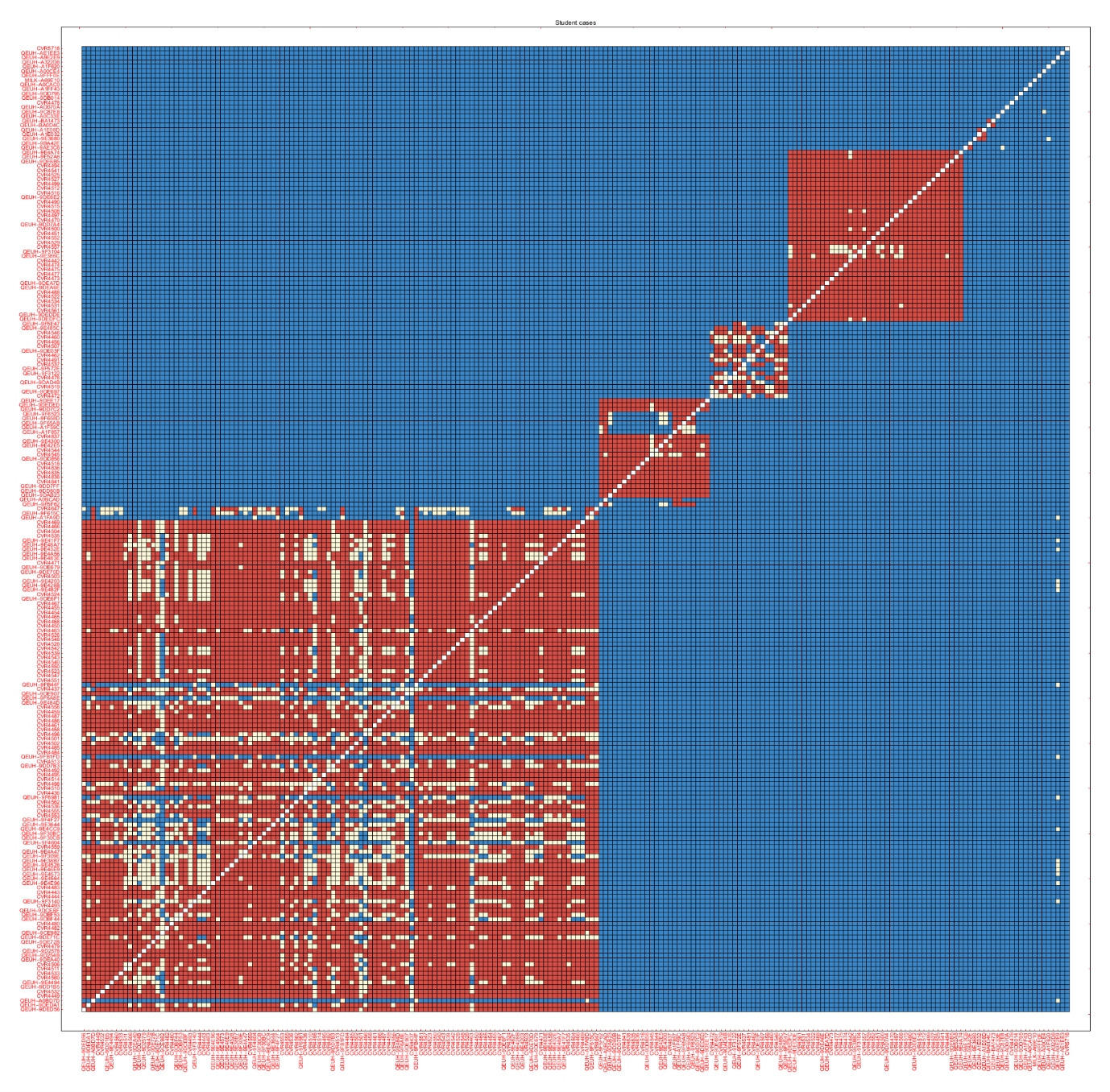
**Figure S1:** Analysis of viral genome sequences and dates of collection from students. A red square suggests that the data are consistent with one individual (vertical axis) directly infecting another with SARS-CoV-2 (horizontal axis). A blue square suggests that the same transmission is unlikely, while a yellow square indicates a “borderline” case. Clusters of cases on the diagonal indicate sets of cases that are consistent with a single introduction to the community. Statistics were calculated using the A2B-COVID software package ^17^.


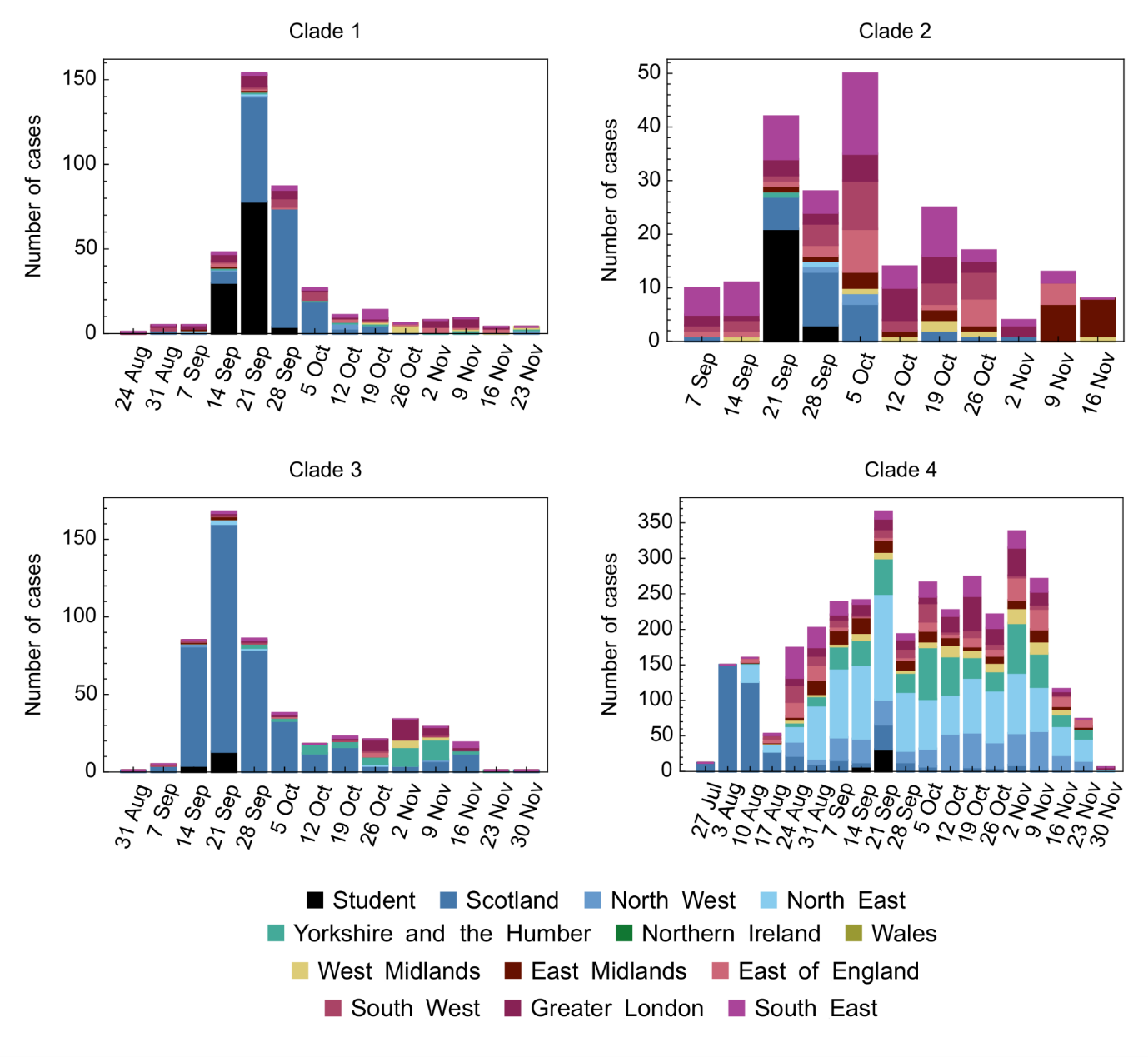
**Figure S2:** Location of PCR-confirmed cases of SARS-CoV-2 infection in the UK associated with the different clades identified among the sequence data collected from University of Glasgow (UoG) students. Cases from UoG students are shown in black, with other colours corresponding to community cases from different regions of the United Kingdom. Numbers of sequences informing this analysis are as follows: Clade 1 n=412, Clade 2 n=255, Clade 3 n=541, Clade 4 n=4006.


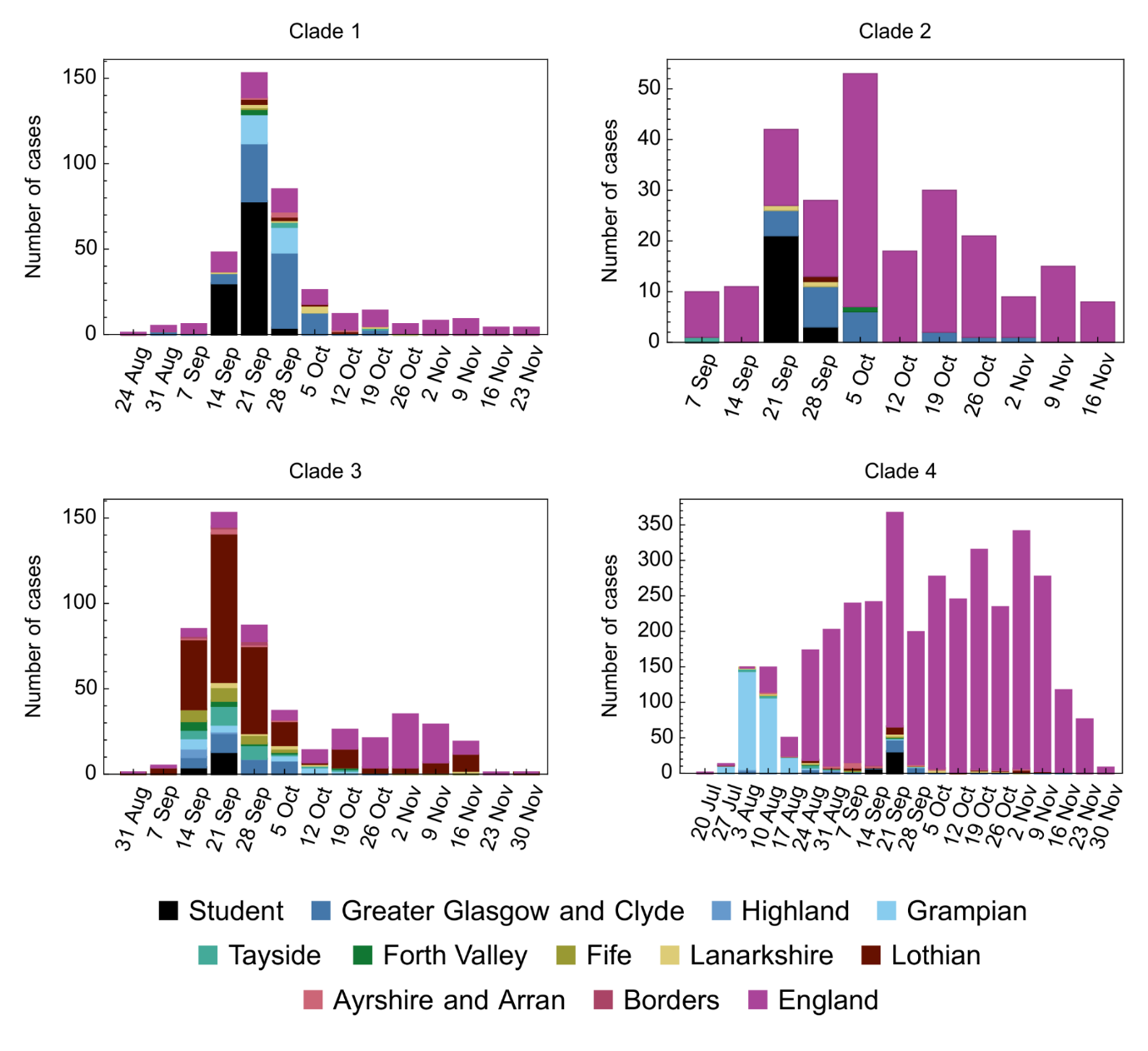
**Figure S3:** Location of PCR-confirmed cases of SARS-CoV-2 infection in the UK associated with the different clades identified among the sequence data collected from University of Glasgow students. Cases in Scotland were divided into geographical areas defined by each NHS Board. Numbers of sequences informing this analysis are as follows: Clade 1 n=412, Clade 2 n=255, Clade 3 n=541, Clade 4 n=4006.


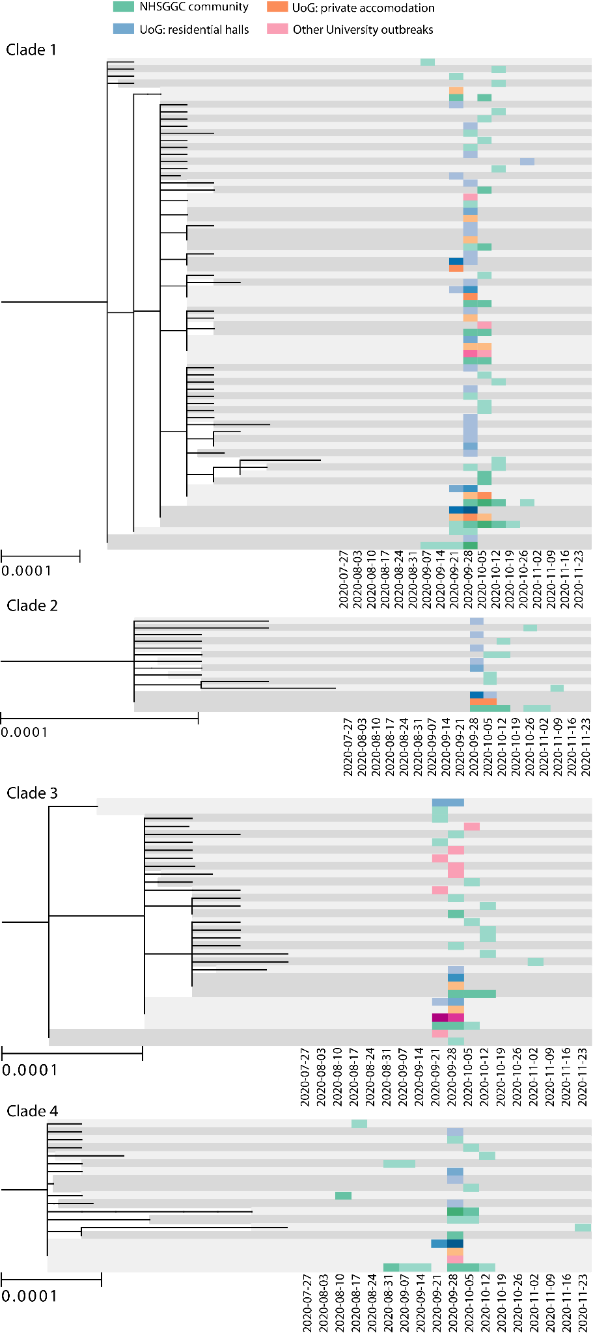
**Figure S4: Phylogenetic trees for sequences derived from cases of SARS-CoV-2 infection resident in NHS Greater Glasgow and Clyde (NHSGGC) for each transmission clade.** Sequences derived from different subgroups are highlighted by colour coding: University of Glasgow (UoG) students either resident in halls or private accommodation, and non-UoG associated cases deemed to be community cases. Colour shading from light-to-dark represents numbers of sequences per week from low-to-high. Sequences derived from cases associated with six other Scottish university outbreaks and phylogenetically clustering with NHSGGC cases are also highlighted; clades 1, 3, and 4 were each associated with 2, 3 and 1 other universities respectively. The scale represents the number of substitutions per site. Phylogenetic trees were created in The Environment for Tree Exploration ^43^. Numbers of sequences informing this analysis are as follows: Clade 1 n=217, Clade 2 n=48, Clade 3 n=86, Clade 4 n=90.

**Table S1**. Proportion of students domiciled within each United Kingdom nation, for higher education providers grouped by nation. Numbers derived from Higher Education Statistics Agency 2019/20 estimates ^29^.

| Nation of HE provider | Nation domicile | Proportion of students |
| --- | --- | --- |
| England | England | 0.966 |
| England | Northern Ireland | 0.008 |
| England | Scotland | 0.006 |
| England | Wales | 0.020 |
| Northern Ireland | England | 0.063 |
| Northern Ireland | Northern Ireland | 0.930 |
| Northern Ireland | Scotland | 0.005 |
| Northern Ireland | Wales | 0.003 |
| Scotland | England | 0.136 |
| Scotland | Northern Ireland | 0.020 |
| Scotland | Scotland | 0.840 |
| Scotland | Wales | 0.004 |
| Wales | England | 0.372 |
| Wales | Northern Ireland | 0.005 |
| Wales | Scotland | 0.003 |
| Wales | Wales | 0.620 |

**Table S2.** Proportion of University of Glasgow students domiciled in each region of the United Kingdom. Numbers derived from Higher Education Statistics Agency 2019/20 estimates ^29^.

| UK Region of UoG  student domicile | Proportion of students |
| --- | --- |
| North East | 0.017 |
| North West | 0.030 |
| Yorkshire and The Humber | 0.016 |
| East Midlands | 0.007 |
| West Midlands | 0.008 |
| East of England | 0.014 |
| London | 0.026 |
| South East | 0.022 |
| South West | 0.011 |
| Wales | 0.004 |
| Scotland | 0.814 |
| Northern Ireland | 0.030 |
