# Supplementary material for: Genomic epidemiology of SARS-CoV-2 in a university outbreak setting and implications for public health planning
Source: Sci Rep. 2022 Jul 19;12:11735. doi: 10.1038/s41598-022-15661-1 (PMC9296497; doi:10.1038/s41598-022-15661-1)
Supplement: Supplementary file 2 — Supplementary Information 2. [file 41598_2022_15661_MOESM2_ESM.docx]

Genomic epidemiology of SARS-CoV-2 in a university outbreak setting and implications for public health planning

Genome Sequence Identifiers

Tables 1-4 provide the SARS-CoV-2 genome sequence identifiers relating to each phylogenetic clade inferred by the study, together with the specimen collection date and an identifier for the University of Glasgow student cases.

**Table 1: Phylogenetic clade 1**

| **COG-ID** | **Specimen collection date** | **Student case indicator*** |
| --- | --- | --- |
| England/MILK-99CD45/2020 | 2020-08-30 | NA |
| England/MILK-99DDAE/2020 | 2020-08-31 | NA |
| England/MILK-9AB095/2020 | 2020-08-31 | NA |
| England/MILK-9A509B/2020 | 2020-09-02 | NA |
| Scotland/QEUH-9AF19E/2020 | 2020-09-03 | NA |
| Scotland/QEUH-9AD29D/2020 | 2020-09-05 | NA |
| England/NOTT-113027/2020 | 2020-09-07 | NA |
| Scotland/QEUH-9BB36C/2020 | 2020-09-09 | NA |
| England/MILK-9C4351/2020 | 2020-09-09 | NA |
| England/CAMC-9C014C/2020 | 2020-09-09 | NA |
| England/MILK-9C2292/2020 | 2020-09-11 | NA |
| England/QEUH-9CA43A/2020 | 2020-09-13 | NA |
| Belgium/rega-0915953/2020 | 2020-09-15 | NA |
| England/ALDP-9CAA1A/2020 | 2020-09-16 | NA |
| Belgium/rega-0916979/2020 | 2020-09-16 | NA |
| Belgium/rega-0916963/2020 | 2020-09-16 | NA |
| Scotland/QEUH-9D11B1/2020 | 2020-09-16 | NA |
| England/ALDP-9CDCB7/2020 | 2020-09-16 | NA |
| England/ALDP-9CB554/2020 | 2020-09-16 | NA |
| Scotland/QEUH-9D07A1/2020 | 2020-09-16 | NA |
| Scotland/QEUH-9D0CA2/2020 | 2020-09-16 | NA |
| Scotland/QEUH-9CE7D3/2020 | 2020-09-17 | NA |
| Scotland/QEUH-9CF620/2020 | 2020-09-17 | NA |
| Scotland/QEUH-9CE982/2020 | 2020-09-17 | H5 |
| England/MILK-9D3B39/2020 | 2020-09-18 | NA |
| England/MILK-9E029E/2020 | 2020-09-19 | NA |
| England/MILK-9D5496/2020 | 2020-09-19 | NA |
| Scotland/CVR4438/2020 | 2020-09-19 | NA |
| Scotland/CVR4437/2020 | 2020-09-19 | Private |
| Scotland/QEUH-9D2578/2020 | 2020-09-19 | H8 |
| Scotland/QEUH-9D252D/2020 | 2020-09-19 | NA |
| Scotland/CVR4436/2020 | 2020-09-19 | H5 |
| Scotland/CVR4443/2020 | 2020-09-19 | H7 |
| Scotland/CVR4444/2020 | 2020-09-19 | H7 |
| Scotland/QEUH-9D254B/2020 | 2020-09-19 | H8 |
| England/MILK-9E1831/2020 | 2020-09-20 | NA |
| England/MILK-9E22BA/2020 | 2020-09-20 | NA |
| England/MILK-9E05C2/2020 | 2020-09-20 | NA |
| England/MILK-9E2092/2020 | 2020-09-20 | NA |
| England/MILK-9E0683/2020 | 2020-09-20 | NA |
| Scotland/QEUH-9DE907/2020 | 2020-09-20 | H6 |
| Scotland/CVR4454/2020 | 2020-09-20 | H5 |
| Scotland/CVR4467/2020 | 2020-09-20 | H7 |
| Scotland/CVR4480/2020 | 2020-09-20 | H8 |
| Scotland/CVR4484/2020 | 2020-09-20 | H7 |
| Scotland/CVR4487/2020 | 2020-09-20 | H7 |
| Scotland/CVR4486/2020 | 2020-09-20 | H7 |
| Scotland/CVR4450/2020 | 2020-09-20 | H7 |
| Scotland/CVR4461/2020 | 2020-09-20 | H6 |
| Scotland/CVR4455/2020 | 2020-09-20 | H5 |
| Scotland/CVR4449/2020 | 2020-09-20 | Private |
| Scotland/CVR4479/2020 | 2020-09-20 | H5 |
| Scotland/CVR4504/2020 | 2020-09-20 | H7 |
| Scotland/CVR4482/2020 | 2020-09-20 | H7 |
| Scotland/CVR4466/2020 | 2020-09-20 | H7 |
| Scotland/CVR4468/2020 | 2020-09-20 | H7 |
| Scotland/CVR4469/2020 | 2020-09-20 | H7 |
| Scotland/CVR4459/2020 | 2020-09-20 | Private |
| Scotland/CVR4458/2020 | 2020-09-20 | Private |
| Scotland/CVR4485/2020 | 2020-09-20 | H7 |
| Scotland/CVR4465/2020 | 2020-09-20 | H7 |
| Scotland/CVR4471/2020 | 2020-09-20 | H7 |
| Scotland/CVR4463/2020 | 2020-09-20 | H5 |
| Scotland/QEUH-9DEC0E/2020 | 2020-09-21 | NA |
| England/NOTT-1133F1/2020 | 2020-09-21 | NA |
| Scotland/QEUH-9DE679/2020 | 2020-09-21 | H8 |
| Scotland/CVR4496/2020 | 2020-09-21 | H8 |
| Scotland/CVR4511/2020 | 2020-09-21 | H5 |
| Scotland/QEUH-9DE70D/2020 | 2020-09-21 | Private |
| Scotland/QEUH-9DE6F1/2020 | 2020-09-21 | H5 |
| Scotland/CVR4495/2020 | 2020-09-21 | H5 |
| Scotland/CVR4514/2020 | 2020-09-21 | H8 |
| Scotland/QEUH-9DE688/2020 | 2020-09-21 | NA |
| Scotland/QEUH-9DEAE6/2020 | 2020-09-21 | NA |
| Scotland/QEUH-9DED56/2020 | 2020-09-21 | Private |
| Scotland/CVR4502/2020 | 2020-09-21 | H7 |
| Scotland/CVR4501/2020 | 2020-09-21 | H7 |
| Scotland/CVR4492/2020 | 2020-09-21 | H5 |
| Scotland/QEUH-9DEDA1/2020 | 2020-09-21 | H9 |
| Scotland/CVR4510/2020 | 2020-09-21 | H5 |
| Scotland/CVR4503/2020 | 2020-09-21 | H5 |
| Scotland/CVR5105/2020 | 2020-09-21 | NA |
| Scotland/QEUH-9DEB01/2020 | 2020-09-21 | NA |
| Scotland/QEUH-9DEAAA/2020 | 2020-09-21 | NA |
| Scotland/CVR4498/2020 | 2020-09-21 | H7 |
| Scotland/CVR4493/2020 | 2020-09-21 | H10 |
| Scotland/CVR4513/2020 | 2020-09-21 | H5 |
| Scotland/CVR4483/2020 | 2020-09-21 | H8 |
| Scotland/CVR4506/2020 | 2020-09-21 | H5 |
| Scotland/QEUH-9DE71C/2020 | 2020-09-21 | H5 |
| Scotland/QEUH-9DD7B3/2020 | 2020-09-21 | H5 |
| Scotland/QEUH-9DEA40/2020 | 2020-09-21 | H5 |
| Scotland/QEUH-9DE72B/2020 | 2020-09-21 | H5 |
| Scotland/QEUH-9DA859/2020 | 2020-09-22 | NA |
| Scotland/QEUH-9DA9B0/2020 | 2020-09-22 | NA |
| Scotland/QEUH-9DA9CF/2020 | 2020-09-22 | NA |
| England/QEUH-9F330E/2020 | 2020-09-22 | NA |
| Scotland/QEUH-9DD407/2020 | 2020-09-22 | NA |
| Scotland/QEUH-9DAA08/2020 | 2020-09-22 | NA |
| Scotland/QEUH-9DAA9F/2020 | 2020-09-22 | NA |
| Scotland/QEUH-9DD294/2020 | 2020-09-22 | NA |
| Scotland/CVR4533/2020 | 2020-09-22 | H7 |
| Scotland/CVR5403/2020 | 2020-09-22 | NA |
| Scotland/CVR4559/2020 | 2020-09-22 | H8 |
| Scotland/CVR5387/2020 | 2020-09-22 | NA |
| Scotland/CVR4543/2020 | 2020-09-22 | H6 |
| Scotland/CVR4526/2020 | 2020-09-22 | H8 |
| Scotland/CVR4550/2020 | 2020-09-22 | H5 |
| Scotland/CVR4528/2020 | 2020-09-22 | H5 |
| Scotland/CVR4540/2020 | 2020-09-22 | H6 |
| Scotland/CVR5414/2020 | 2020-09-22 | NA |
| Scotland/QEUH-9DD1B5/2020 | 2020-09-22 | H5 |
| Scotland/CVR4532/2020 | 2020-09-22 | Private |
| Scotland/CVR4547/2020 | 2020-09-22 | H5 |
| Scotland/CVR4548/2020 | 2020-09-22 | H6 |
| Scotland/CVR4555/2020 | 2020-09-22 | H6 |
| Scotland/QEUH-9DCEBF/2020 | 2020-09-22 | H5 |
| Scotland/QEUH-9DAE93/2020 | 2020-09-22 | NA |
| Scotland/CVR4524/2020 | 2020-09-22 | H5 |
| Scotland/CVR4539/2020 | 2020-09-22 | H5 |
| Scotland/CVR4542/2020 | 2020-09-22 | H5 |
| Scotland/CVR4535/2020 | 2020-09-22 | H5 |
| Scotland/CVR4523/2020 | 2020-09-22 | H5 |
| Scotland/QEUH-9DBE92/2020 | 2020-09-22 | NA |
| Scotland/CVR4553/2020 | 2020-09-22 | H5 |
| Scotland/CVR4560/2020 | 2020-09-22 | H5 |
| Scotland/QEUH-9DD3FB/2020 | 2020-09-22 | NA |
| Scotland/QEUH-9DD3BF/2020 | 2020-09-22 | NA |
| Scotland/QEUH-9DBF53/2020 | 2020-09-22 | H7 |
| Scotland/CVR4562/2020 | 2020-09-22 | H8 |
| Scotland/CVR4536/2020 | 2020-09-22 | H8 |
| Scotland/CVR4556/2020 | 2020-09-22 | H7 |
| Scotland/QEUH-9DBF44/2020 | 2020-09-22 | H7 |
| Scotland/CVR5402/2020 | 2020-09-22 | NA |
| Scotland/CVR4551/2020 | 2020-09-22 | H5 |
| Scotland/QEUH-9DC9DC/2020 | 2020-09-22 | NA |
| Scotland/QEUH-9DCEA0/2020 | 2020-09-22 | NA |
| Scotland/QEUH-9DC3ED/2020 | 2020-09-22 | NA |
| England/QEUH-9DCE82/2020 | 2020-09-22 | NA |
| England/MILK-9EE7DD/2020 | 2020-09-23 | NA |
| England/CAMC-9EBCA4/2020 | 2020-09-23 | NA |
| England/CAMC-9EC881/2020 | 2020-09-23 | NA |
| England/CAMC-9ECB1F/2020 | 2020-09-23 | NA |
| Scotland/CVR4655/2020 | 2020-09-23 | NA |
| Scotland/QEUH-9E531E/2020 | 2020-09-23 | NA |
| Northern_Ireland/NIRE-233239/2020 | 2020-09-23 | NA |
| Scotland/QEUH-9E484D/2020 | 2020-09-23 | H7 |
| Scotland/QEUH-9E4898/2020 | 2020-09-23 | NA |
| Scotland/QEUH-9E4573/2020 | 2020-09-23 | H7 |
| Scotland/QEUH-9E41F7/2020 | 2020-09-23 | H9 |
| Scotland/QEUH-9E4203/2020 | 2020-09-23 | H5 |
| Scotland/QEUH-9E428B/2020 | 2020-09-23 | Private |
| Scotland/QEUH-9E4528/2020 | 2020-09-23 | H7 |
| Scotland/QEUH-9E46E9/2020 | 2020-09-23 | H5 |
| Scotland/QEUH-9E432E/2020 | 2020-09-23 | Private |
| Scotland/QEUH-9E482F/2020 | 2020-09-23 | H8 |
| Scotland/QEUH-9E4CC9/2020 | 2020-09-23 | H7 |
| Scotland/QEUH-9E4A47/2020 | 2020-09-23 | Private |
| Scotland/QEUH-9E48B6/2020 | 2020-09-23 | NA |
| Scotland/QEUH-9E4494/2020 | 2020-09-23 | Private |
| Scotland/QEUH-9E48A7/2020 | 2020-09-23 | H9 |
| Scotland/QEUH-9E429A/2020 | 2020-09-23 | NA |
| Scotland/QEUH-9E42F4/2020 | 2020-09-23 | NA |
| Scotland/QEUH-9E42A9/2020 | 2020-09-23 | NA |
| Scotland/QEUH-9E4564/2020 | 2020-09-23 | H7 |
| Scotland/QEUH-9E4537/2020 | 2020-09-23 | H7 |
| Scotland/QEUH-9E41CA/2020 | 2020-09-23 | NA |
| Scotland/QEUH-9E4A56/2020 | 2020-09-23 | Private |
| Scotland/QEUH-9E417F/2020 | 2020-09-23 | NA |
| Scotland/QEUH-9E483E/2020 | 2020-09-23 | H7 |
| England/ALDP-9E7C11/2020 | 2020-09-24 | NA |
| England/MILK-9F224E/2020 | 2020-09-24 | NA |
| England/MILK-9F1D8A/2020 | 2020-09-24 | NA |
| England/MILK-9F1D7B/2020 | 2020-09-24 | NA |
| England/MILK-9F1D20/2020 | 2020-09-24 | NA |
| Scotland/QEUH-9E54DF/2020 | 2020-09-24 | NA |
| Northern_Ireland/NIRE-23321B/2020 | 2020-09-24 | NA |
| Scotland/QEUH-9F30BC/2020 | 2020-09-24 | H5 |
| Scotland/QEUH-9E38B7/2020 | 2020-09-24 | H4 |
| Scotland/QEUH-9F3140/2020 | 2020-09-24 | H5 |
| Scotland/QEUH-9E4E96/2020 | 2020-09-24 | Private |
| Scotland/QEUH-9E3644/2020 | 2020-09-24 | Private |
| Scotland/QEUH-9F309E/2020 | 2020-09-24 | H5 |
| Scotland/QEUH-9F30CB/2020 | 2020-09-24 | H5 |
| England/QEUH-9F60AA/2020 | 2020-09-25 | NA |
| Scotland/CVR4647/2020 | 2020-09-26 | Private |
| Scotland/QEUH-9F565E/2020 | 2020-09-27 | NA |
| England/QEUH-9F9CC1/2020 | 2020-09-27 | NA |
| Scotland/QEUH-9F6AD9/2020 | 2020-09-27 | NA |
| Scotland/QEUH-9F6ABB/2020 | 2020-09-27 | NA |
| Scotland/QEUH-9F67E1/2020 | 2020-09-27 | NA |
| Scotland/QEUH-9F6981/2020 | 2020-09-27 | H6 |
| Scotland/QEUH-9F854F/2020 | 2020-09-27 | NA |
| Scotland/QEUH-9F4F27/2020 | 2020-09-27 | Private |
| Scotland/QEUH-9F61E3/2020 | 2020-09-27 | NA |
| Scotland/QEUH-9F6A8E/2020 | 2020-09-27 | Private |
| Scotland/QEUH-9F77B3/2020 | 2020-09-27 | NA |
| Scotland/QEUH-9F77C2/2020 | 2020-09-27 | NA |
| Scotland/QEUH-9F65F6/2020 | 2020-09-27 | NA |
| Scotland/QEUH-9F46AA/2020 | 2020-09-27 | NA |
| Scotland/QEUH-9F76F2/2020 | 2020-09-27 | NA |
| Scotland/QEUH-9F4F54/2020 | 2020-09-27 | NA |
| Scotland/QEUH-9F65E7/2020 | 2020-09-27 | NA |
| Scotland/QEUH-9F6602/2020 | 2020-09-27 | NA |
| Scotland/QEUH-9F5AAD/2020 | 2020-09-27 | NA |
| Scotland/QEUH-9F5F71/2020 | 2020-09-27 | NA |
| Scotland/QEUH-9F620E/2020 | 2020-09-27 | NA |
| Scotland/QEUH-9F61D4/2020 | 2020-09-27 | NA |
| Scotland/QEUH-9F614D/2020 | 2020-09-27 | NA |
| Scotland/QEUH-9F6198/2020 | 2020-09-27 | NA |
| Scotland/QEUH-9F615C/2020 | 2020-09-27 | Private |
| Scotland/QEUH-9F6189/2020 | 2020-09-27 | NA |
| Scotland/QEUH-9F616B/2020 | 2020-09-27 | NA |
| Scotland/QEUH-9F5FAE/2020 | 2020-09-27 | NA |
| Scotland/QEUH-9F69AF/2020 | 2020-09-27 | NA |
| Scotland/QEUH-9F59EC/2020 | 2020-09-27 | NA |
| Scotland/QEUH-9F4604/2020 | 2020-09-27 | H5 |
| Scotland/QEUH-9F4631/2020 | 2020-09-27 | NA |
| Scotland/QEUH-9F6A33/2020 | 2020-09-27 | NA |
| Wales/PHWC-16DEF7/2020 | 2020-09-28 | NA |
| England/QEUH-A025AB/2020 | 2020-09-29 | NA |
| Scotland/QEUH-9FB49A/2020 | 2020-09-29 | NA |
| Scotland/QEUH-9FB62B/2020 | 2020-09-29 | NA |
| Scotland/QEUH-9FB44F/2020 | 2020-09-29 | Private |
| Scotland/QEUH-9FB245/2020 | 2020-09-29 | NA |
| Scotland/QEUH-A02927/2020 | 2020-09-29 | NA |
| Scotland/QEUH-9FB3AC/2020 | 2020-09-29 | NA |
| Scotland/QEUH-9FB5C4/2020 | 2020-09-29 | NA |
| Scotland/QEUH-9FB351/2020 | 2020-09-29 | NA |
| Scotland/QEUH-9FB5E2/2020 | 2020-09-29 | NA |
| Scotland/QEUH-9FB5A6/2020 | 2020-09-29 | NA |
| Scotland/QEUH-9FB5F1/2020 | 2020-09-29 | NA |
| Scotland/QEUH-9FB61C/2020 | 2020-09-29 | NA |
| Scotland/QEUH-9FB597/2020 | 2020-09-29 | NA |
| Scotland/QEUH-9FB60D/2020 | 2020-09-29 | NA |
| Scotland/QEUH-9FB254/2020 | 2020-09-29 | NA |
| Scotland/QEUH-9FB46D/2020 | 2020-09-29 | NA |
| Scotland/QEUH-9FB1FD/2020 | 2020-09-29 | Private |
| Scotland/QEUH-9FB218/2020 | 2020-09-29 | NA |
| England/MILK-A19143/2020 | 2020-09-30 | NA |
| England/QEUH-A0B01A/2020 | 2020-09-30 | NA |
| England/MILK-A054D8/2020 | 2020-09-30 | NA |
| England/MILK-A0603D/2020 | 2020-09-30 | NA |
| Scotland/EDB8327/2020 | 2020-09-30 | NA |
| Scotland/QEUH-A02A15/2020 | 2020-09-30 | NA |
| Scotland/EDB8326/2020 | 2020-09-30 | NA |
| England/QEUH-A0BB55/2020 | 2020-10-01 | NA |
| Scotland/QEUH-A1FE55/2020 | 2020-10-01 | NA |
| England/MILK-A1843C/2020 | 2020-10-01 | NA |
| Scotland/QEUH-A1F866/2020 | 2020-10-01 | NA |
| Scotland/QEUH-A1FFBC/2020 | 2020-10-01 | NA |
| Scotland/QEUH-A1F64E/2020 | 2020-10-01 | NA |
| Scotland/QEUH-A1F5E7/2020 | 2020-10-01 | NA |
| Scotland/QEUH-A1FF16/2020 | 2020-10-01 | NA |
| Scotland/QEUH-A1F8C0/2020 | 2020-10-01 | NA |
| Scotland/QEUH-A1FDEF/2020 | 2020-10-01 | NA |
| Scotland/QEUH-A1FE37/2020 | 2020-10-01 | NA |
| Scotland/QEUH-A226FD/2020 | 2020-10-01 | NA |
| Scotland/QEUH-A226B1/2020 | 2020-10-01 | NA |
| Scotland/QEUH-A226C0/2020 | 2020-10-01 | NA |
| Scotland/QEUH-A1FF8F/2020 | 2020-10-01 | NA |
| Scotland/QEUH-A1F611/2020 | 2020-10-01 | NA |
| Scotland/QEUH-A1FABB/2020 | 2020-10-01 | NA |
| Scotland/QEUH-A20DAF/2020 | 2020-10-01 | NA |
| Scotland/QEUH-A1F58D/2020 | 2020-10-01 | NA |
| Scotland/QEUH-A1EE38/2020 | 2020-10-01 | NA |
| Scotland/QEUH-A1FA9D/2020 | 2020-10-01 | Private |
| Scotland/QEUH-A22727/2020 | 2020-10-01 | NA |
| Scotland/QEUH-A1F57E/2020 | 2020-10-01 | NA |
| Scotland/QEUH-A221B0/2020 | 2020-10-01 | NA |
| Scotland/QEUH-A1FD67/2020 | 2020-10-01 | NA |
| Scotland/QEUH-A1FE64/2020 | 2020-10-01 | NA |
| Scotland/QEUH-A1F5C9/2020 | 2020-10-01 | NA |
| Scotland/QEUH-A1E436/2020 | 2020-10-01 | NA |
| Scotland/QEUH-A1F5BA/2020 | 2020-10-01 | NA |
| Scotland/QEUH-A1F8DF/2020 | 2020-10-01 | NA |
| Scotland/QEUH-A1F70F/2020 | 2020-10-01 | NA |
| Scotland/QEUH-A2195F/2020 | 2020-10-01 | NA |
| Scotland/QEUH-A1F82A/2020 | 2020-10-01 | NA |
| Scotland/QEUH-A1F875/2020 | 2020-10-01 | NA |
| Scotland/QEUH-A1F927/2020 | 2020-10-01 | NA |
| Scotland/QEUH-9FFE3E/2020 | 2020-10-02 | NA |
| Scotland/QEUH-A007F2/2020 | 2020-10-02 | NA |
| Scotland/QEUH-A00B05/2020 | 2020-10-02 | NA |
| Scotland/QEUH-A013DF/2020 | 2020-10-02 | NA |
| Scotland/QEUH-A00A53/2020 | 2020-10-02 | NA |
| Scotland/QEUH-A00A08/2020 | 2020-10-02 | NA |
| Scotland/QEUH-A00A80/2020 | 2020-10-02 | NA |
| Scotland/QEUH-A00A26/2020 | 2020-10-02 | NA |
| Scotland/QEUH-9FF672/2020 | 2020-10-02 | NA |
| England/CAMC-A3EA01/2020 | 2020-10-03 | NA |
| England/MILK-A1D30D/2020 | 2020-10-03 | NA |
| England/MILK-A1D534/2020 | 2020-10-03 | NA |
| England/MILK-A1D4A0/2020 | 2020-10-03 | NA |
| England/MILK-A1D31C/2020 | 2020-10-03 | NA |
| Scotland/QEUH-A0BD22/2020 | 2020-10-03 | NA |
| Scotland/QEUH-A0B7D9/2020 | 2020-10-03 | NA |
| Scotland/QEUH-A0B8D6/2020 | 2020-10-03 | NA |
| Scotland/QEUH-A0B86D/2020 | 2020-10-03 | NA |
| Scotland/QEUH-A0BC52/2020 | 2020-10-03 | NA |
| Scotland/QEUH-A0BC34/2020 | 2020-10-03 | NA |
| Scotland/QEUH-A0B85E/2020 | 2020-10-03 | NA |
| Scotland/QEUH-A0BD40/2020 | 2020-10-03 | NA |
| Scotland/QEUH-A0BD7D/2020 | 2020-10-03 | Private |
| Scotland/QEUH-A0BC61/2020 | 2020-10-03 | NA |
| England/QEUH-A37060/2020 | 2020-10-04 | NA |
| Northern_Ireland/NIRE-23399E/2020 | 2020-10-04 | NA |
| Scotland/QEUH-A33583/2020 | 2020-10-04 | NA |
| Scotland/CVR4889/2020 | 2020-10-05 | NA |
| Scotland/QEUH-A4C305/2020 | 2020-10-05 | NA |
| Scotland/EDB8325/2020 | 2020-10-05 | NA |
| Scotland/CVR4954/2020 | 2020-10-05 | NA |
| Ireland/LH-NVRL-75IRL19164/2020 | 2020-10-06 | NA |
| England/MILK-A456D6/2020 | 2020-10-06 | NA |
| England/MILK-A4805D/2020 | 2020-10-06 | NA |
| England/EXET-138CFB/2020 | 2020-10-06 | NA |
| England/EXET-1381FC/2020 | 2020-10-06 | NA |
| Scotland/QEUH-A49B6A/2020 | 2020-10-06 | NA |
| Scotland/CVR4936/2020 | 2020-10-06 | NA |
| England/ALDP-A51D5A/2020 | 2020-10-07 | NA |
| Scotland/QEUH-A4B4B8/2020 | 2020-10-08 | NA |
| Northern_Ireland/NIRE-108B15/2020 | 2020-10-08 | NA |
| Scotland/QEUH-A4B676/2020 | 2020-10-08 | NA |
| Scotland/QEUH-A4AA8A/2020 | 2020-10-08 | NA |
| Scotland/CVR5026/2020 | 2020-10-08 | NA |
| Scotland/QEUH-A4B63A/2020 | 2020-10-08 | NA |
| Scotland/QEUH-A4B5A6/2020 | 2020-10-08 | NA |
| Scotland/QEUH-A48E17/2020 | 2020-10-08 | NA |
| Scotland/QEUH-A4B2CD/2020 | 2020-10-08 | NA |
| Scotland/QEUH-A4AAC6/2020 | 2020-10-08 | NA |
| Scotland/QEUH-A48E80/2020 | 2020-10-08 | NA |
| Scotland/QEUH-A639F7/2020 | 2020-10-09 | NA |
| Scotland/CVR5045/2020 | 2020-10-09 | NA |
| Scotland/QEUH-A63906/2020 | 2020-10-09 | NA |
| England/CAMC-A64E60/2020 | 2020-10-11 | NA |
| England/CAMC-A66129/2020 | 2020-10-11 | NA |
| England/CAMC-A65B77/2020 | 2020-10-11 | NA |
| Netherlands/UT-EMC-115/2020 | 2020-10-12 | NA |
| England/ALDP-A68B1A/2020 | 2020-10-12 | NA |
| England/QEUH-A60383/2020 | 2020-10-12 | NA |
| England/MILK-A7CEFC/2020 | 2020-10-12 | NA |
| Scotland/CAMC-A8236D/2020 | 2020-10-12 | NA |
| England/NORW-F17C1/2020 | 2020-10-12 | NA |
| USA/NY-NYCPHL-001064/2020 | 2020-10-13 | NA |
| Ireland/D-NVRL-75IRL86056/2020 | 2020-10-14 | NA |
| Northern_Ireland/NIRE-234669/2020 | 2020-10-14 | NA |
| England/ALDP-A6F9EC/2020 | 2020-10-14 | NA |
| Scotland/QEUH-A7934B/2020 | 2020-10-14 | NA |
| England/ALDP-A6C721/2020 | 2020-10-14 | NA |
| England/MILK-A91055/2020 | 2020-10-16 | NA |
| Scotland/QEUH-A8B267/2020 | 2020-10-16 | NA |
| England/NORW-F01A6/2020 | 2020-10-16 | NA |
| England/ALDP-A87ACD/2020 | 2020-10-17 | NA |
| England/PHEC-14A29A/2020 | 2020-10-19 | NA |
| Northern_Ireland/NIRE-10909D/2020 | 2020-10-20 | NA |
| Scotland/QEUH-A9E313/2020 | 2020-10-20 | NA |
| Scotland/QEUH-A9E2E9/2020 | 2020-10-20 | H6 |
| Scotland/QEUH-A9E216/2020 | 2020-10-20 | NA |
| Scotland/QEUH-A9E3F5/2020 | 2020-10-20 | NA |
| England/MILK-ACEFAD/2020 | 2020-10-21 | NA |
| Luxembourg/LNS0917978/2020 | 2020-10-21 | NA |
| England/MILK-AC933D/2020 | 2020-10-21 | NA |
| Scotland/QEUH-AACB0B/2020 | 2020-10-21 | NA |
| England/MILK-ACC7A7/2020 | 2020-10-21 | NA |
| England/MILK-ABBD6D/2020 | 2020-10-21 | NA |
| England/MILK-ACC07F/2020 | 2020-10-21 | NA |
| Luxembourg/LNS5605056/2020 | 2020-10-22 | NA |
| England/CAMC-AB02E1/2020 | 2020-10-22 | NA |
| England/MILK-AB936D/2020 | 2020-10-22 | NA |
| England/ALDP-AA1BBB/2020 | 2020-10-22 | NA |
| Luxembourg/LNS6216319/2020 | 2020-10-24 | NA |
| Scotland/QEUH-AD4AEF/2020 | 2020-10-27 | NA |
| Netherlands/FL-EMC-42/2020 | 2020-10-29 | NA |
| England/CAMC-B07AD3/2020 | 2020-10-29 | NA |
| England/CAMC-B07DDA/2020 | 2020-10-29 | NA |
| Luxembourg/LNS1149202/2020 | 2020-11-01 | NA |
| England/MILK-B3C99C/2020 | 2020-11-01 | NA |
| England/CAMC-B210C2/2020 | 2020-11-01 | NA |
| England/CAMC-B2289C/2020 | 2020-11-01 | NA |
| Luxembourg/LNS6456581/2020 | 2020-11-02 | NA |
| England/CAMC-B202CD/2020 | 2020-11-02 | NA |
| England/CAMC-B2279F/2020 | 2020-11-02 | NA |
| England/QEUH-B11C58/2020 | 2020-11-02 | NA |
| England/LOND-12F03E7/2020 | 2020-11-03 | NA |
| Ireland/MH-NVRL-77IRL69686/2020 | 2020-11-05 | NA |
| England/MILK-B5557B/2020 | 2020-11-05 | NA |
| England/CAMC-B33625/2020 | 2020-11-05 | NA |
| Portugal/PT1650/2020 | 2020-11-07 | NA |
| England/CAMC-B54187/2020 | 2020-11-08 | NA |
| England/CAMC-B51278/2020 | 2020-11-08 | NA |
| Portugal/PT1640/2020 | 2020-11-09 | NA |
| England/ALDP-B5BDE6/2020 | 2020-11-09 | NA |
| England/CAMC-B778A7/2020 | 2020-11-11 | NA |
| England/QEUH-B6BA46/2020 | 2020-11-11 | NA |
| England/CAMC-B77EF0/2020 | 2020-11-12 | NA |
| England/CAMC-B7B393/2020 | 2020-11-12 | NA |
| England/CAMC-B7AF63/2020 | 2020-11-12 | NA |
| Portugal/PT1919/2020 | 2020-11-13 | NA |
| England/MILK-B9730A/2020 | 2020-11-13 | NA |
| Portugal/PT1756/2020 | 2020-11-13 | NA |
| Portugal/PT1909/2020 | 2020-11-13 | NA |
| Portugal/PT1912/2020 | 2020-11-13 | NA |
| England/MILK-B8E3F7/2020 | 2020-11-13 | NA |
| England/QEUH-B7CC1F/2020 | 2020-11-14 | NA |
| England/MILK-BB00B9/2020 | 2020-11-17 | NA |
| England/NORW-F7B47/2020 | 2020-11-18 | NA |
| England/MILK-BAFFFC/2020 | 2020-11-18 | NA |
| England/ALDP-BB31D1/2020 | 2020-11-19 | NA |
| England/ALDP-BC62C9/2020 | 2020-11-23 | NA |
| England/ALDP-BC62E7/2020 | 2020-11-23 | NA |
| England/QEUH-BC3EE6/2020 | 2020-11-24 | NA |
| England/QEUH-BC489C/2020 | 2020-11-25 | NA |

*University of Glasgow student cases are indicated with ‘H’ and ‘Private’ denoting students residing in university halls and private accommodation respectively. NA indicates cases deemed to be non-students.

**Table 2: Phylogenetic clade 2**

| **COG-ID** | **Specimen collection date** | **Student case indicator*** |
| --- | --- | --- |
| England/QEUH-9BAF69/2020 | 2020-09-07 | NA |
| England/MILK-9C2AB8/2020 | 2020-09-10 | NA |
| England/MILK-9C2678/2020 | 2020-09-10 | NA |
| Scotland/MILK-9C263C/2020 | 2020-09-10 | NA |
| England/MILK-9C2696/2020 | 2020-09-10 | NA |
| England/MILK-9C2A8B/2020 | 2020-09-10 | NA |
| England/MILK-9C262D/2020 | 2020-09-10 | NA |
| England/MILK-9C2687/2020 | 2020-09-10 | NA |
| England/MILK-9C2669/2020 | 2020-09-10 | NA |
| England/MILK-9C264B/2020 | 2020-09-10 | NA |
| England/QEUH-9D0589/2020 | 2020-09-14 | NA |
| England/MILK-9D4039/2020 | 2020-09-18 | NA |
| England/MILK-9D4048/2020 | 2020-09-18 | NA |
| England/MILK-9E0DE8/2020 | 2020-09-19 | NA |
| England/CAMC-9D1E7E/2020 | 2020-09-19 | NA |
| England/MILK-9E028F/2020 | 2020-09-19 | NA |
| England/MILK-9D4B38/2020 | 2020-09-19 | NA |
| England/CAMC-9D6EB5/2020 | 2020-09-20 | NA |
| England/CAMC-9D6EE2/2020 | 2020-09-20 | NA |
| England/MILK-9E1DAB/2020 | 2020-09-20 | NA |
| England/CAMC-9DF274/2020 | 2020-09-20 | NA |
| Scotland/QEUH-9DD7C2/2020 | 2020-09-21 | H1 |
| Scotland/CVR4837/2020 | 2020-09-21 | H1 |
| England/MILK-9E2F77/2020 | 2020-09-21 | NA |
| Scotland/QEUH-9DEDED/2020 | 2020-09-21 | H1 |
| England/QEUH-9DE925/2020 | 2020-09-21 | NA |
| Scotland/CVR4838/2020 | 2020-09-21 | H1 |
| Scotland/CVR4841/2020 | 2020-09-21 | H1 |
| Scotland/CVR4836/2020 | 2020-09-21 | H1 |
| Scotland/CVR4835/2020 | 2020-09-21 | H1 |
| Scotland/QEUH-9DD856/2020 | 2020-09-21 | H1 |
| Scotland/QEUH-9DD80B/2020 | 2020-09-21 | H1 |
| Scotland/QEUH-9DD7FF/2020 | 2020-09-21 | H1 |
| Scotland/CVR4518/2020 | 2020-09-21 | H7 |
| England/QEUH-9DC31A/2020 | 2020-09-21 | NA |
| Scotland/QEUH-9DEE17/2020 | 2020-09-21 | H1 |
| Wales/CVR4829/2020 | 2020-09-21 | NA |
| Scotland/CVR4545/2020 | 2020-09-22 | H7 |
| Scotland/CVR4544/2020 | 2020-09-22 | H5 |
| Scotland/QEUH-9DAB23/2020 | 2020-09-22 | H2 |
| Scotland/QEUH-9DA7C5/2020 | 2020-09-22 | NA |
| Wales/PHWC-16DB5A/2020 | 2020-09-22 | NA |
| England/MILK-9EF736/2020 | 2020-09-23 | NA |
| Scotland/QEUH-9E42E5/2020 | 2020-09-23 | H1 |
| Scotland/QEUH-9E4D02/2020 | 2020-09-23 | Private |
| Scotland/QEUH-9E4300/2020 | 2020-09-23 | H3 |
| Scotland/QEUH-9E4CE7/2020 | 2020-09-23 | NA |
| Scotland/QEUH-9E46BC/2020 | 2020-09-23 | NA |
| England/MILK-9EF824/2020 | 2020-09-23 | NA |
| England/MILK-9EF806/2020 | 2020-09-23 | NA |
| England/MILK-9EEEE7/2020 | 2020-09-24 | NA |
| England/MILK-9F0AFD/2020 | 2020-09-24 | NA |
| Scotland/QEUH-9E5150/2020 | 2020-09-24 | NA |
| England/MILK-9F0AEE/2020 | 2020-09-24 | NA |
| England/MILK-9F084E/2020 | 2020-09-24 | NA |
| England/MILK-9F041D/2020 | 2020-09-24 | NA |
| England/QEUH-9F6AE8/2020 | 2020-09-26 | NA |
| England/QEUH-9F9CB2/2020 | 2020-09-27 | NA |
| Scotland/QEUH-9F658D/2020 | 2020-09-27 | Private |
| Scotland/QEUH-9F65AB/2020 | 2020-09-27 | Private |
| Scotland/QEUH-9F7179/2020 | 2020-09-27 | NA |
| Scotland/QEUH-9F6523/2020 | 2020-09-27 | Private |
| Scotland/QEUH-9F5F62/2020 | 2020-09-27 | Private |
| England/QEUH-9F9EF8/2020 | 2020-09-27 | NA |
| England/QEUH-9F9CA3/2020 | 2020-09-27 | NA |
| Wales/PHWC-16E6D0/2020 | 2020-09-28 | NA |
| England/MILK-A05915/2020 | 2020-09-29 | NA |
| Scotland/QEUH-9FB37F/2020 | 2020-09-29 | NA |
| Scotland/CVR4685/2020 | 2020-09-29 | NA |
| England/MILK-A05F6E/2020 | 2020-09-30 | NA |
| England/MILK-A05B88/2020 | 2020-09-30 | NA |
| England/QEUH-9FF9B5/2020 | 2020-09-30 | NA |
| England/QEUH-A0BEA7/2020 | 2020-09-30 | NA |
| England/MILK-A19152/2020 | 2020-09-30 | NA |
| Scotland/QEUH-A1FBA9/2020 | 2020-10-01 | NA |
| Scotland/QEUH-A1DA17/2020 | 2020-10-01 | NA |
| Scotland/QEUH-A1F59C/2020 | 2020-10-01 | Private |
| Scotland/QEUH-A1F857/2020 | 2020-10-01 | Private |
| Scotland/CVR4915/2020 | 2020-10-01 | NA |
| Wales/QEUH-A0B627/2020 | 2020-10-01 | NA |
| Scotland/QEUH-A00C02/2020 | 2020-10-02 | NA |
| Scotland/QEUH-A003D0/2020 | 2020-10-02 | NA |
| England/MILK-A23BDF/2020 | 2020-10-03 | NA |
| England/MILK-A229C7/2020 | 2020-10-03 | NA |
| England/MILK-A23BFD/2020 | 2020-10-03 | NA |
| England/MILK-A2296D/2020 | 2020-10-03 | NA |
| Scotland/QEUH-A0C36B/2020 | 2020-10-03 | NA |
| Scotland/QEUH-A0BCAD/2020 | 2020-10-03 | H1 |
| Scotland/QEUH-A0BC8F/2020 | 2020-10-03 | NA |
| Scotland/QEUH-A0B92E/2020 | 2020-10-03 | NA |
| England/MILK-A228F7/2020 | 2020-10-03 | NA |
| England/MILK-A2294F/2020 | 2020-10-03 | NA |
| England/QEUH-A49B79/2020 | 2020-10-03 | NA |
| England/MILK-A22D8F/2020 | 2020-10-03 | NA |
| England/QEUH-A4CFD4/2020 | 2020-10-04 | NA |
| England/QEUH-A4BD08/2020 | 2020-10-05 | NA |
| Scotland/QEUH-A4B861/2020 | 2020-10-05 | NA |
| England/QEUH-A4D726/2020 | 2020-10-05 | NA |
| England/QEUH-A37A62/2020 | 2020-10-05 | NA |
| England/MILK-A46426/2020 | 2020-10-05 | NA |
| Wales/QEUH-A4BC38/2020 | 2020-10-05 | NA |
| England/QEUH-A4CDE9/2020 | 2020-10-05 | NA |
| England/MILK-A47003/2020 | 2020-10-06 | NA |
| England/MILK-A458FE/2020 | 2020-10-06 | NA |
| England/MILK-A46040/2020 | 2020-10-06 | NA |
| England/MILK-A45E65/2020 | 2020-10-06 | NA |
| England/MILK-A46F70/2020 | 2020-10-06 | NA |
| England/MILK-A46D3A/2020 | 2020-10-06 | NA |
| England/MILK-A47030/2020 | 2020-10-06 | NA |
| England/MILK-A4706D/2020 | 2020-10-06 | NA |
| England/MILK-A4705E/2020 | 2020-10-06 | NA |
| England/MILK-A46F61/2020 | 2020-10-06 | NA |
| England/MILK-A46F34/2020 | 2020-10-06 | NA |
| England/MILK-A46FBC/2020 | 2020-10-06 | NA |
| England/MILK-A46F43/2020 | 2020-10-06 | NA |
| Scotland/QEUH-A4CA88/2020 | 2020-10-07 | NA |
| England/CAMC-A58421/2020 | 2020-10-07 | NA |
| England/MILK-A6AB54/2020 | 2020-10-07 | NA |
| Scotland/QEUH-A3777A/2020 | 2020-10-07 | NA |
| England/CAMC-A3FA88/2020 | 2020-10-07 | NA |
| Scotland/QEUH-A4AAD5/2020 | 2020-10-08 | NA |
| Scotland/QEUH-A499F7/2020 | 2020-10-08 | NA |
| Scotland/QEUH-A4B44F/2020 | 2020-10-08 | NA |
| England/CAMC-A829D4/2020 | 2020-10-08 | NA |
| England/BRIS-134B7A/2020 | 2020-10-08 | NA |
| England/BRIS-134AC8/2020 | 2020-10-08 | NA |
| Scotland/QEUH-A638FA/2020 | 2020-10-09 | NA |
| England/MILK-A69A85/2020 | 2020-10-09 | NA |
| England/ALDP-A6937B/2020 | 2020-10-09 | NA |
| England/CAMC-A57598/2020 | 2020-10-09 | NA |
| England/CAMC-A575B6/2020 | 2020-10-09 | NA |
| England/MILK-A6A36B/2020 | 2020-10-09 | NA |
| England/MILK-A6A4C2/2020 | 2020-10-09 | NA |
| England/MILK-A6A50B/2020 | 2020-10-09 | NA |
| England/CAMC-A6497E/2020 | 2020-10-10 | NA |
| England/CAMC-A64A20/2020 | 2020-10-10 | NA |
| England/CAMC-A65A89/2020 | 2020-10-10 | NA |
| England/CAMC-A6562B/2020 | 2020-10-10 | NA |
| England/CAMC-A65685/2020 | 2020-10-10 | NA |
| England/CAMC-A649F6/2020 | 2020-10-10 | NA |
| England/CAMC-A65667/2020 | 2020-10-10 | NA |
| England/CAMC-A662DB/2020 | 2020-10-11 | NA |
| England/CAMC-A65BA4/2020 | 2020-10-11 | NA |
| England/CAMC-A65B59/2020 | 2020-10-11 | NA |
| England/CAMC-A65FD5/2020 | 2020-10-11 | NA |
| England/CAMC-A64941/2020 | 2020-10-11 | NA |
| England/CAMC-A6556A/2020 | 2020-10-11 | NA |
| England/CAMC-A64FD6/2020 | 2020-10-11 | NA |
| England/CAMC-A65A2F/2020 | 2020-10-11 | NA |
| Wales/QEUH-A63AA9/2020 | 2020-10-12 | NA |
| England/MILK-A795FA/2020 | 2020-10-13 | NA |
| England/MILK-A7D3ED/2020 | 2020-10-13 | NA |
| Wales/CAMC-A80D43/2020 | 2020-10-13 | NA |
| England/MILK-A7DA9D/2020 | 2020-10-13 | NA |
| England/CAMC-A80CFB/2020 | 2020-10-13 | NA |
| England/CAMC-A80CEC/2020 | 2020-10-13 | NA |
| England/CAMC-A820C0/2020 | 2020-10-13 | NA |
| England/ALDP-A6C985/2020 | 2020-10-14 | NA |
| Wales/PHWC-482F50/2020 | 2020-10-14 | NA |
| England/MILK-A7BEDF/2020 | 2020-10-15 | NA |
| England/MILK-A79A37/2020 | 2020-10-15 | NA |
| England/MILK-A79AA0/2020 | 2020-10-15 | NA |
| England/MILK-A7BEEE/2020 | 2020-10-15 | NA |
| England/MILK-A79B61/2020 | 2020-10-15 | NA |
| England/QEUH-A8B971/2020 | 2020-10-16 | NA |
| England/PHEC-148FD1/2020 | 2020-10-16 | NA |
| England/QEUH-A8A1B6/2020 | 2020-10-16 | NA |
| England/QEUH-A89C3E/2020 | 2020-10-16 | NA |
| England/QEUH-A8B908/2020 | 2020-10-16 | NA |
| England/MILK-A913D4/2020 | 2020-10-17 | NA |
| Wales/PHWC-4810A9/2020 | 2020-10-19 | NA |
| England/BRIS-134F23/2020 | 2020-10-19 | NA |
| England/BRIS-1854F23/2020 | 2020-10-19 | NA |
| England/CAMB-1B72E3/2020 | 2020-10-19 | NA |
| England/MILK-AC7B37/2020 | 2020-10-21 | NA |
| England/MILK-ACCA53/2020 | 2020-10-21 | NA |
| England/MILK-ACCA71/2020 | 2020-10-21 | NA |
| England/MILK-ACC9A1/2020 | 2020-10-21 | NA |
| England/MILK-ACE63F/2020 | 2020-10-21 | NA |
| England/MILK-AC77BB/2020 | 2020-10-21 | NA |
| England/MILK-AB82AD/2020 | 2020-10-21 | NA |
| England/MILK-AB82BC/2020 | 2020-10-21 | NA |
| England/MILK-AC844A/2020 | 2020-10-21 | NA |
| England/MILK-AC74A5/2020 | 2020-10-21 | NA |
| England/MILK-AC7821/2020 | 2020-10-21 | NA |
| England/MILK-ACC947/2020 | 2020-10-21 | NA |
| England/MILK-B147CD/2020 | 2020-10-21 | NA |
| England/ALDP-AA223D/2020 | 2020-10-22 | NA |
| England/MILK-ABB2C8/2020 | 2020-10-22 | NA |
| Scotland/CVR5300/2020 | 2020-10-22 | NA |
| England/MILK-AC9018/2020 | 2020-10-22 | NA |
| England/MILK-ACCC5D/2020 | 2020-10-22 | NA |
| England/MILK-AB937C/2020 | 2020-10-22 | NA |
| England/CAMC-AAFA47/2020 | 2020-10-22 | NA |
| Wales/QEUH-AD0764/2020 | 2020-10-23 | NA |
| England/MILK-B1536E/2020 | 2020-10-23 | NA |
| England/MILK-B1535F/2020 | 2020-10-23 | NA |
| England/MILK-B04A03/2020 | 2020-10-24 | NA |
| Scotland/QEUH-AD1AC4/2020 | 2020-10-24 | NA |
| England/MILK-B01CD3/2020 | 2020-10-24 | NA |
| England/MILK-B02C69/2020 | 2020-10-24 | NA |
| England/MILK-B02D57/2020 | 2020-10-24 | NA |
| England/BRIS-18550E3/2020 | 2020-10-26 | NA |
| Scotland/CVR5338/2020 | 2020-10-26 | NA |
| England/CAMC-AEAD74/2020 | 2020-10-26 | NA |
| England/CAMC-AEAE08/2020 | 2020-10-26 | NA |
| England/QEUH-AE20BF/2020 | 2020-10-26 | NA |
| England/BRIS-1855186/2020 | 2020-10-26 | NA |
| England/BRIS-185505C/2020 | 2020-10-26 | NA |
| England/QEUH-AD3E02/2020 | 2020-10-26 | NA |
| England/QEUH-AD28C9/2020 | 2020-10-26 | NA |
| England/BRIS-18551FF/2020 | 2020-10-26 | NA |
| England/CAMC-B08FE2/2020 | 2020-10-29 | NA |
| England/CAMC-B09312/2020 | 2020-10-29 | NA |
| England/QEUH-AF094A/2020 | 2020-10-29 | NA |
| England/MILK-B3D272/2020 | 2020-10-31 | NA |
| England/CAMC-B0788E/2020 | 2020-10-31 | NA |
| England/MILK-B38B03/2020 | 2020-11-01 | NA |
| England/MILK-B38CC4/2020 | 2020-11-01 | NA |
| England/ALDP-B1F8FC/2020 | 2020-11-01 | NA |
| England/CAMC-B1FF06/2020 | 2020-11-01 | NA |
| England/MILK-B3CC1B/2020 | 2020-11-01 | NA |
| England/MILK-B3CCEE/2020 | 2020-11-01 | NA |
| England/CAMB-1B958E/2020 | 2020-11-02 | NA |
| England/CAMC-B3331F/2020 | 2020-11-05 | NA |
| England/CAMC-B34150/2020 | 2020-11-05 | NA |
| England/CAMC-B32C33/2020 | 2020-11-05 | NA |
| England/MILK-B5A7BC/2020 | 2020-11-06 | NA |
| England/BRIS-1855C1C/2020 | 2020-11-06 | NA |
| England/CAMB-1B9955/2020 | 2020-11-07 | NA |
| Scotland/CVR5696/2020 | 2020-11-08 | NA |
| England/ALDP-B5C1CB/2020 | 2020-11-08 | NA |
| England/CAMC-B78ACE/2020 | 2020-11-12 | NA |
| England/MILK-B93C03/2020 | 2020-11-13 | NA |
| England/MILK-B96F43/2020 | 2020-11-13 | NA |
| England/MILK-B8ED26/2020 | 2020-11-13 | NA |
| England/ALDP-B83AB1/2020 | 2020-11-14 | NA |
| England/ALDP-B83B18/2020 | 2020-11-14 | NA |
| England/ALDP-B83C60/2020 | 2020-11-14 | NA |
| England/ALDP-B84977/2020 | 2020-11-14 | NA |
| England/PHEC-14D2E2/2020 | 2020-11-14 | NA |
| England/PHEC-14D30D/2020 | 2020-11-14 | NA |
| England/PHEC-14D3A3/2020 | 2020-11-14 | NA |
| England/PHEC-14D31C/2020 | 2020-11-14 | NA |
| England/PHEC-14D2F1/2020 | 2020-11-14 | NA |
| England/PHEC-14D3B2/2020 | 2020-11-14 | NA |
| England/CAMC-B980C6/2020 | 2020-11-15 | NA |
| England/MILK-BB1CF0/2020 | 2020-11-17 | NA |
| England/QEUH-BA3CD3/2020 | 2020-11-17 | NA |
| England/QEUH-B9ED50/2020 | 2020-11-17 | NA |
| England/MILK-BAC627/2020 | 2020-11-18 | NA |
| England/MILK-BAB9A7/2020 | 2020-11-18 | NA |
| England/MILK-BB01F2/2020 | 2020-11-18 | NA |
| England/MILK-BAB488/2020 | 2020-11-18 | NA |
| England/ALDP-BC65DF/2020 | 2020-11-20 | NA |

*University of Glasgow student cases are indicated with ‘H’ and ‘Private’ denoting students residing in university halls and private accommodation respectively. NA indicates cases deemed to be non-students.

**Table 3: Phylogenetic clade 3**

| **COG-ID** | **Specimen collection date** | **Student case indicator*** |
| --- | --- | --- |
| Scotland/QEUH-9ADED5/2020 | 2020-09-05 | NA |
| Scotland/MILK-9C30D3/2020 | 2020-09-07 | NA |
| Scotland/QEUH-9BA44C/2020 | 2020-09-09 | NA |
| England/MILK-9C2E8F/2020 | 2020-09-10 | NA |
| Scotland/QEUH-9CA4B2/2020 | 2020-09-13 | NA |
| Scotland/QEUH-9CA42B/2020 | 2020-09-13 | NA |
| Scotland/QEUH-9C9B72/2020 | 2020-09-14 | NA |
| Scotland/QEUH-9C9B54/2020 | 2020-09-14 | NA |
| Scotland/CVR4428/2020 | 2020-09-14 | NA |
| Scotland/CVR4425/2020 | 2020-09-14 | NA |
| Scotland/QEUH-9C9240/2020 | 2020-09-14 | NA |
| Scotland/QEUH-9C7D23/2020 | 2020-09-15 | NA |
| Scotland/CVR4434/2020 | 2020-09-15 | NA |
| Scotland/CVR4430/2020 | 2020-09-15 | NA |
| Scotland/CVR4431/2020 | 2020-09-15 | NA |
| Scotland/CVR4427/2020 | 2020-09-15 | NA |
| Scotland/CVR4429/2020 | 2020-09-15 | NA |
| Scotland/CVR4433/2020 | 2020-09-15 | NA |
| Scotland/QEUH-9C879D/2020 | 2020-09-15 | NA |
| Scotland/EDB7776/2020 | 2020-09-16 | NA |
| Scotland/QEUH-9D03CB/2020 | 2020-09-16 | NA |
| Scotland/QEUH-9D0808/2020 | 2020-09-16 | NA |
| Scotland/QEUH-9CFDA3/2020 | 2020-09-16 | NA |
| England/ALDP-9CC3A4/2020 | 2020-09-16 | NA |
| Scotland/QEUH-9D11C0/2020 | 2020-09-16 | NA |
| Scotland/QEUH-9D0B1E/2020 | 2020-09-16 | NA |
| Scotland/QEUH-9CE278/2020 | 2020-09-16 | NA |
| Scotland/QEUH-9CFDFE/2020 | 2020-09-16 | NA |
| Scotland/QEUH-9D03E9/2020 | 2020-09-16 | NA |
| Scotland/QEUH-9CE296/2020 | 2020-09-16 | NA |
| Scotland/QEUH-9CE269/2020 | 2020-09-16 | NA |
| Scotland/QEUH-9CE2C3/2020 | 2020-09-16 | NA |
| Scotland/QEUH-9D155B/2020 | 2020-09-16 | NA |
| Scotland/QEUH-9D195F/2020 | 2020-09-16 | NA |
| Scotland/QEUH-9D096F/2020 | 2020-09-16 | NA |
| Scotland/QEUH-9D07FC/2020 | 2020-09-16 | NA |
| Scotland/QEUH-9CFCE2/2020 | 2020-09-16 | NA |
| Scotland/QEUH-9D0B4B/2020 | 2020-09-16 | NA |
| Scotland/QEUH-9D0CC0/2020 | 2020-09-16 | NA |
| Scotland/QEUH-9D020A/2020 | 2020-09-16 | NA |
| Scotland/QEUH-9D0219/2020 | 2020-09-16 | NA |
| Scotland/QEUH-9D08F9/2020 | 2020-09-16 | NA |
| Scotland/QEUH-9CE384/2020 | 2020-09-16 | NA |
| Scotland/QEUH-9CE24B/2020 | 2020-09-16 | NA |
| Scotland/QEUH-9CE2A5/2020 | 2020-09-16 | NA |
| Scotland/QEUH-9CE2D2/2020 | 2020-09-16 | NA |
| Scotland/QEUH-9CFCD3/2020 | 2020-09-16 | NA |
| Scotland/QEUH-9D08AE/2020 | 2020-09-16 | NA |
| Scotland/QEUH-9CF347/2020 | 2020-09-17 | NA |
| Scotland/QEUH-9CE797/2020 | 2020-09-17 | NA |
| England/QEUH-9CF3DE/2020 | 2020-09-17 | NA |
| Scotland/QEUH-9CEFBD/2020 | 2020-09-17 | NA |
| Scotland/QEUH-9CEEDE/2020 | 2020-09-17 | NA |
| Scotland/CVR4564/2020 | 2020-09-17 | NA |
| Scotland/QEUH-9CEEED/2020 | 2020-09-17 | NA |
| Scotland/QEUH-9CE928/2020 | 2020-09-17 | NA |
| Scotland/QEUH-9CEF9F/2020 | 2020-09-17 | NA |
| Scotland/QEUH-9CE937/2020 | 2020-09-17 | NA |
| Scotland/QEUH-9CEECF/2020 | 2020-09-17 | NA |
| Scotland/QEUH-9CF6E4/2020 | 2020-09-17 | NA |
| Scotland/QEUH-9CE5F7/2020 | 2020-09-17 | NA |
| Scotland/QEUH-9CEEFC/2020 | 2020-09-17 | NA |
| Scotland/QEUH-9CF6C6/2020 | 2020-09-17 | NA |
| Scotland/QEUH-9CF68A/2020 | 2020-09-17 | NA |
| Scotland/QEUH-9CEF26/2020 | 2020-09-17 | NA |
| Scotland/QEUH-9CFC3D/2020 | 2020-09-17 | NA |
| Scotland/QEUH-9CEF35/2020 | 2020-09-17 | NA |
| Scotland/QEUH-9CEF62/2020 | 2020-09-17 | NA |
| Scotland/QEUH-9CEA70/2020 | 2020-09-17 | NA |
| Scotland/QEUH-9CEA25/2020 | 2020-09-17 | NA |
| Scotland/QEUH-9CF699/2020 | 2020-09-17 | NA |
| England/ALDP-9DA18B/2020 | 2020-09-18 | NA |
| Scotland/CVR4394/2020 | 2020-09-18 | NA |
| Scotland/CVR4435/2020 | 2020-09-18 | NA |
| Scotland/QEUH-9D24A8/2020 | 2020-09-19 | NA |
| Scotland/QEUH-9D25B4/2020 | 2020-09-19 | NA |
| Scotland/CVR4445/2020 | 2020-09-19 | NA |
| Scotland/CVR4440/2020 | 2020-09-19 | NA |
| Scotland/CVR4441/2020 | 2020-09-19 | NA |
| England/MILK-9D44A6/2020 | 2020-09-19 | NA |
| Scotland/CVR4460/2020 | 2020-09-20 | H6 |
| Scotland/CVR4476/2020 | 2020-09-20 | H7 |
| Scotland/CVR4457/2020 | 2020-09-20 | NA |
| Scotland/CVR4456/2020 | 2020-09-20 | H6 |
| Scotland/CVR4776/2020 | 2020-09-20 | NA |
| Scotland/EDB7853/2020 | 2020-09-20 | NA |
| Scotland/CVR4462/2020 | 2020-09-20 | H3 |
| Scotland/EDB7858/2020 | 2020-09-20 | NA |
| Scotland/EDB7856/2020 | 2020-09-20 | NA |
| Scotland/CVR4778/2020 | 2020-09-20 | NA |
| Scotland/CVR4775/2020 | 2020-09-20 | NA |
| Scotland/QEUH-9DEA22/2020 | 2020-09-21 | NA |
| England/QEUH-9DC602/2020 | 2020-09-21 | NA |
| Scotland/CVR4520/2020 | 2020-09-21 | NA |
| Scotland/CVR4491/2020 | 2020-09-21 | H6 |
| Scotland/CVR4472/2020 | 2020-09-21 | H6 |
| Scotland/QEUH-9DE697/2020 | 2020-09-21 | H5 |
| Scotland/CVR5107/2020 | 2020-09-21 | NA |
| Scotland/CVR5104/2020 | 2020-09-21 | NA |
| Scotland/CVR4519/2020 | 2020-09-21 | H5 |
| Scotland/QEUH-9DE891/2020 | 2020-09-21 | NA |
| Scotland/EDB7826/2020 | 2020-09-21 | NA |
| Scotland/EDB7824/2020 | 2020-09-21 | NA |
| Scotland/EDB7827/2020 | 2020-09-21 | NA |
| Scotland/EDB7825/2020 | 2020-09-21 | NA |
| Scotland/EDB7812/2020 | 2020-09-21 | NA |
| Scotland/EDB7857/2020 | 2020-09-21 | NA |
| Scotland/EDB7828/2020 | 2020-09-21 | NA |
| Scotland/QEUH-9DD8A1/2020 | 2020-09-21 | NA |
| Scotland/QEUH-9DEAB9/2020 | 2020-09-21 | NA |
| Scotland/CVR4839/2020 | 2020-09-21 | NA |
| Scotland/QEUH-9DEAC8/2020 | 2020-09-21 | NA |
| Scotland/QEUH-9DE873/2020 | 2020-09-21 | NA |
| Scotland/QEUH-9DD57D/2020 | 2020-09-21 | NA |
| Scotland/QEUH-9DEAD7/2020 | 2020-09-21 | NA |
| Scotland/CVR5109/2020 | 2020-09-21 | NA |
| Scotland/CVR4507/2020 | 2020-09-21 | H8 |
| Scotland/QEUH-9DD7E0/2020 | 2020-09-21 | NA |
| Scotland/QEUH-9DE03F/2020 | 2020-09-21 | H5 |
| Scotland/QEUH-9DA5CB/2020 | 2020-09-22 | NA |
| Scotland/QEUH-9DD4BC/2020 | 2020-09-22 | NA |
| Scotland/QEUH-9DD4CB/2020 | 2020-09-22 | NA |
| Scotland/EDB7833/2020 | 2020-09-22 | NA |
| Scotland/QEUH-9DBEA1/2020 | 2020-09-22 | NA |
| Scotland/EDB7845/2020 | 2020-09-22 | NA |
| Scotland/EDB7839/2020 | 2020-09-22 | NA |
| Scotland/EDB7840/2020 | 2020-09-22 | NA |
| Scotland/QEUH-9DAD4B/2020 | 2020-09-22 | H5 |
| Scotland/EDB7851/2020 | 2020-09-22 | NA |
| Scotland/QEUH-9DC3CF/2020 | 2020-09-22 | NA |
| Scotland/EDB7860/2020 | 2020-09-22 | NA |
| Scotland/EDB7854/2020 | 2020-09-22 | NA |
| Scotland/EDB7810/2020 | 2020-09-22 | NA |
| Scotland/EDB7808/2020 | 2020-09-22 | NA |
| Scotland/EDB7811/2020 | 2020-09-22 | NA |
| Scotland/QEUH-9DC338/2020 | 2020-09-22 | NA |
| Scotland/EDB7837/2020 | 2020-09-22 | NA |
| Scotland/QEUH-9DABC9/2020 | 2020-09-22 | NA |
| Scotland/QEUH-9DAC02/2020 | 2020-09-22 | NA |
| Scotland/QEUH-9DAB32/2020 | 2020-09-22 | NA |
| Scotland/QEUH-9DABF6/2020 | 2020-09-22 | NA |
| Scotland/QEUH-9DC356/2020 | 2020-09-22 | NA |
| Scotland/CVR5401/2020 | 2020-09-22 | NA |
| Scotland/CVR5392/2020 | 2020-09-22 | NA |
| Scotland/CVR4546/2020 | 2020-09-22 | Private |
| Scotland/QEUH-9DC17A/2020 | 2020-09-22 | NA |
| Scotland/EDB7846/2020 | 2020-09-22 | NA |
| Scotland/EDB7843/2020 | 2020-09-22 | NA |
| Scotland/CVR5395/2020 | 2020-09-22 | NA |
| England/QEUH-9DC3B0/2020 | 2020-09-22 | NA |
| Scotland/EDB7844/2020 | 2020-09-22 | NA |
| Scotland/EDB7835/2020 | 2020-09-22 | NA |
| Scotland/CVR4537/2020 | 2020-09-22 | H5 |
| Scotland/CVR4549/2020 | 2020-09-22 | H5 |
| Scotland/EDB7838/2020 | 2020-09-22 | NA |
| Scotland/QEUH-9E4634/2020 | 2020-09-23 | NA |
| Scotland/QEUH-9E523F/2020 | 2020-09-23 | NA |
| Scotland/QEUH-9E5211/2020 | 2020-09-23 | NA |
| Scotland/QEUH-9E5202/2020 | 2020-09-23 | NA |
| Scotland/EDB7884/2020 | 2020-09-23 | NA |
| Scotland/EDB7876/2020 | 2020-09-23 | NA |
| Scotland/EDB7874/2020 | 2020-09-23 | NA |
| Scotland/QEUH-9E526C/2020 | 2020-09-23 | NA |
| Scotland/QEUH-9E5220/2020 | 2020-09-23 | NA |
| Scotland/EDB7877/2020 | 2020-09-23 | NA |
| Scotland/EDB7882/2020 | 2020-09-23 | NA |
| Scotland/EDB7881/2020 | 2020-09-23 | NA |
| Scotland/QEUH-9E524E/2020 | 2020-09-23 | NA |
| Scotland/EDB7886/2020 | 2020-09-23 | NA |
| Scotland/EDB7883/2020 | 2020-09-23 | NA |
| Scotland/EDB7871/2020 | 2020-09-23 | NA |
| Scotland/EDB7885/2020 | 2020-09-23 | NA |
| Scotland/QEUH-9E4485/2020 | 2020-09-23 | NA |
| Scotland/QEUH-9E485C/2020 | 2020-09-23 | H7 |
| Scotland/QEUH-9E3820/2020 | 2020-09-24 | NA |
| Scotland/QEUH-9E3547/2020 | 2020-09-24 | NA |
| Scotland/QEUH-9E54C0/2020 | 2020-09-24 | NA |
| Scotland/QEUH-9E52E4/2020 | 2020-09-24 | NA |
| Scotland/QEUH-9E384E/2020 | 2020-09-24 | NA |
| Scotland/QEUH-9E3529/2020 | 2020-09-24 | NA |
| Scotland/QEUH-9E3556/2020 | 2020-09-24 | NA |
| Scotland/QEUH-9F32E3/2020 | 2020-09-24 | NA |
| Scotland/QEUH-9E4D8A/2020 | 2020-09-24 | NA |
| Scotland/QEUH-9F3131/2020 | 2020-09-24 | NA |
| Scotland/QEUH-9F3122/2020 | 2020-09-24 | H5 |
| Scotland/QEUH-9F6110/2020 | 2020-09-25 | NA |
| England/QEUH-9F2CB9/2020 | 2020-09-25 | NA |
| Scotland/QEUH-9F9DCE/2020 | 2020-09-25 | NA |
| Scotland/QEUH-9F2D5C/2020 | 2020-09-25 | NA |
| Scotland/QEUH-9F2D01/2020 | 2020-09-25 | NA |
| Scotland/EDB7926/2020 | 2020-09-26 | NA |
| Scotland/EDB7927/2020 | 2020-09-26 | NA |
| Scotland/QEUH-9F4F63/2020 | 2020-09-27 | NA |
| Scotland/QEUH-9F67D2/2020 | 2020-09-27 | NA |
| Scotland/QEUH-9F85A9/2020 | 2020-09-27 | NA |
| Scotland/QEUH-9F5E0B/2020 | 2020-09-27 | NA |
| England/QEUH-9F5DD1/2020 | 2020-09-27 | NA |
| England/QEUH-9F8CB3/2020 | 2020-09-27 | NA |
| Scotland/QEUH-9F7E18/2020 | 2020-09-27 | NA |
| Scotland/QEUH-9F5339/2020 | 2020-09-27 | NA |
| Scotland/CVR4703/2020 | 2020-09-27 | NA |
| Scotland/QEUH-9F7DB1/2020 | 2020-09-27 | NA |
| Scotland/QEUH-9F8503/2020 | 2020-09-27 | NA |
| Scotland/QEUH-9F42C4/2020 | 2020-09-27 | NA |
| Scotland/QEUH-9F84BB/2020 | 2020-09-27 | NA |
| Scotland/QEUH-9F849D/2020 | 2020-09-27 | NA |
| Scotland/QEUH-9F46F5/2020 | 2020-09-27 | NA |
| Scotland/QEUH-9F67A5/2020 | 2020-09-27 | NA |
| England/QEUH-9F6884/2020 | 2020-09-27 | NA |
| Scotland/QEUH-9F6FE9/2020 | 2020-09-27 | NA |
| Scotland/QEUH-9F8DDE/2020 | 2020-09-27 | NA |
| Wales/QEUH-9F5FDB/2020 | 2020-09-27 | NA |
| Scotland/QEUH-9F46D7/2020 | 2020-09-27 | NA |
| Scotland/QEUH-9F5DE0/2020 | 2020-09-27 | NA |
| Scotland/QEUH-9F5DFF/2020 | 2020-09-27 | NA |
| Scotland/QEUH-9F7DFD/2020 | 2020-09-27 | NA |
| Scotland/QEUH-9F7DEE/2020 | 2020-09-27 | NA |
| Scotland/QEUH-9F6FDA/2020 | 2020-09-27 | NA |
| Scotland/QEUH-9F67B4/2020 | 2020-09-27 | NA |
| England/QEUH-9F8512/2020 | 2020-09-27 | NA |
| Scotland/QEUH-9F6893/2020 | 2020-09-27 | NA |
| Scotland/QEUH-9F7416/2020 | 2020-09-27 | NA |
| England/QEUH-9F7E45/2020 | 2020-09-27 | NA |
| Scotland/QEUH-9F7D57/2020 | 2020-09-27 | NA |
| Scotland/QEUH-9F52F0/2020 | 2020-09-27 | NA |
| Scotland/QEUH-9F8DFC/2020 | 2020-09-27 | NA |
| Scotland/QEUH-9F6FF8/2020 | 2020-09-27 | NA |
| Scotland/QEUH-9F4F72/2020 | 2020-09-27 | NA |
| Scotland/QEUH-9F52D2/2020 | 2020-09-27 | NA |
| Scotland/QEUH-9F7EFA/2020 | 2020-09-27 | NA |
| Scotland/QEUH-9F6FAD/2020 | 2020-09-27 | NA |
| Scotland/QEUH-9F6C2E/2020 | 2020-09-27 | NA |
| Scotland/QEUH-9F5E29/2020 | 2020-09-27 | NA |
| Scotland/QEUH-9F67C3/2020 | 2020-09-27 | NA |
| Scotland/QEUH-9F5D77/2020 | 2020-09-27 | NA |
| Scotland/QEUH-9F681B/2020 | 2020-09-27 | NA |
| Scotland/QEUH-9F6866/2020 | 2020-09-27 | NA |
| Scotland/QEUH-9F5D68/2020 | 2020-09-27 | NA |
| Scotland/QEUH-9F6936/2020 | 2020-09-27 | NA |
| Scotland/QEUH-9F848E/2020 | 2020-09-27 | NA |
| Scotland/QEUH-9F84CA/2020 | 2020-09-27 | NA |
| Scotland/QEUH-9F8521/2020 | 2020-09-27 | NA |
| Scotland/QEUH-9F847F/2020 | 2020-09-27 | NA |
| Scotland/QEUH-9F84F7/2020 | 2020-09-27 | NA |
| Scotland/QEUH-9F8E26/2020 | 2020-09-27 | NA |
| Scotland/QEUH-9F856D/2020 | 2020-09-27 | NA |
| Scotland/QEUH-9F8451/2020 | 2020-09-27 | NA |
| Scotland/QEUH-9F617A/2020 | 2020-09-27 | NA |
| Scotland/QEUH-9F7D2A/2020 | 2020-09-27 | NA |
| Scotland/QEUH-9F7D75/2020 | 2020-09-27 | NA |
| Scotland/QEUH-9F95F3/2020 | 2020-09-27 | NA |
| Scotland/QEUH-9F6839/2020 | 2020-09-27 | NA |
| Scotland/QEUH-9F6857/2020 | 2020-09-27 | NA |
| Scotland/QEUH-9F711F/2020 | 2020-09-27 | NA |
| Scotland/QEUH-9F680C/2020 | 2020-09-27 | NA |
| Scotland/QEUH-9F94C9/2020 | 2020-09-27 | NA |
| Scotland/QEUH-9F7DA2/2020 | 2020-09-27 | NA |
| Scotland/QEUH-9F5E47/2020 | 2020-09-27 | Private |
| Scotland/QEUH-9F572E/2020 | 2020-09-27 | H9 |
| Scotland/QEUH-9F4F18/2020 | 2020-09-27 | NA |
| Scotland/QEUH-9FA862/2020 | 2020-09-28 | NA |
| Scotland/QEUH-9FB45E/2020 | 2020-09-29 | NA |
| Scotland/QEUH-9FB403/2020 | 2020-09-29 | NA |
| Scotland/QEUH-9FA98D/2020 | 2020-09-29 | NA |
| Scotland/QEUH-9FB38E/2020 | 2020-09-29 | NA |
| Scotland/QEUH-9FB3BB/2020 | 2020-09-29 | NA |
| Scotland/QEUH-9FB421/2020 | 2020-09-29 | NA |
| England/QEUH-A1ED95/2020 | 2020-09-29 | NA |
| Scotland/QEUH-9FB3F7/2020 | 2020-09-29 | NA |
| Scotland/QEUH-9FB39D/2020 | 2020-09-29 | NA |
| Scotland/QEUH-9FB430/2020 | 2020-09-29 | NA |
| Scotland/QEUH-9FB412/2020 | 2020-09-29 | NA |
| Scotland/QEUH-9FB47C/2020 | 2020-09-29 | NA |
| Scotland/QEUH-9FB4A9/2020 | 2020-09-29 | NA |
| Scotland/QEUH-9FB48B/2020 | 2020-09-29 | NA |
| Scotland/QEUH-9FB3CA/2020 | 2020-09-29 | NA |
| Scotland/QEUH-9FA9C9/2020 | 2020-09-29 | NA |
| Scotland/QEUH-9FA914/2020 | 2020-09-29 | NA |
| Scotland/QEUH-9FB1DF/2020 | 2020-09-29 | NA |
| Scotland/QEUH-A006C8/2020 | 2020-09-30 | NA |
| Scotland/QEUH-A006D7/2020 | 2020-09-30 | NA |
| England/QEUH-9FF8B8/2020 | 2020-09-30 | NA |
| Scotland/QEUH-A00279/2020 | 2020-09-30 | NA |
| Scotland/QEUH-A20316/2020 | 2020-10-01 | NA |
| Scotland/QEUH-A203CB/2020 | 2020-10-01 | NA |
| England/QEUH-A014AF/2020 | 2020-10-01 | NA |
| Scotland/QEUH-A0C0BF/2020 | 2020-10-01 | NA |
| Scotland/QEUH-A1FF34/2020 | 2020-10-01 | NA |
| Scotland/QEUH-A20DDC/2020 | 2020-10-01 | NA |
| Scotland/QEUH-A1FF9E/2020 | 2020-10-01 | NA |
| Scotland/QEUH-A20DFA/2020 | 2020-10-01 | NA |
| Scotland/QEUH-A20E33/2020 | 2020-10-01 | NA |
| England/QEUH-A0B180/2020 | 2020-10-01 | NA |
| Scotland/QEUH-A20E51/2020 | 2020-10-01 | NA |
| Scotland/QEUH-A20DEB/2020 | 2020-10-01 | NA |
| Scotland/QEUH-A2097E/2020 | 2020-10-01 | NA |
| Scotland/QEUH-A20E06/2020 | 2020-10-01 | NA |
| Scotland/QEUH-A20D90/2020 | 2020-10-01 | NA |
| Scotland/QEUH-A20E15/2020 | 2020-10-01 | NA |
| Scotland/QEUH-A2154C/2020 | 2020-10-01 | NA |
| Scotland/QEUH-A21588/2020 | 2020-10-01 | NA |
| Scotland/EDB8245/2020 | 2020-10-01 | NA |
| Scotland/QEUH-A217DD/2020 | 2020-10-01 | NA |
| Scotland/QEUH-A21816/2020 | 2020-10-01 | NA |
| Scotland/QEUH-A20923/2020 | 2020-10-01 | NA |
| Scotland/QEUH-A20941/2020 | 2020-10-01 | NA |
| Scotland/QEUH-A208BD/2020 | 2020-10-01 | NA |
| Scotland/QEUH-A2104B/2020 | 2020-10-01 | NA |
| Scotland/QEUH-A2103C/2020 | 2020-10-01 | NA |
| Scotland/QEUH-A0B3E4/2020 | 2020-10-01 | NA |
| Scotland/QEUH-A0C6AE/2020 | 2020-10-01 | NA |
| Scotland/QEUH-A21658/2020 | 2020-10-01 | NA |
| Scotland/QEUH-A217FB/2020 | 2020-10-01 | NA |
| Scotland/QEUH-A210B4/2020 | 2020-10-01 | NA |
| Scotland/QEUH-A1FF70/2020 | 2020-10-01 | NA |
| Scotland/QEUH-A1EA16/2020 | 2020-10-01 | NA |
| Scotland/QEUH-A1E9EC/2020 | 2020-10-01 | NA |
| Scotland/QEUH-A00DD2/2020 | 2020-10-02 | NA |
| Scotland/QEUH-A00E1B/2020 | 2020-10-02 | NA |
| Scotland/CVR4843/2020 | 2020-10-02 | NA |
| Scotland/QEUH-A00789/2020 | 2020-10-02 | NA |
| England/BRIS-1346F1/2020 | 2020-10-02 | NA |
| England/BRIS-13470D/2020 | 2020-10-02 | NA |
| England/QEUH-A0077A/2020 | 2020-10-02 | NA |
| Scotland/QEUH-9FFE10/2020 | 2020-10-02 | NA |
| Scotland/QEUH-A007E3/2020 | 2020-10-02 | NA |
| Scotland/QEUH-A007C5/2020 | 2020-10-02 | NA |
| England/QEUH-9FFFFF/2020 | 2020-10-02 | NA |
| Scotland/CVR4854/2020 | 2020-10-02 | NA |
| Scotland/QEUH-9FFCE9/2020 | 2020-10-02 | NA |
| Scotland/QEUH-9FF681/2020 | 2020-10-02 | NA |
| Scotland/QEUH-A0117B/2020 | 2020-10-02 | NA |
| Scotland/CVR4927/2020 | 2020-10-02 | NA |
| Scotland/QEUH-A0B7CA/2020 | 2020-10-03 | NA |
| Scotland/QEUH-A0C29B/2020 | 2020-10-03 | NA |
| Scotland/QEUH-A0C459/2020 | 2020-10-03 | NA |
| Scotland/QEUH-A0C4C2/2020 | 2020-10-03 | NA |
| Scotland/QEUH-A0B706/2020 | 2020-10-03 | NA |
| Scotland/QEUH-A0C2C8/2020 | 2020-10-03 | NA |
| Scotland/QEUH-A0C2B9/2020 | 2020-10-03 | NA |
| Scotland/QEUH-A0C2AA/2020 | 2020-10-03 | NA |
| Scotland/QEUH-A0C301/2020 | 2020-10-03 | NA |
| Scotland/QEUH-A0C28C/2020 | 2020-10-03 | NA |
| Scotland/QEUH-A0B6DC/2020 | 2020-10-03 | NA |
| Scotland/QEUH-A0B8F4/2020 | 2020-10-03 | NA |
| Scotland/EDB8101/2020 | 2020-10-04 | NA |
| England/QEUH-A35EC2/2020 | 2020-10-04 | NA |
| Scotland/EDB8343/2020 | 2020-10-04 | NA |
| Scotland/CAMC-A3F718/2020 | 2020-10-05 | NA |
| Scotland/QEUH-A4CB2B/2020 | 2020-10-05 | NA |
| England/ALDP-A2F5A8/2020 | 2020-10-05 | NA |
| Scotland/EDB8346/2020 | 2020-10-05 | NA |
| Scotland/QEUH-A4BE9C/2020 | 2020-10-05 | NA |
| England/MILK-A46C97/2020 | 2020-10-06 | NA |
| Scotland/EDB10445/2020 | 2020-10-06 | NA |
| Scotland/CVR4885/2020 | 2020-10-06 | NA |
| Scotland/QEUH-A36C30/2020 | 2020-10-06 | NA |
| England/MILK-A45F17/2020 | 2020-10-06 | NA |
| England/CAMC-A41C7E/2020 | 2020-10-06 | NA |
| Scotland/QEUH-A4C46C/2020 | 2020-10-07 | NA |
| Scotland/QEUH-A4C4E4/2020 | 2020-10-07 | NA |
| Wales/QEUH-A4C4C6/2020 | 2020-10-07 | NA |
| Scotland/QEUH-A4C4B7/2020 | 2020-10-07 | NA |
| Scotland/QEUH-A378B3/2020 | 2020-10-07 | NA |
| Scotland/QEUH-A322BA/2020 | 2020-10-07 | NA |
| Scotland/EDB10448/2020 | 2020-10-08 | NA |
| Scotland/EDB10444/2020 | 2020-10-08 | NA |
| Scotland/QEUH-A4C851/2020 | 2020-10-08 | NA |
| Scotland/QEUH-A49001/2020 | 2020-10-08 | NA |
| Scotland/QEUH-A4903E/2020 | 2020-10-08 | NA |
| Scotland/EDB8352/2020 | 2020-10-08 | NA |
| Scotland/EDB8205/2020 | 2020-10-08 | NA |
| Scotland/QEUH-A4CCDD/2020 | 2020-10-08 | NA |
| Scotland/QEUH-A4B834/2020 | 2020-10-08 | NA |
| Scotland/QEUH-A4B51F/2020 | 2020-10-08 | NA |
| Scotland/QEUH-A4B53D/2020 | 2020-10-08 | NA |
| Scotland/QEUH-A4CCA0/2020 | 2020-10-08 | NA |
| Scotland/MILK-A6A3E3/2020 | 2020-10-09 | NA |
| Scotland/MILK-A6A9A5/2020 | 2020-10-09 | NA |
| Scotland/CAMC-A581B1/2020 | 2020-10-09 | NA |
| Scotland/MILK-A6B77D/2020 | 2020-10-09 | NA |
| Scotland/QEUH-A63960/2020 | 2020-10-09 | NA |
| Scotland/QEUH-A63B5B/2020 | 2020-10-09 | NA |
| Scotland/QEUH-A618DE/2020 | 2020-10-10 | NA |
| England/CAMC-A666FD/2020 | 2020-10-11 | NA |
| Scotland/QEUH-A5E643/2020 | 2020-10-11 | NA |
| Scotland/CAMC-A822CA/2020 | 2020-10-11 | NA |
| Scotland/EDB10122/2020 | 2020-10-12 | NA |
| England/QEUH-A792A8/2020 | 2020-10-12 | NA |
| England/ALDP-A66A1F/2020 | 2020-10-12 | NA |
| England/QEUH-A603DE/2020 | 2020-10-12 | NA |
| Scotland/EDB10447/2020 | 2020-10-13 | NA |
| Scotland/EDB10123/2020 | 2020-10-13 | NA |
| England/WSFT-25C524/2020 | 2020-10-13 | NA |
| Scotland/QEUH-A61C0F/2020 | 2020-10-13 | NA |
| Singapore/1105/2020 | 2020-10-14 | NA |
| Scotland/QEUH-A780CD/2020 | 2020-10-14 | NA |
| Scotland/QEUH-A78203/2020 | 2020-10-14 | NA |
| Scotland/EDB10121/2020 | 2020-10-14 | NA |
| Scotland/QEUH-A793E1/2020 | 2020-10-14 | NA |
| Scotland/QEUH-A78072/2020 | 2020-10-14 | NA |
| England/ALDP-A6DF37/2020 | 2020-10-14 | NA |
| Scotland/CVR5216/2020 | 2020-10-15 | NA |
| Scotland/EDB10124/2020 | 2020-10-16 | NA |
| Scotland/QEUH-A8A8B1/2020 | 2020-10-16 | NA |
| England/ALDP-A86642/2020 | 2020-10-17 | NA |
| England/ALDP-A84A84/2020 | 2020-10-18 | NA |
| Scotland/EDB10125/2020 | 2020-10-19 | NA |
| England/QEUH-AA03F0/2020 | 2020-10-19 | NA |
| Scotland/QEUH-A9D806/2020 | 2020-10-20 | NA |
| Scotland/QEUH-AAD311/2020 | 2020-10-20 | NA |
| England/QEUH-A9F3B8/2020 | 2020-10-20 | NA |
| England/MILK-ABC43A/2020 | 2020-10-20 | NA |
| Scotland/QEUH-A9D7DC/2020 | 2020-10-20 | NA |
| England/MILK-AB762A/2020 | 2020-10-20 | NA |
| Scotland/QEUH-A9D824/2020 | 2020-10-20 | NA |
| Scotland/QEUH-A9AE07/2020 | 2020-10-20 | NA |
| Scotland/QEUH-A9B63B/2020 | 2020-10-20 | NA |
| Scotland/QEUH-A9DB49/2020 | 2020-10-20 | NA |
| England/QEUH-A9FCAE/2020 | 2020-10-20 | NA |
| England/ALDP-AA5285/2020 | 2020-10-21 | NA |
| Scotland/QEUH-AAC42E/2020 | 2020-10-21 | NA |
| Scotland/QEUH-AAB647/2020 | 2020-10-21 | NA |
| Scotland/QEUH-AAB4E3/2020 | 2020-10-21 | NA |
| England/QEUH-AAC60A/2020 | 2020-10-21 | NA |
| Scotland/QEUH-AA069D/2020 | 2020-10-21 | NA |
| England/QEUH-AABC90/2020 | 2020-10-21 | NA |
| England/ALDP-AA2F45/2020 | 2020-10-22 | NA |
| Scotland/EDB8679/2020 | 2020-10-23 | NA |
| England/CAMC-AAF670/2020 | 2020-10-23 | NA |
| Scotland/EDB8662/2020 | 2020-10-24 | NA |
| England/MILK-B056B3/2020 | 2020-10-24 | NA |
| Scotland/EDB8683/2020 | 2020-10-24 | NA |
| Scotland/EDB8713/2020 | 2020-10-24 | NA |
| England/QEUH-AE3090/2020 | 2020-10-26 | NA |
| Scotland/QEUH-AD0218/2020 | 2020-10-26 | NA |
| England/QEUH-AD0236/2020 | 2020-10-26 | NA |
| England/QEUH-AD2EA9/2020 | 2020-10-26 | NA |
| England/CAMC-AEB186/2020 | 2020-10-26 | NA |
| Scotland/QEUH-AD0245/2020 | 2020-10-26 | NA |
| Scotland/QEUH-AD2A5A/2020 | 2020-10-27 | NA |
| Scotland/QEUH-AE4C04/2020 | 2020-10-28 | NA |
| England/CAMC-B0B0CE/2020 | 2020-10-29 | NA |
| England/MILK-B0688F/2020 | 2020-10-29 | NA |
| England/QEUH-AEF9C6/2020 | 2020-10-29 | NA |
| England/CAMC-B0B4D1/2020 | 2020-10-29 | NA |
| England/QEUH-AF4885/2020 | 2020-10-30 | NA |
| England/QEUH-AF5F70/2020 | 2020-10-30 | NA |
| England/QEUH-AF521D/2020 | 2020-10-30 | NA |
| England/MILK-B3E147/2020 | 2020-11-01 | NA |
| England/MILK-B398B0/2020 | 2020-11-01 | NA |
| England/MILK-B3CB96/2020 | 2020-11-01 | NA |
| England/MILK-B3B450/2020 | 2020-11-01 | NA |
| England/MILK-B3D7EC/2020 | 2020-11-01 | NA |
| England/CAMC-B20B86/2020 | 2020-11-01 | NA |
| England/CAMC-B20D26/2020 | 2020-11-02 | NA |
| England/QEUH-B11C0D/2020 | 2020-11-02 | NA |
| England/QEUH-B10A5F/2020 | 2020-11-02 | NA |
| England/QEUH-B10E9F/2020 | 2020-11-02 | NA |
| England/QEUH-B122FB/2020 | 2020-11-02 | NA |
| England/QEUH-B0F18B/2020 | 2020-11-02 | NA |
| England/QEUH-B0FFEB/2020 | 2020-11-02 | NA |
| England/QEUH-B12747/2020 | 2020-11-02 | NA |
| England/QEUH-B2DAF5/2020 | 2020-11-03 | NA |
| England/QEUH-B2D9E9/2020 | 2020-11-03 | NA |
| Scotland/EDB9266/2020 | 2020-11-03 | NA |
| Scotland/QEUH-B2B2A4/2020 | 2020-11-03 | NA |
| Scotland/QEUH-B132DC/2020 | 2020-11-03 | NA |
| England/LOND-DA12A/2020 | 2020-11-03 | NA |
| England/LOND-DA10C/2020 | 2020-11-03 | NA |
| England/CAMB-1B96F4/2020 | 2020-11-04 | NA |
| England/CAMB-1B9700/2020 | 2020-11-04 | NA |
| England/QEUH-B31DB9/2020 | 2020-11-04 | NA |
| England/QEUH-B31E89/2020 | 2020-11-04 | NA |
| Scotland/QEUH-B317D9/2020 | 2020-11-04 | NA |
| England/CAMC-B3228C/2020 | 2020-11-05 | NA |
| England/CAMC-B373B1/2020 | 2020-11-05 | NA |
| England/CAMC-B365E9/2020 | 2020-11-05 | NA |
| England/CAMC-B365DA/2020 | 2020-11-05 | NA |
| England/MILK-B559D9/2020 | 2020-11-05 | NA |
| England/MILK-B569C9/2020 | 2020-11-05 | NA |
| England/MILK-B54AC8/2020 | 2020-11-05 | NA |
| England/MILK-B59814/2020 | 2020-11-06 | NA |
| England/LOND-DA01E/2020 | 2020-11-07 | NA |
| England/ALDP-B6205C/2020 | 2020-11-08 | NA |
| England/ALDP-B5E2D5/2020 | 2020-11-08 | NA |
| England/ALDP-B6260F/2020 | 2020-11-08 | NA |
| England/CAMC-B54679/2020 | 2020-11-08 | NA |
| England/QEUH-B41A0A/2020 | 2020-11-08 | NA |
| England/QEUH-B3F2F8/2020 | 2020-11-08 | NA |
| England/ALDP-B5D1F7/2020 | 2020-11-09 | NA |
| England/ALDP-B5D50A/2020 | 2020-11-09 | NA |
| England/ALDP-B5D564/2020 | 2020-11-09 | NA |
| England/ALDP-B5B6AF/2020 | 2020-11-09 | NA |
| Denmark/DCGC-20335/2020 | 2020-11-09 | NA |
| England/QEUH-B66726/2020 | 2020-11-10 | NA |
| England/QEUH-B696CC/2020 | 2020-11-11 | NA |
| England/QEUH-B680FC/2020 | 2020-11-11 | NA |
| England/QEUH-B66E7C/2020 | 2020-11-11 | NA |
| England/QEUH-B66C09/2020 | 2020-11-11 | NA |
| England/QEUH-B6A6AD/2020 | 2020-11-11 | NA |
| England/QEUH-B6C483/2020 | 2020-11-11 | NA |
| England/QEUH-B66B2A/2020 | 2020-11-11 | NA |
| England/QEUH-B68083/2020 | 2020-11-11 | NA |
| England/QEUH-B66B1B/2020 | 2020-11-11 | NA |
| Scotland/QEUH-B69626/2020 | 2020-11-11 | NA |
| Scotland/QEUH-B69723/2020 | 2020-11-11 | NA |
| Scotland/QEUH-B69705/2020 | 2020-11-11 | NA |
| Scotland/QEUH-B696F9/2020 | 2020-11-11 | NA |
| Scotland/QEUH-B696AE/2020 | 2020-11-11 | NA |
| England/LOND-1250F2D/2020 | 2020-11-11 | NA |
| England/QEUH-B6728E/2020 | 2020-11-11 | NA |
| Scotland/QEUH-B69486/2020 | 2020-11-11 | NA |
| England/CAMC-B7AF81/2020 | 2020-11-12 | NA |
| England/LOND-DB420/2020 | 2020-11-13 | NA |
| England/QEUH-B7CE46/2020 | 2020-11-14 | NA |
| England/QEUH-B7F05C/2020 | 2020-11-14 | NA |
| England/QEUH-B7C5BA/2020 | 2020-11-14 | NA |
| Scotland/QEUH-B7EAE6/2020 | 2020-11-14 | NA |
| England/LOND-BD43F/2020 | 2020-11-14 | NA |
| Scotland/EDB9804/2020 | 2020-11-16 | NA |
| England/ALDP-BBACE8/2020 | 2020-11-18 | NA |
| Scotland/QEUH-B9FBFB/2020 | 2020-11-18 | NA |
| Scotland/QEUH-BA14A0/2020 | 2020-11-18 | NA |
| Scotland/QEUH-B9FC61/2020 | 2020-11-18 | NA |
| Scotland/QEUH-B9FF4A/2020 | 2020-11-18 | NA |
| Scotland/QEUH-B9FEC5/2020 | 2020-11-18 | NA |
| Scotland/QEUH-B9FCAD/2020 | 2020-11-18 | NA |
| Scotland/QEUH-B9FCDA/2020 | 2020-11-18 | NA |
| Scotland/QEUH-B9FCF8/2020 | 2020-11-18 | NA |
| Scotland/QEUH-B9FC25/2020 | 2020-11-18 | NA |
| Scotland/QEUH-B9FCCB/2020 | 2020-11-18 | NA |
| Scotland/EDB10046/2020 | 2020-11-19 | NA |
| England/SHEF-C9285/2020 | 2020-11-20 | NA |
| England/ALDP-BBA76F/2020 | 2020-11-20 | NA |
| England/ALDP-BB98E5/2020 | 2020-11-20 | NA |
| England/CAMC-BC1EBB/2020 | 2020-11-22 | NA |
| England/CAMC-BC1EAC/2020 | 2020-11-22 | NA |
| England/ALDP-BC7468/2020 | 2020-11-22 | NA |
| England/QEUH-BCDD76/2020 | 2020-11-26 | NA |
| Scotland/EDB10588/2020 | 2020-12-01 | NA |

*University of Glasgow student cases are indicated with ‘H’ and ‘Private’ denoting students residing in university halls and private accommodation respectively. NA indicates cases deemed to be non-students.

**Table 4: Phylogenetic clade 4**

| **COG-ID** | **Specimen collection date** | **Student case indicator*** |
| --- | --- | --- |
| England/NORT-286167/2020 | 2020-07-21 | NA |
| Scotland/QEUH-89C0D4/2020 | 2020-07-28 | NA |
| Scotland/QEUH-9439C1/2020 | 2020-07-31 | NA |
| Scotland/QEUH-9435AF/2020 | 2020-08-01 | NA |
| Scotland/QEUH-943439/2020 | 2020-08-01 | NA |
| Scotland/QEUH-943ABF/2020 | 2020-08-01 | NA |
| Scotland/EDB7118/2020 | 2020-08-01 | NA |
| Scotland/EDB7079/2020 | 2020-08-02 | NA |
| Scotland/EDB7080/2020 | 2020-08-02 | NA |
| England/NORT-286185/2020 | 2020-08-02 | NA |
| Scotland/EDB7088/2020 | 2020-08-02 | NA |
| Scotland/EDB7086/2020 | 2020-08-02 | NA |
| Scotland/EDB7081/2020 | 2020-08-02 | NA |
| Scotland/EDB7084/2020 | 2020-08-02 | NA |
| Scotland/EDB7106/2020 | 2020-08-03 | NA |
| Scotland/EDB7076/2020 | 2020-08-03 | NA |
| Scotland/EDB7116/2020 | 2020-08-03 | NA |
| Scotland/EDB7094/2020 | 2020-08-03 | NA |
| Scotland/EDB7102/2020 | 2020-08-03 | NA |
| Scotland/EDB7093/2020 | 2020-08-03 | NA |
| Scotland/EDB7107/2020 | 2020-08-03 | NA |
| Scotland/EDB7120/2020 | 2020-08-03 | NA |
| Scotland/EDB7115/2020 | 2020-08-03 | NA |
| Scotland/EDB7104/2020 | 2020-08-03 | NA |
| Scotland/EDB7103/2020 | 2020-08-03 | NA |
| Scotland/EDB7108/2020 | 2020-08-03 | NA |
| Scotland/EDB7155/2020 | 2020-08-03 | NA |
| Scotland/EDB7111/2020 | 2020-08-03 | NA |
| Scotland/EDB7105/2020 | 2020-08-03 | NA |
| Scotland/EDB7092/2020 | 2020-08-03 | NA |
| Scotland/EDB7110/2020 | 2020-08-03 | NA |
| Scotland/EDB7127/2020 | 2020-08-04 | NA |
| Scotland/QEUH-961347/2020 | 2020-08-04 | NA |
| Scotland/EDB7133/2020 | 2020-08-04 | NA |
| Scotland/EDB7139/2020 | 2020-08-04 | NA |
| Scotland/EDB7132/2020 | 2020-08-04 | NA |
| Scotland/EDB7151/2020 | 2020-08-04 | NA |
| Scotland/EDB7147/2020 | 2020-08-04 | NA |
| Scotland/QEUH-96131A/2020 | 2020-08-04 | NA |
| Scotland/EDB7123/2020 | 2020-08-04 | NA |
| Scotland/EDB7124/2020 | 2020-08-04 | NA |
| Scotland/EDB7136/2020 | 2020-08-04 | NA |
| Scotland/EDB7135/2020 | 2020-08-04 | NA |
| Scotland/EDB7149/2020 | 2020-08-04 | NA |
| Scotland/EDB7154/2020 | 2020-08-04 | NA |
| Scotland/EDB7126/2020 | 2020-08-04 | NA |
| Scotland/EDB7109/2020 | 2020-08-04 | NA |
| Scotland/EDB7148/2020 | 2020-08-04 | NA |
| Scotland/EDB7152/2020 | 2020-08-04 | NA |
| Scotland/EDB7156/2020 | 2020-08-04 | NA |
| Scotland/QEUH-95FE0F/2020 | 2020-08-05 | NA |
| Scotland/QEUH-9604AF/2020 | 2020-08-05 | NA |
| Scotland/QEUH-961189/2020 | 2020-08-05 | NA |
| Scotland/QEUH-960560/2020 | 2020-08-05 | NA |
| Scotland/QEUH-960797/2020 | 2020-08-05 | NA |
| Scotland/QEUH-96109B/2020 | 2020-08-05 | NA |
| Scotland/QEUH-960542/2020 | 2020-08-05 | NA |
| Scotland/QEUH-9606F4/2020 | 2020-08-05 | NA |
| Scotland/QEUH-960CF2/2020 | 2020-08-05 | NA |
| Scotland/QEUH-9611D4/2020 | 2020-08-05 | NA |
| Scotland/QEUH-95F6AD/2020 | 2020-08-05 | NA |
| Scotland/QEUH-9610B9/2020 | 2020-08-05 | NA |
| Scotland/QEUH-96122C/2020 | 2020-08-05 | NA |
| Scotland/EDB7146/2020 | 2020-08-05 | NA |
| Scotland/QEUH-96121D/2020 | 2020-08-05 | NA |
| Scotland/QEUH-96120E/2020 | 2020-08-05 | NA |
| Scotland/QEUH-96116B/2020 | 2020-08-05 | NA |
| Scotland/QEUH-960533/2020 | 2020-08-05 | NA |
| Scotland/QEUH-9611C5/2020 | 2020-08-05 | NA |
| Scotland/EDB7153/2020 | 2020-08-05 | NA |
| Scotland/EDB7150/2020 | 2020-08-05 | NA |
| Scotland/EDB7121/2020 | 2020-08-05 | NA |
| Scotland/EDB7142/2020 | 2020-08-05 | NA |
| Scotland/EDB7145/2020 | 2020-08-05 | NA |
| Scotland/EDB7140/2020 | 2020-08-05 | NA |
| Scotland/QEUH-9606E5/2020 | 2020-08-05 | NA |
| Scotland/QEUH-9610AA/2020 | 2020-08-05 | NA |
| Scotland/QEUH-9611B6/2020 | 2020-08-05 | NA |
| Scotland/QEUH-95F9D1/2020 | 2020-08-06 | NA |
| Scotland/QEUH-95F9C2/2020 | 2020-08-06 | NA |
| Scotland/QEUH-95EF3A/2020 | 2020-08-06 | NA |
| Scotland/QEUH-9600F6/2020 | 2020-08-06 | NA |
| Scotland/QEUH-960014/2020 | 2020-08-06 | NA |
| Scotland/QEUH-95FFEE/2020 | 2020-08-06 | NA |
| Scotland/QEUH-95FE1E/2020 | 2020-08-06 | NA |
| Scotland/QEUH-960C01/2020 | 2020-08-06 | NA |
| Scotland/QEUH-95F0DC/2020 | 2020-08-06 | NA |
| Scotland/QEUH-95F9E0/2020 | 2020-08-06 | NA |
| Scotland/QEUH-95FA92/2020 | 2020-08-06 | NA |
| Scotland/QEUH-95FB71/2020 | 2020-08-06 | NA |
| Scotland/QEUH-95E617/2020 | 2020-08-06 | NA |
| Scotland/QEUH-95FA47/2020 | 2020-08-06 | NA |
| Scotland/QEUH-95F713/2020 | 2020-08-06 | NA |
| Scotland/QEUH-95F616/2020 | 2020-08-06 | NA |
| Scotland/QEUH-95FBCC/2020 | 2020-08-06 | NA |
| Scotland/QEUH-960445/2020 | 2020-08-06 | NA |
| Scotland/QEUH-95F33D/2020 | 2020-08-06 | NA |
| Scotland/EDB7177/2020 | 2020-08-06 | NA |
| Scotland/QEUH-95F9FF/2020 | 2020-08-06 | NA |
| Scotland/QEUH-95FFFD/2020 | 2020-08-06 | NA |
| Scotland/QEUH-960032/2020 | 2020-08-06 | NA |
| Scotland/QEUH-960041/2020 | 2020-08-06 | NA |
| Scotland/QEUH-95F90E/2020 | 2020-08-06 | NA |
| Scotland/QEUH-960C10/2020 | 2020-08-06 | NA |
| Scotland/QEUH-95F9A4/2020 | 2020-08-06 | NA |
| Scotland/QEUH-95F9B3/2020 | 2020-08-06 | NA |
| Scotland/QEUH-960023/2020 | 2020-08-06 | NA |
| Scotland/QEUH-960C2F/2020 | 2020-08-06 | NA |
| Scotland/QEUH-95FA1A/2020 | 2020-08-06 | NA |
| Scotland/QEUH-95F27C/2020 | 2020-08-06 | NA |
| Scotland/QEUH-95FF66/2020 | 2020-08-07 | NA |
| Scotland/QEUH-95FE78/2020 | 2020-08-07 | NA |
| Scotland/QEUH-95DFA4/2020 | 2020-08-07 | NA |
| Scotland/QEUH-95DF95/2020 | 2020-08-07 | NA |
| Scotland/QEUH-95FCD8/2020 | 2020-08-07 | NA |
| Scotland/QEUH-96073D/2020 | 2020-08-07 | NA |
| Scotland/QEUH-95F106/2020 | 2020-08-07 | NA |
| Scotland/QEUH-95FF39/2020 | 2020-08-07 | NA |
| Scotland/QEUH-95E662/2020 | 2020-08-07 | NA |
| Scotland/EDB7353/2020 | 2020-08-07 | NA |
| Scotland/QEUH-960515/2020 | 2020-08-07 | NA |
| Scotland/EDB7181/2020 | 2020-08-07 | NA |
| Scotland/QEUH-95FCF6/2020 | 2020-08-07 | NA |
| Scotland/QEUH-95E082/2020 | 2020-08-07 | NA |
| Scotland/QEUH-95FDB7/2020 | 2020-08-07 | NA |
| Scotland/QEUH-96076A/2020 | 2020-08-07 | NA |
| Scotland/QEUH-96072E/2020 | 2020-08-07 | NA |
| Scotland/QEUH-95F740/2020 | 2020-08-08 | NA |
| Scotland/QEUH-95F731/2020 | 2020-08-08 | NA |
| Scotland/QEUH-95F4FE/2020 | 2020-08-08 | NA |
| Scotland/QEUH-95F4EF/2020 | 2020-08-08 | NA |
| Scotland/QEUH-95F41C/2020 | 2020-08-08 | NA |
| Scotland/QEUH-95E152/2020 | 2020-08-08 | NA |
| Scotland/QEUH-95E1F8/2020 | 2020-08-08 | NA |
| Scotland/QEUH-95E091/2020 | 2020-08-08 | NA |
| Scotland/QEUH-95EDA9/2020 | 2020-08-08 | NA |
| Scotland/QEUH-95ED03/2020 | 2020-08-08 | NA |
| Scotland/QEUH-95EE97/2020 | 2020-08-08 | NA |
| Scotland/EDB7178/2020 | 2020-08-08 | NA |
| Scotland/QEUH-95ECF7/2020 | 2020-08-08 | NA |
| Scotland/QEUH-95EEA6/2020 | 2020-08-08 | NA |
| Scotland/QEUH-95E44A/2020 | 2020-08-08 | NA |
| Scotland/QEUH-96355E/2020 | 2020-08-08 | NA |
| Scotland/QEUH-95F40D/2020 | 2020-08-08 | NA |
| Scotland/QEUH-95ED6D/2020 | 2020-08-08 | NA |
| Scotland/QEUH-95E8F3/2020 | 2020-08-09 | NA |
| Scotland/QEUH-95E653/2020 | 2020-08-09 | NA |
| Scotland/QEUH-95E86C/2020 | 2020-08-09 | NA |
| Scotland/QEUH-95E85D/2020 | 2020-08-09 | NA |
| Scotland/QEUH-95F1D9/2020 | 2020-08-09 | NA |
| Scotland/QEUH-95E35C/2020 | 2020-08-09 | NA |
| Scotland/QEUH-95E88A/2020 | 2020-08-09 | NA |
| Scotland/QEUH-95EA0C/2020 | 2020-08-09 | NA |
| Scotland/QEUH-963002/2020 | 2020-08-09 | NA |
| Scotland/QEUH-95E161/2020 | 2020-08-09 | NA |
| Scotland/QEUH-96372B/2020 | 2020-08-09 | NA |
| Scotland/QEUH-95EA39/2020 | 2020-08-09 | NA |
| Scotland/QEUH-95E8B7/2020 | 2020-08-09 | NA |
| Scotland/QEUH-95EA48/2020 | 2020-08-09 | NA |
| Scotland/QEUH-95EA1B/2020 | 2020-08-09 | NA |
| Scotland/QEUH-95E90F/2020 | 2020-08-09 | NA |
| Scotland/QEUH-95EA57/2020 | 2020-08-09 | NA |
| Scotland/QEUH-95E8A8/2020 | 2020-08-09 | NA |
| Scotland/QEUH-95EA2A/2020 | 2020-08-09 | NA |
| Scotland/QEUH-95E19E/2020 | 2020-08-10 | NA |
| Scotland/QEUH-95E1AD/2020 | 2020-08-10 | NA |
| Scotland/QEUH-96357C/2020 | 2020-08-10 | NA |
| Scotland/QEUH-961F34/2020 | 2020-08-10 | NA |
| Scotland/QEUH-961F61/2020 | 2020-08-10 | NA |
| Scotland/QEUH-95E468/2020 | 2020-08-10 | NA |
| Scotland/QEUH-963907/2020 | 2020-08-10 | NA |
| Scotland/QEUH-961FDA/2020 | 2020-08-10 | NA |
| Scotland/QEUH-965AE4/2020 | 2020-08-10 | NA |
| Scotland/QEUH-9636E2/2020 | 2020-08-10 | NA |
| Scotland/QEUH-95E26E/2020 | 2020-08-10 | NA |
| Scotland/QEUH-962B02/2020 | 2020-08-10 | NA |
| Scotland/QEUH-962FE8/2020 | 2020-08-10 | NA |
| Scotland/QEUH-95E4C2/2020 | 2020-08-10 | NA |
| Scotland/QEUH-961FBC/2020 | 2020-08-10 | NA |
| Scotland/QEUH-963530/2020 | 2020-08-10 | NA |
| Scotland/QEUH-96363D/2020 | 2020-08-10 | NA |
| Scotland/QEUH-962276/2020 | 2020-08-10 | NA |
| Scotland/QEUH-95E459/2020 | 2020-08-10 | NA |
| Scotland/QEUH-95E477/2020 | 2020-08-10 | NA |
| Scotland/QEUH-961F9E/2020 | 2020-08-10 | NA |
| Scotland/EDB7354/2020 | 2020-08-10 | NA |
| Scotland/EDB7184/2020 | 2020-08-10 | NA |
| Scotland/QEUH-961FAD/2020 | 2020-08-10 | NA |
| Scotland/QEUH-9639BC/2020 | 2020-08-10 | NA |
| Scotland/EDB7186/2020 | 2020-08-10 | NA |
| Scotland/QEUH-9621D3/2020 | 2020-08-10 | NA |
| Scotland/QEUH-962328/2020 | 2020-08-10 | NA |
| Scotland/QEUH-95E27D/2020 | 2020-08-10 | NA |
| Scotland/QEUH-964AF4/2020 | 2020-08-11 | NA |
| Scotland/QEUH-964D28/2020 | 2020-08-11 | NA |
| Scotland/QEUH-964344/2020 | 2020-08-11 | NA |
| Scotland/QEUH-96314B/2020 | 2020-08-11 | NA |
| Scotland/QEUH-96315A/2020 | 2020-08-11 | NA |
| Scotland/QEUH-963354/2020 | 2020-08-11 | NA |
| Scotland/QEUH-962C69/2020 | 2020-08-11 | NA |
| Scotland/QEUH-962FCA/2020 | 2020-08-11 | NA |
| Scotland/QEUH-963345/2020 | 2020-08-11 | NA |
| Scotland/QEUH-964A6D/2020 | 2020-08-11 | NA |
| Scotland/QEUH-962B20/2020 | 2020-08-11 | NA |
| Scotland/QEUH-9631D2/2020 | 2020-08-11 | NA |
| Scotland/QEUH-9631E1/2020 | 2020-08-11 | NA |
| Scotland/QEUH-962B5D/2020 | 2020-08-11 | NA |
| Scotland/QEUH-9629BD/2020 | 2020-08-11 | NA |
| Scotland/QEUH-962B11/2020 | 2020-08-11 | NA |
| Scotland/QEUH-962C78/2020 | 2020-08-11 | NA |
| Scotland/QEUH-962B3F/2020 | 2020-08-11 | NA |
| Scotland/QEUH-962EDC/2020 | 2020-08-11 | NA |
| Scotland/QEUH-962BA8/2020 | 2020-08-11 | NA |
| Scotland/EDB7355/2020 | 2020-08-11 | NA |
| Scotland/EDB7185/2020 | 2020-08-11 | NA |
| England/QEUH-963381/2020 | 2020-08-11 | NA |
| Scotland/QEUH-964CB2/2020 | 2020-08-11 | NA |
| Scotland/QEUH-964A5E/2020 | 2020-08-11 | NA |
| Scotland/QEUH-963178/2020 | 2020-08-11 | NA |
| Scotland/QEUH-96280B/2020 | 2020-08-11 | NA |
| Scotland/QEUH-964B1F/2020 | 2020-08-11 | NA |
| Scotland/QEUH-964A4F/2020 | 2020-08-11 | NA |
| England/QEUH-96538F/2020 | 2020-08-11 | NA |
| Scotland/QEUH-96313C/2020 | 2020-08-11 | NA |
| Scotland/QEUH-962C0F/2020 | 2020-08-11 | NA |
| Scotland/QEUH-96303F/2020 | 2020-08-11 | NA |
| Scotland/QEUH-9627FF/2020 | 2020-08-11 | NA |
| Scotland/QEUH-9640C5/2020 | 2020-08-12 | NA |
| Scotland/QEUH-964308/2020 | 2020-08-12 | NA |
| Scotland/QEUH-963AE6/2020 | 2020-08-12 | NA |
| Scotland/QEUH-9655E3/2020 | 2020-08-12 | NA |
| Scotland/QEUH-96A663/2020 | 2020-08-12 | NA |
| Scotland/EDB7358/2020 | 2020-08-12 | NA |
| Scotland/QEUH-96407A/2020 | 2020-08-12 | NA |
| Scotland/QEUH-964089/2020 | 2020-08-12 | NA |
| Scotland/EDB7357/2020 | 2020-08-12 | NA |
| England/QEUH-963AC8/2020 | 2020-08-12 | NA |
| Scotland/QEUH-963DFC/2020 | 2020-08-12 | NA |
| Scotland/QEUH-963A7D/2020 | 2020-08-12 | NA |
| Scotland/QEUH-9640D4/2020 | 2020-08-12 | NA |
| Scotland/QEUH-963A8C/2020 | 2020-08-12 | NA |
| Scotland/QEUH-963A6E/2020 | 2020-08-12 | NA |
| Scotland/QEUH-963E08/2020 | 2020-08-12 | NA |
| Scotland/QEUH-963BE3/2020 | 2020-08-12 | NA |
| Scotland/QEUH-964195/2020 | 2020-08-12 | NA |
| Scotland/QEUH-963F50/2020 | 2020-08-12 | NA |
| Scotland/QEUH-963FAB/2020 | 2020-08-12 | NA |
| Scotland/QEUH-964010/2020 | 2020-08-12 | NA |
| Scotland/QEUH-964B3D/2020 | 2020-08-13 | NA |
| Scotland/QEUH-96506A/2020 | 2020-08-13 | NA |
| Scotland/EDB7359/2020 | 2020-08-13 | NA |
| England/CAMB-1B5B17/2020 | 2020-08-13 | NA |
| England/CAMB-1B5B62/2020 | 2020-08-13 | NA |
| England/CAMB-1B5B26/2020 | 2020-08-13 | NA |
| England/CAMB-1B5B44/2020 | 2020-08-13 | NA |
| England/CAMB-1B5B35/2020 | 2020-08-13 | NA |
| England/CAMB-1B5B53/2020 | 2020-08-13 | NA |
| Scotland/QEUH-965079/2020 | 2020-08-13 | NA |
| Scotland/QEUH-96A672/2020 | 2020-08-13 | NA |
| Scotland/EDB7274/2020 | 2020-08-13 | NA |
| Scotland/QEUH-96A654/2020 | 2020-08-13 | NA |
| Scotland/QEUH-965088/2020 | 2020-08-13 | NA |
| England/QEUH-964D91/2020 | 2020-08-13 | NA |
| England/QEUH-964BF1/2020 | 2020-08-13 | NA |
| Scotland/QEUH-964D46/2020 | 2020-08-13 | NA |
| England/QEUH-964D73/2020 | 2020-08-13 | NA |
| England/QEUH-964B88/2020 | 2020-08-13 | NA |
| Scotland/QEUH-96A1BD/2020 | 2020-08-13 | NA |
| Scotland/QEUH-96C4EF/2020 | 2020-08-13 | NA |
| Scotland/QEUH-964A21/2020 | 2020-08-13 | NA |
| Scotland/QEUH-964B4C/2020 | 2020-08-13 | NA |
| Scotland/QEUH-96BBFA/2020 | 2020-08-13 | NA |
| Scotland/QEUH-964F8C/2020 | 2020-08-13 | NA |
| Scotland/QEUH-969707/2020 | 2020-08-14 | NA |
| Scotland/QEUH-969646/2020 | 2020-08-14 | NA |
| Scotland/QEUH-965774/2020 | 2020-08-14 | NA |
| Scotland/QEUH-9697E9/2020 | 2020-08-14 | NA |
| Scotland/QEUH-969840/2020 | 2020-08-14 | NA |
| Scotland/QEUH-965589/2020 | 2020-08-14 | NA |
| Scotland/QEUH-969743/2020 | 2020-08-14 | NA |
| Scotland/QEUH-965792/2020 | 2020-08-14 | NA |
| Scotland/QEUH-965765/2020 | 2020-08-14 | NA |
| Scotland/QEUH-9657A1/2020 | 2020-08-14 | NA |
| Scotland/QEUH-9659BA/2020 | 2020-08-14 | NA |
| Scotland/QEUH-96986E/2020 | 2020-08-14 | NA |
| Scotland/QEUH-965334/2020 | 2020-08-14 | NA |
| Scotland/QEUH-9697DA/2020 | 2020-08-14 | NA |
| Scotland/QEUH-969637/2020 | 2020-08-14 | NA |
| Scotland/QEUH-969752/2020 | 2020-08-14 | NA |
| Scotland/EDB7360/2020 | 2020-08-14 | NA |
| Scotland/EDB7275/2020 | 2020-08-14 | NA |
| England/QEUH-96978F/2020 | 2020-08-14 | NA |
| England/QEUH-9697AD/2020 | 2020-08-14 | NA |
| England/QEUH-9697CB/2020 | 2020-08-14 | NA |
| England/QEUH-9695EF/2020 | 2020-08-14 | NA |
| England/QEUH-96BCF7/2020 | 2020-08-14 | NA |
| England/QEUH-96B43B/2020 | 2020-08-14 | NA |
| England/QEUH-96B723/2020 | 2020-08-14 | NA |
| England/QEUH-9657CF/2020 | 2020-08-14 | NA |
| England/QEUH-9695FE/2020 | 2020-08-14 | NA |
| England/QEUH-969734/2020 | 2020-08-14 | NA |
| England/QEUH-96979E/2020 | 2020-08-14 | NA |
| England/QEUH-96589F/2020 | 2020-08-14 | NA |
| Scotland/QEUH-96B0DD/2020 | 2020-08-14 | NA |
| Scotland/QEUH-969549/2020 | 2020-08-14 | NA |
| Scotland/QEUH-9698C8/2020 | 2020-08-14 | NA |
| Scotland/QEUH-9698B9/2020 | 2020-08-14 | NA |
| Scotland/QEUH-965598/2020 | 2020-08-14 | NA |
| Scotland/QEUH-96A28D/2020 | 2020-08-15 | NA |
| Scotland/QEUH-96C212/2020 | 2020-08-15 | NA |
| Scotland/QEUH-96C203/2020 | 2020-08-15 | NA |
| England/QEUH-96BE97/2020 | 2020-08-15 | NA |
| England/QEUH-96A2E7/2020 | 2020-08-15 | NA |
| England/QEUH-96A205/2020 | 2020-08-15 | NA |
| England/QEUH-96C2E5/2020 | 2020-08-15 | NA |
| England/QEUH-96BEB5/2020 | 2020-08-15 | NA |
| England/QEUH-96BEA6/2020 | 2020-08-15 | NA |
| England/QEUH-96C124/2020 | 2020-08-15 | NA |
| England/QEUH-96F21F/2020 | 2020-08-15 | NA |
| England/QEUH-96A214/2020 | 2020-08-15 | NA |
| Scotland/QEUH-96C28B/2020 | 2020-08-15 | NA |
| Scotland/QEUH-96B77E/2020 | 2020-08-16 | NA |
| Wales/MILK-971010/2020 | 2020-08-16 | NA |
| Wales/MILK-971001/2020 | 2020-08-16 | NA |
| Scotland/QEUH-96ADE6/2020 | 2020-08-16 | NA |
| Scotland/EDB7276/2020 | 2020-08-16 | NA |
| Scotland/QEUH-96B635/2020 | 2020-08-17 | NA |
| Scotland/QEUH-96A9E2/2020 | 2020-08-17 | NA |
| Scotland/QEUH-96B644/2020 | 2020-08-17 | NA |
| Scotland/QEUH-96B44A/2020 | 2020-08-17 | NA |
| Wales/MILK-97020C/2020 | 2020-08-17 | NA |
| Scotland/QEUH-96ACAD/2020 | 2020-08-17 | NA |
| England/NORT-28CBAF/2020 | 2020-08-17 | NA |
| England/QEUH-96B556/2020 | 2020-08-17 | NA |
| Scotland/QEUH-96AC34/2020 | 2020-08-17 | NA |
| England/QEUH-96FF90/2020 | 2020-08-17 | NA |
| Scotland/QEUH-96B653/2020 | 2020-08-17 | NA |
| Scotland/QEUH-96EF64/2020 | 2020-08-18 | NA |
| Scotland/QEUH-96B240/2020 | 2020-08-18 | NA |
| Scotland/QEUH-96EF73/2020 | 2020-08-18 | NA |
| Scotland/QEUH-9700F3/2020 | 2020-08-18 | NA |
| Wales/MILK-970B89/2020 | 2020-08-18 | NA |
| Wales/MILK-9712ED/2020 | 2020-08-18 | NA |
| Wales/MILK-970B6B/2020 | 2020-08-18 | NA |
| Wales/MILK-9712B0/2020 | 2020-08-18 | NA |
| Wales/MILK-9712DE/2020 | 2020-08-18 | NA |
| England/MILK-975173/2020 | 2020-08-18 | NA |
| England/QEUH-96FC8A/2020 | 2020-08-18 | NA |
| England/QEUH-97005D/2020 | 2020-08-18 | NA |
| England/QEUH-96FB50/2020 | 2020-08-18 | NA |
| Scotland/QEUH-96B26E/2020 | 2020-08-18 | NA |
| Scotland/QEUH-96EDE2/2020 | 2020-08-19 | NA |
| England/CAMB-1B5CAB/2020 | 2020-08-19 | NA |
| Wales/MILK-973588/2020 | 2020-08-19 | NA |
| Wales/MILK-9762D9/2020 | 2020-08-19 | NA |
| Wales/MILK-975CBE/2020 | 2020-08-19 | NA |
| Scotland/EDB7372/2020 | 2020-08-19 | NA |
| Scotland/QEUH-96E84B/2020 | 2020-08-19 | NA |
| Scotland/QEUH-96D31E/2020 | 2020-08-20 | NA |
| Scotland/QEUH-96D3B4/2020 | 2020-08-20 | NA |
| Scotland/QEUH-96D32D/2020 | 2020-08-20 | NA |
| England/MILK-97353D/2020 | 2020-08-20 | NA |
| England/MILK-9735D3/2020 | 2020-08-20 | NA |
| England/MILK-9758F6/2020 | 2020-08-20 | NA |
| Wales/MILK-973139/2020 | 2020-08-20 | NA |
| Wales/MILK-973175/2020 | 2020-08-20 | NA |
| Wales/MILK-973157/2020 | 2020-08-20 | NA |
| Wales/MILK-97312A/2020 | 2020-08-20 | NA |
| Scotland/QEUH-96D581/2020 | 2020-08-20 | NA |
| England/NORT-28CBDC/2020 | 2020-08-20 | NA |
| England/QEUH-96E23E/2020 | 2020-08-20 | NA |
| Scotland/QEUH-96E210/2020 | 2020-08-20 | NA |
| England/QEUH-96E201/2020 | 2020-08-20 | NA |
| England/QEUH-96E22F/2020 | 2020-08-20 | NA |
| Scotland/EDB7271/2020 | 2020-08-20 | NA |
| Scotland/QEUH-96D4C0/2020 | 2020-08-20 | NA |
| Scotland/QEUH-96D28A/2020 | 2020-08-21 | NA |
| Scotland/QEUH-96C810/2020 | 2020-08-21 | NA |
| Scotland/QEUH-96CCBA/2020 | 2020-08-21 | NA |
| Scotland/QEUH-96C79B/2020 | 2020-08-21 | NA |
| Wales/MILK-976840/2020 | 2020-08-21 | NA |
| Wales/PHWC-169F70/2020 | 2020-08-21 | NA |
| Wales/MILK-97689B/2020 | 2020-08-21 | NA |
| Wales/MILK-993DD5/2020 | 2020-08-21 | NA |
| Scotland/QEUH-96C7AA/2020 | 2020-08-21 | NA |
| England/MILK-974842/2020 | 2020-08-21 | NA |
| England/MILK-97535F/2020 | 2020-08-21 | NA |
| England/MILK-993DE4/2020 | 2020-08-22 | NA |
| Scotland/QEUH-96D23F/2020 | 2020-08-22 | NA |
| England/MILK-9959A2/2020 | 2020-08-23 | NA |
| England/MILK-995984/2020 | 2020-08-23 | NA |
| England/MILK-995993/2020 | 2020-08-23 | NA |
| England/NORT-28CBEB/2020 | 2020-08-23 | NA |
| England/QEUH-998893/2020 | 2020-08-23 | NA |
| England/QEUH-998972/2020 | 2020-08-23 | NA |
| England/MILK-995966/2020 | 2020-08-23 | NA |
| England/QEUH-997CE3/2020 | 2020-08-24 | NA |
| England/QEUH-998F52/2020 | 2020-08-24 | NA |
| England/MILK-995359/2020 | 2020-08-24 | NA |
| Wales/PHWC-169E37/2020 | 2020-08-24 | NA |
| England/NORT-2861A3/2020 | 2020-08-24 | NA |
| England/QEUH-997D59/2020 | 2020-08-24 | NA |
| England/MILK-994369/2020 | 2020-08-24 | NA |
| Scotland/EDB7376/2020 | 2020-08-24 | NA |
| England/QEUH-99906D/2020 | 2020-08-24 | NA |
| Scotland/EDB7374/2020 | 2020-08-24 | NA |
| Scotland/QEUH-990136/2020 | 2020-08-25 | NA |
| England/MILK-9941F6/2020 | 2020-08-25 | NA |
| England/QEUH-98F2CE/2020 | 2020-08-25 | NA |
| Wales/MILK-99476D/2020 | 2020-08-25 | NA |
| England/MILK-994484/2020 | 2020-08-25 | NA |
| England/MILK-994897/2020 | 2020-08-25 | NA |
| Scotland/EDB7382/2020 | 2020-08-25 | NA |
| England/MILK-994615/2020 | 2020-08-25 | NA |
| England/MILK-994105/2020 | 2020-08-25 | NA |
| England/QEUH-98F0A6/2020 | 2020-08-25 | NA |
| Wales/MILK-993F66/2020 | 2020-08-25 | NA |
| Wales/MILK-995007/2020 | 2020-08-25 | NA |
| England/MILK-99434B/2020 | 2020-08-25 | NA |
| England/MILK-994AA0/2020 | 2020-08-25 | NA |
| England/MILK-995AFA/2020 | 2020-08-25 | NA |
| England/MILK-994202/2020 | 2020-08-25 | NA |
| England/MILK-994A91/2020 | 2020-08-25 | NA |
| Wales/MILK-994FB0/2020 | 2020-08-25 | NA |
| United_Arab_Emirates/skmc-3079344/2020 | 2020-08-26 | NA |
| Scotland/QEUH-998E37/2020 | 2020-08-26 | NA |
| England/MILK-992D21/2020 | 2020-08-26 | NA |
| Scotland/QEUH-9900EE/2020 | 2020-08-26 | NA |
| Wales/MILK-992C51/2020 | 2020-08-26 | NA |
| Wales/MILK-9931BB/2020 | 2020-08-26 | NA |
| England/MILK-992D5E/2020 | 2020-08-26 | NA |
| England/MILK-99386B/2020 | 2020-08-26 | NA |
| England/MILK-99394A/2020 | 2020-08-26 | NA |
| England/MILK-993A0B/2020 | 2020-08-26 | NA |
| England/MILK-993968/2020 | 2020-08-26 | NA |
| England/MILK-99423F/2020 | 2020-08-26 | NA |
| England/MILK-992D7C/2020 | 2020-08-26 | NA |
| Wales/MILK-993A29/2020 | 2020-08-26 | NA |
| England/MILK-99341C/2020 | 2020-08-26 | NA |
| Wales/MILK-9936BC/2020 | 2020-08-26 | NA |
| England/QEUH-9906FB/2020 | 2020-08-26 | NA |
| Scotland/QEUH-9903F4/2020 | 2020-08-26 | NA |
| England/MILK-992D8B/2020 | 2020-08-26 | NA |
| Scotland/QEUH-98FFC7/2020 | 2020-08-26 | NA |
| England/MILK-992CBB/2020 | 2020-08-26 | NA |
| England/QEUH-9981B6/2020 | 2020-08-26 | NA |
| England/QEUH-998BD6/2020 | 2020-08-26 | NA |
| England/QEUH-998BB8/2020 | 2020-08-26 | NA |
| England/QEUH-9981A7/2020 | 2020-08-26 | NA |
| England/QEUH-99041F/2020 | 2020-08-26 | NA |
| Wales/MILK-992CF7/2020 | 2020-08-26 | NA |
| England/QEUH-990655/2020 | 2020-08-26 | NA |
| England/MILK-9919C4/2020 | 2020-08-26 | NA |
| England/MILK-9919B5/2020 | 2020-08-26 | NA |
| England/MILK-992ED3/2020 | 2020-08-26 | NA |
| England/MILK-991C07/2020 | 2020-08-27 | NA |
| Scotland/QEUH-98F9D8/2020 | 2020-08-27 | NA |
| Wales/MILK-9928D5/2020 | 2020-08-27 | NA |
| England/MILK-993124/2020 | 2020-08-27 | NA |
| England/MILK-993106/2020 | 2020-08-27 | NA |
| England/QEUH-998AE8/2020 | 2020-08-27 | NA |
| England/MILK-992750/2020 | 2020-08-27 | NA |
| England/QEUH-9963D0/2020 | 2020-08-27 | NA |
| England/MILK-991C16/2020 | 2020-08-27 | NA |
| England/MILK-991BEC/2020 | 2020-08-27 | NA |
| England/MILK-991B28/2020 | 2020-08-27 | NA |
| Scotland/QEUH-98F9BA/2020 | 2020-08-27 | NA |
| Scotland/QEUH-98F6D1/2020 | 2020-08-27 | NA |
| England/QEUH-998B12/2020 | 2020-08-27 | NA |
| England/MILK-9921E9/2020 | 2020-08-27 | NA |
| England/MILK-9923B6/2020 | 2020-08-27 | NA |
| England/QEUH-997F17/2020 | 2020-08-27 | NA |
| England/QEUH-997EDE/2020 | 2020-08-27 | NA |
| England/QEUH-997F35/2020 | 2020-08-27 | NA |
| England/MILK-993133/2020 | 2020-08-27 | NA |
| England/MILK-993B62/2020 | 2020-08-27 | NA |
| England/MILK-993D5D/2020 | 2020-08-27 | NA |
| England/MILK-993CC9/2020 | 2020-08-27 | NA |
| England/MILK-992A39/2020 | 2020-08-27 | NA |
| Wales/MILK-992820/2020 | 2020-08-27 | NA |
| Wales/MILK-9928A8/2020 | 2020-08-27 | NA |
| England/MILK-991F1D/2020 | 2020-08-27 | NA |
| England/MILK-9924A4/2020 | 2020-08-27 | NA |
| England/MILK-992468/2020 | 2020-08-27 | NA |
| England/QEUH-997F08/2020 | 2020-08-27 | NA |
| England/MILK-99232F/2020 | 2020-08-27 | NA |
| Scotland/QEUH-997F80/2020 | 2020-08-27 | NA |
| England/QEUH-997F44/2020 | 2020-08-27 | NA |
| Wales/MILK-9920FB/2020 | 2020-08-27 | NA |
| Scotland/QEUH-98F57A/2020 | 2020-08-27 | NA |
| England/QEUH-98F589/2020 | 2020-08-27 | NA |
| England/QEUH-99809B/2020 | 2020-08-27 | NA |
| England/MILK-9937C8/2020 | 2020-08-27 | NA |
| England/QEUH-998B9A/2020 | 2020-08-27 | NA |
| England/MILK-993C9C/2020 | 2020-08-27 | NA |
| England/MILK-993CAB/2020 | 2020-08-27 | NA |
| England/QEUH-98EEF8/2020 | 2020-08-28 | NA |
| England/QEUH-98EF04/2020 | 2020-08-28 | NA |
| England/ALDP-9B62FB/2020 | 2020-08-28 | NA |
| England/ALDP-9B62CE/2020 | 2020-08-28 | NA |
| England/ALDP-9B6325/2020 | 2020-08-28 | NA |
| England/ALDP-9B6431/2020 | 2020-08-28 | NA |
| England/ALDP-9B61EF/2020 | 2020-08-28 | NA |
| England/ALDP-9B5A7C/2020 | 2020-08-28 | NA |
| England/ALDP-9B5BD3/2020 | 2020-08-28 | NA |
| England/ALDP-9B5B00/2020 | 2020-08-28 | NA |
| England/MILK-99A61F/2020 | 2020-08-28 | NA |
| Scotland/QEUH-98F03D/2020 | 2020-08-28 | NA |
| England/CAMC-991575/2020 | 2020-08-28 | NA |
| England/QEUH-997BB9/2020 | 2020-08-28 | NA |
| England/ALDP-9B5B5B/2020 | 2020-08-28 | NA |
| England/ALDP-9B645F/2020 | 2020-08-28 | NA |
| Wales/PHWC-169F52/2020 | 2020-08-28 | NA |
| England/ALDP-9B5890/2020 | 2020-08-28 | NA |
| England/QEUH-98EEDA/2020 | 2020-08-28 | NA |
| England/ALDP-9B646E/2020 | 2020-08-28 | NA |
| England/ALDP-9B66D1/2020 | 2020-08-28 | NA |
| England/ALDP-9B5C58/2020 | 2020-08-28 | NA |
| England/QEUH-98F2EC/2020 | 2020-08-28 | NA |
| England/ALDP-9B660E/2020 | 2020-08-28 | NA |
| England/ALDP-9B5793/2020 | 2020-08-28 | NA |
| Scotland/QEUH-98F06A/2020 | 2020-08-28 | NA |
| England/ALDP-9B63F8/2020 | 2020-08-28 | NA |
| United_Arab_Emirates/skmc-3124949/2020 | 2020-08-29 | NA |
| United_Arab_Emirates/skmc-3124740/2020 | 2020-08-29 | NA |
| England/MILK-999C5A/2020 | 2020-08-29 | NA |
| Wales/MILK-999CC3/2020 | 2020-08-29 | NA |
| England/MILK-999D66/2020 | 2020-08-29 | NA |
| England/MILK-999C3C/2020 | 2020-08-29 | NA |
| England/QEUH-9A465E/2020 | 2020-08-29 | NA |
| England/MILK-9AAA98/2020 | 2020-08-29 | NA |
| Wales/MILK-999CF0/2020 | 2020-08-29 | NA |
| Wales/MILK-99A0A8/2020 | 2020-08-29 | NA |
| Scotland/CVR4169/2020 | 2020-08-29 | NA |
| England/MILK-99A5A9/2020 | 2020-08-29 | NA |
| Wales/MILK-999CD2/2020 | 2020-08-29 | NA |
| Wales/MILK-99A679/2020 | 2020-08-29 | NA |
| Wales/MILK-99A697/2020 | 2020-08-29 | NA |
| England/ALDP-9B5DFB/2020 | 2020-08-29 | NA |
| England/MILK-99A8CE/2020 | 2020-08-29 | NA |
| England/QEUH-99050D/2020 | 2020-08-29 | NA |
| England/MILK-99A372/2020 | 2020-08-29 | NA |
| England/MILK-99A882/2020 | 2020-08-29 | NA |
| England/QEUH-99027F/2020 | 2020-08-29 | NA |
| England/MILK-999D84/2020 | 2020-08-29 | NA |
| England/MILK-99A04E/2020 | 2020-08-29 | NA |
| England/MILK-999D57/2020 | 2020-08-29 | NA |
| England/QEUH-98FB96/2020 | 2020-08-29 | NA |
| England/QEUH-9968A4/2020 | 2020-08-29 | NA |
| England/MILK-99A5B8/2020 | 2020-08-29 | NA |
| England/MILK-99A7A3/2020 | 2020-08-29 | NA |
| Wales/MILK-99368F/2020 | 2020-08-29 | NA |
| England/MILK-99A9F8/2020 | 2020-08-29 | NA |
| England/MILK-99A600/2020 | 2020-08-29 | NA |
| England/MILK-99A688/2020 | 2020-08-29 | NA |
| England/MILK-99CD36/2020 | 2020-08-30 | NA |
| Scotland/CVR4062/2020 | 2020-08-30 | NA |
| England/MILK-99D342/2020 | 2020-08-30 | NA |
| England/MILK-99DAA7/2020 | 2020-08-30 | NA |
| England/MILK-99DFF3/2020 | 2020-08-30 | NA |
| England/MILK-99CE24/2020 | 2020-08-30 | NA |
| England/QEUH-9AF2E6/2020 | 2020-08-30 | NA |
| England/QEUH-99BE16/2020 | 2020-08-30 | NA |
| England/QEUH-99B14A/2020 | 2020-08-30 | NA |
| Scotland/QEUH-99BF5F/2020 | 2020-08-30 | NA |
| England/MILK-99CFF4/2020 | 2020-08-30 | NA |
| England/MILK-99D00F/2020 | 2020-08-30 | NA |
| England/MILK-99CDCD/2020 | 2020-08-30 | NA |
| England/MILK-99A15A/2020 | 2020-08-30 | NA |
| Scotland/QEUH-990190/2020 | 2020-08-30 | NA |
| England/MILK-99E077/2020 | 2020-08-30 | NA |
| England/MILK-99E10B/2020 | 2020-08-30 | NA |
| England/MILK-99E068/2020 | 2020-08-30 | NA |
| Wales/MILK-99DE8D/2020 | 2020-08-30 | NA |
| England/MILK-99DA3E/2020 | 2020-08-30 | NA |
| England/MILK-99C64A/2020 | 2020-08-30 | NA |
| Wales/PHWC-169EBF/2020 | 2020-08-30 | NA |
| England/MILK-99C7ED/2020 | 2020-08-30 | NA |
| Wales/MILK-9AA6B2/2020 | 2020-08-30 | NA |
| Wales/MILK-9A8E43/2020 | 2020-08-30 | NA |
| Scotland/CVR4162/2020 | 2020-08-30 | NA |
| Wales/MILK-99C5B6/2020 | 2020-08-30 | NA |
| England/MILK-99A0E4/2020 | 2020-08-30 | NA |
| England/MILK-99DA2F/2020 | 2020-08-30 | NA |
| England/MILK-99D8BC/2020 | 2020-08-30 | NA |
| Wales/MILK-99C53E/2020 | 2020-08-30 | NA |
| Wales/MILK-99C54D/2020 | 2020-08-30 | NA |
| Wales/MILK-99C52F/2020 | 2020-08-30 | NA |
| Wales/MILK-99DA6B/2020 | 2020-08-30 | NA |
| Wales/MILK-9AA6C1/2020 | 2020-08-30 | NA |
| England/MILK-99D7DD/2020 | 2020-08-30 | NA |
| England/MILK-99C6B3/2020 | 2020-08-30 | NA |
| England/MILK-99D931/2020 | 2020-08-30 | NA |
| Wales/MILK-99DD9F/2020 | 2020-08-30 | NA |
| Wales/MILK-99E156/2020 | 2020-08-30 | NA |
| England/MILK-99DBFF/2020 | 2020-08-30 | NA |
| Scotland/CVR4048/2020 | 2020-08-30 | NA |
| England/MILK-99A0C6/2020 | 2020-08-30 | NA |
| England/MILK-99D272/2020 | 2020-08-30 | NA |
| England/MILK-99DB0E/2020 | 2020-08-30 | NA |
| England/MILK-99DC83/2020 | 2020-08-30 | NA |
| Scotland/QEUH-99BF6E/2020 | 2020-08-30 | NA |
| England/MILK-99E45D/2020 | 2020-08-30 | NA |
| England/MILK-99CD27/2020 | 2020-08-30 | NA |
| England/MILK-99CCDF/2020 | 2020-08-30 | NA |
| England/MILK-99D209/2020 | 2020-08-30 | NA |
| England/MILK-99CBE1/2020 | 2020-08-30 | NA |
| England/MILK-99E44E/2020 | 2020-08-30 | NA |
| England/MILK-99A0F3/2020 | 2020-08-30 | NA |
| England/MILK-9AAB1D/2020 | 2020-08-31 | NA |
| England/MILK-9AB4D5/2020 | 2020-08-31 | NA |
| Scotland/CVR4083/2020 | 2020-08-31 | NA |
| England/MILK-9AB314/2020 | 2020-08-31 | NA |
| England/MILK-9AAF99/2020 | 2020-08-31 | NA |
| England/MILK-9AAF8A/2020 | 2020-08-31 | NA |
| England/MILK-9AB675/2020 | 2020-08-31 | NA |
| England/MILK-9AB03B/2020 | 2020-08-31 | NA |
| England/MILK-9A8757/2020 | 2020-08-31 | NA |
| England/MILK-9AB709/2020 | 2020-08-31 | NA |
| England/MILK-99D48B/2020 | 2020-08-31 | NA |
| England/MILK-99DCCF/2020 | 2020-08-31 | NA |
| England/MILK-99C89F/2020 | 2020-08-31 | NA |
| England/MILK-99CB4B/2020 | 2020-08-31 | NA |
| England/MILK-99C905/2020 | 2020-08-31 | NA |
| England/MILK-99E39C/2020 | 2020-08-31 | NA |
| England/MILK-99E0FF/2020 | 2020-08-31 | NA |
| England/MILK-99E350/2020 | 2020-08-31 | NA |
| Wales/MILK-9AAD62/2020 | 2020-08-31 | NA |
| Wales/MILK-9A9D90/2020 | 2020-08-31 | NA |
| England/MILK-9AB4C6/2020 | 2020-08-31 | NA |
| England/MILK-9AB4E4/2020 | 2020-08-31 | NA |
| England/MILK-99E587/2020 | 2020-08-31 | NA |
| England/MILK-9A9BA5/2020 | 2020-08-31 | NA |
| England/MILK-9A9176/2020 | 2020-08-31 | NA |
| Wales/MILK-99CA7B/2020 | 2020-08-31 | NA |
| England/MILK-99DDCC/2020 | 2020-08-31 | NA |
| England/MILK-99CC48/2020 | 2020-08-31 | NA |
| England/MILK-99E323/2020 | 2020-08-31 | NA |
| Scotland/CVR4205/2020 | 2020-08-31 | NA |
| England/MILK-9A9914/2020 | 2020-08-31 | NA |
| England/MILK-9AA7A0/2020 | 2020-08-31 | NA |
| England/MILK-9AB29F/2020 | 2020-08-31 | NA |
| England/MILK-9AB4B7/2020 | 2020-08-31 | NA |
| England/MILK-9A912B/2020 | 2020-08-31 | NA |
| England/MILK-9AB48A/2020 | 2020-08-31 | NA |
| England/MILK-9A8C67/2020 | 2020-08-31 | NA |
| England/MILK-99DF6C/2020 | 2020-08-31 | NA |
| Scotland/CVR4075/2020 | 2020-08-31 | NA |
| England/MILK-9AB6DF/2020 | 2020-08-31 | NA |
| England/MILK-9AB4A8/2020 | 2020-08-31 | NA |
| England/MILK-99E36F/2020 | 2020-08-31 | NA |
| England/MILK-9AA0A5/2020 | 2020-08-31 | NA |
| England/MILK-9AB341/2020 | 2020-08-31 | NA |
| England/MILK-9AB3AB/2020 | 2020-08-31 | NA |
| England/MILK-9A903D/2020 | 2020-08-31 | NA |
| England/MILK-99DC92/2020 | 2020-08-31 | NA |
| England/MILK-9AB6C0/2020 | 2020-08-31 | NA |
| England/MILK-99E341/2020 | 2020-08-31 | NA |
| Wales/MILK-9A970B/2020 | 2020-09-01 | NA |
| England/MILK-9AA3F7/2020 | 2020-09-01 | NA |
| England/MILK-9A69BD/2020 | 2020-09-01 | NA |
| England/MILK-9AA45E/2020 | 2020-09-01 | NA |
| England/QEUH-9A41E4/2020 | 2020-09-01 | NA |
| England/MILK-9A9AF3/2020 | 2020-09-01 | NA |
| England/MILK-9AA281/2020 | 2020-09-01 | NA |
| England/QEUH-9AE5FD/2020 | 2020-09-01 | NA |
| England/MILK-9A6953/2020 | 2020-09-01 | NA |
| England/MILK-9A66A7/2020 | 2020-09-01 | NA |
| England/QEUH-9A3FDC/2020 | 2020-09-01 | NA |
| Wales/MILK-9A9255/2020 | 2020-09-01 | NA |
| England/QEUH-9A44FA/2020 | 2020-09-01 | NA |
| Wales/MILK-9A8E70/2020 | 2020-09-01 | NA |
| England/MILK-9A899D/2020 | 2020-09-01 | NA |
| Wales/MILK-9A9C1B/2020 | 2020-09-01 | NA |
| England/MILK-9AACA1/2020 | 2020-09-01 | NA |
| Wales/MILK-9AADCC/2020 | 2020-09-01 | NA |
| Wales/PHWC-16A15B/2020 | 2020-09-01 | NA |
| Wales/MILK-9A6908/2020 | 2020-09-01 | NA |
| England/MILK-9AA272/2020 | 2020-09-01 | NA |
| England/MILK-9A9A3F/2020 | 2020-09-01 | NA |
| England/MILK-9A9C48/2020 | 2020-09-01 | NA |
| England/MILK-9A8F6E/2020 | 2020-09-01 | NA |
| England/MILK-9A8EF8/2020 | 2020-09-01 | NA |
| Wales/MILK-9A94AA/2020 | 2020-09-01 | NA |
| England/MILK-9A9E24/2020 | 2020-09-01 | NA |
| England/MILK-9AA087/2020 | 2020-09-01 | NA |
| England/MILK-9A9EE8/2020 | 2020-09-01 | NA |
| England/QEUH-9A4120/2020 | 2020-09-01 | NA |
| England/QEUH-9A4612/2020 | 2020-09-01 | NA |
| England/QEUH-9A406F/2020 | 2020-09-01 | NA |
| Wales/QEUH-9AE00B/2020 | 2020-09-01 | NA |
| England/MILK-9A956B/2020 | 2020-09-01 | NA |
| England/MILK-9A6BF3/2020 | 2020-09-02 | NA |
| England/MILK-9A855D/2020 | 2020-09-02 | NA |
| England/MILK-9A6A9C/2020 | 2020-09-02 | NA |
| England/MILK-9A6EEB/2020 | 2020-09-02 | NA |
| England/MILK-9A6EBE/2020 | 2020-09-02 | NA |
| England/MILK-9A6B20/2020 | 2020-09-02 | NA |
| England/MILK-9A507D/2020 | 2020-09-02 | NA |
| England/MILK-9A846F/2020 | 2020-09-02 | NA |
| England/MILK-9A8669/2020 | 2020-09-02 | NA |
| England/MILK-9A8DA0/2020 | 2020-09-02 | NA |
| England/MILK-9A964A/2020 | 2020-09-02 | NA |
| England/MILK-9A7FD8/2020 | 2020-09-02 | NA |
| England/QEUH-9B4828/2020 | 2020-09-02 | NA |
| England/MILK-9A9291/2020 | 2020-09-02 | NA |
| Wales/QEUH-9AE065/2020 | 2020-09-02 | NA |
| England/QEUH-9AE7AC/2020 | 2020-09-02 | NA |
| England/MILK-9A51D4/2020 | 2020-09-02 | NA |
| England/MILK-9A6A05/2020 | 2020-09-02 | NA |
| England/MILK-9A6EFA/2020 | 2020-09-02 | NA |
| England/QEUH-9ACC72/2020 | 2020-09-02 | NA |
| United_Arab_Emirates/skmc-3358771/2020 | 2020-09-03 | NA |
| England/QEUH-9B48BF/2020 | 2020-09-03 | NA |
| England/QEUH-9AEBEC/2020 | 2020-09-03 | NA |
| England/QEUH-9AEC07/2020 | 2020-09-03 | NA |
| England/QEUH-9B4864/2020 | 2020-09-03 | NA |
| England/QEUH-9B5362/2020 | 2020-09-03 | NA |
| England/QEUH-9B49DA/2020 | 2020-09-03 | NA |
| Wales/QEUH-9B49BC/2020 | 2020-09-03 | NA |
| England/QEUH-9B5405/2020 | 2020-09-03 | NA |
| England/QEUH-9B81A1/2020 | 2020-09-03 | NA |
| Scotland/QEUH-9AF0EC/2020 | 2020-09-03 | NA |
| England/QEUH-9B0F36/2020 | 2020-09-03 | NA |
| England/QEUH-9B0C5D/2020 | 2020-09-03 | NA |
| England/QEUH-9B7D08/2020 | 2020-09-03 | NA |
| Wales/MILK-9A6CC3/2020 | 2020-09-03 | NA |
| England/QEUH-9AEDB9/2020 | 2020-09-03 | NA |
| England/QEUH-9AF28C/2020 | 2020-09-03 | NA |
| England/QEUH-9AEF68/2020 | 2020-09-03 | NA |
| England/QEUH-9AEF95/2020 | 2020-09-03 | NA |
| England/QEUH-9C81CC/2020 | 2020-09-03 | NA |
| England/QEUH-9AED22/2020 | 2020-09-03 | NA |
| England/QEUH-9AEDAA/2020 | 2020-09-03 | NA |
| England/QEUH-9AEE2F/2020 | 2020-09-03 | NA |
| England/QEUH-9AEE01/2020 | 2020-09-03 | NA |
| England/QEUH-9AEE3E/2020 | 2020-09-03 | NA |
| England/QEUH-9AECE9/2020 | 2020-09-03 | NA |
| England/QEUH-9AED04/2020 | 2020-09-03 | NA |
| England/QEUH-9AECF8/2020 | 2020-09-03 | NA |
| England/QEUH-9AEC70/2020 | 2020-09-03 | NA |
| England/QEUH-9AEFB3/2020 | 2020-09-03 | NA |
| England/QEUH-9AEFD1/2020 | 2020-09-03 | NA |
| England/QEUH-9AEB28/2020 | 2020-09-03 | NA |
| England/QEUH-9AEB46/2020 | 2020-09-03 | NA |
| England/QEUH-9AEB73/2020 | 2020-09-03 | NA |
| England/QEUH-9AEBA0/2020 | 2020-09-03 | NA |
| England/QEUH-9C8180/2020 | 2020-09-03 | NA |
| England/QEUH-9C81AE/2020 | 2020-09-03 | NA |
| England/QEUH-9AEDD7/2020 | 2020-09-03 | NA |
| England/QEUH-9AEDC8/2020 | 2020-09-03 | NA |
| England/QEUH-9AEE10/2020 | 2020-09-03 | NA |
| England/QEUH-9AED9B/2020 | 2020-09-03 | NA |
| England/QEUH-9AEB64/2020 | 2020-09-03 | NA |
| England/QEUH-9AED40/2020 | 2020-09-03 | NA |
| England/QEUH-9AEF77/2020 | 2020-09-03 | NA |
| England/QEUH-9AF29B/2020 | 2020-09-03 | NA |
| England/QEUH-9B0D5A/2020 | 2020-09-03 | NA |
| England/QEUH-9B0D0F/2020 | 2020-09-03 | NA |
| England/QEUH-9B0F63/2020 | 2020-09-03 | NA |
| England/MILK-9A5541/2020 | 2020-09-03 | NA |
| England/QEUH-9B0C7B/2020 | 2020-09-03 | NA |
| England/QEUH-9B3D57/2020 | 2020-09-03 | NA |
| Wales/PHWC-16A1E2/2020 | 2020-09-03 | NA |
| England/QEUH-9B4891/2020 | 2020-09-03 | NA |
| England/QEUH-9B0E39/2020 | 2020-09-03 | NA |
| England/MILK-9A6DC0/2020 | 2020-09-03 | NA |
| Wales/PHWC-16D52F/2020 | 2020-09-03 | NA |
| England/QEUH-9B49CB/2020 | 2020-09-03 | NA |
| Wales/PHWC-16A4BC/2020 | 2020-09-03 | NA |
| England/QEUH-9B4916/2020 | 2020-09-03 | NA |
| England/QEUH-9B49F8/2020 | 2020-09-03 | NA |
| England/QEUH-9B0F54/2020 | 2020-09-03 | NA |
| England/QEUH-9B0DF0/2020 | 2020-09-03 | NA |
| England/QEUH-9AEE4D/2020 | 2020-09-03 | NA |
| England/QEUH-9AEB37/2020 | 2020-09-03 | NA |
| England/QEUH-9AF222/2020 | 2020-09-03 | NA |
| Scotland/QEUH-9B0C20/2020 | 2020-09-03 | NA |
| Wales/MILK-9A53DE/2020 | 2020-09-03 | NA |
| England/MILK-9A53FC/2020 | 2020-09-03 | NA |
| Wales/MILK-9A53ED/2020 | 2020-09-03 | NA |
| Northern_Ireland/QEUH-9B46D3/2020 | 2020-09-04 | NA |
| England/QEUH-9B4AB9/2020 | 2020-09-04 | NA |
| England/QEUH-9B7A3E/2020 | 2020-09-04 | NA |
| England/QEUH-9AE1BD/2020 | 2020-09-04 | NA |
| England/CAMC-9B25C9/2020 | 2020-09-04 | NA |
| England/QEUH-9AF76F/2020 | 2020-09-04 | NA |
| England/QEUH-9B7AF2/2020 | 2020-09-04 | NA |
| England/CAMC-9B1ADA/2020 | 2020-09-04 | NA |
| England/CAMC-9B1AE9/2020 | 2020-09-04 | NA |
| England/QEUH-9B7B77/2020 | 2020-09-04 | NA |
| England/QEUH-9B3F06/2020 | 2020-09-04 | NA |
| England/CAMC-9B2462/2020 | 2020-09-04 | NA |
| Wales/PHWC-16A2EF/2020 | 2020-09-04 | NA |
| England/CAMC-9B2EEC/2020 | 2020-09-04 | NA |
| England/QEUH-9B4E62/2020 | 2020-09-04 | NA |
| England/CAMC-9B1DB3/2020 | 2020-09-04 | NA |
| England/QEUH-9B46F1/2020 | 2020-09-04 | NA |
| England/QEUH-9AE1DB/2020 | 2020-09-04 | NA |
| England/CAMC-9B2004/2020 | 2020-09-04 | NA |
| England/CAMC-9B1C3E/2020 | 2020-09-04 | NA |
| England/QEUH-9B3B4E/2020 | 2020-09-04 | NA |
| Wales/PHWC-16A012/2020 | 2020-09-05 | NA |
| England/QEUH-9B87EB/2020 | 2020-09-05 | NA |
| England/CAMC-9B2839/2020 | 2020-09-05 | NA |
| England/QEUH-9AE9B5/2020 | 2020-09-05 | NA |
| Scotland/QEUH-9AD075/2020 | 2020-09-05 | NA |
| Scotland/QEUH-9ACF2E/2020 | 2020-09-05 | NA |
| Scotland/QEUH-9ACF97/2020 | 2020-09-05 | NA |
| England/QEUH-9ACB93/2020 | 2020-09-05 | NA |
| Wales/PHWC-16A05E/2020 | 2020-09-05 | NA |
| England/QEUH-9AE0B0/2020 | 2020-09-05 | NA |
| England/QEUH-9AE0CF/2020 | 2020-09-05 | NA |
| England/QEUH-9AE4F0/2020 | 2020-09-05 | NA |
| England/QEUH-9ACFC4/2020 | 2020-09-05 | NA |
| England/QEUH-9AEA2B/2020 | 2020-09-05 | NA |
| England/QEUH-9AE0A1/2020 | 2020-09-05 | NA |
| England/QEUH-9AEA3A/2020 | 2020-09-05 | NA |
| England/QEUH-9AE9A6/2020 | 2020-09-05 | NA |
| England/QEUH-9AEAA3/2020 | 2020-09-05 | NA |
| England/QEUH-9AD224/2020 | 2020-09-05 | NA |
| England/QEUH-9AD27F/2020 | 2020-09-05 | NA |
| England/QEUH-9ACFA6/2020 | 2020-09-05 | NA |
| England/QEUH-9AE539/2020 | 2020-09-05 | NA |
| England/QEUH-9AD1BE/2020 | 2020-09-05 | NA |
| England/QEUH-9ACBEE/2020 | 2020-09-05 | NA |
| England/QEUH-9AE51B/2020 | 2020-09-05 | NA |
| England/QEUH-9AEA85/2020 | 2020-09-05 | NA |
| Wales/QEUH-9B9E4F/2020 | 2020-09-05 | NA |
| Scotland/QEUH-9ADF5A/2020 | 2020-09-05 | NA |
| Scotland/QEUH-9ADF87/2020 | 2020-09-05 | NA |
| Wales/PHWC-16A3FB/2020 | 2020-09-05 | NA |
| Scotland/CVR4001/2020 | 2020-09-05 | NA |
| England/QEUH-9AE6AF/2020 | 2020-09-05 | NA |
| England/SHEF-CC5B6/2020 | 2020-09-06 | NA |
| Wales/PHWC-16A285/2020 | 2020-09-06 | NA |
| Wales/PHWC-4833AE/2020 | 2020-09-06 | NA |
| England/CAMC-9C0258/2020 | 2020-09-06 | NA |
| Wales/PHWC-16A522/2020 | 2020-09-06 | NA |
| England/QEUH-9BA37C/2020 | 2020-09-06 | NA |
| England/QEUH-9B7272/2020 | 2020-09-06 | NA |
| Scotland/QEUH-9B5089/2020 | 2020-09-07 | NA |
| England/QEUH-9B74D6/2020 | 2020-09-07 | NA |
| England/QEUH-9B9331/2020 | 2020-09-07 | NA |
| England/QEUH-9B9FD3/2020 | 2020-09-07 | NA |
| England/QEUH-9B8F10/2020 | 2020-09-07 | NA |
| England/QEUH-9BA109/2020 | 2020-09-07 | NA |
| Scotland/QEUH-9B4CE0/2020 | 2020-09-07 | NA |
| England/QEUH-9B49AD/2020 | 2020-09-07 | NA |
| England/QEUH-9B7B59/2020 | 2020-09-07 | NA |
| England/QEUH-9B744F/2020 | 2020-09-07 | NA |
| England/QEUH-9B7E23/2020 | 2020-09-07 | NA |
| England/QEUH-9B9137/2020 | 2020-09-07 | NA |
| England/QEUH-9B8FF2/2020 | 2020-09-07 | NA |
| England/QEUH-9B8AF1/2020 | 2020-09-07 | NA |
| England/QEUH-9BA2F7/2020 | 2020-09-07 | NA |
| England/QEUH-9B4FF6/2020 | 2020-09-07 | NA |
| England/QEUH-9B4F50/2020 | 2020-09-07 | NA |
| England/CAMC-9C03DD/2020 | 2020-09-07 | NA |
| England/QEUH-9B4F41/2020 | 2020-09-07 | NA |
| England/MILK-9C4676/2020 | 2020-09-07 | NA |
| England/QEUH-9B50E3/2020 | 2020-09-07 | NA |
| England/QEUH-9B9CAF/2020 | 2020-09-07 | NA |
| England/QEUH-9BA172/2020 | 2020-09-07 | NA |
| England/MILK-9C258A/2020 | 2020-09-07 | NA |
| England/MILK-9C3774/2020 | 2020-09-07 | NA |
| England/QEUH-9BB0CF/2020 | 2020-09-07 | NA |
| England/QEUH-9B9E8B/2020 | 2020-09-07 | NA |
| England/QEUH-9BA0DF/2020 | 2020-09-07 | NA |
| England/SHEF-CF29E/2020 | 2020-09-07 | NA |
| England/QEUH-9B8E22/2020 | 2020-09-07 | NA |
| Scotland/CVR4122/2020 | 2020-09-07 | NA |
| England/QEUH-9B4D74/2020 | 2020-09-07 | NA |
| England/QEUH-9B8FE3/2020 | 2020-09-07 | NA |
| England/MILK-9C3A3F/2020 | 2020-09-07 | NA |
| England/QEUH-9B9F00/2020 | 2020-09-07 | NA |
| England/PORT-2D4504/2020 | 2020-09-07 | NA |
| England/PORT-2D45D7/2020 | 2020-09-07 | NA |
| England/QEUH-9B50C5/2020 | 2020-09-07 | NA |
| Wales/PHWC-16A57D/2020 | 2020-09-07 | NA |
| England/QEUH-9BB092/2020 | 2020-09-07 | NA |
| England/QEUH-9BAC08/2020 | 2020-09-07 | NA |
| England/QEUH-9B7685/2020 | 2020-09-08 | NA |
| England/MILK-9C2CB2/2020 | 2020-09-08 | NA |
| Scotland/MILK-9C3817/2020 | 2020-09-08 | NA |
| England/CAMC-9C0452/2020 | 2020-09-08 | NA |
| England/QEUH-9C8654/2020 | 2020-09-08 | NA |
| England/QEUH-9B78DA/2020 | 2020-09-08 | NA |
| England/QEUH-9B7649/2020 | 2020-09-08 | NA |
| England/QEUH-9B78BC/2020 | 2020-09-08 | NA |
| Scotland/QEUH-9B4521/2020 | 2020-09-08 | NA |
| Wales/PHWC-16AE27/2020 | 2020-09-08 | NA |
| England/QEUH-9B829F/2020 | 2020-09-08 | NA |
| England/QEUH-9B3ABA/2020 | 2020-09-08 | NA |
| England/QEUH-9B79C8/2020 | 2020-09-08 | NA |
| England/QEUH-9B3C2D/2020 | 2020-09-08 | NA |
| England/QEUH-9B3B99/2020 | 2020-09-08 | NA |
| England/QEUH-9B77A0/2020 | 2020-09-08 | NA |
| England/CAMC-9C17FE/2020 | 2020-09-08 | NA |
| England/QEUH-9B78E9/2020 | 2020-09-08 | NA |
| England/QEUH-9B7870/2020 | 2020-09-08 | NA |
| England/CAMC-9C00E5/2020 | 2020-09-08 | NA |
| England/ALDP-9BCBBE/2020 | 2020-09-08 | NA |
| England/QEUH-9BA8B9/2020 | 2020-09-08 | NA |
| England/QEUH-9B79AA/2020 | 2020-09-08 | NA |
| England/QEUH-9B7FC6/2020 | 2020-09-08 | NA |
| England/QEUH-9B795F/2020 | 2020-09-08 | NA |
| England/QEUH-9B3EAF/2020 | 2020-09-08 | NA |
| England/QEUH-9B789E/2020 | 2020-09-08 | NA |
| England/QEUH-9B3C87/2020 | 2020-09-08 | NA |
| England/QEUH-9B788F/2020 | 2020-09-08 | NA |
| England/QEUH-9B8FC5/2020 | 2020-09-08 | NA |
| England/MILK-9C28DC/2020 | 2020-09-08 | NA |
| England/MILK-9C26E1/2020 | 2020-09-08 | NA |
| England/MILK-9C739A/2020 | 2020-09-08 | NA |
| England/MILK-9C70EE/2020 | 2020-09-08 | NA |
| England/MILK-9C7084/2020 | 2020-09-08 | NA |
| England/MILK-9C736D/2020 | 2020-09-08 | NA |
| England/QEUH-9B3C3C/2020 | 2020-09-08 | NA |
| England/CAMC-9C1CFF/2020 | 2020-09-08 | NA |
| England/QEUH-9B7719/2020 | 2020-09-08 | NA |
| England/QEUH-9B3C4B/2020 | 2020-09-08 | NA |
| England/PORT-2D45B9/2020 | 2020-09-08 | NA |
| England/PORT-2D44CB/2020 | 2020-09-08 | NA |
| England/QEUH-9B8CCE/2020 | 2020-09-08 | NA |
| England/MILK-9C3F6D/2020 | 2020-09-09 | NA |
| England/CAMC-9C048F/2020 | 2020-09-09 | NA |
| England/QEUH-9C96DB/2020 | 2020-09-09 | NA |
| England/MILK-9C3DEB/2020 | 2020-09-09 | NA |
| Wales/MILK-9C255D/2020 | 2020-09-09 | NA |
| Wales/MILK-9C3AB7/2020 | 2020-09-09 | NA |
| England/MILK-9C45A6/2020 | 2020-09-09 | NA |
| England/CAMC-9BFF0A/2020 | 2020-09-09 | NA |
| Scotland/QEUH-9BADD8/2020 | 2020-09-09 | NA |
| Scotland/QEUH-9BA5EF/2020 | 2020-09-09 | NA |
| England/QEUH-9BA576/2020 | 2020-09-09 | NA |
| England/CAMC-9C07FF/2020 | 2020-09-09 | NA |
| England/ALDP-9BC84E/2020 | 2020-09-09 | NA |
| England/ALDP-9BC811/2020 | 2020-09-09 | NA |
| England/ALDP-9BC7E7/2020 | 2020-09-09 | NA |
| England/QEUH-9B9AC3/2020 | 2020-09-09 | NA |
| England/QEUH-9B9BC0/2020 | 2020-09-09 | NA |
| Scotland/QEUH-9B9AA5/2020 | 2020-09-09 | NA |
| Wales/PHWC-16A67A/2020 | 2020-09-09 | NA |
| England/QEUH-9B9B48/2020 | 2020-09-09 | NA |
| England/QEUH-9B9D33/2020 | 2020-09-09 | NA |
| England/QEUH-9BB311/2020 | 2020-09-09 | NA |
| England/QEUH-9BB1AE/2020 | 2020-09-09 | NA |
| England/ALDP-9BCC24/2020 | 2020-09-09 | NA |
| England/QEUH-9B8F7A/2020 | 2020-09-09 | NA |
| England/QEUH-9BB38A/2020 | 2020-09-09 | NA |
| England/QEUH-9BB2D8/2020 | 2020-09-09 | NA |
| England/QEUH-9BB2E7/2020 | 2020-09-09 | NA |
| England/QEUH-9B8C91/2020 | 2020-09-09 | NA |
| England/QEUH-9BB135/2020 | 2020-09-09 | NA |
| England/QEUH-9BA7AD/2020 | 2020-09-09 | NA |
| England/QEUH-9B6ED9/2020 | 2020-09-09 | NA |
| England/QEUH-9BB3A8/2020 | 2020-09-09 | NA |
| England/QEUH-9BB399/2020 | 2020-09-09 | NA |
| England/CAMC-9C0540/2020 | 2020-09-09 | NA |
| England/CAMC-9C09EA/2020 | 2020-09-09 | NA |
| England/MILK-9C3CB1/2020 | 2020-09-09 | NA |
| Wales/MILK-9C2335/2020 | 2020-09-09 | NA |
| England/MILK-9C239F/2020 | 2020-09-09 | NA |
| England/MILK-9C2380/2020 | 2020-09-09 | NA |
| England/ALDP-9BE7F4/2020 | 2020-09-09 | NA |
| England/ALDP-9BE81F/2020 | 2020-09-09 | NA |
| England/CAMC-9C1CE0/2020 | 2020-09-09 | NA |
| England/ALDP-9BE25D/2020 | 2020-09-09 | NA |
| Scotland/CVR4120/2020 | 2020-09-09 | NA |
| Wales/PHWC-16A926/2020 | 2020-09-09 | NA |
| England/MILK-9C3EAC/2020 | 2020-09-09 | NA |
| England/QEUH-9BB126/2020 | 2020-09-09 | NA |
| England/QEUH-9BB232/2020 | 2020-09-09 | NA |
| England/ALDP-9BDCAB/2020 | 2020-09-09 | NA |
| England/QEUH-9BA479/2020 | 2020-09-09 | NA |
| Scotland/QEUH-9BA43D/2020 | 2020-09-09 | NA |
| Scotland/QEUH-9B8305/2020 | 2020-09-09 | NA |
| Scotland/QEUH-9BA45B/2020 | 2020-09-09 | NA |
| England/QEUH-9BA79E/2020 | 2020-09-09 | NA |
| England/QEUH-9B837E/2020 | 2020-09-09 | NA |
| England/QEUH-9BB171/2020 | 2020-09-09 | NA |
| England/CAMC-9C0425/2020 | 2020-09-09 | NA |
| England/QEUH-9BA770/2020 | 2020-09-09 | NA |
| England/NOTT-113616/2020 | 2020-09-09 | NA |
| England/QEUH-9B701E/2020 | 2020-09-09 | NA |
| England/QEUH-9B702D/2020 | 2020-09-09 | NA |
| England/ALDP-9BC7D8/2020 | 2020-09-09 | NA |
| England/ALDP-9BED3E/2020 | 2020-09-09 | NA |
| England/CAMC-9C1AD7/2020 | 2020-09-10 | NA |
| England/ALDP-9C55A5/2020 | 2020-09-10 | NA |
| England/ALDP-9C4E9C/2020 | 2020-09-10 | NA |
| England/ALDP-9BE2E4/2020 | 2020-09-10 | NA |
| Wales/MILK-9C35F2/2020 | 2020-09-10 | NA |
| Scotland/QEUH-9B8657/2020 | 2020-09-10 | NA |
| England/ALDP-9C4EF6/2020 | 2020-09-10 | NA |
| England/CAMC-9C1A04/2020 | 2020-09-10 | NA |
| England/QEUH-9C7DD8/2020 | 2020-09-10 | NA |
| Wales/QEUH-9C98D5/2020 | 2020-09-10 | NA |
| England/QEUH-9C9B27/2020 | 2020-09-10 | NA |
| England/QEUH-9CA722/2020 | 2020-09-10 | NA |
| England/QEUH-9C933E/2020 | 2020-09-10 | NA |
| England/QEUH-9CA713/2020 | 2020-09-10 | NA |
| England/ALDP-9C4E50/2020 | 2020-09-10 | NA |
| England/ALDP-9C6182/2020 | 2020-09-10 | NA |
| England/ALDP-9C61BF/2020 | 2020-09-10 | NA |
| England/ALDP-9BEAA0/2020 | 2020-09-10 | NA |
| Scotland/QEUH-9B8736/2020 | 2020-09-10 | NA |
| Wales/PHWC-16A7C2/2020 | 2020-09-10 | NA |
| Wales/PHWC-16A698/2020 | 2020-09-10 | NA |
| England/ALDP-9C5587/2020 | 2020-09-10 | NA |
| England/QEUH-9CA8A7/2020 | 2020-09-10 | NA |
| England/ALDP-9C54F3/2020 | 2020-09-10 | NA |
| Scotland/QEUH-9B82BD/2020 | 2020-09-10 | NA |
| England/QEUH-9C9E88/2020 | 2020-09-10 | NA |
| England/QEUH-9C934D/2020 | 2020-09-10 | NA |
| England/QEUH-9CA573/2020 | 2020-09-10 | NA |
| England/ALDP-9C550F/2020 | 2020-09-10 | NA |
| England/QEUH-9B85A5/2020 | 2020-09-10 | NA |
| England/CAMC-9C19E9/2020 | 2020-09-10 | NA |
| England/QEUH-9CA5BF/2020 | 2020-09-10 | NA |
| England/QEUH-9C7AD1/2020 | 2020-09-10 | NA |
| England/ALDP-9C5B94/2020 | 2020-09-10 | NA |
| England/MILK-9C3246/2020 | 2020-09-10 | NA |
| England/MILK-9C41A2/2020 | 2020-09-10 | NA |
| England/ALDP-9C5C91/2020 | 2020-09-10 | NA |
| England/QEUH-9C8C8F/2020 | 2020-09-10 | NA |
| England/NOTT-113115/2020 | 2020-09-10 | NA |
| England/QEUH-9C984E/2020 | 2020-09-10 | NA |
| England/QEUH-9C93E3/2020 | 2020-09-10 | NA |
| England/QEUH-9CA3D3/2020 | 2020-09-10 | NA |
| England/QEUH-9C9468/2020 | 2020-09-10 | NA |
| England/ALDP-9C6207/2020 | 2020-09-10 | NA |
| England/ALDP-9C61CE/2020 | 2020-09-10 | NA |
| England/QEUH-9C80B0/2020 | 2020-09-10 | NA |
| Wales/PHWC-16B099/2020 | 2020-09-10 | NA |
| England/MILK-9C4AD4/2020 | 2020-09-10 | NA |
| England/ALDP-9C54B7/2020 | 2020-09-10 | NA |
| England/ALDP-9C5C37/2020 | 2020-09-10 | NA |
| England/ALDP-9C5F7A/2020 | 2020-09-10 | NA |
| England/QEUH-9C9E00/2020 | 2020-09-10 | NA |
| England/QEUH-9CA986/2020 | 2020-09-10 | NA |
| England/QEUH-9C9705/2020 | 2020-09-10 | NA |
| England/QEUH-9C9C15/2020 | 2020-09-10 | NA |
| England/ALDP-9BE9EF/2020 | 2020-09-10 | NA |
| England/ALDP-9BE9D0/2020 | 2020-09-10 | NA |
| England/ALDP-9BEA19/2020 | 2020-09-10 | NA |
| England/NOTT-1136AD/2020 | 2020-09-10 | NA |
| England/QEUH-9C9DE5/2020 | 2020-09-10 | NA |
| England/QEUH-9CA0AF/2020 | 2020-09-10 | NA |
| England/QEUH-9CA94A/2020 | 2020-09-10 | NA |
| England/QEUH-9CA090/2020 | 2020-09-10 | NA |
| Wales/PHWC-16AC2D/2020 | 2020-09-10 | NA |
| Wales/PHWC-16ABA8/2020 | 2020-09-10 | NA |
| England/CAMC-9C1D29/2020 | 2020-09-10 | NA |
| England/QEUH-9CA063/2020 | 2020-09-10 | NA |
| England/ALDP-9BE5BE/2020 | 2020-09-10 | NA |
| England/QEUH-9C995A/2020 | 2020-09-10 | NA |
| England/QEUH-9C8566/2020 | 2020-09-11 | NA |
| England/ALDP-9C4D80/2020 | 2020-09-11 | NA |
| Wales/PHWC-16ABC6/2020 | 2020-09-11 | NA |
| Wales/QEUH-9C87AC/2020 | 2020-09-11 | NA |
| England/ALDP-9C4CB0/2020 | 2020-09-11 | NA |
| England/QEUH-9C7DAB/2020 | 2020-09-11 | NA |
| England/ALDP-9C5A88/2020 | 2020-09-11 | NA |
| England/MILK-9C3AA8/2020 | 2020-09-11 | NA |
| England/QEUH-9C7FE1/2020 | 2020-09-11 | NA |
| England/QEUH-9C7DBA/2020 | 2020-09-11 | NA |
| England/ALDP-9C5A79/2020 | 2020-09-11 | NA |
| England/ALDP-9C5AA6/2020 | 2020-09-11 | NA |
| England/MILK-9C312B/2020 | 2020-09-11 | NA |
| England/ALDP-9C5860/2020 | 2020-09-11 | NA |
| England/QEUH-9C7B1A/2020 | 2020-09-11 | NA |
| England/QEUH-9C91E9/2020 | 2020-09-11 | NA |
| England/QEUH-9D16B2/2020 | 2020-09-11 | NA |
| England/NORT-2A4804/2020 | 2020-09-11 | NA |
| England/NORT-2A47F8/2020 | 2020-09-11 | NA |
| England/QEUH-9C7E4E/2020 | 2020-09-11 | NA |
| England/QEUH-9C7FB4/2020 | 2020-09-11 | NA |
| England/QEUH-9C7ED5/2020 | 2020-09-11 | NA |
| England/NORT-2A485F/2020 | 2020-09-11 | NA |
| England/QEUH-9C7F69/2020 | 2020-09-11 | NA |
| England/QEUH-9C835D/2020 | 2020-09-11 | NA |
| England/QEUH-9C896A/2020 | 2020-09-11 | NA |
| England/NOTT-11317F/2020 | 2020-09-11 | NA |
| England/NOTT-11369E/2020 | 2020-09-11 | NA |
| Wales/PHWC-16AE72/2020 | 2020-09-11 | NA |
| England/ALDP-9C5A3D/2020 | 2020-09-11 | NA |
| Wales/PHWC-16B14B/2020 | 2020-09-12 | NA |
| Wales/PHWC-16A6D4/2020 | 2020-09-12 | NA |
| England/NORT-2A48C8/2020 | 2020-09-12 | NA |
| England/NORT-2A48B9/2020 | 2020-09-12 | NA |
| England/QEUH-9CFF52/2020 | 2020-09-13 | NA |
| England/QEUH-9CA33D/2020 | 2020-09-13 | NA |
| England/QEUH-9CA2F4/2020 | 2020-09-13 | NA |
| England/QEUH-9CA31F/2020 | 2020-09-13 | NA |
| England/QEUH-9C9310/2020 | 2020-09-13 | NA |
| England/QEUH-9CA388/2020 | 2020-09-13 | NA |
| Scotland/CVR4318/2020 | 2020-09-13 | NA |
| Wales/PHWC-16B73A/2020 | 2020-09-14 | NA |
| England/QEUH-9D1209/2020 | 2020-09-14 | NA |
| England/QEUH-9CFE82/2020 | 2020-09-14 | NA |
| England/SHEF-C3C05/2020 | 2020-09-14 | NA |
| England/QEUH-9D1658/2020 | 2020-09-14 | NA |
| England/QEUH-9D0D81/2020 | 2020-09-14 | NA |
| England/QEUH-9D0E7F/2020 | 2020-09-14 | NA |
| England/NORT-2A49E3/2020 | 2020-09-14 | NA |
| England/NORT-2A49F2/2020 | 2020-09-14 | NA |
| Wales/QEUH-9CFF70/2020 | 2020-09-14 | NA |
| England/QEUH-9D05D4/2020 | 2020-09-14 | NA |
| Wales/PHWC-16B2FD/2020 | 2020-09-14 | NA |
| England/NOTT-1136BC/2020 | 2020-09-14 | NA |
| England/NOTT-11387A/2020 | 2020-09-14 | NA |
| Wales/PHWC-16B0E4/2020 | 2020-09-14 | NA |
| England/QEUH-9CE1E4/2020 | 2020-09-15 | NA |
| Wales/PHWC-16B22A/2020 | 2020-09-15 | NA |
| England/QEUH-9D0668/2020 | 2020-09-15 | NA |
| England/QEUH-9CE1F3/2020 | 2020-09-15 | NA |
| England/QEUH-9D1236/2020 | 2020-09-15 | NA |
| England/NOTT-11385C/2020 | 2020-09-15 | NA |
| England/MILK-9D5144/2020 | 2020-09-15 | NA |
| England/QEUH-9CF0C8/2020 | 2020-09-15 | NA |
| England/NOTT-11393B/2020 | 2020-09-15 | NA |
| England/NOTT-11386B/2020 | 2020-09-15 | NA |
| England/NOTT-1138B6/2020 | 2020-09-15 | NA |
| England/NOTT-1132E5/2020 | 2020-09-15 | NA |
| England/NOTT-1138A7/2020 | 2020-09-15 | NA |
| England/NOTT-11392C/2020 | 2020-09-15 | NA |
| England/NOTT-113898/2020 | 2020-09-15 | NA |
| England/NOTT-1138F2/2020 | 2020-09-15 | NA |
| England/NOTT-11391D/2020 | 2020-09-15 | NA |
| England/NOTT-11390E/2020 | 2020-09-15 | NA |
| Wales/PHWC-16BD47/2020 | 2020-09-15 | NA |
| England/QEUH-9D1193/2020 | 2020-09-16 | NA |
| England/QEUH-9D1157/2020 | 2020-09-16 | NA |
| England/ALDP-9CCE85/2020 | 2020-09-16 | NA |
| England/ALDP-9CBCF5/2020 | 2020-09-16 | NA |
| England/QEUH-9D1791/2020 | 2020-09-16 | NA |
| England/ALDP-9CAF57/2020 | 2020-09-16 | NA |
| England/ALDP-9CDA08/2020 | 2020-09-16 | NA |
| England/ALDP-9CCE58/2020 | 2020-09-16 | NA |
| England/ALDP-9CD9DE/2020 | 2020-09-16 | NA |
| England/QEUH-9D1B77/2020 | 2020-09-16 | NA |
| England/QEUH-9D1746/2020 | 2020-09-16 | NA |
| England/QEUH-9D0835/2020 | 2020-09-16 | NA |
| England/QEUH-9D0194/2020 | 2020-09-16 | NA |
| England/QEUH-9CFD94/2020 | 2020-09-16 | NA |
| England/QEUH-9D01C1/2020 | 2020-09-16 | NA |
| England/QEUH-9D112A/2020 | 2020-09-16 | NA |
| England/ALDP-9CD8C2/2020 | 2020-09-16 | NA |
| England/ALDP-9CDDB4/2020 | 2020-09-16 | NA |
| Wales/PHWC-16B9CB/2020 | 2020-09-16 | NA |
| Wales/PHWC-16AF7F/2020 | 2020-09-16 | NA |
| England/QEUH-9CFDB2/2020 | 2020-09-16 | NA |
| England/QEUH-9D02EC/2020 | 2020-09-16 | NA |
| England/QEUH-9D1254/2020 | 2020-09-16 | NA |
| England/QEUH-9CFDC1/2020 | 2020-09-16 | NA |
| England/ALDP-9CAFC0/2020 | 2020-09-16 | NA |
| England/ALDP-9CAF93/2020 | 2020-09-16 | NA |
| England/QEUH-9D1843/2020 | 2020-09-16 | NA |
| England/QEUH-9D1861/2020 | 2020-09-16 | NA |
| England/QEUH-9D0756/2020 | 2020-09-16 | NA |
| England/QEUH-9D02FB/2020 | 2020-09-16 | NA |
| England/QEUH-9D18DA/2020 | 2020-09-16 | NA |
| England/QEUH-9D188F/2020 | 2020-09-16 | NA |
| England/QEUH-9D07ED/2020 | 2020-09-16 | NA |
| England/QEUH-9D06E0/2020 | 2020-09-16 | NA |
| England/ALDP-9CD9B0/2020 | 2020-09-16 | NA |
| England/ALDP-9CCE67/2020 | 2020-09-16 | NA |
| Scotland/QEUH-9CE2F0/2020 | 2020-09-16 | NA |
| England/ALDP-9CBD98/2020 | 2020-09-16 | NA |
| England/ALDP-9CBD7A/2020 | 2020-09-16 | NA |
| England/ALDP-9CD112/2020 | 2020-09-16 | NA |
| England/ALDP-9CD6AA/2020 | 2020-09-16 | NA |
| England/ALDP-9CD68C/2020 | 2020-09-16 | NA |
| England/ALDP-9CAEB4/2020 | 2020-09-16 | NA |
| England/ALDP-9CAEA5/2020 | 2020-09-16 | NA |
| England/ALDP-9CD69B/2020 | 2020-09-16 | NA |
| England/ALDP-9CD77A/2020 | 2020-09-16 | NA |
| England/CAMC-9D7467/2020 | 2020-09-16 | NA |
| Wales/ALDP-9CBBAD/2020 | 2020-09-16 | NA |
| England/ALDP-9CAE5A/2020 | 2020-09-16 | NA |
| England/ALDP-9CC034/2020 | 2020-09-16 | NA |
| England/ALDP-9CDCF3/2020 | 2020-09-16 | NA |
| England/ALDP-9CDD69/2020 | 2020-09-16 | NA |
| England/ALDP-9CCD6A/2020 | 2020-09-16 | NA |
| England/ALDP-9CDA44/2020 | 2020-09-16 | NA |
| England/ALDP-9CC456/2020 | 2020-09-16 | NA |
| England/ALDP-9CDA53/2020 | 2020-09-16 | NA |
| England/ALDP-9CAA65/2020 | 2020-09-16 | NA |
| Scotland/CVR4315/2020 | 2020-09-16 | NA |
| England/ALDP-9CAE4B/2020 | 2020-09-16 | NA |
| England/ALDP-9CD3EF/2020 | 2020-09-16 | NA |
| England/QEUH-9D2BFE/2020 | 2020-09-16 | NA |
| England/QEUH-9D17EC/2020 | 2020-09-16 | NA |
| England/MILK-9D3E8B/2020 | 2020-09-16 | NA |
| England/QEUH-9D09F6/2020 | 2020-09-16 | NA |
| England/QEUH-9D02CE/2020 | 2020-09-16 | NA |
| England/QEUH-9D19B9/2020 | 2020-09-16 | NA |
| England/QEUH-9D0A6C/2020 | 2020-09-16 | NA |
| England/QEUH-9D189E/2020 | 2020-09-16 | NA |
| England/QEUH-9D08BD/2020 | 2020-09-16 | NA |
| England/QEUH-9D01D0/2020 | 2020-09-16 | NA |
| England/QEUH-9D08DB/2020 | 2020-09-16 | NA |
| England/QEUH-9D0B5A/2020 | 2020-09-16 | NA |
| England/ALDP-9CCF73/2020 | 2020-09-16 | NA |
| England/ALDP-9CCF19/2020 | 2020-09-16 | NA |
| England/NOTT-1136E9/2020 | 2020-09-16 | NA |
| England/QEUH-9D0361/2020 | 2020-09-16 | NA |
| Scotland/QEUH-9D09BA/2020 | 2020-09-16 | NA |
| England/ALDP-9CB1AB/2020 | 2020-09-16 | NA |
| England/ALDP-9CB123/2020 | 2020-09-16 | NA |
| England/QEUH-9D1904/2020 | 2020-09-16 | NA |
| England/QEUH-9D0B0F/2020 | 2020-09-16 | NA |
| England/QEUH-9D0A20/2020 | 2020-09-16 | NA |
| England/QEUH-9CF963/2020 | 2020-09-16 | NA |
| England/ALDP-9CBF65/2020 | 2020-09-16 | NA |
| England/ALDP-9CAD11/2020 | 2020-09-16 | NA |
| England/ALDP-9CC447/2020 | 2020-09-16 | NA |
| England/MILK-9D3D33/2020 | 2020-09-17 | NA |
| England/NORT-2A4D14/2020 | 2020-09-17 | NA |
| England/MILK-9D4F87/2020 | 2020-09-17 | NA |
| England/QEUH-9CF7F0/2020 | 2020-09-17 | NA |
| England/QEUH-9CF3ED/2020 | 2020-09-17 | NA |
| England/QEUH-9CE8A3/2020 | 2020-09-17 | NA |
| England/QEUH-9CF031/2020 | 2020-09-17 | NA |
| England/QEUH-9CE4CD/2020 | 2020-09-17 | NA |
| England/QEUH-9CE490/2020 | 2020-09-17 | NA |
| England/QEUH-9CED86/2020 | 2020-09-17 | NA |
| England/NORT-2A4DAB/2020 | 2020-09-17 | NA |
| England/NORT-2A4DC9/2020 | 2020-09-17 | NA |
| England/QEUH-9CED95/2020 | 2020-09-17 | NA |
| England/QEUH-9CF839/2020 | 2020-09-17 | NA |
| England/NORT-2A4D41/2020 | 2020-09-17 | NA |
| England/QEUH-9CE849/2020 | 2020-09-17 | NA |
| England/ALDP-9CC0BC/2020 | 2020-09-17 | NA |
| England/QEUH-9CF65D/2020 | 2020-09-17 | NA |
| England/QEUH-9D2C19/2020 | 2020-09-17 | NA |
| England/NORT-2A4B92/2020 | 2020-09-17 | NA |
| England/QEUH-9CF4EA/2020 | 2020-09-17 | NA |
| England/ALDP-9D7DF3/2020 | 2020-09-17 | NA |
| England/QEUH-9CE9FB/2020 | 2020-09-17 | NA |
| England/QEUH-9CE4BE/2020 | 2020-09-17 | NA |
| England/QEUH-9CE83A/2020 | 2020-09-17 | NA |
| England/QEUH-9CEB31/2020 | 2020-09-17 | NA |
| England/QEUH-9CE7E2/2020 | 2020-09-17 | NA |
| England/ALDP-9D7DE4/2020 | 2020-09-17 | NA |
| England/MILK-9D45EF/2020 | 2020-09-17 | NA |
| Scotland/CVR4398/2020 | 2020-09-17 | NA |
| England/MILK-9D3D42/2020 | 2020-09-17 | NA |
| England/QEUH-9CE8D0/2020 | 2020-09-17 | NA |
| England/QEUH-9CF59C/2020 | 2020-09-17 | NA |
| England/QEUH-9CE454/2020 | 2020-09-17 | NA |
| England/QEUH-9CF7C3/2020 | 2020-09-17 | NA |
| England/QEUH-9CE876/2020 | 2020-09-17 | NA |
| England/QEUH-9CF6F3/2020 | 2020-09-17 | NA |
| England/QEUH-9D2806/2020 | 2020-09-17 | NA |
| England/PORT-2D4337/2020 | 2020-09-17 | NA |
| England/QEUH-9CE472/2020 | 2020-09-17 | NA |
| Wales/PHWC-16C12C/2020 | 2020-09-17 | NA |
| Wales/PHWC-16BFD8/2020 | 2020-09-17 | NA |
| England/QEUH-9CE5AC/2020 | 2020-09-17 | NA |
| England/CAMC-9D7212/2020 | 2020-09-18 | NA |
| England/ALDP-9D7FA2/2020 | 2020-09-18 | NA |
| England/ALDP-9D786B/2020 | 2020-09-18 | NA |
| England/ALDP-9D783E/2020 | 2020-09-18 | NA |
| England/ALDP-9DA2D3/2020 | 2020-09-18 | NA |
| England/MILK-9F2879/2020 | 2020-09-18 | NA |
| England/QEUH-9D303A/2020 | 2020-09-18 | NA |
| Wales/MILK-9D3F6A/2020 | 2020-09-18 | NA |
| England/MILK-9D3DBB/2020 | 2020-09-18 | NA |
| Wales/MILK-9D3FB5/2020 | 2020-09-18 | NA |
| England/MILK-9D4716/2020 | 2020-09-18 | NA |
| England/NORT-2A4EA8/2020 | 2020-09-18 | NA |
| Wales/ALDP-9D8BF8/2020 | 2020-09-18 | NA |
| England/MILK-9E02BC/2020 | 2020-09-18 | NA |
| England/ALDP-9D75CE/2020 | 2020-09-18 | NA |
| England/MILK-9D4549/2020 | 2020-09-18 | NA |
| England/NORT-1B6BEDE/2020 | 2020-09-18 | NA |
| England/QEUH-9D2CFB/2020 | 2020-09-18 | NA |
| England/NORT-2A4D9C/2020 | 2020-09-18 | NA |
| Wales/PHWC-16BE35/2020 | 2020-09-18 | NA |
| England/ALDP-9D923E/2020 | 2020-09-18 | NA |
| England/ALDP-9D9298/2020 | 2020-09-18 | NA |
| England/ALDP-9D8C8C/2020 | 2020-09-18 | NA |
| England/QEUH-9D3119/2020 | 2020-09-18 | NA |
| England/QEUH-9D36FC/2020 | 2020-09-18 | NA |
| England/CAMC-9D731F/2020 | 2020-09-18 | NA |
| England/ALDP-9D9BD9/2020 | 2020-09-18 | NA |
| England/CAMC-9D750A/2020 | 2020-09-18 | NA |
| England/ALDP-9D8F56/2020 | 2020-09-18 | NA |
| England/MILK-9D47AD/2020 | 2020-09-18 | NA |
| Wales/PHWC-483881/2020 | 2020-09-18 | NA |
| Wales/QEUH-9D2F7A/2020 | 2020-09-18 | NA |
| Wales/MILK-9D526F/2020 | 2020-09-18 | NA |
| England/MILK-9D3FD3/2020 | 2020-09-18 | NA |
| England/ALDP-9DA26A/2020 | 2020-09-18 | NA |
| England/ALDP-9D95AE/2020 | 2020-09-18 | NA |
| England/ALDP-9D9DE2/2020 | 2020-09-18 | NA |
| England/QEUH-9D32E9/2020 | 2020-09-18 | NA |
| England/MILK-9E03D7/2020 | 2020-09-18 | NA |
| England/MILK-9D402A/2020 | 2020-09-18 | NA |
| Scotland/MILK-9D51BD/2020 | 2020-09-18 | NA |
| England/NOTT-113986/2020 | 2020-09-18 | NA |
| England/ALDP-9D9A81/2020 | 2020-09-18 | NA |
| England/QEUH-9D2DE9/2020 | 2020-09-18 | NA |
| Wales/MILK-9D52BA/2020 | 2020-09-18 | NA |
| England/ALDP-9D925C/2020 | 2020-09-18 | NA |
| England/ALDP-9D99FD/2020 | 2020-09-18 | NA |
| England/ALDP-9DA17C/2020 | 2020-09-18 | NA |
| England/ALDP-9D785C/2020 | 2020-09-18 | NA |
| Wales/MILK-9D4CDB/2020 | 2020-09-19 | NA |
| Wales/MILK-9D4CEA/2020 | 2020-09-19 | NA |
| England/QEUH-9D26A2/2020 | 2020-09-19 | NA |
| England/QEUH-9D26DF/2020 | 2020-09-19 | NA |
| England/QEUH-9D2420/2020 | 2020-09-19 | NA |
| Wales/MILK-9E153A/2020 | 2020-09-19 | NA |
| Wales/MILK-9D4C26/2020 | 2020-09-19 | NA |
| England/CAMC-9D1EF6/2020 | 2020-09-19 | NA |
| England/MILK-9D4BCF/2020 | 2020-09-19 | NA |
| England/QEUH-9D23F6/2020 | 2020-09-19 | NA |
| England/NORT-2A4E7B/2020 | 2020-09-19 | NA |
| England/QEUH-9D23D8/2020 | 2020-09-19 | NA |
| England/MILK-9D46CE/2020 | 2020-09-19 | NA |
| England/CAMC-9D22CC/2020 | 2020-09-19 | NA |
| England/QEUH-9D2684/2020 | 2020-09-19 | NA |
| England/QEUH-9D2736/2020 | 2020-09-19 | NA |
| England/QEUH-9D2A2E/2020 | 2020-09-19 | NA |
| England/QEUH-9D29E5/2020 | 2020-09-19 | NA |
| England/QEUH-9D2A5B/2020 | 2020-09-19 | NA |
| England/QEUH-9D260C/2020 | 2020-09-19 | NA |
| England/QEUH-9D2402/2020 | 2020-09-19 | NA |
| England/QEUH-9D295E/2020 | 2020-09-19 | NA |
| Wales/MILK-9D56EB/2020 | 2020-09-19 | NA |
| Wales/MILK-9D4CBD/2020 | 2020-09-19 | NA |
| Scotland/CVR4442/2020 | 2020-09-19 | H7 |
| England/QEUH-9D246C/2020 | 2020-09-19 | NA |
| England/QEUH-9D294F/2020 | 2020-09-19 | NA |
| England/QEUH-9D24C6/2020 | 2020-09-19 | NA |
| England/QEUH-9D2E8C/2020 | 2020-09-19 | NA |
| England/QEUH-9D2709/2020 | 2020-09-19 | NA |
| England/MILK-9E02DA/2020 | 2020-09-19 | NA |
| England/CAMC-9D1D35/2020 | 2020-09-19 | NA |
| England/NORT-2A4E5D/2020 | 2020-09-19 | NA |
| England/QEUH-9D28CA/2020 | 2020-09-19 | NA |
| England/MILK-9D4F1E/2020 | 2020-09-19 | NA |
| England/NOTT-1134C1/2020 | 2020-09-19 | NA |
| England/CAMC-9D1F11/2020 | 2020-09-19 | NA |
| England/CAMC-9D2068/2020 | 2020-09-19 | NA |
| England/MILK-9E1716/2020 | 2020-09-19 | NA |
| England/MILK-9D46DD/2020 | 2020-09-19 | NA |
| Wales/MILK-9E0FC4/2020 | 2020-09-20 | NA |
| Wales/CAMC-9D70EB/2020 | 2020-09-20 | NA |
| England/MILK-9E2214/2020 | 2020-09-20 | NA |
| Scotland/CVR4474/2020 | 2020-09-20 | H8 |
| Scotland/CVR4475/2020 | 2020-09-20 | H7 |
| Scotland/CVR4477/2020 | 2020-09-20 | H8 |
| Scotland/CVR4451/2020 | 2020-09-20 | H8 |
| Scotland/CVR4470/2020 | 2020-09-20 | H7 |
| Scotland/CVR4473/2020 | 2020-09-20 | H8 |
| Wales/MILK-9E1181/2020 | 2020-09-20 | NA |
| England/MILK-9E2171/2020 | 2020-09-20 | NA |
| Wales/MILK-9E2047/2020 | 2020-09-20 | NA |
| Wales/MILK-9E2038/2020 | 2020-09-20 | NA |
| England/MILK-9E097B/2020 | 2020-09-20 | NA |
| Wales/MILK-9E087E/2020 | 2020-09-20 | NA |
| Wales/MILK-9E2AA3/2020 | 2020-09-20 | NA |
| Wales/MILK-9E05E0/2020 | 2020-09-20 | NA |
| Scotland/CVR4481/2020 | 2020-09-20 | H8 |
| England/QEUH-9D3243/2020 | 2020-09-20 | NA |
| Wales/CVR4762/2020 | 2020-09-20 | NA |
| England/MILK-9E07EA/2020 | 2020-09-20 | NA |
| England/MILK-9E1F0F/2020 | 2020-09-20 | NA |
| England/MILK-9E096C/2020 | 2020-09-20 | NA |
| England/MILK-9E0999/2020 | 2020-09-20 | NA |
| England/QEUH-9DBE65/2020 | 2020-09-21 | NA |
| Scotland/QEUH-9DEA7D/2020 | 2020-09-21 | H7 |
| England/QEUH-9DCC79/2020 | 2020-09-21 | NA |
| England/QEUH-9DD58C/2020 | 2020-09-21 | NA |
| England/QEUH-9DC6D5/2020 | 2020-09-21 | NA |
| England/QEUH-9DEE35/2020 | 2020-09-21 | NA |
| England/QEUH-9DDEAF/2020 | 2020-09-21 | NA |
| England/QEUH-9DD6A7/2020 | 2020-09-21 | NA |
| England/QEUH-9DDB7B/2020 | 2020-09-21 | NA |
| England/QEUH-9DDA41/2020 | 2020-09-21 | NA |
| England/QEUH-9DDC3C/2020 | 2020-09-21 | NA |
| England/QEUH-9DDC5A/2020 | 2020-09-21 | NA |
| Scotland/CVR4516/2020 | 2020-09-21 | H7 |
| Scotland/CVR4499/2020 | 2020-09-21 | H8 |
| Scotland/CVR4515/2020 | 2020-09-21 | H8 |
| Scotland/CVR4490/2020 | 2020-09-21 | H8 |
| Scotland/CVR4512/2020 | 2020-09-21 | H7 |
| Scotland/CVR4488/2020 | 2020-09-21 | H7 |
| Scotland/QEUH-9DE6B5/2020 | 2020-09-21 | H8 |
| Scotland/QEUH-9DEA6E/2020 | 2020-09-21 | Private |
| Germany/NW-HHU-263/2020 | 2020-09-21 | NA |
| England/QEUH-9DE13C/2020 | 2020-09-21 | NA |
| England/QEUH-9DED1A/2020 | 2020-09-21 | NA |
| England/QEUH-9DDD0C/2020 | 2020-09-21 | NA |
| England/QEUH-9DE4E8/2020 | 2020-09-21 | NA |
| England/QEUH-9DD935/2020 | 2020-09-21 | NA |
| England/QEUH-9DEF9C/2020 | 2020-09-21 | NA |
| England/QEUH-9DE99E/2020 | 2020-09-21 | NA |
| England/QEUH-9DEFC9/2020 | 2020-09-21 | NA |
| England/QEUH-9DD908/2020 | 2020-09-21 | NA |
| England/QEUH-9DDAC9/2020 | 2020-09-21 | NA |
| England/QEUH-9DD8DE/2020 | 2020-09-21 | NA |
| England/QEUH-9DDB8A/2020 | 2020-09-21 | NA |
| England/QEUH-9DDA23/2020 | 2020-09-21 | NA |
| England/QEUH-9DD980/2020 | 2020-09-21 | NA |
| England/QEUH-9DD012/2020 | 2020-09-21 | NA |
| England/QEUH-9DE257/2020 | 2020-09-21 | NA |
| England/QEUH-9DD100/2020 | 2020-09-21 | NA |
| England/QEUH-9DE916/2020 | 2020-09-21 | NA |
| England/QEUH-9DF03E/2020 | 2020-09-21 | NA |
| Scotland/QEUH-9DE6E2/2020 | 2020-09-21 | H5 |
| Scotland/CVR4500/2020 | 2020-09-21 | H5 |
| England/CAMC-9DFE34/2020 | 2020-09-21 | NA |
| England/QEUH-9DD416/2020 | 2020-09-21 | NA |
| England/CAMC-9DFBD3/2020 | 2020-09-21 | NA |
| England/CAMC-9E0049/2020 | 2020-09-21 | NA |
| England/QEUH-9CEE74/2020 | 2020-09-21 | NA |
| England/QEUH-9DCAAC/2020 | 2020-09-21 | NA |
| Scotland/QEUH-9DD7A4/2020 | 2020-09-21 | H5 |
| England/CAMC-9DF5D5/2020 | 2020-09-21 | NA |
| England/CAMB-1B634B/2020 | 2020-09-21 | NA |
| England/QEUH-9DE21B/2020 | 2020-09-21 | NA |
| Scotland/QEUH-9DA877/2020 | 2020-09-21 | NA |
| Scotland/CVR4494/2020 | 2020-09-21 | H5 |
| Scotland/CVR4497/2020 | 2020-09-21 | H5 |
| Scotland/CVR4522/2020 | 2020-09-21 | H7 |
| England/QEUH-9DE239/2020 | 2020-09-21 | NA |
| England/QEUH-9DDA9C/2020 | 2020-09-21 | NA |
| England/QEUH-9DDFCA/2020 | 2020-09-21 | NA |
| England/CAMC-9DFF7D/2020 | 2020-09-21 | NA |
| England/CAMC-9DFDCE/2020 | 2020-09-21 | NA |
| England/CAMC-9DFDEC/2020 | 2020-09-21 | NA |
| Scotland/CVR5106/2020 | 2020-09-21 | NA |
| England/NOTT-1139FF/2020 | 2020-09-21 | NA |
| England/CAMC-9DF57B/2020 | 2020-09-21 | NA |
| England/QEUH-9DC620/2020 | 2020-09-21 | NA |
| England/MILK-9E2E98/2020 | 2020-09-21 | NA |
| England/QEUH-9DC65D/2020 | 2020-09-21 | NA |
| England/QEUH-9DC6A8/2020 | 2020-09-21 | NA |
| Scotland/QEUH-9DEDDE/2020 | 2020-09-21 | H8 |
| Scotland/CVR4508/2020 | 2020-09-21 | H5 |
| Scotland/QEUH-9DEDFC/2020 | 2020-09-21 | H8 |
| England/QEUH-9DDB5D/2020 | 2020-09-21 | NA |
| England/MILK-9E0647/2020 | 2020-09-21 | NA |
| England/QEUH-9DEF7E/2020 | 2020-09-21 | NA |
| England/QEUH-9DDFAC/2020 | 2020-09-21 | NA |
| England/QEUH-9DD0F4/2020 | 2020-09-21 | NA |
| Scotland/QEUH-9DD786/2020 | 2020-09-21 | H8 |
| England/QEUH-9DDA14/2020 | 2020-09-21 | NA |
| England/QEUH-9DE187/2020 | 2020-09-21 | NA |
| England/QEUH-9DD892/2020 | 2020-09-21 | NA |
| England/QEUH-9DED29/2020 | 2020-09-21 | NA |
| England/QEUH-9E4BAE/2020 | 2020-09-21 | NA |
| Scotland/CVR5405/2020 | 2020-09-22 | NA |
| Scotland/QEUH-9DA80E/2020 | 2020-09-22 | NA |
| Scotland/QEUH-9DA9DE/2020 | 2020-09-22 | NA |
| England/QEUH-9E4B44/2020 | 2020-09-22 | NA |
| Scotland/CVR5398/2020 | 2020-09-22 | NA |
| Scotland/CVR5394/2020 | 2020-09-22 | NA |
| Scotland/QEUH-9DCC97/2020 | 2020-09-22 | NA |
| Scotland/QEUH-9DD15B/2020 | 2020-09-22 | NA |
| Scotland/CVR4525/2020 | 2020-09-22 | H8 |
| Scotland/CVR4552/2020 | 2020-09-22 | H8 |
| Scotland/CVR4534/2020 | 2020-09-22 | H7 |
| Scotland/CVR4529/2020 | 2020-09-22 | H8 |
| Scotland/CVR4541/2020 | 2020-09-22 | H8 |
| Scotland/CVR4561/2020 | 2020-09-22 | H7 |
| Wales/PHWC-16D4E6/2020 | 2020-09-22 | NA |
| England/MILK-9F2958/2020 | 2020-09-22 | NA |
| England/QEUH-9DBFAE/2020 | 2020-09-22 | NA |
| England/QEUH-9DAE75/2020 | 2020-09-22 | NA |
| England/QEUH-9DAEA2/2020 | 2020-09-22 | NA |
| Scotland/CVR4558/2020 | 2020-09-22 | H7 |
| England/QEUH-9DABBA/2020 | 2020-09-22 | NA |
| England/QEUH-9E426D/2020 | 2020-09-22 | NA |
| England/QEUH-9DACF3/2020 | 2020-09-22 | NA |
| England/QEUH-9FAF03/2020 | 2020-09-22 | NA |
| Wales/PHWC-16CAD6/2020 | 2020-09-22 | NA |
| England/QEUH-9DABE7/2020 | 2020-09-22 | NA |
| England/QEUH-9DA73E/2020 | 2020-09-22 | NA |
| England/QEUH-9DA72F/2020 | 2020-09-22 | NA |
| England/QEUH-9E5633/2020 | 2020-09-22 | NA |
| Scotland/QEUH-9DAA35/2020 | 2020-09-22 | NA |
| Scotland/QEUH-9DC347/2020 | 2020-09-22 | NA |
| Scotland/CVR4557/2020 | 2020-09-22 | H7 |
| Scotland/CVR4527/2020 | 2020-09-22 | H6 |
| England/QEUH-9E4E5A/2020 | 2020-09-22 | NA |
| Scotland/CVR4531/2020 | 2020-09-22 | H7 |
| Scotland/CVR4530/2020 | 2020-09-22 | NA |
| England/QEUH-9DADD2/2020 | 2020-09-22 | NA |
| England/QEUH-9DAD2D/2020 | 2020-09-22 | NA |
| England/QEUH-9DAFDC/2020 | 2020-09-22 | NA |
| England/QEUH-9DADE1/2020 | 2020-09-22 | NA |
| England/QEUH-9DAD1E/2020 | 2020-09-22 | NA |
| England/QEUH-9DADC3/2020 | 2020-09-22 | NA |
| Scotland/QEUH-9DC408/2020 | 2020-09-22 | NA |
| England/QEUH-9E45EC/2020 | 2020-09-22 | NA |
| United_Arab_Emirates/skmc-3890161/2020 | 2020-09-23 | NA |
| England/CAMC-9EC9BB/2020 | 2020-09-23 | NA |
| Scotland/QEUH-9E386C/2020 | 2020-09-23 | H5 |
| England/QEUH-9E4616/2020 | 2020-09-23 | NA |
| England/QEUH-9F33FF/2020 | 2020-09-23 | NA |
| Scotland/QEUH-9E4A74/2020 | 2020-09-23 | H7 |
| Scotland/QEUH-9E435B/2020 | 2020-09-23 | NA |
| England/MILK-9EE63A/2020 | 2020-09-23 | NA |
| England/QEUH-9E477D/2020 | 2020-09-23 | NA |
| England/QEUH-9E47B9/2020 | 2020-09-23 | NA |
| England/MILK-9EE834/2020 | 2020-09-23 | NA |
| England/CAMC-9ECA21/2020 | 2020-09-23 | NA |
| England/QEUH-9E492C/2020 | 2020-09-23 | NA |
| England/QEUH-9E387B/2020 | 2020-09-23 | NA |
| England/QEUH-9E53E1/2020 | 2020-09-23 | NA |
| Netherlands/GE-RIVM-10374/2020 | 2020-09-23 | NA |
| Scotland/QEUH-9E52A8/2020 | 2020-09-23 | H8 |
| England/MILK-9F2730/2020 | 2020-09-23 | NA |
| England/ALDP-9E85BB/2020 | 2020-09-23 | NA |
| Scotland/EDB7914/2020 | 2020-09-23 | NA |
| Wales/PHWC-480ACA/2020 | 2020-09-23 | NA |
| England/QEUH-9F4EA2/2020 | 2020-09-23 | NA |
| England/MILK-9EE8E9/2020 | 2020-09-23 | NA |
| Wales/MILK-9EE9E6/2020 | 2020-09-23 | NA |
| England/QEUH-9E478C/2020 | 2020-09-23 | NA |
| England/CAMC-9EBB4D/2020 | 2020-09-23 | NA |
| England/QEUH-9E493B/2020 | 2020-09-23 | NA |
| England/CAMC-9EC308/2020 | 2020-09-23 | NA |
| England/MILK-9EE9AA/2020 | 2020-09-23 | NA |
| England/MILK-9EE9C8/2020 | 2020-09-23 | NA |
| Scotland/QEUH-9E52C6/2020 | 2020-09-23 | NA |
| England/MILK-9EE931/2020 | 2020-09-23 | NA |
| England/MILK-9EE676/2020 | 2020-09-23 | NA |
| England/MILK-9EE588/2020 | 2020-09-23 | NA |
| England/QEUH-9E4977/2020 | 2020-09-23 | NA |
| Scotland/QEUH-9E4F0C/2020 | 2020-09-24 | NA |
| England/ALDP-9E8FAE/2020 | 2020-09-24 | NA |
| England/MILK-9F0D30/2020 | 2020-09-24 | NA |
| England/QEUH-9E36EA/2020 | 2020-09-24 | NA |
| England/ALDP-9E767D/2020 | 2020-09-24 | NA |
| England/QEUH-9F332C/2020 | 2020-09-24 | NA |
| England/MILK-9F1CC9/2020 | 2020-09-24 | NA |
| England/MILK-9F0A1B/2020 | 2020-09-24 | NA |
| England/ALDP-9EA197/2020 | 2020-09-24 | NA |
| England/CAMC-9EC58A/2020 | 2020-09-24 | NA |
| England/ALDP-9E97D2/2020 | 2020-09-24 | NA |
| England/QEUH-9F3C6D/2020 | 2020-09-24 | NA |
| England/ALDP-9EA14C/2020 | 2020-09-24 | NA |
| England/QEUH-9E378D/2020 | 2020-09-24 | NA |
| England/QEUH-9F3A45/2020 | 2020-09-24 | NA |
| England/ALDP-9E8858/2020 | 2020-09-24 | NA |
| England/ALDP-9EA1A6/2020 | 2020-09-24 | NA |
| England/ALDP-9E691B/2020 | 2020-09-24 | NA |
| England/ALDP-9E7D69/2020 | 2020-09-24 | NA |
| England/QEUH-9F373F/2020 | 2020-09-24 | NA |
| England/ALDP-9E72E2/2020 | 2020-09-24 | NA |
| England/QEUH-9E5475/2020 | 2020-09-24 | NA |
| England/ALDP-9E6CB8/2020 | 2020-09-24 | NA |
| England/ALDP-9E59B2/2020 | 2020-09-24 | NA |
| England/ALDP-9E84FA/2020 | 2020-09-24 | NA |
| England/QEUH-9F3896/2020 | 2020-09-24 | NA |
| England/ALDP-9E83A2/2020 | 2020-09-24 | NA |
| England/ALDP-9E818A/2020 | 2020-09-24 | NA |
| England/ALDP-9E6BAC/2020 | 2020-09-24 | NA |
| England/ALDP-9E8199/2020 | 2020-09-24 | NA |
| England/ALDP-9E77A7/2020 | 2020-09-24 | NA |
| England/ALDP-9E625C/2020 | 2020-09-24 | NA |
| England/ALDP-9E7D87/2020 | 2020-09-24 | NA |
| England/QEUH-9F3A54/2020 | 2020-09-24 | NA |
| England/ALDP-9E9C97/2020 | 2020-09-24 | NA |
| England/ALDP-9E816C/2020 | 2020-09-24 | NA |
| England/MILK-9F268E/2020 | 2020-09-24 | NA |
| England/ALDP-9E59EF/2020 | 2020-09-24 | NA |
| England/MILK-9F0A75/2020 | 2020-09-24 | NA |
| England/ALDP-9E908C/2020 | 2020-09-24 | NA |
| England/ALDP-9E707F/2020 | 2020-09-24 | NA |
| England/CAMC-9EC57B/2020 | 2020-09-24 | NA |
| England/ALDP-9EAB5D/2020 | 2020-09-24 | NA |
| England/ALDP-9EA9EA/2020 | 2020-09-24 | NA |
| England/ALDP-9EABF3/2020 | 2020-09-24 | NA |
| England/ALDP-9E73EF/2020 | 2020-09-24 | NA |
| England/ALDP-9E9347/2020 | 2020-09-24 | NA |
| Wales/PHWC-16D756/2020 | 2020-09-24 | NA |
| England/ALDP-9E94EA/2020 | 2020-09-24 | NA |
| England/ALDP-9E7E0C/2020 | 2020-09-24 | NA |
| England/QEUH-9F51D5/2020 | 2020-09-24 | NA |
| England/CAMC-9EC0B6/2020 | 2020-09-24 | NA |
| England/CAMC-9EC1FF/2020 | 2020-09-24 | NA |
| England/CAMC-9EC1D1/2020 | 2020-09-24 | NA |
| England/QEUH-9F40D9/2020 | 2020-09-24 | NA |
| England/QEUH-9F374E/2020 | 2020-09-24 | NA |
| Scotland/QEUH-9F3104/2020 | 2020-09-24 | H5 |
| England/ALDP-9EA81A/2020 | 2020-09-24 | NA |
| England/ALDP-9E8F17/2020 | 2020-09-24 | NA |
| England/ALDP-9EA328/2020 | 2020-09-24 | NA |
| England/ALDP-9E60E9/2020 | 2020-09-24 | NA |
| England/ALDP-9EA0C7/2020 | 2020-09-24 | NA |
| England/ALDP-9EA07C/2020 | 2020-09-24 | NA |
| Wales/PHWC-16CD28/2020 | 2020-09-24 | NA |
| England/ALDP-9E766E/2020 | 2020-09-24 | NA |
| England/ALDP-9E76AA/2020 | 2020-09-24 | NA |
| England/ALDP-9EA892/2020 | 2020-09-24 | NA |
| England/ALDP-9E6FEC/2020 | 2020-09-24 | NA |
| England/CAMC-9EC2ED/2020 | 2020-09-24 | NA |
| England/QEUH-9F2C7D/2020 | 2020-09-24 | NA |
| England/QEUH-9F3DA6/2020 | 2020-09-24 | NA |
| England/QEUH-9E3EE2/2020 | 2020-09-24 | NA |
| England/QEUH-9F36E7/2020 | 2020-09-24 | NA |
| Scotland/QEUH-9E54EE/2020 | 2020-09-24 | NA |
| England/ALDP-9EA1C4/2020 | 2020-09-24 | NA |
| England/ALDP-9E5712/2020 | 2020-09-24 | NA |
| England/ALDP-9E674E/2020 | 2020-09-24 | NA |
| England/ALDP-9E8AAD/2020 | 2020-09-24 | NA |
| England/QEUH-9F3C8B/2020 | 2020-09-24 | NA |
| Scotland/QEUH-9E51BA/2020 | 2020-09-24 | NA |
| England/QEUH-9F75F5/2020 | 2020-09-24 | NA |
| England/ALDP-9E9392/2020 | 2020-09-24 | NA |
| England/QEUH-9F36F6/2020 | 2020-09-24 | NA |
| England/ALDP-9E6948/2020 | 2020-09-24 | NA |
| Scotland/QEUH-9E541B/2020 | 2020-09-24 | NA |
| England/ALDP-9E6720/2020 | 2020-09-24 | NA |
| England/ALDP-9E8F9F/2020 | 2020-09-24 | NA |
| England/ALDP-9E95BA/2020 | 2020-09-24 | NA |
| England/ALDP-9E95D8/2020 | 2020-09-24 | NA |
| England/ALDP-9E95F6/2020 | 2020-09-24 | NA |
| England/ALDP-9E8849/2020 | 2020-09-24 | NA |
| England/QEUH-9F523C/2020 | 2020-09-25 | NA |
| England/QEUH-9F5C10/2020 | 2020-09-25 | NA |
| England/EXET-13803B/2020 | 2020-09-25 | NA |
| Scotland/QEUH-9F6BB8/2020 | 2020-09-25 | NA |
| England/QEUH-9F4877/2020 | 2020-09-25 | NA |
| England/QEUH-9F7434/2020 | 2020-09-25 | NA |
| England/MILK-9F134C/2020 | 2020-09-25 | NA |
| England/QEUH-9F93F9/2020 | 2020-09-25 | NA |
| England/QEUH-9F5B8C/2020 | 2020-09-25 | NA |
| England/QEUH-9F961E/2020 | 2020-09-25 | NA |
| England/QEUH-9F4DC3/2020 | 2020-09-25 | NA |
| England/QEUH-9F58FE/2020 | 2020-09-25 | NA |
| England/QEUH-9F474D/2020 | 2020-09-25 | NA |
| England/QEUH-9F9809/2020 | 2020-09-25 | NA |
| England/QEUH-9F963C/2020 | 2020-09-25 | NA |
| England/MILK-9F135B/2020 | 2020-09-25 | NA |
| England/QEUH-9F921A/2020 | 2020-09-25 | NA |
| England/QEUH-9F473E/2020 | 2020-09-25 | NA |
| England/QEUH-9F49A1/2020 | 2020-09-25 | NA |
| England/QEUH-9F6426/2020 | 2020-09-25 | NA |
| England/QEUH-9F5EDE/2020 | 2020-09-25 | NA |
| England/QEUH-9F9890/2020 | 2020-09-25 | NA |
| England/ALDP-9E75BC/2020 | 2020-09-25 | NA |
| England/MILK-9F2A55/2020 | 2020-09-25 | NA |
| England/QEUH-9F5D0E/2020 | 2020-09-25 | NA |
| England/MILK-9F133D/2020 | 2020-09-25 | NA |
| England/QEUH-9F5BD7/2020 | 2020-09-25 | NA |
| England/QEUH-9F7470/2020 | 2020-09-25 | NA |
| England/QEUH-9F409D/2020 | 2020-09-25 | NA |
| England/QEUH-9F6435/2020 | 2020-09-25 | NA |
| England/MILK-9F283D/2020 | 2020-09-25 | NA |
| England/MILK-9EF50F/2020 | 2020-09-25 | NA |
| England/QEUH-9F9A8B/2020 | 2020-09-25 | NA |
| England/QEUH-9F9362/2020 | 2020-09-26 | NA |
| Wales/PHWC-16DB2D/2020 | 2020-09-26 | NA |
| England/QEUH-9F38F0/2020 | 2020-09-26 | NA |
| England/QEUH-9FA835/2020 | 2020-09-26 | NA |
| England/SHEF-CE87F/2020 | 2020-09-26 | NA |
| England/SHEF-CF1FB/2020 | 2020-09-26 | NA |
| England/NOTT-113AB0/2020 | 2020-09-26 | NA |
| England/QEUH-9F9DDD/2020 | 2020-09-26 | NA |
| England/QEUH-9F92FC/2020 | 2020-09-26 | NA |
| Scotland/QEUH-9F4A71/2020 | 2020-09-27 | NA |
| Scotland/QEUH-9F4516/2020 | 2020-09-27 | NA |
| Scotland/QEUH-9F6B12/2020 | 2020-09-27 | NA |
| Scotland/QEUH-9F4CA8/2020 | 2020-09-27 | NA |
| Scotland/QEUH-9F4CE4/2020 | 2020-09-27 | NA |
| Scotland/QEUH-9F756E/2020 | 2020-09-27 | NA |
| Scotland/QEUH-9F7100/2020 | 2020-09-27 | NA |
| England/QEUH-9F89AD/2020 | 2020-09-27 | NA |
| Scotland/QEUH-9F5FBD/2020 | 2020-09-27 | NA |
| England/QEUH-9F7EEB/2020 | 2020-09-27 | NA |
| England/QEUH-9F7F15/2020 | 2020-09-27 | NA |
| England/QEUH-9F5A52/2020 | 2020-09-27 | NA |
| England/QEUH-9F772C/2020 | 2020-09-27 | NA |
| England/QEUH-9F7C3C/2020 | 2020-09-27 | NA |
| England/QEUH-9F8390/2020 | 2020-09-27 | NA |
| England/QEUH-9F7A50/2020 | 2020-09-27 | NA |
| England/QEUH-9F5357/2020 | 2020-09-27 | NA |
| England/QEUH-9F5032/2020 | 2020-09-27 | NA |
| England/QEUH-9F532A/2020 | 2020-09-27 | NA |
| England/QEUH-9F8099/2020 | 2020-09-27 | NA |
| England/QEUH-9FAEE8/2020 | 2020-09-27 | NA |
| Scotland/QEUH-9F6945/2020 | 2020-09-27 | NA |
| England/QEUH-9F898F/2020 | 2020-09-27 | NA |
| England/QEUH-9F95C6/2020 | 2020-09-27 | NA |
| England/QEUH-9F7E27/2020 | 2020-09-27 | NA |
| England/QEUH-9F7C5A/2020 | 2020-09-27 | NA |
| England/QEUH-9F7908/2020 | 2020-09-27 | NA |
| England/QEUH-9F5E38/2020 | 2020-09-27 | NA |
| England/QEUH-9F5ADA/2020 | 2020-09-27 | NA |
| England/QEUH-9F774A/2020 | 2020-09-27 | NA |
| England/QEUH-9F8925/2020 | 2020-09-27 | NA |
| England/QEUH-9F8BA7/2020 | 2020-09-27 | NA |
| England/QEUH-9F50AB/2020 | 2020-09-27 | NA |
| England/QEUH-9F513F/2020 | 2020-09-27 | NA |
| England/QEUH-9F7865/2020 | 2020-09-27 | NA |
| England/QEUH-9F4E39/2020 | 2020-09-27 | NA |
| England/QEUH-9F4A80/2020 | 2020-09-27 | NA |
| England/QEUH-9F73FB/2020 | 2020-09-27 | NA |
| England/QEUH-9F76D4/2020 | 2020-09-27 | NA |
| England/QEUH-9F5A9E/2020 | 2020-09-27 | NA |
| England/QEUH-9F5A61/2020 | 2020-09-27 | NA |
| England/QEUH-9F873A/2020 | 2020-09-27 | NA |
| England/QEUH-9F7E63/2020 | 2020-09-27 | NA |
| England/QEUH-9F7AAB/2020 | 2020-09-27 | NA |
| England/QEUH-9F484A/2020 | 2020-09-27 | NA |
| England/QEUH-9F5ACB/2020 | 2020-09-27 | NA |
| England/QEUH-9F78A1/2020 | 2020-09-27 | NA |
| England/QEUH-9F8293/2020 | 2020-09-27 | NA |
| England/QEUH-9F74BC/2020 | 2020-09-27 | NA |
| Wales/PHWC-16DDEB/2020 | 2020-09-27 | NA |
| England/QEUH-9F5ABC/2020 | 2020-09-27 | NA |
| England/QEUH-9F8916/2020 | 2020-09-27 | NA |
| England/QEUH-9F8970/2020 | 2020-09-27 | NA |
| England/QEUH-9F6C6A/2020 | 2020-09-27 | NA |
| England/QEUH-9F515D/2020 | 2020-09-27 | NA |
| England/QEUH-9F7D84/2020 | 2020-09-27 | NA |
| England/QEUH-9F7F8E/2020 | 2020-09-27 | NA |
| England/QEUH-9F80E4/2020 | 2020-09-27 | NA |
| England/QEUH-9F88A0/2020 | 2020-09-27 | NA |
| England/QEUH-9F8BD4/2020 | 2020-09-27 | NA |
| England/QEUH-9F803F/2020 | 2020-09-27 | NA |
| England/QEUH-9F8837/2020 | 2020-09-27 | NA |
| England/QEUH-9FAB96/2020 | 2020-09-27 | NA |
| England/QEUH-9F95E4/2020 | 2020-09-27 | NA |
| England/QEUH-9F516C/2020 | 2020-09-27 | NA |
| England/QEUH-9F7980/2020 | 2020-09-27 | NA |
| England/QEUH-9F5D86/2020 | 2020-09-27 | NA |
| England/QEUH-9F7E36/2020 | 2020-09-27 | NA |
| England/QEUH-9F87EF/2020 | 2020-09-27 | NA |
| Scotland/QEUH-9F4F09/2020 | 2020-09-27 | NA |
| England/QEUH-9F7276/2020 | 2020-09-27 | NA |
| England/QEUH-9F9BE2/2020 | 2020-09-27 | NA |
| England/QEUH-9F8CA4/2020 | 2020-09-27 | NA |
| England/QEUH-9FAE60/2020 | 2020-09-27 | NA |
| England/QEUH-9F5630/2020 | 2020-09-27 | NA |
| England/QEUH-9F7AD8/2020 | 2020-09-27 | NA |
| England/QEUH-9F8336/2020 | 2020-09-27 | NA |
| England/QEUH-9F74DA/2020 | 2020-09-27 | NA |
| England/QEUH-9F74E9/2020 | 2020-09-27 | NA |
| England/QEUH-9F7522/2020 | 2020-09-27 | NA |
| England/QEUH-9F82EE/2020 | 2020-09-27 | NA |
| Scotland/QEUH-9F44DD/2020 | 2020-09-27 | NA |
| Scotland/QEUH-9F4419/2020 | 2020-09-27 | NA |
| England/EXET-138068/2020 | 2020-09-28 | NA |
| England/QEUH-9FA792/2020 | 2020-09-28 | NA |
| England/QEUH-9FAE9D/2020 | 2020-09-28 | NA |
| England/QEUH-9FAD27/2020 | 2020-09-28 | NA |
| Wales/PHWC-16DF8B/2020 | 2020-09-28 | NA |
| England/SHEF-D2052/2020 | 2020-09-28 | NA |
| Denmark/DCGC-20355/2020 | 2020-09-28 | NA |
| Scotland/CVR4732/2020 | 2020-09-28 | NA |
| England/QEUH-9FA422/2020 | 2020-09-28 | NA |
| England/QEUH-9FA6E0/2020 | 2020-09-29 | NA |
| England/QEUH-A1DE48/2020 | 2020-09-29 | NA |
| England/QEUH-9FB078/2020 | 2020-09-29 | NA |
| England/MILK-A05A03/2020 | 2020-09-29 | NA |
| England/QEUH-9FA4F5/2020 | 2020-09-29 | NA |
| England/QEUH-9FA589/2020 | 2020-09-29 | NA |
| England/QEUH-9FA923/2020 | 2020-09-29 | NA |
| England/QEUH-9FB209/2020 | 2020-09-29 | NA |
| England/QEUH-A02954/2020 | 2020-09-29 | NA |
| England/QEUH-9FB0E1/2020 | 2020-09-29 | NA |
| England/QEUH-9FAA3F/2020 | 2020-09-29 | NA |
| Scotland/QEUH-9FB3E8/2020 | 2020-09-29 | NA |
| England/QEUH-A025C9/2020 | 2020-09-29 | NA |
| England/QEUH-A026A8/2020 | 2020-09-29 | NA |
| England/QEUH-A02918/2020 | 2020-09-29 | NA |
| England/QEUH-A024F9/2020 | 2020-09-29 | NA |
| England/NORT-1B871F1/2020 | 2020-09-29 | NA |
| England/QEUH-9FB227/2020 | 2020-09-29 | NA |
| England/QEUH-A029AF/2020 | 2020-09-29 | NA |
| England/QEUH-A1E885/2020 | 2020-09-29 | NA |
| England/NOTT-113C41/2020 | 2020-09-29 | NA |
| England/QEUH-A1DB14/2020 | 2020-09-29 | NA |
| England/QEUH-9FA738/2020 | 2020-09-29 | NA |
| England/PHEC-149671/2020 | 2020-09-29 | NA |
| England/QEUH-9FB281/2020 | 2020-09-29 | NA |
| England/CAMB-1B6378/2020 | 2020-09-30 | NA |
| England/QEUH-9FFC9E/2020 | 2020-09-30 | NA |
| Wales/PHWC-16E0D2/2020 | 2020-09-30 | NA |
| England/MILK-A05C67/2020 | 2020-09-30 | NA |
| England/NORT-28CF3A/2020 | 2020-09-30 | NA |
| England/MILK-A05C85/2020 | 2020-09-30 | NA |
| England/MILK-A05D28/2020 | 2020-09-30 | NA |
| England/MILK-A05BC4/2020 | 2020-09-30 | NA |
| England/MILK-A064D7/2020 | 2020-09-30 | NA |
| England/NORT-1B6BFEA/2020 | 2020-09-30 | NA |
| Wales/PHWC-16E0A5/2020 | 2020-09-30 | NA |
| England/BIRM-653C4/2020 | 2020-09-30 | NA |
| England/QEUH-9FFC07/2020 | 2020-09-30 | NA |
| England/QEUH-A00E48/2020 | 2020-09-30 | NA |
| England/LIVE-1DD672/2020 | 2020-09-30 | NA |
| Scotland/QEUH-A1E050/2020 | 2020-10-01 | NA |
| Scotland/QEUH-A1E09C/2020 | 2020-10-01 | NA |
| England/ALDP-9FCF2F/2020 | 2020-10-01 | NA |
| England/QEUH-A20905/2020 | 2020-10-01 | NA |
| England/QEUH-A1F9BE/2020 | 2020-10-01 | NA |
| England/MILK-A17F87/2020 | 2020-10-01 | NA |
| England/QEUH-A212AF/2020 | 2020-10-01 | NA |
| England/QEUH-A202EC/2020 | 2020-10-01 | NA |
| England/QEUH-A20774/2020 | 2020-10-01 | NA |
| England/QEUH-A0AF1E/2020 | 2020-10-01 | NA |
| England/QEUH-A0AED5/2020 | 2020-10-01 | NA |
| England/ALDP-9FCEC8/2020 | 2020-10-01 | NA |
| England/ALDP-9FDE30/2020 | 2020-10-01 | NA |
| England/QEUH-A00631/2020 | 2020-10-01 | NA |
| England/QEUH-9FFF86/2020 | 2020-10-01 | NA |
| England/QEUH-A202BF/2020 | 2020-10-01 | NA |
| England/NORT-1B6C0C8/2020 | 2020-10-01 | NA |
| England/QEUH-A1EE1A/2020 | 2020-10-01 | NA |
| England/QEUH-A2045F/2020 | 2020-10-01 | NA |
| England/NORT-1B6C0B9/2020 | 2020-10-01 | NA |
| England/MILK-A05D55/2020 | 2020-10-01 | NA |
| England/QEUH-A21290/2020 | 2020-10-01 | NA |
| England/QEUH-A1EE0B/2020 | 2020-10-01 | NA |
| England/QEUH-A1EE83/2020 | 2020-10-01 | NA |
| England/QEUH-A22341/2020 | 2020-10-01 | NA |
| England/QEUH-A1E894/2020 | 2020-10-01 | NA |
| England/QEUH-A209E7/2020 | 2020-10-01 | NA |
| England/MILK-A1D2B5/2020 | 2020-10-01 | NA |
| England/QEUH-A1EE47/2020 | 2020-10-01 | NA |
| England/ALDP-9FC8F7/2020 | 2020-10-01 | NA |
| England/MILK-A17EC6/2020 | 2020-10-01 | NA |
| England/NORT-1B6C09B/2020 | 2020-10-01 | NA |
| England/NORT-2863F8/2020 | 2020-10-01 | NA |
| England/NORT-1B6C08C/2020 | 2020-10-01 | NA |
| England/QEUH-A2239C/2020 | 2020-10-01 | NA |
| England/MILK-A192F5/2020 | 2020-10-01 | NA |
| England/QEUH-A2055C/2020 | 2020-10-01 | NA |
| England/QEUH-A02B6D/2020 | 2020-10-01 | NA |
| England/MILK-A060C4/2020 | 2020-10-01 | NA |
| England/QEUH-A1F480/2020 | 2020-10-01 | NA |
| England/ALDP-9FCEAA/2020 | 2020-10-01 | NA |
| England/QEUH-A0C222/2020 | 2020-10-01 | NA |
| England/ALDP-9FDEF4/2020 | 2020-10-01 | NA |
| England/ALDP-9FCAB5/2020 | 2020-10-01 | NA |
| England/QEUH-A0AEF3/2020 | 2020-10-01 | NA |
| England/QEUH-A0C398/2020 | 2020-10-01 | NA |
| England/ALDP-9FCA88/2020 | 2020-10-01 | NA |
| England/QEUH-A0C1F8/2020 | 2020-10-01 | NA |
| England/ALDP-9FDE9A/2020 | 2020-10-01 | NA |
| Wales/PHWC-16EA10/2020 | 2020-10-01 | NA |
| England/QEUH-A0C3D4/2020 | 2020-10-01 | NA |
| England/QEUH-A1F4F9/2020 | 2020-10-01 | NA |
| England/QEUH-A1F9DC/2020 | 2020-10-01 | NA |
| England/ALDP-9FD6A1/2020 | 2020-10-01 | NA |
| England/QEUH-A0CADF/2020 | 2020-10-01 | NA |
| England/QEUH-A0B0B0/2020 | 2020-10-01 | NA |
| England/MILK-A0670B/2020 | 2020-10-01 | NA |
| England/MILK-A17F2D/2020 | 2020-10-01 | NA |
| England/QEUH-A21870/2020 | 2020-10-01 | NA |
| England/QEUH-A1FCE2/2020 | 2020-10-01 | NA |
| England/QEUH-A1DFEB/2020 | 2020-10-01 | NA |
| England/QEUH-A1F444/2020 | 2020-10-01 | NA |
| England/QEUH-A22332/2020 | 2020-10-01 | NA |
| England/QEUH-A2100F/2020 | 2020-10-01 | NA |
| England/ALDP-9FCB0D/2020 | 2020-10-01 | NA |
| England/NORT-1B6BFF9/2020 | 2020-10-01 | NA |
| Wales/PHWC-47D3B6/2020 | 2020-10-01 | NA |
| England/QEUH-A0AF5A/2020 | 2020-10-01 | NA |
| England/QEUH-A01B8C/2020 | 2020-10-01 | NA |
| Scotland/QEUH-A0076B/2020 | 2020-10-02 | NA |
| England/NORT-1B6C101/2020 | 2020-10-02 | NA |
| England/QEUH-A005F8/2020 | 2020-10-02 | NA |
| Scotland/QEUH-9FFF1D/2020 | 2020-10-02 | NA |
| England/QEUH-A0009D/2020 | 2020-10-02 | NA |
| England/ALDP-9FD1BF/2020 | 2020-10-02 | NA |
| England/QEUH-A00A17/2020 | 2020-10-02 | NA |
| England/QEUH-9FFBFB/2020 | 2020-10-02 | NA |
| England/NORT-28CD8B/2020 | 2020-10-02 | NA |
| England/QEUH-A00103/2020 | 2020-10-02 | NA |
| England/QEUH-A0007F/2020 | 2020-10-02 | NA |
| England/QEUH-A0031C/2020 | 2020-10-02 | NA |
| England/QEUH-9FF654/2020 | 2020-10-02 | NA |
| England/QEUH-9FF4E1/2020 | 2020-10-02 | NA |
| England/QEUH-9FFBDD/2020 | 2020-10-02 | NA |
| England/QEUH-9FF5B1/2020 | 2020-10-02 | NA |
| England/QEUH-A00464/2020 | 2020-10-02 | NA |
| England/NORT-28CE00/2020 | 2020-10-02 | NA |
| England/QEUH-A01C10/2020 | 2020-10-02 | NA |
| England/QEUH-9FFBBF/2020 | 2020-10-02 | NA |
| England/QEUH-9FF2AB/2020 | 2020-10-02 | NA |
| England/QEUH-9FFA2B/2020 | 2020-10-02 | NA |
| England/BRIS-13403F/2020 | 2020-10-02 | NA |
| England/QEUH-A000CA/2020 | 2020-10-02 | NA |
| England/QEUH-9FF1EA/2020 | 2020-10-02 | NA |
| England/QEUH-A000BB/2020 | 2020-10-02 | NA |
| England/QEUH-9FF117/2020 | 2020-10-02 | NA |
| England/ALDP-9FD340/2020 | 2020-10-02 | NA |
| England/ALDP-9FD058/2020 | 2020-10-02 | NA |
| England/ALDP-9FD243/2020 | 2020-10-02 | NA |
| England/ALDP-9FD261/2020 | 2020-10-02 | NA |
| England/QEUH-A01C2F/2020 | 2020-10-02 | NA |
| Scotland/CVR4961/2020 | 2020-10-02 | NA |
| England/QEUH-9FFFE0/2020 | 2020-10-02 | NA |
| England/QEUH-A00130/2020 | 2020-10-02 | NA |
| England/QEUH-A00006/2020 | 2020-10-02 | NA |
| England/BRIS-1346A6/2020 | 2020-10-02 | NA |
| England/BRIS-134011/2020 | 2020-10-02 | NA |
| England/BRIS-13454F/2020 | 2020-10-02 | NA |
| Scotland/QEUH-9FFED4/2020 | 2020-10-02 | NA |
| England/ALDP-9FD0A3/2020 | 2020-10-02 | NA |
| England/QEUH-A01C7A/2020 | 2020-10-02 | NA |
| England/QEUH-9FFF95/2020 | 2020-10-02 | NA |
| England/ALDP-9FD89C/2020 | 2020-10-02 | NA |
| England/ALDP-9FD850/2020 | 2020-10-02 | NA |
| Scotland/QEUH-A00DB4/2020 | 2020-10-02 | NA |
| Scotland/CVR4905/2020 | 2020-10-02 | NA |
| England/NOTT-113FA2/2020 | 2020-10-02 | NA |
| Wales/PHWC-16F129/2020 | 2020-10-03 | NA |
| Scotland/QEUH-A0BD9B/2020 | 2020-10-03 | NA |
| England/MILK-A19389/2020 | 2020-10-03 | NA |
| England/MILK-A1937A/2020 | 2020-10-03 | NA |
| England/QEUH-A0BA3A/2020 | 2020-10-03 | NA |
| Scotland/QEUH-A0ADD8/2020 | 2020-10-03 | NA |
| England/QEUH-A0C8D5/2020 | 2020-10-03 | NA |
| England/QEUH-A0C8B7/2020 | 2020-10-03 | NA |
| England/NORT-28CD6D/2020 | 2020-10-03 | NA |
| England/QEUH-A0ACDB/2020 | 2020-10-03 | NA |
| England/MILK-A1D6E6/2020 | 2020-10-03 | NA |
| England/NORT-28CEA6/2020 | 2020-10-03 | NA |
| England/MILK-A1D710/2020 | 2020-10-03 | NA |
| England/QEUH-A0C88A/2020 | 2020-10-03 | NA |
| England/MILK-A23A96/2020 | 2020-10-03 | NA |
| England/MILK-A22C28/2020 | 2020-10-03 | NA |
| England/QEUH-A0BF77/2020 | 2020-10-03 | NA |
| England/MILK-A22BA3/2020 | 2020-10-03 | NA |
| England/MILK-A1D21F/2020 | 2020-10-03 | NA |
| England/SHEF-C5720/2020 | 2020-10-03 | NA |
| England/MILK-A1B6E8/2020 | 2020-10-03 | NA |
| Scotland/QEUH-A0CAFD/2020 | 2020-10-03 | NA |
| England/QEUH-A0BA58/2020 | 2020-10-03 | NA |
| England/QEUH-A4BE41/2020 | 2020-10-03 | NA |
| England/QEUH-A0B584/2020 | 2020-10-03 | NA |
| England/MILK-A463CF/2020 | 2020-10-03 | NA |
| England/QEUH-A3335C/2020 | 2020-10-03 | NA |
| England/MILK-A23182/2020 | 2020-10-03 | NA |
| England/MILK-A231BF/2020 | 2020-10-03 | NA |
| England/ALDP-ABD299/2020 | 2020-10-04 | NA |
| England/LIVE-DA7AF1/2020 | 2020-10-04 | NA |
| Wales/PHWC-16F077/2020 | 2020-10-04 | NA |
| England/NORT-28CD9A/2020 | 2020-10-04 | NA |
| England/QEUH-A36C03/2020 | 2020-10-04 | NA |
| England/ALDP-ABD23F/2020 | 2020-10-04 | NA |
| England/MILK-A2400C/2020 | 2020-10-04 | NA |
| England/NORT-28648C/2020 | 2020-10-04 | NA |
| England/SHEF-CF28F/2020 | 2020-10-04 | NA |
| Wales/PHWC-16EF5D/2020 | 2020-10-04 | NA |
| England/QEUH-A370D9/2020 | 2020-10-04 | NA |
| England/QEUH-A33574/2020 | 2020-10-04 | NA |
| England/CAMC-A3E728/2020 | 2020-10-04 | NA |
| England/QEUH-A3136D/2020 | 2020-10-05 | NA |
| Wales/MILK-A47B99/2020 | 2020-10-05 | NA |
| Wales/ALDP-A2EEDB/2020 | 2020-10-05 | NA |
| England/MILK-A46444/2020 | 2020-10-05 | NA |
| England/MILK-A464AE/2020 | 2020-10-05 | NA |
| England/QEUH-A4D37D/2020 | 2020-10-05 | NA |
| England/ALDP-A2E6D3/2020 | 2020-10-05 | NA |
| England/MILK-A47B8A/2020 | 2020-10-05 | NA |
| Wales/MILK-A47B5D/2020 | 2020-10-05 | NA |
| England/CAMC-A3E8E9/2020 | 2020-10-05 | NA |
| England/ALDP-A2F317/2020 | 2020-10-05 | NA |
| England/ALDP-A2FC0D/2020 | 2020-10-05 | NA |
| England/ALDP-A2EAF5/2020 | 2020-10-05 | NA |
| England/CAMC-A422A8/2020 | 2020-10-05 | NA |
| England/QEUH-A317AD/2020 | 2020-10-05 | NA |
| England/CAMC-A3E4F4/2020 | 2020-10-05 | NA |
| England/ALDP-A2E758/2020 | 2020-10-05 | NA |
| England/NORT-28CFFE/2020 | 2020-10-05 | NA |
| England/NORT-28CDA9/2020 | 2020-10-05 | NA |
| England/NORT-28CD21/2020 | 2020-10-05 | NA |
| England/NORT-28CD12/2020 | 2020-10-05 | NA |
| England/NORT-2862DD/2020 | 2020-10-05 | NA |
| England/QEUH-A37AAE/2020 | 2020-10-05 | NA |
| England/QEUH-A37ADB/2020 | 2020-10-05 | NA |
| England/QEUH-A4DB2A/2020 | 2020-10-05 | NA |
| England/QEUH-A4CEB9/2020 | 2020-10-05 | NA |
| England/QEUH-A336CC/2020 | 2020-10-05 | NA |
| England/QEUH-A3707F/2020 | 2020-10-05 | NA |
| England/QEUH-A36D4C/2020 | 2020-10-05 | NA |
| England/CAMC-A41713/2020 | 2020-10-05 | NA |
| England/MILK-A468C0/2020 | 2020-10-05 | NA |
| England/ALDP-A2D3FB/2020 | 2020-10-05 | NA |
| England/QEUH-A31770/2020 | 2020-10-05 | NA |
| England/CAMC-A41494/2020 | 2020-10-05 | NA |
| England/CAMC-A41C8D/2020 | 2020-10-05 | NA |
| England/QEUH-A364A1/2020 | 2020-10-05 | NA |
| England/QEUH-A4DB66/2020 | 2020-10-05 | NA |
| England/QEUH-A33547/2020 | 2020-10-05 | NA |
| England/QEUH-A326BE/2020 | 2020-10-05 | NA |
| England/QEUH-A4B209/2020 | 2020-10-05 | NA |
| England/ALDP-A2F61E/2020 | 2020-10-05 | NA |
| England/ALDP-A2F5E4/2020 | 2020-10-05 | NA |
| England/ALDP-A2D3EC/2020 | 2020-10-05 | NA |
| Wales/PHWC-47CDF5/2020 | 2020-10-05 | NA |
| England/ALDP-A2D13D/2020 | 2020-10-05 | NA |
| England/ALDP-A2E80A/2020 | 2020-10-05 | NA |
| England/CAMC-A3E8F8/2020 | 2020-10-05 | NA |
| England/ALDP-A2ED38/2020 | 2020-10-05 | NA |
| England/ALDP-A2EC59/2020 | 2020-10-05 | NA |
| Wales/PHWC-47CE01/2020 | 2020-10-05 | NA |
| England/NORW-F06E3/2020 | 2020-10-05 | NA |
| England/QEUH-A37446/2020 | 2020-10-05 | NA |
| England/MILK-A466D5/2020 | 2020-10-05 | NA |
| England/MILK-A468B1/2020 | 2020-10-05 | NA |
| Wales/ALDP-A2EF23/2020 | 2020-10-05 | NA |
| England/ALDP-A2E61F/2020 | 2020-10-05 | NA |
| England/ALDP-A2D3A0/2020 | 2020-10-05 | NA |
| Scotland/QEUH-A3152B/2020 | 2020-10-05 | NA |
| Scotland/QEUH-A314F1/2020 | 2020-10-05 | NA |
| England/QEUH-A4B139/2020 | 2020-10-05 | NA |
| Wales/MILK-A477B3/2020 | 2020-10-05 | NA |
| England/MILK-A44F90/2020 | 2020-10-06 | NA |
| England/CAMC-A419D1/2020 | 2020-10-06 | NA |
| Wales/ALDP-A2FECB/2020 | 2020-10-06 | NA |
| England/MILK-A4615C/2020 | 2020-10-06 | NA |
| England/QEUH-A490A7/2020 | 2020-10-06 | NA |
| England/QEUH-A49098/2020 | 2020-10-06 | NA |
| England/CAMC-A40E4C/2020 | 2020-10-06 | NA |
| England/NORT-28D063/2020 | 2020-10-06 | NA |
| England/NORT-28D027/2020 | 2020-10-06 | NA |
| England/MILK-A46D58/2020 | 2020-10-06 | NA |
| England/MILK-A456E5/2020 | 2020-10-06 | NA |
| England/CAMC-A3E3F7/2020 | 2020-10-06 | NA |
| England/MILK-A46D85/2020 | 2020-10-06 | NA |
| England/MILK-A47FAC/2020 | 2020-10-06 | NA |
| England/MILK-A471B5/2020 | 2020-10-06 | NA |
| England/ALDP-A2E1D2/2020 | 2020-10-06 | NA |
| England/ALDP-A2DA7E/2020 | 2020-10-06 | NA |
| England/EXET-138226/2020 | 2020-10-06 | NA |
| England/MILK-A44E57/2020 | 2020-10-06 | NA |
| Wales/MILK-A45C01/2020 | 2020-10-06 | NA |
| England/CAMC-A3F596/2020 | 2020-10-06 | NA |
| England/CAMC-A419B3/2020 | 2020-10-06 | NA |
| England/CAMC-A3E4E5/2020 | 2020-10-06 | NA |
| England/ALDP-A2D768/2020 | 2020-10-06 | NA |
| England/ALDP-A2D944/2020 | 2020-10-06 | NA |
| England/ALDP-A2D461/2020 | 2020-10-06 | NA |
| England/QEUH-A3287C/2020 | 2020-10-06 | NA |
| England/CAMC-A41801/2020 | 2020-10-06 | NA |
| England/MILK-A46E37/2020 | 2020-10-06 | NA |
| England/ALDP-A2DF60/2020 | 2020-10-06 | NA |
| England/NORT-2A5EE3/2020 | 2020-10-06 | NA |
| England/NORT-2A5E4D/2020 | 2020-10-06 | NA |
| England/NORT-2A5E5C/2020 | 2020-10-06 | NA |
| England/NORT-2A5E6B/2020 | 2020-10-06 | NA |
| England/NORT-2A5EF2/2020 | 2020-10-06 | NA |
| England/NORT-2A5E89/2020 | 2020-10-06 | NA |
| England/ALDP-A514A0/2020 | 2020-10-06 | NA |
| England/ALDP-A2D829/2020 | 2020-10-06 | NA |
| England/MILK-A47F51/2020 | 2020-10-06 | NA |
| England/CAMC-A4194A/2020 | 2020-10-06 | NA |
| England/MILK-A469FA/2020 | 2020-10-06 | NA |
| England/MILK-A48002/2020 | 2020-10-06 | NA |
| England/MILK-A45EFC/2020 | 2020-10-06 | NA |
| England/QEUH-A49942/2020 | 2020-10-06 | NA |
| England/MILK-A44D69/2020 | 2020-10-06 | NA |
| England/MILK-A47391/2020 | 2020-10-06 | NA |
| England/MILK-A47373/2020 | 2020-10-06 | NA |
| England/MILK-A47364/2020 | 2020-10-06 | NA |
| England/CAMC-A3F4E4/2020 | 2020-10-06 | NA |
| England/ALDP-A2D908/2020 | 2020-10-06 | NA |
| England/MILK-A45F26/2020 | 2020-10-06 | NA |
| England/ALDP-A2D4AD/2020 | 2020-10-06 | NA |
| England/ALDP-A2F3CC/2020 | 2020-10-06 | NA |
| England/ALDP-A2D689/2020 | 2020-10-06 | NA |
| England/NOTT-114642/2020 | 2020-10-06 | NA |
| Wales/PHWC-47D2D7/2020 | 2020-10-07 | NA |
| England/ALDP-A510D9/2020 | 2020-10-07 | NA |
| England/ALDP-A5213F/2020 | 2020-10-07 | NA |
| Wales/PHWC-480839/2020 | 2020-10-07 | NA |
| Wales/ALDP-A4FF2C/2020 | 2020-10-07 | NA |
| England/NORT-28D045/2020 | 2020-10-07 | NA |
| England/NORT-2A5F0E/2020 | 2020-10-07 | NA |
| England/CAMC-A585A6/2020 | 2020-10-07 | NA |
| England/CAMC-A585B5/2020 | 2020-10-07 | NA |
| England/CAMC-A585D3/2020 | 2020-10-07 | NA |
| England/CAMC-A585E2/2020 | 2020-10-07 | NA |
| England/CAMC-A5860D/2020 | 2020-10-07 | NA |
| England/CAMC-A585C4/2020 | 2020-10-07 | NA |
| England/ALDP-A51FAF/2020 | 2020-10-07 | NA |
| England/ALDP-A51FBE/2020 | 2020-10-07 | NA |
| England/ALDP-A4FCDA/2020 | 2020-10-07 | NA |
| England/ALDP-A51FCD/2020 | 2020-10-07 | NA |
| England/CAMC-A585F1/2020 | 2020-10-07 | NA |
| England/MILK-A44A62/2020 | 2020-10-07 | NA |
| England/MILK-A44B14/2020 | 2020-10-07 | NA |
| England/MILK-A44B23/2020 | 2020-10-07 | NA |
| England/QEUH-A32302/2020 | 2020-10-07 | NA |
| England/CAMC-A58A7A/2020 | 2020-10-07 | NA |
| England/ALDP-A4FA67/2020 | 2020-10-07 | NA |
| Wales/ALDP-A51B9C/2020 | 2020-10-07 | NA |
| England/ALDP-A50D3D/2020 | 2020-10-07 | NA |
| England/ALDP-A511F4/2020 | 2020-10-07 | NA |
| England/ALDP-A51516/2020 | 2020-10-07 | NA |
| England/ALDP-A4FBFB/2020 | 2020-10-07 | NA |
| England/ALDP-A517B6/2020 | 2020-10-07 | NA |
| England/ALDP-A4F9D3/2020 | 2020-10-07 | NA |
| England/ALDP-A51297/2020 | 2020-10-07 | NA |
| England/ALDP-A514CE/2020 | 2020-10-07 | NA |
| England/ALDP-A51534/2020 | 2020-10-07 | NA |
| England/ALDP-A501C8/2020 | 2020-10-07 | NA |
| England/SHEF-C6CA8/2020 | 2020-10-07 | NA |
| Scotland/QEUH-A4C5D2/2020 | 2020-10-07 | NA |
| England/ALDP-A515DA/2020 | 2020-10-07 | NA |
| England/ALDP-A51DD2/2020 | 2020-10-07 | NA |
| England/ALDP-A51F27/2020 | 2020-10-07 | NA |
| England/ALDP-A50D88/2020 | 2020-10-07 | NA |
| England/ALDP-A50D97/2020 | 2020-10-07 | NA |
| England/NORT-2A5F2C/2020 | 2020-10-07 | NA |
| England/CAMC-A41704/2020 | 2020-10-07 | NA |
| England/ALDP-A4FC52/2020 | 2020-10-07 | NA |
| England/ALDP-A5281C/2020 | 2020-10-07 | NA |
| England/ALDP-A500F8/2020 | 2020-10-07 | NA |
| England/ALDP-A5173E/2020 | 2020-10-07 | NA |
| England/NORW-F04BC/2020 | 2020-10-07 | NA |
| England/ALDP-A51E93/2020 | 2020-10-07 | NA |
| Scotland/QEUH-A376C8/2020 | 2020-10-07 | NA |
| England/CAMC-A3FA79/2020 | 2020-10-07 | NA |
| England/ALDP-A4FD04/2020 | 2020-10-07 | NA |
| England/ALDP-A500E9/2020 | 2020-10-07 | NA |
| England/ALDP-A522C3/2020 | 2020-10-07 | NA |
| England/ALDP-A4FCBC/2020 | 2020-10-07 | NA |
| England/ALDP-A4FCAD/2020 | 2020-10-07 | NA |
| England/QEUH-A326AF/2020 | 2020-10-07 | NA |
| England/ALDP-A501E6/2020 | 2020-10-07 | NA |
| England/ALDP-A527B5/2020 | 2020-10-07 | NA |
| England/ALDP-A51200/2020 | 2020-10-07 | NA |
| England/ALDP-A516D7/2020 | 2020-10-07 | NA |
| England/QEUH-A4C6A2/2020 | 2020-10-07 | NA |
| England/CAMC-A412F4/2020 | 2020-10-07 | NA |
| England/ALDP-A524DC/2020 | 2020-10-07 | NA |
| England/ALDP-A5183B/2020 | 2020-10-07 | NA |
| England/ALDP-A4FFB3/2020 | 2020-10-07 | NA |
| England/ALDP-A5184A/2020 | 2020-10-07 | NA |
| England/NORT-1B6C14D/2020 | 2020-10-07 | NA |
| England/ALDP-A4FBCE/2020 | 2020-10-07 | NA |
| England/BRIS-134C2C/2020 | 2020-10-08 | NA |
| England/CAMC-A57B96/2020 | 2020-10-08 | NA |
| England/CAMB-1B6ABF/2020 | 2020-10-08 | NA |
| England/CAMC-A58175/2020 | 2020-10-08 | NA |
| England/QEUH-A49A8B/2020 | 2020-10-08 | NA |
| England/NORT-2867DE/2020 | 2020-10-08 | NA |
| England/NORT-2A7F1B/2020 | 2020-10-08 | NA |
| England/CAMC-A57DFA/2020 | 2020-10-08 | NA |
| England/CAMC-A57B2D/2020 | 2020-10-08 | NA |
| England/SHEF-D09FF/2020 | 2020-10-08 | NA |
| England/CAMC-A57CC0/2020 | 2020-10-08 | NA |
| England/CAMC-A59235/2020 | 2020-10-08 | NA |
| England/BRIS-134E08/2020 | 2020-10-08 | NA |
| England/BRIS-134DFC/2020 | 2020-10-08 | NA |
| England/CAMC-A57404/2020 | 2020-10-08 | NA |
| England/CAMC-A5899B/2020 | 2020-10-08 | NA |
| England/CAMC-A5898C/2020 | 2020-10-08 | NA |
| England/CAMC-A57F21/2020 | 2020-10-08 | NA |
| England/CAMC-A57422/2020 | 2020-10-08 | NA |
| England/CAMC-A57413/2020 | 2020-10-08 | NA |
| England/NOTT-1147C7/2020 | 2020-10-08 | NA |
| Wales/PHWC-47D86C/2020 | 2020-10-08 | NA |
| Scotland/QEUH-A4B2AF/2020 | 2020-10-08 | NA |
| England/MILK-A6B230/2020 | 2020-10-08 | NA |
| England/NORT-2A7E0F/2020 | 2020-10-08 | NA |
| England/NORT-2A7E1E/2020 | 2020-10-08 | NA |
| England/CAMC-A593BA/2020 | 2020-10-08 | NA |
| England/CAMC-A5902C/2020 | 2020-10-08 | NA |
| England/CAMC-A5747D/2020 | 2020-10-08 | NA |
| England/QEUH-A49AC7/2020 | 2020-10-08 | NA |
| England/BRIS-1349DA/2020 | 2020-10-08 | NA |
| England/BRIS-134D83/2020 | 2020-10-08 | NA |
| England/BRIS-134C1D/2020 | 2020-10-08 | NA |
| England/BRIS-134C68/2020 | 2020-10-08 | NA |
| England/SHEF-C2495/2020 | 2020-10-08 | NA |
| Scotland/QEUH-A4B825/2020 | 2020-10-08 | NA |
| England/CAMC-A596A2/2020 | 2020-10-08 | NA |
| England/MILK-A6A1BC/2020 | 2020-10-08 | NA |
| England/MILK-A6A2B9/2020 | 2020-10-08 | NA |
| Scotland/QEUH-A48EBD/2020 | 2020-10-08 | NA |
| England/MILK-A6BAFC/2020 | 2020-10-09 | NA |
| Wales/CAMC-A58E9C/2020 | 2020-10-09 | NA |
| England/CAMC-A5738F/2020 | 2020-10-09 | NA |
| England/MILK-A6B1CA/2020 | 2020-10-09 | NA |
| England/CAMC-A64774/2020 | 2020-10-09 | NA |
| England/CAMC-A581C0/2020 | 2020-10-09 | NA |
| England/MILK-A6A32F/2020 | 2020-10-09 | NA |
| England/CAMC-A5711C/2020 | 2020-10-09 | NA |
| England/MILK-A6BD7B/2020 | 2020-10-09 | NA |
| England/EXET-1382AE/2020 | 2020-10-09 | NA |
| Wales/MILK-A6AC60/2020 | 2020-10-09 | NA |
| England/CAMC-A594F3/2020 | 2020-10-09 | NA |
| England/CAMC-A65351/2020 | 2020-10-09 | NA |
| England/NORT-2A7DE4/2020 | 2020-10-09 | NA |
| England/CAMC-A58148/2020 | 2020-10-09 | NA |
| England/CAMC-A6463B/2020 | 2020-10-09 | NA |
| England/CAMC-A64AF3/2020 | 2020-10-09 | NA |
| England/CAMC-A58AE3/2020 | 2020-10-09 | NA |
| England/NORT-2A7EF0/2020 | 2020-10-09 | NA |
| England/CAMC-A574C8/2020 | 2020-10-09 | NA |
| England/QEUH-A5DE88/2020 | 2020-10-09 | NA |
| England/CAMC-A586FE/2020 | 2020-10-09 | NA |
| England/PHEC-148AC1/2020 | 2020-10-09 | NA |
| England/CAMC-A59C28/2020 | 2020-10-09 | NA |
| England/LIVE-1E08B2/2020 | 2020-10-09 | NA |
| England/MILK-A69CAD/2020 | 2020-10-09 | NA |
| England/CAMC-A823D6/2020 | 2020-10-10 | NA |
| England/CAMC-A822D9/2020 | 2020-10-10 | NA |
| England/NORT-2865C5/2020 | 2020-10-10 | NA |
| England/QEUH-A61E90/2020 | 2020-10-10 | NA |
| England/ALDP-AC12A1/2020 | 2020-10-10 | NA |
| England/ALDP-AC1256/2020 | 2020-10-10 | NA |
| England/ALDP-AC11D1/2020 | 2020-10-10 | NA |
| England/NORT-2A7FEE/2020 | 2020-10-10 | NA |
| England/CAMC-A646D1/2020 | 2020-10-10 | NA |
| England/QEUH-A767AC/2020 | 2020-10-10 | NA |
| England/CAMC-A646FF/2020 | 2020-10-10 | NA |
| Wales/PHWC-47DFA3/2020 | 2020-10-10 | NA |
| England/PORT-2F578F/2020 | 2020-10-10 | NA |
| England/QEUH-A60329/2020 | 2020-10-10 | NA |
| England/QEUH-A6210F/2020 | 2020-10-11 | NA |
| England/CAMC-A647FC/2020 | 2020-10-11 | NA |
| England/CAMC-A657CE/2020 | 2020-10-11 | NA |
| England/CAMC-A82C26/2020 | 2020-10-11 | NA |
| England/CAMC-A6546D/2020 | 2020-10-11 | NA |
| England/CAMC-A64D36/2020 | 2020-10-11 | NA |
| England/CAMC-A65281/2020 | 2020-10-11 | NA |
| England/CAMC-A66675/2020 | 2020-10-11 | NA |
| England/CAMC-A65F3F/2020 | 2020-10-11 | NA |
| England/CAMC-A662AE/2020 | 2020-10-11 | NA |
| England/QEUH-A5DD8B/2020 | 2020-10-11 | NA |
| England/CAMC-A65579/2020 | 2020-10-11 | NA |
| England/SHEF-C4E86/2020 | 2020-10-11 | NA |
| England/CAMC-A64DAF/2020 | 2020-10-11 | NA |
| England/CAMC-A829F2/2020 | 2020-10-11 | NA |
| England/CAMC-A64BC3/2020 | 2020-10-11 | NA |
| England/CAMC-A662CC/2020 | 2020-10-11 | NA |
| England/CAMC-A662BD/2020 | 2020-10-11 | NA |
| England/QEUH-A643F8/2020 | 2020-10-11 | NA |
| England/CAMC-A651C0/2020 | 2020-10-11 | NA |
| England/CAMC-A651B1/2020 | 2020-10-11 | NA |
| England/PORT-2F57DA/2020 | 2020-10-11 | NA |
| England/CAMC-A65333/2020 | 2020-10-11 | NA |
| Northern_Ireland/NIRE-1089EE/2020 | 2020-10-11 | NA |
| Northern_Ireland/NIRE-1089C0/2020 | 2020-10-11 | NA |
| England/CAMC-A64817/2020 | 2020-10-11 | NA |
| England/CAMC-A65737/2020 | 2020-10-11 | NA |
| England/CAMC-A65719/2020 | 2020-10-11 | NA |
| England/CAMC-A6560D/2020 | 2020-10-11 | NA |
| England/CAMC-A65940/2020 | 2020-10-11 | NA |
| England/QEUH-A794DF/2020 | 2020-10-12 | NA |
| England/QEUH-A61953/2020 | 2020-10-12 | NA |
| England/QEUH-A6162F/2020 | 2020-10-12 | NA |
| England/CAMC-A8241F/2020 | 2020-10-12 | NA |
| England/ALDP-A684F1/2020 | 2020-10-12 | NA |
| England/QEUH-A5DEC4/2020 | 2020-10-12 | NA |
| England/ALDP-A67717/2020 | 2020-10-12 | NA |
| England/ALDP-A68664/2020 | 2020-10-12 | NA |
| England/CAMC-A82303/2020 | 2020-10-12 | NA |
| England/CAMC-A82989/2020 | 2020-10-12 | NA |
| Wales/QEUH-A76BEC/2020 | 2020-10-12 | NA |
| England/ALDP-A6737D/2020 | 2020-10-12 | NA |
| England/QEUH-A6206C/2020 | 2020-10-12 | NA |
| England/ALDP-A684E2/2020 | 2020-10-12 | NA |
| England/ALDP-A67119/2020 | 2020-10-12 | NA |
| England/PORT-2F5804/2020 | 2020-10-12 | NA |
| England/ALDP-A673B9/2020 | 2020-10-12 | NA |
| England/QEUH-A60365/2020 | 2020-10-12 | NA |
| England/QEUH-A643CB/2020 | 2020-10-12 | NA |
| Denmark/DCGC-7597/2020 | 2020-10-12 | NA |
| England/ALDP-A6802A/2020 | 2020-10-12 | NA |
| England/ALDP-A68691/2020 | 2020-10-12 | NA |
| England/ALDP-A675FF/2020 | 2020-10-12 | NA |
| England/ALDP-A692AB/2020 | 2020-10-12 | NA |
| England/ALDP-A670C1/2020 | 2020-10-12 | NA |
| England/QEUH-A61E36/2020 | 2020-10-12 | NA |
| England/CAMC-A829B6/2020 | 2020-10-12 | NA |
| England/ALDP-A6852B/2020 | 2020-10-12 | NA |
| England/ALDP-A6851C/2020 | 2020-10-12 | NA |
| England/ALDP-A68488/2020 | 2020-10-12 | NA |
| England/QEUH-A78D4E/2020 | 2020-10-12 | NA |
| England/CAMC-A82AB3/2020 | 2020-10-12 | NA |
| England/ALDP-A68D7E/2020 | 2020-10-12 | NA |
| England/QEUH-A5E1BB/2020 | 2020-10-12 | NA |
| England/ALDP-A68BA1/2020 | 2020-10-12 | NA |
| England/QEUH-A5E072/2020 | 2020-10-12 | NA |
| England/CAMC-A81304/2020 | 2020-10-12 | NA |
| England/CAMC-A82B0B/2020 | 2020-10-12 | NA |
| England/CAMC-A82BFC/2020 | 2020-10-12 | NA |
| England/MILK-A7CF9F/2020 | 2020-10-12 | NA |
| England/ALDP-A67F2E/2020 | 2020-10-12 | NA |
| England/QEUH-A63AB8/2020 | 2020-10-12 | NA |
| England/ALDP-A67FA6/2020 | 2020-10-12 | NA |
| England/ALDP-A67999/2020 | 2020-10-12 | NA |
| England/ALDP-A66AC4/2020 | 2020-10-12 | NA |
| Wales/PHWC-481DC0/2020 | 2020-10-12 | NA |
| England/QEUH-A6211E/2020 | 2020-10-12 | NA |
| England/ALDP-A68B29/2020 | 2020-10-12 | NA |
| England/QEUH-A624F7/2020 | 2020-10-12 | NA |
| England/ALDP-A69108/2020 | 2020-10-12 | NA |
| England/ALDP-A68E3F/2020 | 2020-10-12 | NA |
| England/QEUH-A61EEB/2020 | 2020-10-12 | NA |
| England/PORT-2F50C0/2020 | 2020-10-13 | NA |
| England/CAMC-A82CEA/2020 | 2020-10-13 | NA |
| England/MILK-A7DF34/2020 | 2020-10-13 | NA |
| England/MILK-A7DECE/2020 | 2020-10-13 | NA |
| England/QEUH-A61A50/2020 | 2020-10-13 | NA |
| England/MILK-A7D23B/2020 | 2020-10-13 | NA |
| England/QEUH-A616F2/2020 | 2020-10-13 | NA |
| England/QEUH-A61777/2020 | 2020-10-13 | NA |
| England/WSFT-25C5224/2020 | 2020-10-13 | NA |
| England/WSFT-25C4DAC/2020 | 2020-10-13 | NA |
| England/ALDP-A6919F/2020 | 2020-10-13 | NA |
| England/ALDP-A691EA/2020 | 2020-10-13 | NA |
| England/MILK-A7CE56/2020 | 2020-10-13 | NA |
| England/WSFT-25C53C7/2020 | 2020-10-13 | NA |
| England/QEUH-A61A05/2020 | 2020-10-13 | NA |
| England/QEUH-A60857/2020 | 2020-10-13 | NA |
| England/QEUH-A60945/2020 | 2020-10-13 | NA |
| Northern_Ireland/NIRE-109561/2020 | 2020-10-13 | NA |
| England/MILK-A7DABB/2020 | 2020-10-13 | NA |
| England/CAMC-A81C63/2020 | 2020-10-13 | NA |
| Wales/PHWC-47EFA2/2020 | 2020-10-13 | NA |
| England/QEUH-A78300/2020 | 2020-10-14 | NA |
| England/NORT-1B6111B/2020 | 2020-10-14 | NA |
| Wales/PHWC-47F23F/2020 | 2020-10-14 | NA |
| England/QEUH-A77BFA/2020 | 2020-10-14 | NA |
| England/EXET-138A5B/2020 | 2020-10-14 | NA |
| England/ALDP-A6DED0/2020 | 2020-10-14 | NA |
| England/CAMC-A8102B/2020 | 2020-10-14 | NA |
| England/CAMC-A81E6D/2020 | 2020-10-14 | NA |
| Wales/PHWC-49105B/2020 | 2020-10-14 | NA |
| England/QEUH-A78036/2020 | 2020-10-14 | NA |
| England/ALDP-A6D1E6/2020 | 2020-10-14 | NA |
| England/ALDP-AC5D06/2020 | 2020-10-14 | NA |
| England/ALDP-A6DB24/2020 | 2020-10-14 | NA |
| England/MILK-A7B3D0/2020 | 2020-10-14 | NA |
| England/ALDP-A6CF0B/2020 | 2020-10-14 | NA |
| England/ALDP-A6E130/2020 | 2020-10-14 | NA |
| England/ALDP-A6CB16/2020 | 2020-10-14 | NA |
| Northern_Ireland/NIRE-2341D1/2020 | 2020-10-14 | NA |
| England/ALDP-A6DEEF/2020 | 2020-10-14 | NA |
| England/ALDP-A6FD59/2020 | 2020-10-14 | NA |
| England/ALDP-A6DFBF/2020 | 2020-10-14 | NA |
| England/ALDP-A6CF56/2020 | 2020-10-14 | NA |
| England/ALDP-A6DB51/2020 | 2020-10-14 | NA |
| England/ALDP-A6D2D4/2020 | 2020-10-14 | NA |
| England/ALDP-A6DB15/2020 | 2020-10-14 | NA |
| England/ALDP-A6CC31/2020 | 2020-10-14 | NA |
| England/ALDP-A6CC40/2020 | 2020-10-14 | NA |
| England/ALDP-A6F4BE/2020 | 2020-10-14 | NA |
| England/MILK-A7B419/2020 | 2020-10-14 | NA |
| England/QEUH-A77BDC/2020 | 2020-10-14 | NA |
| England/ALDP-A6D553/2020 | 2020-10-14 | NA |
| England/ALDP-A6DFDD/2020 | 2020-10-14 | NA |
| England/ALDP-A6D887/2020 | 2020-10-14 | NA |
| England/ALDP-A6C7F4/2020 | 2020-10-14 | NA |
| England/NORT-1B61166/2020 | 2020-10-15 | NA |
| England/ALDP-A6F409/2020 | 2020-10-15 | NA |
| England/QEUH-A8AA7F/2020 | 2020-10-15 | NA |
| Germany/SL-SU-10451375/2020 | 2020-10-15 | NA |
| Wales/PHWC-481C96/2020 | 2020-10-15 | NA |
| England/ALDP-A6CA91/2020 | 2020-10-15 | NA |
| England/ALDP-A6EFDC/2020 | 2020-10-15 | NA |
| England/ALDP-A6C642/2020 | 2020-10-15 | NA |
| England/QEUH-A8A9FA/2020 | 2020-10-15 | NA |
| England/MILK-A7E0A9/2020 | 2020-10-15 | NA |
| England/MILK-A914FF/2020 | 2020-10-15 | NA |
| England/MILK-A7B90B/2020 | 2020-10-15 | NA |
| England/MILK-A7B938/2020 | 2020-10-15 | NA |
| England/MILK-A7BF63/2020 | 2020-10-15 | NA |
| England/MILK-A7BFFA/2020 | 2020-10-15 | NA |
| England/MILK-A7BA26/2020 | 2020-10-15 | NA |
| England/EXET-138A2E/2020 | 2020-10-15 | NA |
| England/ALDP-A6C67F/2020 | 2020-10-15 | NA |
| England/QEUH-A8B3BF/2020 | 2020-10-15 | NA |
| England/EXET-138772/2020 | 2020-10-15 | NA |
| England/EXET-138790/2020 | 2020-10-15 | NA |
| England/ALDP-A7047E/2020 | 2020-10-15 | NA |
| England/MILK-A79FA1/2020 | 2020-10-15 | NA |
| England/ALDP-A6E66E/2020 | 2020-10-15 | NA |
| England/ALDP-A6EC8A/2020 | 2020-10-15 | NA |
| England/ALDP-A705B7/2020 | 2020-10-15 | NA |
| England/ALDP-A6ED69/2020 | 2020-10-15 | NA |
| England/ALDP-A7053F/2020 | 2020-10-15 | NA |
| England/ALDP-A6E5AD/2020 | 2020-10-15 | NA |
| England/ALDP-A6EB9C/2020 | 2020-10-15 | NA |
| England/ALDP-A6E437/2020 | 2020-10-15 | NA |
| England/ALDP-A6E482/2020 | 2020-10-15 | NA |
| England/NORT-1B61157/2020 | 2020-10-15 | NA |
| England/NORT-286AE4/2020 | 2020-10-15 | NA |
| England/ALDP-A6C5AF/2020 | 2020-10-15 | NA |
| England/NORT-286AD5/2020 | 2020-10-15 | NA |
| England/NORT-286AF3/2020 | 2020-10-15 | NA |
| England/MILK-A7B561/2020 | 2020-10-15 | NA |
| England/MILK-A7C6D6/2020 | 2020-10-15 | NA |
| England/MILK-A7B525/2020 | 2020-10-15 | NA |
| England/MILK-A7B570/2020 | 2020-10-15 | NA |
| England/MILK-A7A720/2020 | 2020-10-15 | NA |
| England/ALDP-A6C8D3/2020 | 2020-10-15 | NA |
| England/MILK-A7BDF0/2020 | 2020-10-15 | NA |
| England/MILK-A7BD5A/2020 | 2020-10-15 | NA |
| England/QEUH-A8C785/2020 | 2020-10-15 | NA |
| England/ALDP-A6D377/2020 | 2020-10-15 | NA |
| England/ALDP-A6D33B/2020 | 2020-10-15 | NA |
| England/MILK-A7A939/2020 | 2020-10-15 | NA |
| England/MILK-A7A2F2/2020 | 2020-10-15 | NA |
| England/ALDP-A6E4CE/2020 | 2020-10-15 | NA |
| England/MILK-A7A122/2020 | 2020-10-15 | NA |
| England/ALDP-A6EC02/2020 | 2020-10-15 | NA |
| England/MILK-A7AD6A/2020 | 2020-10-15 | NA |
| England/MILK-A7ADB5/2020 | 2020-10-15 | NA |
| England/ALDP-A6D74E/2020 | 2020-10-15 | NA |
| England/ALDP-A6FB31/2020 | 2020-10-15 | NA |
| England/MILK-A7C0D8/2020 | 2020-10-15 | NA |
| England/ALDP-A6D702/2020 | 2020-10-15 | NA |
| England/ALDP-A6F68B/2020 | 2020-10-15 | NA |
| England/MILK-A7CB7D/2020 | 2020-10-15 | NA |
| England/ALDP-A6FABC/2020 | 2020-10-15 | NA |
| England/ALDP-A6E59E/2020 | 2020-10-15 | NA |
| England/ALDP-A6E4DD/2020 | 2020-10-15 | NA |
| England/ALDP-A6E561/2020 | 2020-10-15 | NA |
| Australia/NSW2939/2020 | 2020-10-16 | NA |
| Wales/PHWC-4813DD/2020 | 2020-10-16 | NA |
| Wales/PHWC-4810B8/2020 | 2020-10-16 | NA |
| Wales/PHWC-481C1E/2020 | 2020-10-16 | NA |
| Wales/PHWC-481C78/2020 | 2020-10-16 | NA |
| England/MILK-A8EEF7/2020 | 2020-10-16 | NA |
| England/MILK-A9150B/2020 | 2020-10-16 | NA |
| England/QEUH-A8C13C/2020 | 2020-10-16 | NA |
| England/ALDP-A86C40/2020 | 2020-10-16 | NA |
| England/QEUH-A89CC5/2020 | 2020-10-16 | NA |
| Wales/PHWC-4841EF/2020 | 2020-10-16 | NA |
| England/QEUH-A8BB20/2020 | 2020-10-16 | NA |
| England/MILK-A7C9BF/2020 | 2020-10-16 | NA |
| England/QEUH-A8B5D7/2020 | 2020-10-16 | NA |
| England/ALDP-A879C0/2020 | 2020-10-16 | NA |
| England/ALDP-A850EB/2020 | 2020-10-16 | NA |
| England/QEUH-A8AABB/2020 | 2020-10-16 | NA |
| England/QEUH-A8B285/2020 | 2020-10-16 | NA |
| England/QEUH-A8AA9D/2020 | 2020-10-16 | NA |
| England/SHEF-C1AD0/2020 | 2020-10-16 | NA |
| Wales/PHWC-48181A/2020 | 2020-10-16 | NA |
| England/MILK-A7C7E2/2020 | 2020-10-16 | NA |
| England/MILK-A7CC89/2020 | 2020-10-16 | NA |
| England/QEUH-A8CA13/2020 | 2020-10-16 | NA |
| England/QEUH-A8C891/2020 | 2020-10-16 | NA |
| England/ALDP-A84FFE/2020 | 2020-10-16 | NA |
| England/QEUH-A8A981/2020 | 2020-10-16 | NA |
| England/ALDP-A8792A/2020 | 2020-10-17 | NA |
| England/ALDP-A86633/2020 | 2020-10-17 | NA |
| England/ALDP-A85BF9/2020 | 2020-10-17 | NA |
| England/ALDP-A874ED/2020 | 2020-10-17 | NA |
| England/ALDP-A85C14/2020 | 2020-10-17 | NA |
| England/ALDP-A85C41/2020 | 2020-10-17 | NA |
| England/QEUH-A8C855/2020 | 2020-10-17 | NA |
| England/ALDP-A872B6/2020 | 2020-10-17 | NA |
| England/ALDP-A85B08/2020 | 2020-10-17 | NA |
| England/ALDP-A85A47/2020 | 2020-10-17 | NA |
| England/ALDP-A85AB0/2020 | 2020-10-17 | NA |
| England/ALDP-A8624E/2020 | 2020-10-17 | NA |
| Wales/ALDP-A85643/2020 | 2020-10-17 | NA |
| Wales/ALDP-A862D5/2020 | 2020-10-17 | NA |
| Wales/ALDP-A8640C/2020 | 2020-10-17 | NA |
| England/ALDP-A858C5/2020 | 2020-10-17 | NA |
| England/ALDP-A85801/2020 | 2020-10-17 | NA |
| England/ALDP-A858E3/2020 | 2020-10-17 | NA |
| England/ALDP-A8583E/2020 | 2020-10-17 | NA |
| England/ALDP-A858B6/2020 | 2020-10-17 | NA |
| England/ALDP-A858D4/2020 | 2020-10-17 | NA |
| England/ALDP-A84DF4/2020 | 2020-10-17 | NA |
| Wales/PHWC-481980/2020 | 2020-10-17 | NA |
| England/QEUH-A8C293/2020 | 2020-10-17 | NA |
| England/ALDP-A85CE7/2020 | 2020-10-17 | NA |
| England/NORT-286C39/2020 | 2020-10-17 | NA |
| England/NORT-286C48/2020 | 2020-10-17 | NA |
| England/ALDP-A85CAB/2020 | 2020-10-17 | NA |
| England/ALDP-A871C8/2020 | 2020-10-17 | NA |
| Wales/PHWC-48156E/2020 | 2020-10-17 | NA |
| England/ALDP-A86123/2020 | 2020-10-17 | NA |
| Wales/ALDP-A87D3D/2020 | 2020-10-17 | NA |
| England/ALDP-A87D2E/2020 | 2020-10-17 | NA |
| England/ALDP-A87B7F/2020 | 2020-10-17 | NA |
| England/ALDP-A85F2A/2020 | 2020-10-17 | NA |
| England/LOND-D9B96/2020 | 2020-10-17 | NA |
| England/ALDP-A85A29/2020 | 2020-10-17 | NA |
| England/ALDP-A86FED/2020 | 2020-10-17 | NA |
| England/ALDP-A87052/2020 | 2020-10-17 | NA |
| England/ALDP-A84D9A/2020 | 2020-10-17 | NA |
| England/ALDP-A86545/2020 | 2020-10-17 | NA |
| Wales/PHWC-4845C5/2020 | 2020-10-18 | NA |
| England/QEUH-A8C600/2020 | 2020-10-18 | NA |
| England/ALDP-A862B7/2020 | 2020-10-18 | NA |
| England/QEUH-A8C6B5/2020 | 2020-10-18 | NA |
| England/QEUH-A8C62E/2020 | 2020-10-18 | NA |
| England/QEUH-A8C5F4/2020 | 2020-10-18 | NA |
| Scotland/EDB8490/2020 | 2020-10-18 | NA |
| Wales/PHWC-4817C2/2020 | 2020-10-18 | NA |
| England/QEUH-A8C318/2020 | 2020-10-18 | NA |
| England/QEUH-A8C372/2020 | 2020-10-18 | NA |
| England/QEUH-A8C406/2020 | 2020-10-18 | NA |
| England/QEUH-A8C48E/2020 | 2020-10-18 | NA |
| England/QEUH-A8C697/2020 | 2020-10-18 | NA |
| England/QEUH-A8C4D9/2020 | 2020-10-18 | NA |
| England/QEUH-A8C64C/2020 | 2020-10-18 | NA |
| England/QEUH-A8C5E5/2020 | 2020-10-18 | NA |
| England/QEUH-A8C71C/2020 | 2020-10-18 | NA |
| England/QEUH-A8C4E8/2020 | 2020-10-18 | NA |
| England/QEUH-A8C309/2020 | 2020-10-18 | NA |
| England/QEUH-A8C451/2020 | 2020-10-18 | NA |
| England/QEUH-A8C503/2020 | 2020-10-18 | NA |
| England/QEUH-A8C5C7/2020 | 2020-10-18 | NA |
| England/QEUH-A8C433/2020 | 2020-10-18 | NA |
| England/QEUH-A8C688/2020 | 2020-10-18 | NA |
| England/QEUH-A9F628/2020 | 2020-10-18 | NA |
| England/NOTT-115F64/2020 | 2020-10-18 | NA |
| England/ALDP-A85A1A/2020 | 2020-10-18 | NA |
| England/ALDP-A84FA3/2020 | 2020-10-18 | NA |
| England/ALDP-A85995/2020 | 2020-10-18 | NA |
| England/ALDP-A859FF/2020 | 2020-10-18 | NA |
| England/ALDP-A92643/2020 | 2020-10-19 | NA |
| England/ALDP-A93B8F/2020 | 2020-10-19 | NA |
| England/EXET-13860C/2020 | 2020-10-19 | NA |
| England/ALDP-A929A4/2020 | 2020-10-19 | NA |
| England/MILK-AB76EE/2020 | 2020-10-19 | NA |
| England/MILK-A91AC0/2020 | 2020-10-19 | NA |
| England/EXET-138A6A/2020 | 2020-10-19 | NA |
| Singapore/1109/2020 | 2020-10-19 | NA |
| England/EXET-1386FD/2020 | 2020-10-19 | NA |
| England/WSFT-25C56CE/2020 | 2020-10-19 | NA |
| England/ALDP-A92AB0/2020 | 2020-10-19 | NA |
| England/ALDP-A92379/2020 | 2020-10-19 | NA |
| England/ALDP-A93D10/2020 | 2020-10-19 | NA |
| England/QEUH-A9F96B/2020 | 2020-10-19 | NA |
| England/ALDP-A92661/2020 | 2020-10-19 | NA |
| England/QEUH-A9F1EB/2020 | 2020-10-19 | NA |
| England/QEUH-A9F251/2020 | 2020-10-19 | NA |
| England/ALDP-A929B3/2020 | 2020-10-19 | NA |
| England/QEUH-AA0545/2020 | 2020-10-19 | NA |
| England/QEUH-A9F242/2020 | 2020-10-19 | NA |
| England/QEUH-A9F95C/2020 | 2020-10-19 | NA |
| England/ALDP-A961D5/2020 | 2020-10-19 | NA |
| England/ALDP-A958A4/2020 | 2020-10-19 | NA |
| England/QEUH-A9FBED/2020 | 2020-10-19 | NA |
| Northern_Ireland/NIRE-109358/2020 | 2020-10-19 | NA |
| England/ALDP-A94896/2020 | 2020-10-19 | NA |
| England/MILK-A9185D/2020 | 2020-10-19 | NA |
| England/ALDP-A9282F/2020 | 2020-10-19 | NA |
| England/ALDP-A943D1/2020 | 2020-10-19 | NA |
| England/ALDP-A95A53/2020 | 2020-10-19 | NA |
| Wales/QEUH-AA0509/2020 | 2020-10-19 | NA |
| Wales/QEUH-A9F9A7/2020 | 2020-10-19 | NA |
| Wales/QEUH-A9FA95/2020 | 2020-10-19 | NA |
| England/QEUH-AA02B7/2020 | 2020-10-19 | NA |
| England/ALDP-A927C8/2020 | 2020-10-19 | NA |
| England/QEUH-AADB28/2020 | 2020-10-19 | NA |
| England/ALDP-A92670/2020 | 2020-10-19 | NA |
| England/ALDP-A92740/2020 | 2020-10-19 | NA |
| England/ALDP-A92731/2020 | 2020-10-19 | NA |
| Scotland/QEUH-A9EBC0/2020 | 2020-10-20 | NA |
| England/MILK-ACC0F7/2020 | 2020-10-20 | NA |
| England/QEUH-AAEB36/2020 | 2020-10-20 | NA |
| England/ALDP-A96032/2020 | 2020-10-20 | NA |
| England/QEUH-AA00AE/2020 | 2020-10-20 | NA |
| England/QEUH-A9D693/2020 | 2020-10-20 | NA |
| England/QEUH-A9EDD9/2020 | 2020-10-20 | NA |
| England/QEUH-A9E29E/2020 | 2020-10-20 | NA |
| England/QEUH-A9E243/2020 | 2020-10-20 | NA |
| England/QEUH-A9D217/2020 | 2020-10-20 | NA |
| England/QEUH-AAAF4D/2020 | 2020-10-20 | NA |
| England/QEUH-A9D781/2020 | 2020-10-20 | NA |
| England/QEUH-A9E8C9/2020 | 2020-10-20 | NA |
| England/QEUH-A9E182/2020 | 2020-10-20 | NA |
| England/QEUH-A9E8AB/2020 | 2020-10-20 | NA |
| England/QEUH-A9AE16/2020 | 2020-10-20 | NA |
| England/QEUH-A9D6C0/2020 | 2020-10-20 | NA |
| England/QEUH-A9E1FB/2020 | 2020-10-20 | NA |
| England/QEUH-A9D666/2020 | 2020-10-20 | NA |
| England/QEUH-A9D6B1/2020 | 2020-10-20 | NA |
| England/QEUH-A9E270/2020 | 2020-10-20 | NA |
| England/QEUH-A9D305/2020 | 2020-10-20 | NA |
| England/NORT-29C0CB/2020 | 2020-10-20 | NA |
| England/QEUH-AAD36C/2020 | 2020-10-20 | NA |
| England/ALDP-A94ABE/2020 | 2020-10-20 | NA |
| England/QEUH-AACD9C/2020 | 2020-10-20 | NA |
| England/QEUH-A9E629/2020 | 2020-10-20 | NA |
| Scotland/QEUH-A9D2EA/2020 | 2020-10-20 | NA |
| England/QEUH-A9E60B/2020 | 2020-10-20 | NA |
| England/QEUH-AADED4/2020 | 2020-10-20 | NA |
| Wales/PHWC-48212D/2020 | 2020-10-20 | NA |
| England/QEUH-A9D165/2020 | 2020-10-20 | NA |
| England/QEUH-A9E0C1/2020 | 2020-10-20 | NA |
| England/QEUH-A9E0FE/2020 | 2020-10-20 | NA |
| England/QEUH-A9D39C/2020 | 2020-10-20 | NA |
| England/QEUH-AAD399/2020 | 2020-10-20 | NA |
| England/QEUH-A9F154/2020 | 2020-10-20 | NA |
| England/QEUH-A9F6FB/2020 | 2020-10-20 | NA |
| England/QEUH-AA0114/2020 | 2020-10-20 | NA |
| England/QEUH-A9D6EE/2020 | 2020-10-20 | NA |
| England/QEUH-AAD37B/2020 | 2020-10-20 | NA |
| Scotland/QEUH-A9E1CE/2020 | 2020-10-20 | NA |
| Scotland/QEUH-A9AEAD/2020 | 2020-10-20 | NA |
| England/MILK-AC7988/2020 | 2020-10-20 | NA |
| England/QEUH-A9E191/2020 | 2020-10-20 | NA |
| England/QEUH-A9D3E7/2020 | 2020-10-20 | NA |
| England/QEUH-A9C4E5/2020 | 2020-10-20 | NA |
| England/QEUH-AA0581/2020 | 2020-10-20 | NA |
| Wales/ALDP-A955AD/2020 | 2020-10-20 | NA |
| England/QEUH-A9F52B/2020 | 2020-10-20 | NA |
| England/QEUH-A9D96D/2020 | 2020-10-20 | NA |
| England/QEUH-A9ECDC/2020 | 2020-10-20 | NA |
| England/QEUH-AAF42B/2020 | 2020-10-20 | NA |
| England/QEUH-A9EA4B/2020 | 2020-10-20 | NA |
| England/MILK-ACCFDC/2020 | 2020-10-20 | NA |
| England/NORW-F1503/2020 | 2020-10-20 | NA |
| England/NORW-F15A9/2020 | 2020-10-20 | NA |
| England/QEUH-AAF27C/2020 | 2020-10-20 | NA |
| England/ALDP-A936F7/2020 | 2020-10-20 | NA |
| England/ALDP-A92E5A/2020 | 2020-10-20 | NA |
| England/QEUH-AAB01C/2020 | 2020-10-20 | NA |
| England/QEUH-AAEA2A/2020 | 2020-10-20 | NA |
| Wales/PHWC-496D5F/2020 | 2020-10-20 | NA |
| England/QEUH-A9D675/2020 | 2020-10-20 | NA |
| England/QEUH-A9F89B/2020 | 2020-10-20 | NA |
| England/PHEC-14A221/2020 | 2020-10-20 | NA |
| England/MILK-AB8717/2020 | 2020-10-20 | NA |
| England/QEUH-A9F02A/2020 | 2020-10-20 | NA |
| England/QEUH-A9F127/2020 | 2020-10-20 | NA |
| England/QEUH-A9D3C9/2020 | 2020-10-20 | NA |
| England/QEUH-A9F01B/2020 | 2020-10-20 | NA |
| England/ALDP-A92ED2/2020 | 2020-10-20 | NA |
| England/ALDP-A92EA5/2020 | 2020-10-20 | NA |
| England/QEUH-A9D912/2020 | 2020-10-20 | NA |
| England/QEUH-A9D8D9/2020 | 2020-10-20 | NA |
| England/MILK-ACD023/2020 | 2020-10-21 | NA |
| England/ALDP-AA4DA3/2020 | 2020-10-21 | NA |
| England/QEUH-AACF2D/2020 | 2020-10-21 | NA |
| England/MILK-ACE356/2020 | 2020-10-21 | NA |
| England/MILK-ACE972/2020 | 2020-10-21 | NA |
| England/MILK-ACF3A0/2020 | 2020-10-21 | NA |
| England/MILK-AC81CB/2020 | 2020-10-21 | NA |
| England/MILK-AB8735/2020 | 2020-10-21 | NA |
| Wales/ALDP-AA48A2/2020 | 2020-10-21 | NA |
| England/MILK-ACD0F6/2020 | 2020-10-21 | NA |
| England/QEUH-AAC88C/2020 | 2020-10-21 | NA |
| England/QEUH-AAC136/2020 | 2020-10-21 | NA |
| England/QEUH-AAC85F/2020 | 2020-10-21 | NA |
| England/MILK-ACE769/2020 | 2020-10-21 | NA |
| England/MILK-ACE74B/2020 | 2020-10-21 | NA |
| England/MILK-ABBCAC/2020 | 2020-10-21 | NA |
| England/MILK-ACC8C2/2020 | 2020-10-21 | NA |
| England/MILK-AC8125/2020 | 2020-10-21 | NA |
| England/MILK-ACE70F/2020 | 2020-10-21 | NA |
| England/EXET-1388E8/2020 | 2020-10-21 | NA |
| England/MILK-AC8608/2020 | 2020-10-21 | NA |
| England/CAMC-AB14F9/2020 | 2020-10-21 | NA |
| England/MILK-AC8170/2020 | 2020-10-21 | NA |
| England/QEUH-AAB79F/2020 | 2020-10-21 | NA |
| England/ALDP-AA4E82/2020 | 2020-10-21 | NA |
| England/ALDP-AA4E28/2020 | 2020-10-21 | NA |
| England/ALDP-AA4E46/2020 | 2020-10-21 | NA |
| England/QEUH-AAC92F/2020 | 2020-10-21 | NA |
| England/NORT-1B61272/2020 | 2020-10-21 | NA |
| England/QEUH-AAC6FB/2020 | 2020-10-21 | NA |
| England/QEUH-AAAE13/2020 | 2020-10-21 | NA |
| England/ALDP-AA5425/2020 | 2020-10-21 | NA |
| England/MILK-AB7FA7/2020 | 2020-10-21 | NA |
| England/MILK-AB7F98/2020 | 2020-10-21 | NA |
| England/MILK-AC7D9B/2020 | 2020-10-21 | NA |
| England/MILK-AB7F3E/2020 | 2020-10-21 | NA |
| England/QEUH-AAC6EC/2020 | 2020-10-21 | NA |
| England/QEUH-AAB45C/2020 | 2020-10-21 | NA |
| England/MILK-AC842C/2020 | 2020-10-21 | NA |
| England/MILK-ABB2B9/2020 | 2020-10-21 | NA |
| England/MILK-AB746C/2020 | 2020-10-21 | NA |
| England/MILK-AB753C/2020 | 2020-10-21 | NA |
| England/MILK-ACC4BF/2020 | 2020-10-21 | NA |
| England/QEUH-AAAC73/2020 | 2020-10-21 | NA |
| England/QEUH-AAA8AC/2020 | 2020-10-21 | NA |
| England/MILK-ACC4DD/2020 | 2020-10-21 | NA |
| England/MILK-ACC4EC/2020 | 2020-10-21 | NA |
| Wales/ALDP-AA49AF/2020 | 2020-10-21 | NA |
| England/MILK-AB83F5/2020 | 2020-10-21 | NA |
| England/MILK-AC7E4D/2020 | 2020-10-21 | NA |
| England/ALDP-AA40F5/2020 | 2020-10-21 | NA |
| England/MILK-B031C3/2020 | 2020-10-21 | NA |
| England/MILK-AC79C4/2020 | 2020-10-21 | NA |
| England/MILK-AC80BF/2020 | 2020-10-21 | NA |
| England/MILK-AC7CAD/2020 | 2020-10-21 | NA |
| England/NORW-F14F7/2020 | 2020-10-21 | NA |
| England/NORW-F20B6/2020 | 2020-10-21 | NA |
| England/MILK-ACC570/2020 | 2020-10-21 | NA |
| Wales/ALDP-AA5443/2020 | 2020-10-21 | NA |
| Wales/ALDP-AA4D58/2020 | 2020-10-21 | NA |
| England/MILK-ACEE0A/2020 | 2020-10-21 | NA |
| England/ALDP-AA4E55/2020 | 2020-10-21 | NA |
| England/ALDP-AA4DFE/2020 | 2020-10-21 | NA |
| England/ALDP-AA0C04/2020 | 2020-10-21 | NA |
| Scotland/QEUH-AAC9F2/2020 | 2020-10-21 | NA |
| England/MILK-ACD6B8/2020 | 2020-10-21 | NA |
| Wales/PHWC-485922/2020 | 2020-10-21 | NA |
| England/LIVE-DB3C65/2020 | 2020-10-21 | NA |
| England/CAMC-AAFC23/2020 | 2020-10-22 | NA |
| Wales/PHWC-4855C4/2020 | 2020-10-22 | NA |
| England/MILK-AC73E4/2020 | 2020-10-22 | NA |
| Wales/ALDP-AA3797/2020 | 2020-10-22 | NA |
| England/ALDP-AA2200/2020 | 2020-10-22 | NA |
| England/MILK-AC9300/2020 | 2020-10-22 | NA |
| England/QEUH-AAECAC/2020 | 2020-10-22 | NA |
| England/QEUH-AADCDA/2020 | 2020-10-22 | NA |
| England/QEUH-AAE662/2020 | 2020-10-22 | NA |
| England/QEUH-AAED21/2020 | 2020-10-22 | NA |
| England/QEUH-AAE77E/2020 | 2020-10-22 | NA |
| England/QEUH-AAE7E7/2020 | 2020-10-22 | NA |
| England/QEUH-AAE644/2020 | 2020-10-22 | NA |
| England/ALDP-AA32B4/2020 | 2020-10-22 | NA |
| England/MILK-ACD1A8/2020 | 2020-10-22 | NA |
| England/MILK-AC90CD/2020 | 2020-10-22 | NA |
| England/MILK-ACF2B2/2020 | 2020-10-22 | NA |
| England/MILK-ACF917/2020 | 2020-10-22 | NA |
| England/ALDP-AA1E85/2020 | 2020-10-22 | NA |
| England/QEUH-AAED7C/2020 | 2020-10-22 | NA |
| England/QEUH-AAEC7F/2020 | 2020-10-22 | NA |
| England/QEUH-AAEC60/2020 | 2020-10-22 | NA |
| England/QEUH-AADFFF/2020 | 2020-10-22 | NA |
| England/MILK-ACFEFA/2020 | 2020-10-22 | NA |
| England/MILK-AC8DD6/2020 | 2020-10-22 | NA |
| England/QEUH-AAE107/2020 | 2020-10-22 | NA |
| England/MILK-ABBFEF/2020 | 2020-10-22 | NA |
| England/ALDP-AA1F0A/2020 | 2020-10-22 | NA |
| England/QEUH-AAE7AB/2020 | 2020-10-22 | NA |
| England/ALDP-AA3FBD/2020 | 2020-10-22 | NA |
| England/QEUH-AAE073/2020 | 2020-10-22 | NA |
| England/ALDP-AA4DB2/2020 | 2020-10-22 | NA |
| England/CAMC-AAFEF0/2020 | 2020-10-22 | NA |
| Wales/PHWC-48370C/2020 | 2020-10-22 | NA |
| England/ALDP-AA22A6/2020 | 2020-10-22 | NA |
| England/ALDP-AA4EBF/2020 | 2020-10-22 | NA |
| England/ALDP-AA572C/2020 | 2020-10-22 | NA |
| England/ALDP-AA2F18/2020 | 2020-10-22 | NA |
| England/ALDP-AA284A/2020 | 2020-10-22 | NA |
| England/ALDP-AA3BC8/2020 | 2020-10-22 | NA |
| England/ALDP-AA3937/2020 | 2020-10-22 | NA |
| England/ALDP-AA3DC2/2020 | 2020-10-22 | NA |
| England/ALDP-AA3E0B/2020 | 2020-10-22 | NA |
| England/ALDP-AA39EC/2020 | 2020-10-22 | NA |
| England/ALDP-AA2051/2020 | 2020-10-22 | NA |
| Wales/PHWC-48610B/2020 | 2020-10-22 | NA |
| England/MILK-ABB86C/2020 | 2020-10-22 | NA |
| England/ALDP-AA38EF/2020 | 2020-10-22 | NA |
| England/QEUH-AAE046/2020 | 2020-10-22 | NA |
| England/QEUH-AAEF67/2020 | 2020-10-22 | NA |
| England/ALDP-AA2789/2020 | 2020-10-22 | NA |
| England/QEUH-AAE6F9/2020 | 2020-10-22 | NA |
| England/ALDP-AA0E2C/2020 | 2020-10-22 | NA |
| England/ALDP-AA3946/2020 | 2020-10-22 | NA |
| England/MILK-ACE9BE/2020 | 2020-10-22 | NA |
| England/MILK-ACDCD4/2020 | 2020-10-22 | NA |
| England/MILK-ACDCB6/2020 | 2020-10-22 | NA |
| England/QEUH-AAE0CE/2020 | 2020-10-22 | NA |
| England/QEUH-AAE055/2020 | 2020-10-22 | NA |
| England/QEUH-AAE028/2020 | 2020-10-22 | NA |
| England/ALDP-AA1D3D/2020 | 2020-10-22 | NA |
| England/ALDP-AA281D/2020 | 2020-10-22 | NA |
| England/ALDP-AA3A70/2020 | 2020-10-22 | NA |
| England/ALDP-AA3BD7/2020 | 2020-10-22 | NA |
| England/ALDP-AA21F4/2020 | 2020-10-22 | NA |
| England/NOTT-117490/2020 | 2020-10-22 | NA |
| England/MILK-ACEEEC/2020 | 2020-10-22 | NA |
| England/MILK-ACEEFB/2020 | 2020-10-22 | NA |
| England/ALDP-AA34FA/2020 | 2020-10-22 | NA |
| England/ALDP-AA357F/2020 | 2020-10-22 | NA |
| England/ALDP-AA3463/2020 | 2020-10-22 | NA |
| Northern_Ireland/NIRE-109428/2020 | 2020-10-22 | NA |
| England/MILK-ACBE94/2020 | 2020-10-22 | NA |
| England/MILK-AD002D/2020 | 2020-10-22 | NA |
| England/ALDP-AA2E57/2020 | 2020-10-22 | NA |
| England/ALDP-AA0BF8/2020 | 2020-10-22 | NA |
| England/MILK-B14E9B/2020 | 2020-10-22 | NA |
| England/MILK-AC89F0/2020 | 2020-10-22 | NA |
| England/MILK-AB7A88/2020 | 2020-10-22 | NA |
| Wales/ALDP-AA0949/2020 | 2020-10-22 | NA |
| England/MILK-AC827D/2020 | 2020-10-22 | NA |
| England/MILK-AC828C/2020 | 2020-10-22 | NA |
| England/NORW-F1512/2020 | 2020-10-22 | NA |
| England/ALDP-AA2D4B/2020 | 2020-10-22 | NA |
| England/CAMC-AAFC32/2020 | 2020-10-22 | NA |
| Wales/ALDP-AA1C6D/2020 | 2020-10-22 | NA |
| Wales/ALDP-AA1C30/2020 | 2020-10-22 | NA |
| England/ALDP-AA380D/2020 | 2020-10-22 | NA |
| England/ALDP-AA383A/2020 | 2020-10-22 | NA |
| Scotland/QEUH-AADD31/2020 | 2020-10-22 | NA |
| England/ALDP-AA29FC/2020 | 2020-10-22 | NA |
| England/ALDP-AA2DF0/2020 | 2020-10-22 | NA |
| England/ALDP-AA1623/2020 | 2020-10-22 | NA |
| England/ALDP-AA2A80/2020 | 2020-10-22 | NA |
| England/ALDP-AA2A53/2020 | 2020-10-22 | NA |
| England/PORT-2D73BC/2020 | 2020-10-22 | NA |
| England/ALDP-AA34CD/2020 | 2020-10-22 | NA |
| England/ALDP-AA1201/2020 | 2020-10-22 | NA |
| England/ALDP-AA369A/2020 | 2020-10-22 | NA |
| England/MILK-AC8E5B/2020 | 2020-10-22 | NA |
| England/QEUH-AADDC8/2020 | 2020-10-22 | NA |
| England/ALDP-AA2A44/2020 | 2020-10-22 | NA |
| England/ALDP-AA3AAD/2020 | 2020-10-22 | NA |
| England/ALDP-AA1887/2020 | 2020-10-22 | NA |
| England/ALDP-AA29DE/2020 | 2020-10-22 | NA |
| England/ALDP-AA2A17/2020 | 2020-10-22 | NA |
| England/MILK-AC8FFE/2020 | 2020-10-22 | NA |
| England/LIVE-DB3A2F/2020 | 2020-10-22 | NA |
| England/LIVE-DC88F0/2020 | 2020-10-22 | NA |
| England/ALDP-AA17A8/2020 | 2020-10-22 | NA |
| England/CAMC-AB13DE/2020 | 2020-10-23 | NA |
| England/CAMC-AB1408/2020 | 2020-10-23 | NA |
| England/NORT-1B61737/2020 | 2020-10-23 | NA |
| England/MILK-B04DCE/2020 | 2020-10-23 | NA |
| England/CAMC-AAF959/2020 | 2020-10-23 | NA |
| England/QEUH-AD442D/2020 | 2020-10-23 | NA |
| England/MILK-B024E9/2020 | 2020-10-23 | NA |
| England/MILK-B04775/2020 | 2020-10-23 | NA |
| England/EXET-138ED7/2020 | 2020-10-23 | NA |
| Wales/PHWC-484774/2020 | 2020-10-23 | NA |
| England/NORT-286D90/2020 | 2020-10-23 | NA |
| England/MILK-B150FE/2020 | 2020-10-23 | NA |
| England/QEUH-AD15C3/2020 | 2020-10-23 | NA |
| England/CAMC-AAFB62/2020 | 2020-10-23 | NA |
| England/CAMC-AB15E7/2020 | 2020-10-23 | NA |
| England/CAMC-AB1EFB/2020 | 2020-10-23 | NA |
| England/PHEC-14AA29/2020 | 2020-10-23 | NA |
| Wales/PHWC-487085/2020 | 2020-10-23 | NA |
| Wales/PHWC-485324/2020 | 2020-10-23 | NA |
| England/QEUH-AD11DE/2020 | 2020-10-24 | NA |
| England/MILK-B046C3/2020 | 2020-10-24 | NA |
| England/MILK-B02917/2020 | 2020-10-24 | NA |
| England/MILK-B01D58/2020 | 2020-10-24 | NA |
| England/MILK-B05255/2020 | 2020-10-24 | NA |
| England/MILK-B01E0A/2020 | 2020-10-24 | NA |
| Wales/MILK-B0639D/2020 | 2020-10-24 | NA |
| Wales/MILK-B06236/2020 | 2020-10-24 | NA |
| England/QEUH-AD260B/2020 | 2020-10-24 | NA |
| England/EXET-13A154/2020 | 2020-10-24 | NA |
| England/QEUH-AD0A98/2020 | 2020-10-24 | NA |
| England/QEUH-AD37CB/2020 | 2020-10-24 | NA |
| England/MILK-B0601E/2020 | 2020-10-24 | NA |
| England/MILK-B027B3/2020 | 2020-10-24 | NA |
| England/MILK-B02601/2020 | 2020-10-24 | NA |
| England/QEUH-AD0F5D/2020 | 2020-10-24 | NA |
| England/QEUH-AD07BF/2020 | 2020-10-24 | NA |
| England/MILK-B04ECB/2020 | 2020-10-24 | NA |
| England/MILK-B0545F/2020 | 2020-10-24 | NA |
| England/MILK-B02829/2020 | 2020-10-24 | NA |
| England/MILK-B02865/2020 | 2020-10-24 | NA |
| England/MILK-B046D2/2020 | 2020-10-24 | NA |
| England/LIVE-DB4A5B/2020 | 2020-10-24 | NA |
| England/MILK-B049AC/2020 | 2020-10-24 | NA |
| England/CAMB-1B77B7/2020 | 2020-10-25 | NA |
| England/NORT-286D45/2020 | 2020-10-25 | NA |
| England/QEUH-AD2F4C/2020 | 2020-10-25 | NA |
| England/NORT-286D63/2020 | 2020-10-25 | NA |
| England/QEUH-AD578D/2020 | 2020-10-25 | NA |
| Wales/PHWC-485184/2020 | 2020-10-25 | NA |
| England/CAMC-AEACE0/2020 | 2020-10-25 | NA |
| Wales/PHWC-4894D2/2020 | 2020-10-25 | NA |
| England/QEUH-AD1C46/2020 | 2020-10-26 | NA |
| England/QEUH-AD2FF1/2020 | 2020-10-26 | NA |
| England/QEUH-AD04E5/2020 | 2020-10-26 | NA |
| England/BRIS-1855292/2020 | 2020-10-26 | NA |
| England/QEUH-AD2DE8/2020 | 2020-10-26 | NA |
| England/BRIS-1855238/2020 | 2020-10-26 | NA |
| England/QEUH-AD3C71/2020 | 2020-10-26 | NA |
| England/QEUH-AD2726/2020 | 2020-10-26 | NA |
| Luxembourg/LNS1057454/2020 | 2020-10-26 | NA |
| England/CAMB-1B91B7/2020 | 2020-10-26 | NA |
| England/CAMB-1B924B/2020 | 2020-10-26 | NA |
| England/CAMB-1B91F3/2020 | 2020-10-26 | NA |
| England/QEUH-AD3D7E/2020 | 2020-10-26 | NA |
| England/QEUH-AD0852/2020 | 2020-10-26 | NA |
| England/NORT-1B6156A/2020 | 2020-10-26 | NA |
| England/QEUH-AD387D/2020 | 2020-10-26 | NA |
| England/QEUH-AD19F4/2020 | 2020-10-26 | NA |
| England/QEUH-AD0AD4/2020 | 2020-10-26 | NA |
| England/QEUH-AD1639/2020 | 2020-10-26 | NA |
| England/QEUH-AD0FB7/2020 | 2020-10-26 | NA |
| England/QEUH-AD388C/2020 | 2020-10-26 | NA |
| Wales/PHWC-489487/2020 | 2020-10-26 | NA |
| Wales/QEUH-AE1D13/2020 | 2020-10-26 | NA |
| Scotland/EDB8814/2020 | 2020-10-26 | NA |
| England/QEUH-AD29B7/2020 | 2020-10-26 | NA |
| England/QEUH-AE21AD/2020 | 2020-10-26 | NA |
| England/QEUH-AD16B1/2020 | 2020-10-26 | NA |
| England/NORT-286D72/2020 | 2020-10-26 | NA |
| England/QEUH-AD0A5C/2020 | 2020-10-26 | NA |
| England/QEUH-AD1F89/2020 | 2020-10-26 | NA |
| England/QEUH-AD1F2F/2020 | 2020-10-26 | NA |
| England/QEUH-AD1E6E/2020 | 2020-10-26 | NA |
| England/QEUH-AD1ED7/2020 | 2020-10-26 | NA |
| England/QEUH-AD09E6/2020 | 2020-10-26 | NA |
| England/NORT-286E33/2020 | 2020-10-26 | NA |
| England/QEUH-AD19C7/2020 | 2020-10-26 | NA |
| England/QEUH-AD3743/2020 | 2020-10-26 | NA |
| England/QEUH-AD19D6/2020 | 2020-10-26 | NA |
| England/QEUH-AD3989/2020 | 2020-10-26 | NA |
| England/QEUH-AD2CEB/2020 | 2020-10-26 | NA |
| England/QEUH-AD5C60/2020 | 2020-10-26 | NA |
| England/QEUH-AD1EF5/2020 | 2020-10-26 | NA |
| England/QEUH-AD25C2/2020 | 2020-10-26 | NA |
| England/QEUH-AD1CEC/2020 | 2020-10-26 | NA |
| England/QEUH-AD396B/2020 | 2020-10-26 | NA |
| England/QEUH-AD5B45/2020 | 2020-10-26 | NA |
| England/CAMC-AEA785/2020 | 2020-10-26 | NA |
| England/QEUH-AD5A1B/2020 | 2020-10-26 | NA |
| England/QEUH-AD491F/2020 | 2020-10-26 | NA |
| England/BRIS-185539F/2020 | 2020-10-26 | NA |
| England/QEUH-AD31FA/2020 | 2020-10-26 | NA |
| England/QEUH-AD5170/2020 | 2020-10-26 | NA |
| England/QEUH-AD5E6A/2020 | 2020-10-26 | NA |
| England/QEUH-AD2AE1/2020 | 2020-10-26 | NA |
| England/QEUH-AD3127/2020 | 2020-10-26 | NA |
| Wales/QEUH-AE1C34/2020 | 2020-10-26 | NA |
| England/QEUH-AE1B91/2020 | 2020-10-26 | NA |
| England/QEUH-AD0FE4/2020 | 2020-10-26 | NA |
| England/QEUH-AD1244/2020 | 2020-10-26 | NA |
| England/QEUH-AD22BC/2020 | 2020-10-26 | NA |
| England/QEUH-AD2674/2020 | 2020-10-26 | NA |
| England/QEUH-AD5125/2020 | 2020-10-26 | NA |
| England/QEUH-AD3761/2020 | 2020-10-26 | NA |
| England/QEUH-AD2058/2020 | 2020-10-26 | NA |
| England/QEUH-AE5438/2020 | 2020-10-26 | NA |
| England/QEUH-AE21E9/2020 | 2020-10-26 | NA |
| Wales/QEUH-AD3A59/2020 | 2020-10-26 | NA |
| Wales/PHWC-486D16/2020 | 2020-10-26 | NA |
| Wales/QEUH-AD4320/2020 | 2020-10-26 | NA |
| England/QEUH-AD09C8/2020 | 2020-10-26 | NA |
| England/QEUH-AD4A1C/2020 | 2020-10-26 | NA |
| England/QEUH-AD30A2/2020 | 2020-10-26 | NA |
| Wales/PHWC-487340/2020 | 2020-10-27 | NA |
| England/MILK-ABB8A8/2020 | 2020-10-27 | NA |
| England/QEUH-AE4F92/2020 | 2020-10-27 | NA |
| England/NORT-1B8716A/2020 | 2020-10-27 | NA |
| England/QEUH-AE4FB0/2020 | 2020-10-27 | NA |
| England/QEUH-AE2714/2020 | 2020-10-27 | NA |
| England/EXET-13910A/2020 | 2020-10-27 | NA |
| England/WSFT-25C5822/2020 | 2020-10-27 | NA |
| England/QEUH-AE2EC4/2020 | 2020-10-27 | NA |
| England/QEUH-AE4E2C/2020 | 2020-10-27 | NA |
| England/QEUH-AE232F/2020 | 2020-10-27 | NA |
| England/QEUH-AD1314/2020 | 2020-10-27 | NA |
| England/QEUH-AE2B45/2020 | 2020-10-27 | NA |
| England/QEUH-AE4FCF/2020 | 2020-10-27 | NA |
| England/QEUH-AE23E3/2020 | 2020-10-27 | NA |
| England/QEUH-AE4994/2020 | 2020-10-27 | NA |
| England/SHEF-CDA01/2020 | 2020-10-27 | NA |
| Scotland/QEUH-AE28B7/2020 | 2020-10-27 | NA |
| Scotland/QEUH-AE26AE/2020 | 2020-10-27 | NA |
| England/QEUH-AE53C2/2020 | 2020-10-27 | NA |
| Scotland/QEUH-AE4A0A/2020 | 2020-10-27 | NA |
| England/LIVE-DBE9CD/2020 | 2020-10-27 | NA |
| Scotland/QEUH-AD2AC3/2020 | 2020-10-27 | NA |
| England/QEUH-AE236B/2020 | 2020-10-27 | NA |
| England/QEUH-AF62FE/2020 | 2020-10-28 | NA |
| England/QEUH-AF62EF/2020 | 2020-10-28 | NA |
| England/PORT-2D7589/2020 | 2020-10-28 | NA |
| England/EKHU-2610186/2020 | 2020-10-28 | NA |
| England/EXET-13901C/2020 | 2020-10-28 | NA |
| England/CAMC-B09619/2020 | 2020-10-28 | NA |
| England/CAMC-B0B705/2020 | 2020-10-28 | NA |
| England/CAMC-B0B6F9/2020 | 2020-10-28 | NA |
| England/QEUH-AEFF97/2020 | 2020-10-28 | NA |
| England/QEUH-AF63A0/2020 | 2020-10-29 | NA |
| England/CAMC-B09479/2020 | 2020-10-29 | NA |
| Norway/5092/2020 | 2020-10-29 | NA |
| England/CAMC-B090FD/2020 | 2020-10-29 | NA |
| England/CAMC-B0AEE3/2020 | 2020-10-29 | NA |
| England/CAMC-B0A609/2020 | 2020-10-29 | NA |
| England/CAMC-B08753/2020 | 2020-10-29 | NA |
| England/CAMC-B080A3/2020 | 2020-10-29 | NA |
| England/CAMC-B08DBB/2020 | 2020-10-29 | NA |
| England/CAMC-B08DAC/2020 | 2020-10-29 | NA |
| England/EXET-138F3E/2020 | 2020-10-29 | NA |
| England/CAMC-B0A8F4/2020 | 2020-10-29 | NA |
| England/CAMC-B098F5/2020 | 2020-10-29 | NA |
| England/QEUH-AEFC18/2020 | 2020-10-29 | NA |
| England/CAMC-B083E6/2020 | 2020-10-29 | NA |
| England/MILK-B06F6C/2020 | 2020-10-29 | NA |
| England/QEUH-AEFFD3/2020 | 2020-10-29 | NA |
| England/EXET-138F5C/2020 | 2020-10-29 | NA |
| England/MILK-B06EBA/2020 | 2020-10-29 | NA |
| England/QEUH-AF72B1/2020 | 2020-10-29 | NA |
| England/CAMC-B07E5F/2020 | 2020-10-29 | NA |
| England/CAMC-B07E8C/2020 | 2020-10-29 | NA |
| England/CAMC-B08911/2020 | 2020-10-29 | NA |
| England/CAMC-B08920/2020 | 2020-10-29 | NA |
| England/CAMC-B0B5FC/2020 | 2020-10-29 | NA |
| Wales/PHWC-48A152/2020 | 2020-10-29 | NA |
| Wales/QEUH-AF62B2/2020 | 2020-10-29 | NA |
| England/QEUH-AF630A/2020 | 2020-10-29 | NA |
| Wales/PHWC-4876ED/2020 | 2020-10-29 | NA |
| England/QEUH-AF06F8/2020 | 2020-10-29 | NA |
| England/CAMC-B073AB/2020 | 2020-10-30 | NA |
| England/CAMC-B08CEB/2020 | 2020-10-30 | NA |
| England/CAMC-B0AB0A/2020 | 2020-10-30 | NA |
| England/QEUH-AF58C0/2020 | 2020-10-30 | NA |
| England/QEUH-AF4C3E/2020 | 2020-10-30 | NA |
| England/BRIS-18556F0/2020 | 2020-10-30 | NA |
| England/QEUH-AF4A43/2020 | 2020-10-30 | NA |
| England/QEUH-AF6777/2020 | 2020-10-30 | NA |
| England/CAMC-B0CAB0/2020 | 2020-10-30 | NA |
| England/CAMC-B0A171/2020 | 2020-10-30 | NA |
| England/QEUH-AF476A/2020 | 2020-10-30 | NA |
| England/QEUH-AF4B9B/2020 | 2020-10-30 | NA |
| England/QEUH-AF61B5/2020 | 2020-10-30 | NA |
| England/QEUH-AF4CE3/2020 | 2020-10-30 | NA |
| England/CAMC-B0A047/2020 | 2020-10-30 | NA |
| England/QEUH-AF49DD/2020 | 2020-10-30 | NA |
| England/CAMC-B073BA/2020 | 2020-10-30 | NA |
| England/CAMC-B080B2/2020 | 2020-10-30 | NA |
| England/QEUH-AF4D1D/2020 | 2020-10-30 | NA |
| England/QEUH-AF60A9/2020 | 2020-10-30 | NA |
| England/QEUH-AF5FF8/2020 | 2020-10-30 | NA |
| England/QEUH-AF5ECE/2020 | 2020-10-30 | NA |
| England/QEUH-AF667A/2020 | 2020-10-30 | NA |
| England/QEUH-AF46D6/2020 | 2020-10-30 | NA |
| England/QEUH-AF665C/2020 | 2020-10-30 | NA |
| England/NORW-F2256/2020 | 2020-10-30 | NA |
| England/EXET-138E7D/2020 | 2020-10-30 | NA |
| England/QEUH-AF6786/2020 | 2020-10-30 | NA |
| England/QEUH-AF53FC/2020 | 2020-10-30 | NA |
| England/QEUH-AF5A8E/2020 | 2020-10-30 | NA |
| England/QEUH-AF5365/2020 | 2020-10-30 | NA |
| England/QEUH-AF5E28/2020 | 2020-10-30 | NA |
| England/QEUH-AF2957/2020 | 2020-10-30 | NA |
| England/QEUH-AF480D/2020 | 2020-10-30 | NA |
| England/QEUH-AF4788/2020 | 2020-10-30 | NA |
| England/QEUH-AF4858/2020 | 2020-10-30 | NA |
| England/QEUH-AF5848/2020 | 2020-10-30 | NA |
| England/QEUH-AF672C/2020 | 2020-10-30 | NA |
| England/QEUH-AF5A7F/2020 | 2020-10-30 | NA |
| England/QEUH-AF56C6/2020 | 2020-10-30 | NA |
| England/QEUH-AF566C/2020 | 2020-10-30 | NA |
| England/QEUH-AF46E5/2020 | 2020-10-30 | NA |
| England/QEUH-AF4120/2020 | 2020-10-30 | NA |
| England/QEUH-AF48D0/2020 | 2020-10-30 | NA |
| England/QEUH-AF226B/2020 | 2020-10-30 | NA |
| England/NORW-F3C1B/2020 | 2020-10-30 | NA |
| England/CAMC-B0AE01/2020 | 2020-10-30 | NA |
| England/BRIS-1855A21/2020 | 2020-10-30 | NA |
| England/CAMC-B096A0/2020 | 2020-10-30 | NA |
| England/CAMC-B084E3/2020 | 2020-10-30 | NA |
| England/QEUH-AF52E0/2020 | 2020-10-30 | NA |
| England/QEUH-AF52FF/2020 | 2020-10-30 | NA |
| England/CAMC-B0C8D4/2020 | 2020-10-30 | NA |
| England/QEUH-AF567B/2020 | 2020-10-30 | NA |
| England/QEUH-AF28B4/2020 | 2020-10-30 | NA |
| England/QEUH-AF28A5/2020 | 2020-10-30 | NA |
| England/QEUH-AF5DEF/2020 | 2020-10-30 | NA |
| England/QEUH-AF55F6/2020 | 2020-10-30 | NA |
| England/QEUH-AF5F16/2020 | 2020-10-30 | NA |
| England/ALDP-B1B2F2/2020 | 2020-10-31 | NA |
| England/EXET-13A5EF/2020 | 2020-10-31 | NA |
| England/QEUH-B0DF83/2020 | 2020-10-31 | NA |
| England/SHEF-CF1BF/2020 | 2020-10-31 | NA |
| England/MILK-B3AB98/2020 | 2020-10-31 | NA |
| England/QEUH-B129E7/2020 | 2020-10-31 | NA |
| England/CAMC-B099A7/2020 | 2020-10-31 | NA |
| England/ALDP-B1B4A1/2020 | 2020-10-31 | NA |
| England/CAMC-B077DC/2020 | 2020-10-31 | NA |
| England/CAMC-B0C83E/2020 | 2020-10-31 | NA |
| England/MILK-B3B177/2020 | 2020-10-31 | NA |
| England/QEUH-B124E6/2020 | 2020-10-31 | NA |
| England/CAMC-B074D5/2020 | 2020-10-31 | NA |
| England/MILK-B38AE8/2020 | 2020-10-31 | NA |
| Wales/MILK-B3C10D/2020 | 2020-10-31 | NA |
| England/MILK-B3B186/2020 | 2020-10-31 | NA |
| England/QEUH-B137CE/2020 | 2020-10-31 | NA |
| England/QEUH-B2E10E/2020 | 2020-10-31 | NA |
| England/MILK-B38769/2020 | 2020-11-01 | NA |
| England/QEUH-B13069/2020 | 2020-11-01 | NA |
| England/MILK-B38699/2020 | 2020-11-01 | NA |
| England/MILK-B3DC1A/2020 | 2020-11-01 | NA |
| England/MILK-B386F3/2020 | 2020-11-01 | NA |
| England/MILK-B386B7/2020 | 2020-11-01 | NA |
| England/MILK-B3CD72/2020 | 2020-11-01 | NA |
| England/MILK-B3ACB3/2020 | 2020-11-01 | NA |
| England/EXET-13A5A3/2020 | 2020-11-01 | NA |
| England/ALDP-B1D15D/2020 | 2020-11-01 | NA |
| England/ALDP-B1D16C/2020 | 2020-11-01 | NA |
| England/MILK-B38444/2020 | 2020-11-01 | NA |
| England/QEUH-B12A3F/2020 | 2020-11-01 | NA |
| England/ALDP-B1D22D/2020 | 2020-11-01 | NA |
| Wales/ALDP-B1BDA6/2020 | 2020-11-01 | NA |
| England/MILK-B3CA11/2020 | 2020-11-01 | NA |
| England/MILK-B3D7DD/2020 | 2020-11-01 | NA |
| England/ALDP-B1CBF6/2020 | 2020-11-01 | NA |
| England/QEUH-B2CA23/2020 | 2020-11-01 | NA |
| England/MILK-B3CAE4/2020 | 2020-11-01 | NA |
| England/MILK-B3C422/2020 | 2020-11-01 | NA |
| England/MILK-B3A099/2020 | 2020-11-01 | NA |
| England/ALDP-B1A93A/2020 | 2020-11-01 | NA |
| Wales/PHWC-48CFFC/2020 | 2020-11-01 | NA |
| Wales/PHWC-48C7E5/2020 | 2020-11-01 | NA |
| England/CAMC-B20F99/2020 | 2020-11-01 | NA |
| England/ALDP-B1DBAA/2020 | 2020-11-01 | NA |
| England/ALDP-B1C77A/2020 | 2020-11-01 | NA |
| England/ALDP-B1B535/2020 | 2020-11-01 | NA |
| England/MILK-B394BC/2020 | 2020-11-01 | NA |
| England/ALDP-B1BB9D/2020 | 2020-11-01 | NA |
| Wales/ALDP-B1A7E5/2020 | 2020-11-01 | NA |
| England/CAMC-B2288D/2020 | 2020-11-01 | NA |
| Wales/ALDP-B1AB16/2020 | 2020-11-01 | NA |
| England/ALDP-B1D07E/2020 | 2020-11-01 | NA |
| England/ALDP-B1A800/2020 | 2020-11-01 | NA |
| England/ALDP-B1D72E/2020 | 2020-11-01 | NA |
| England/ALDP-B1BF19/2020 | 2020-11-01 | NA |
| England/ALDP-B1D533/2020 | 2020-11-01 | NA |
| England/ALDP-B1D603/2020 | 2020-11-01 | NA |
| Denmark/DCGC-10785/2020 | 2020-11-02 | NA |
| England/QEUH-B0E368/2020 | 2020-11-02 | NA |
| England/CAMC-B22D60/2020 | 2020-11-02 | NA |
| England/CAMC-B22D51/2020 | 2020-11-02 | NA |
| England/CAMC-B22D06/2020 | 2020-11-02 | NA |
| England/CAMC-B20A4D/2020 | 2020-11-02 | NA |
| England/MILK-B3955F/2020 | 2020-11-02 | NA |
| England/QEUH-B0E8F0/2020 | 2020-11-02 | NA |
| England/QEUH-B0DF92/2020 | 2020-11-02 | NA |
| England/QEUH-B0E939/2020 | 2020-11-02 | NA |
| England/QEUH-B0E8D2/2020 | 2020-11-02 | NA |
| England/QEUH-B0EAFA/2020 | 2020-11-02 | NA |
| England/MILK-B3C53E/2020 | 2020-11-02 | NA |
| England/MILK-B3D5F1/2020 | 2020-11-02 | NA |
| England/ALDP-B1DD2C/2020 | 2020-11-02 | NA |
| Scotland/QEUH-B12589/2020 | 2020-11-02 | NA |
| Scotland/QEUH-B125B6/2020 | 2020-11-02 | NA |
| England/MILK-B3BDFB/2020 | 2020-11-02 | NA |
| England/MILK-B3868A/2020 | 2020-11-02 | NA |
| England/MILK-B3875A/2020 | 2020-11-02 | NA |
| England/QEUH-B0E8C3/2020 | 2020-11-02 | NA |
| England/QEUH-B0F3C1/2020 | 2020-11-02 | NA |
| England/QEUH-B13843/2020 | 2020-11-02 | NA |
| England/QEUH-B0DFCF/2020 | 2020-11-02 | NA |
| England/QEUH-B0E33B/2020 | 2020-11-02 | NA |
| England/QEUH-B11A21/2020 | 2020-11-02 | NA |
| England/QEUH-B0EC7C/2020 | 2020-11-02 | NA |
| England/QEUH-B10F05/2020 | 2020-11-02 | NA |
| Wales/PHWC-488BCF/2020 | 2020-11-02 | NA |
| England/QEUH-B0E25C/2020 | 2020-11-02 | NA |
| England/QEUH-B114F6/2020 | 2020-11-02 | NA |
| England/QEUH-B10E08/2020 | 2020-11-02 | NA |
| England/QEUH-B117B1/2020 | 2020-11-02 | NA |
| England/QEUH-B118BE/2020 | 2020-11-02 | NA |
| England/CAMC-B20EAB/2020 | 2020-11-02 | NA |
| England/QEUH-B2CAF6/2020 | 2020-11-02 | NA |
| England/MILK-B3CEBB/2020 | 2020-11-02 | NA |
| England/CAMC-B21D07/2020 | 2020-11-02 | NA |
| England/QEUH-B0EC9A/2020 | 2020-11-02 | NA |
| England/QEUH-B0F77A/2020 | 2020-11-02 | NA |
| England/QEUH-B0EA72/2020 | 2020-11-02 | NA |
| England/QEUH-B0F0E8/2020 | 2020-11-02 | NA |
| England/QEUH-B0E571/2020 | 2020-11-02 | NA |
| England/CAMC-B22C09/2020 | 2020-11-02 | NA |
| England/CAMC-B2338B/2020 | 2020-11-02 | NA |
| England/QEUH-B115F3/2020 | 2020-11-02 | NA |
| England/QEUH-B0E966/2020 | 2020-11-02 | NA |
| England/QEUH-B0E92A/2020 | 2020-11-02 | NA |
| England/QEUH-B12659/2020 | 2020-11-02 | NA |
| England/QEUH-B1396E/2020 | 2020-11-02 | NA |
| England/QEUH-B0F376/2020 | 2020-11-02 | NA |
| England/QEUH-B0F2E2/2020 | 2020-11-02 | NA |
| England/QEUH-B30BB0/2020 | 2020-11-02 | NA |
| England/QEUH-B30B47/2020 | 2020-11-02 | NA |
| England/QEUH-B30C08/2020 | 2020-11-02 | NA |
| England/QEUH-B0E993/2020 | 2020-11-02 | NA |
| England/QEUH-B0E8A5/2020 | 2020-11-02 | NA |
| England/QEUH-B12343/2020 | 2020-11-02 | NA |
| England/QEUH-B11845/2020 | 2020-11-02 | NA |
| England/MILK-B3A5F4/2020 | 2020-11-02 | NA |
| England/QEUH-B10776/2020 | 2020-11-02 | NA |
| Scotland/QEUH-B118DC/2020 | 2020-11-02 | NA |
| England/MILK-B3BDA0/2020 | 2020-11-02 | NA |
| England/QEUH-B0EB9D/2020 | 2020-11-02 | NA |
| England/MILK-B39179/2020 | 2020-11-02 | NA |
| England/QEUH-B12A8A/2020 | 2020-11-02 | NA |
| England/NORT-1B8714C/2020 | 2020-11-02 | NA |
| England/QEUH-B10D38/2020 | 2020-11-02 | NA |
| England/QEUH-B10D83/2020 | 2020-11-02 | NA |
| England/QEUH-B13694/2020 | 2020-11-02 | NA |
| England/MILK-B39ABA/2020 | 2020-11-02 | NA |
| England/CAMC-B206EF/2020 | 2020-11-02 | NA |
| England/CAMC-B1FCB4/2020 | 2020-11-02 | NA |
| England/CAMC-B23136/2020 | 2020-11-02 | NA |
| England/CAMC-B2228F/2020 | 2020-11-02 | NA |
| England/CAMC-B2239B/2020 | 2020-11-02 | NA |
| England/CAMC-B225A4/2020 | 2020-11-02 | NA |
| England/QEUH-B2E089/2020 | 2020-11-02 | NA |
| England/QEUH-B0E69C/2020 | 2020-11-02 | NA |
| England/MILK-B3C282/2020 | 2020-11-02 | NA |
| England/QEUH-B2B8FD/2020 | 2020-11-02 | NA |
| England/CAMC-B22AC3/2020 | 2020-11-02 | NA |
| England/MILK-B3BB1F/2020 | 2020-11-02 | NA |
| England/CAMC-B223D7/2020 | 2020-11-02 | NA |
| England/CAMC-B2252C/2020 | 2020-11-02 | NA |
| England/MILK-B3C880/2020 | 2020-11-02 | NA |
| England/QEUH-B11B00/2020 | 2020-11-02 | NA |
| England/QEUH-B13904/2020 | 2020-11-02 | NA |
| England/QEUH-B10020/2020 | 2020-11-02 | NA |
| England/CAMC-B22410/2020 | 2020-11-02 | NA |
| England/QEUH-B0F9B0/2020 | 2020-11-02 | NA |
| England/QEUH-B0DC8C/2020 | 2020-11-02 | NA |
| England/QEUH-B2C9BD/2020 | 2020-11-02 | NA |
| England/QEUH-B1155D/2020 | 2020-11-02 | NA |
| England/QEUH-B1344F/2020 | 2020-11-02 | NA |
| England/QEUH-B1005D/2020 | 2020-11-02 | NA |
| England/QEUH-B2C9DB/2020 | 2020-11-02 | NA |
| England/QEUH-B2E906/2020 | 2020-11-02 | NA |
| England/QEUH-B0E201/2020 | 2020-11-02 | NA |
| England/QEUH-B0E22F/2020 | 2020-11-02 | NA |
| England/QEUH-B0E131/2020 | 2020-11-02 | NA |
| England/QEUH-B0E23E/2020 | 2020-11-02 | NA |
| Scotland/EDB9161/2020 | 2020-11-02 | NA |
| England/ALDP-B1A080/2020 | 2020-11-02 | NA |
| England/QEUH-B122A0/2020 | 2020-11-02 | NA |
| England/QEUH-B0ECE5/2020 | 2020-11-02 | NA |
| England/QEUH-B0EE2B/2020 | 2020-11-02 | NA |
| England/QEUH-B2DD47/2020 | 2020-11-03 | NA |
| England/CAMC-B21F89/2020 | 2020-11-03 | NA |
| England/CAMC-B214D5/2020 | 2020-11-03 | NA |
| England/CAMC-B214C6/2020 | 2020-11-03 | NA |
| England/QEUH-B14226/2020 | 2020-11-03 | NA |
| England/PORT-2D760E/2020 | 2020-11-03 | NA |
| England/QEUH-B14217/2020 | 2020-11-03 | NA |
| England/QEUH-B14402/2020 | 2020-11-03 | NA |
| England/QEUH-B1437E/2020 | 2020-11-03 | NA |
| England/QEUH-B1447B/2020 | 2020-11-03 | NA |
| England/LOND-12F03AB/2020 | 2020-11-03 | NA |
| England/QEUH-B135C4/2020 | 2020-11-03 | NA |
| England/QEUH-B14596/2020 | 2020-11-03 | NA |
| England/QEUH-B14569/2020 | 2020-11-03 | NA |
| England/QEUH-B2D776/2020 | 2020-11-03 | NA |
| England/QEUH-B2B4BD/2020 | 2020-11-03 | NA |
| England/QEUH-B2E098/2020 | 2020-11-03 | NA |
| England/QEUH-B1462A/2020 | 2020-11-03 | NA |
| England/QEUH-B2E274/2020 | 2020-11-03 | NA |
| England/NORT-2A104B/2020 | 2020-11-03 | NA |
| England/QEUH-B141ED/2020 | 2020-11-03 | NA |
| England/QEUH-B14068/2020 | 2020-11-03 | NA |
| England/QEUH-B1438D/2020 | 2020-11-03 | NA |
| England/QEUH-B30B74/2020 | 2020-11-03 | NA |
| England/QEUH-B14323/2020 | 2020-11-03 | NA |
| England/QEUH-B30B1A/2020 | 2020-11-03 | NA |
| England/QEUH-B2E265/2020 | 2020-11-03 | NA |
| England/QEUH-B14129/2020 | 2020-11-03 | NA |
| England/QEUH-B2EC76/2020 | 2020-11-03 | NA |
| England/QEUH-B2EEAD/2020 | 2020-11-03 | NA |
| England/QEUH-B2D767/2020 | 2020-11-03 | NA |
| England/QEUH-B2D9DA/2020 | 2020-11-03 | NA |
| England/QEUH-B2DDED/2020 | 2020-11-03 | NA |
| England/QEUH-B2EEBC/2020 | 2020-11-03 | NA |
| England/CAMC-B21341/2020 | 2020-11-03 | NA |
| England/QEUH-B2F941/2020 | 2020-11-03 | NA |
| England/QEUH-B1443F/2020 | 2020-11-03 | NA |
| England/QEUH-B2F316/2020 | 2020-11-03 | NA |
| Scotland/QEUH-B12FA9/2020 | 2020-11-03 | NA |
| England/QEUH-B2EDDD/2020 | 2020-11-03 | NA |
| Scotland/QEUH-B13263/2020 | 2020-11-03 | NA |
| England/LIVE-DC0BB3/2020 | 2020-11-03 | NA |
| England/QEUH-B2D891/2020 | 2020-11-03 | NA |
| England/ALDP-B271E7/2020 | 2020-11-04 | NA |
| England/ALDP-B26072/2020 | 2020-11-04 | NA |
| England/QEUH-B32170/2020 | 2020-11-04 | NA |
| England/QEUH-B2EB1F/2020 | 2020-11-04 | NA |
| England/QEUH-B2C7A4/2020 | 2020-11-04 | NA |
| England/QEUH-B2C759/2020 | 2020-11-04 | NA |
| England/QEUH-B32143/2020 | 2020-11-04 | NA |
| England/ALDP-B24B19/2020 | 2020-11-04 | NA |
| England/EXET-139AD2/2020 | 2020-11-04 | NA |
| England/QEUH-B2C16A/2020 | 2020-11-04 | NA |
| England/QEUH-B2BF61/2020 | 2020-11-04 | NA |
| England/QEUH-B3191F/2020 | 2020-11-04 | NA |
| England/QEUH-B318B8/2020 | 2020-11-04 | NA |
| England/ALDP-B23822/2020 | 2020-11-04 | NA |
| England/EXET-138BC1/2020 | 2020-11-04 | NA |
| Wales/PHWC-48D122/2020 | 2020-11-04 | NA |
| Wales/PHWC-48B18E/2020 | 2020-11-04 | NA |
| England/QEUH-B2D266/2020 | 2020-11-04 | NA |
| England/QEUH-B2D2C0/2020 | 2020-11-04 | NA |
| England/ALDP-B24F77/2020 | 2020-11-04 | NA |
| England/ALDP-B2493D/2020 | 2020-11-04 | NA |
| England/QEUH-B2D248/2020 | 2020-11-04 | NA |
| England/QEUH-B2D284/2020 | 2020-11-04 | NA |
| England/EXET-139C90/2020 | 2020-11-04 | NA |
| England/QEUH-B2D5C7/2020 | 2020-11-04 | NA |
| England/QEUH-B2D59A/2020 | 2020-11-04 | NA |
| England/ALDP-B23F1E/2020 | 2020-11-04 | NA |
| England/QEUH-B2C847/2020 | 2020-11-04 | NA |
| England/QEUH-B2EC58/2020 | 2020-11-04 | NA |
| England/QEUH-B2EBD3/2020 | 2020-11-04 | NA |
| England/QEUH-B2D239/2020 | 2020-11-04 | NA |
| England/QEUH-B31C9E/2020 | 2020-11-04 | NA |
| England/QEUH-B2EB6A/2020 | 2020-11-04 | NA |
| Scotland/QEUH-B31BCE/2020 | 2020-11-04 | NA |
| England/QEUH-B2EC2B/2020 | 2020-11-04 | NA |
| England/QEUH-B2EB2E/2020 | 2020-11-04 | NA |
| England/QEUH-B302CA/2020 | 2020-11-04 | NA |
| England/QEUH-B31C61/2020 | 2020-11-04 | NA |
| England/MILK-B56DDC/2020 | 2020-11-04 | NA |
| England/ALDP-B24609/2020 | 2020-11-04 | NA |
| England/ALDP-B245FD/2020 | 2020-11-04 | NA |
| England/MILK-B56A7B/2020 | 2020-11-04 | NA |
| Wales/ALDP-B24B91/2020 | 2020-11-04 | NA |
| England/QEUH-B31BEC/2020 | 2020-11-04 | NA |
| England/LOND-DB350/2020 | 2020-11-04 | NA |
| England/QEUH-B302E8/2020 | 2020-11-04 | NA |
| England/QEUH-B2D336/2020 | 2020-11-04 | NA |
| England/QEUH-B3034F/2020 | 2020-11-04 | NA |
| England/QEUH-B30312/2020 | 2020-11-04 | NA |
| England/LIVE-DC0A4D/2020 | 2020-11-04 | NA |
| England/QEUH-B32055/2020 | 2020-11-04 | NA |
| England/ALDP-B23743/2020 | 2020-11-04 | NA |
| England/QEUH-B31A85/2020 | 2020-11-04 | NA |
| England/PHEC-14BC04/2020 | 2020-11-04 | NA |
| England/ALDP-B242F6/2020 | 2020-11-04 | NA |
| England/ALDP-B2485E/2020 | 2020-11-04 | NA |
| England/QEUH-B2D3FA/2020 | 2020-11-04 | NA |
| Scotland/QEUH-B3188B/2020 | 2020-11-04 | NA |
| England/QEUH-B321AD/2020 | 2020-11-04 | NA |
| England/ALDP-B23BB0/2020 | 2020-11-04 | NA |
| England/ALDP-B240ED/2020 | 2020-11-04 | NA |
| England/QEUH-B2DF8D/2020 | 2020-11-04 | NA |
| England/ALDP-B23C35/2020 | 2020-11-04 | NA |
| England/ALDP-B24DF5/2020 | 2020-11-04 | NA |
| England/ALDP-B23F96/2020 | 2020-11-04 | NA |
| Wales/ALDP-B24F68/2020 | 2020-11-04 | NA |
| England/CAMC-B375BB/2020 | 2020-11-05 | NA |
| Wales/PHWC-48DC21/2020 | 2020-11-05 | NA |
| Wales/PHWC-48DCC7/2020 | 2020-11-05 | NA |
| England/MILK-B553F9/2020 | 2020-11-05 | NA |
| England/MILK-B54AB9/2020 | 2020-11-05 | NA |
| England/CAMC-B360CA/2020 | 2020-11-05 | NA |
| England/CAMC-B37463/2020 | 2020-11-05 | NA |
| England/CAMC-B37E38/2020 | 2020-11-05 | NA |
| England/CAMC-B32E3D/2020 | 2020-11-05 | NA |
| England/CAMC-B337D7/2020 | 2020-11-05 | NA |
| England/CAMC-B3467F/2020 | 2020-11-05 | NA |
| England/CAMC-B330AF/2020 | 2020-11-05 | NA |
| England/CAMC-B32B18/2020 | 2020-11-05 | NA |
| England/CAMC-B36A26/2020 | 2020-11-05 | NA |
| England/CAMC-B325FC/2020 | 2020-11-05 | NA |
| England/CAMB-1B97F1/2020 | 2020-11-05 | NA |
| England/CAMB-1B983A/2020 | 2020-11-05 | NA |
| England/CAMB-1B981C/2020 | 2020-11-05 | NA |
| England/CAMB-1B982B/2020 | 2020-11-05 | NA |
| England/CAMC-B340BD/2020 | 2020-11-05 | NA |
| England/CAMC-B363B2/2020 | 2020-11-05 | NA |
| England/CAMC-B3387A/2020 | 2020-11-05 | NA |
| England/MILK-B5645F/2020 | 2020-11-05 | NA |
| England/CAMC-B3279C/2020 | 2020-11-05 | NA |
| England/MILK-B55BE2/2020 | 2020-11-05 | NA |
| England/CAMC-B338F2/2020 | 2020-11-05 | NA |
| England/CAMC-B33E4B/2020 | 2020-11-05 | NA |
| England/MILK-B54CE0/2020 | 2020-11-05 | NA |
| England/CAMC-B324B3/2020 | 2020-11-05 | NA |
| England/CAMC-B32FA3/2020 | 2020-11-05 | NA |
| England/MILK-B54EDB/2020 | 2020-11-06 | NA |
| England/MILK-B56B1E/2020 | 2020-11-06 | NA |
| England/ALDP-B46D48/2020 | 2020-11-06 | NA |
| England/LOND-12638FF/2020 | 2020-11-06 | NA |
| England/ALDP-B46B8A/2020 | 2020-11-06 | NA |
| England/MILK-B5A2E8/2020 | 2020-11-06 | NA |
| England/MILK-B5697E/2020 | 2020-11-06 | NA |
| England/CAMC-B543FA/2020 | 2020-11-06 | NA |
| England/NORT-287755/2020 | 2020-11-06 | NA |
| England/CAMC-B51AF8/2020 | 2020-11-06 | NA |
| England/LIVE-DB627F/2020 | 2020-11-06 | NA |
| Scotland/EDB9324/2020 | 2020-11-06 | NA |
| England/NORT-1B70744/2020 | 2020-11-06 | NA |
| England/CAMC-B5448E/2020 | 2020-11-06 | NA |
| England/MILK-B598AB/2020 | 2020-11-06 | NA |
| England/CAMC-B538B0/2020 | 2020-11-06 | NA |
| England/ALDP-B4864B/2020 | 2020-11-06 | NA |
| England/CAMC-B350F8/2020 | 2020-11-06 | NA |
| England/CAMC-B34509/2020 | 2020-11-06 | NA |
| England/CAMC-B32CAC/2020 | 2020-11-06 | NA |
| England/MILK-B55775/2020 | 2020-11-06 | NA |
| England/MILK-B5A79E/2020 | 2020-11-06 | NA |
| England/MILK-B5A770/2020 | 2020-11-06 | NA |
| England/MILK-B5A78F/2020 | 2020-11-06 | NA |
| Wales/PHWC-48CBF8/2020 | 2020-11-07 | NA |
| England/ALDP-B47EAE/2020 | 2020-11-07 | NA |
| England/ALDP-B47E9F/2020 | 2020-11-07 | NA |
| England/ALDP-B48757/2020 | 2020-11-07 | NA |
| Wales/ALDP-B49237/2020 | 2020-11-07 | NA |
| England/ALDP-B4872A/2020 | 2020-11-07 | NA |
| England/ALDP-B46759/2020 | 2020-11-07 | NA |
| England/ALDP-B45A60/2020 | 2020-11-07 | NA |
| England/ALDP-B462D0/2020 | 2020-11-07 | NA |
| England/ALDP-B46E09/2020 | 2020-11-07 | NA |
| England/ALDP-B48FE6/2020 | 2020-11-07 | NA |
| England/ALDP-B46012/2020 | 2020-11-07 | NA |
| England/ALDP-B46BC6/2020 | 2020-11-07 | NA |
| England/ALDP-B47FD8/2020 | 2020-11-07 | NA |
| England/ALDP-B49352/2020 | 2020-11-07 | NA |
| England/ALDP-B47F41/2020 | 2020-11-07 | NA |
| England/ALDP-B48EAD/2020 | 2020-11-07 | NA |
| England/ALDP-B46E90/2020 | 2020-11-07 | NA |
| England/ALDP-B46F33/2020 | 2020-11-07 | NA |
| England/ALDP-B46F06/2020 | 2020-11-07 | NA |
| England/CAMC-B54855/2020 | 2020-11-08 | NA |
| England/CAMC-B546E2/2020 | 2020-11-08 | NA |
| England/CAMC-B51E47/2020 | 2020-11-08 | NA |
| England/CAMC-B522A4/2020 | 2020-11-08 | NA |
| England/ALDP-B5B8A9/2020 | 2020-11-08 | NA |
| England/QEUH-B4142A/2020 | 2020-11-08 | NA |
| England/ALDP-B62AD6/2020 | 2020-11-08 | NA |
| England/QEUH-B4367D/2020 | 2020-11-08 | NA |
| England/NORT-1B70683/2020 | 2020-11-08 | NA |
| England/CAMC-B51D1D/2020 | 2020-11-08 | NA |
| England/QEUH-B4241A/2020 | 2020-11-08 | NA |
| England/QEUH-B42474/2020 | 2020-11-08 | NA |
| England/ALDP-B62FB9/2020 | 2020-11-08 | NA |
| England/QEUH-B423D1/2020 | 2020-11-08 | NA |
| England/QEUH-B42456/2020 | 2020-11-08 | NA |
| England/QEUH-B3FF97/2020 | 2020-11-08 | NA |
| England/QEUH-B400DC/2020 | 2020-11-08 | NA |
| England/QEUH-B40027/2020 | 2020-11-08 | NA |
| England/QEUH-B3F735/2020 | 2020-11-08 | NA |
| England/QEUH-B3F6B0/2020 | 2020-11-08 | NA |
| England/CAMC-B51F53/2020 | 2020-11-08 | NA |
| England/ALDP-B63B0F/2020 | 2020-11-08 | NA |
| England/ALDP-B5B5B1/2020 | 2020-11-08 | NA |
| England/ALDP-B625C6/2020 | 2020-11-08 | NA |
| England/ALDP-B62520/2020 | 2020-11-08 | NA |
| England/QEUH-B432E2/2020 | 2020-11-08 | NA |
| England/QEUH-B40106/2020 | 2020-11-08 | NA |
| England/QEUH-B4078C/2020 | 2020-11-08 | NA |
| England/CAMB-1B9AE9/2020 | 2020-11-08 | NA |
| England/ALDP-B62CA3/2020 | 2020-11-08 | NA |
| England/ALDP-B62CB2/2020 | 2020-11-08 | NA |
| England/QEUH-B43428/2020 | 2020-11-08 | NA |
| England/QEUH-B42395/2020 | 2020-11-08 | NA |
| England/QEUH-B3FFE2/2020 | 2020-11-08 | NA |
| England/QEUH-B423C2/2020 | 2020-11-08 | NA |
| England/PORT-2E93C6/2020 | 2020-11-08 | NA |
| England/ALDP-B5CC8E/2020 | 2020-11-08 | NA |
| England/ALDP-B5CCE8/2020 | 2020-11-08 | NA |
| England/QEUH-B3F46B/2020 | 2020-11-08 | NA |
| England/CAMC-B519DD/2020 | 2020-11-08 | NA |
| England/CAMC-B53F8E/2020 | 2020-11-08 | NA |
| England/CAMC-B54776/2020 | 2020-11-08 | NA |
| England/QEUH-B3FD33/2020 | 2020-11-08 | NA |
| England/QEUH-B416AC/2020 | 2020-11-08 | NA |
| England/QEUH-B40160/2020 | 2020-11-08 | NA |
| England/QEUH-B4017F/2020 | 2020-11-08 | NA |
| England/CAMC-B51D2C/2020 | 2020-11-08 | NA |
| England/QEUH-B40FC0/2020 | 2020-11-08 | NA |
| England/ALDP-B62E52/2020 | 2020-11-08 | NA |
| England/CAMC-B542C0/2020 | 2020-11-08 | NA |
| England/ALDP-B5B584/2020 | 2020-11-08 | NA |
| England/ALDP-B63B2D/2020 | 2020-11-08 | NA |
| England/QEUH-B41721/2020 | 2020-11-08 | NA |
| England/QEUH-B3F29E/2020 | 2020-11-08 | NA |
| England/ALDP-B62CC1/2020 | 2020-11-08 | NA |
| England/ALDP-B62BA6/2020 | 2020-11-08 | NA |
| England/QEUH-B402A9/2020 | 2020-11-08 | NA |
| England/ALDP-B5E299/2020 | 2020-11-08 | NA |
| England/QEUH-B418F1/2020 | 2020-11-08 | NA |
| England/ALDP-B5B6BE/2020 | 2020-11-08 | NA |
| England/ALDP-B62E25/2020 | 2020-11-08 | NA |
| England/ALDP-B630C4/2020 | 2020-11-08 | NA |
| England/ALDP-B63088/2020 | 2020-11-08 | NA |
| Denmark/DCGC-10036/2020 | 2020-11-09 | NA |
| Denmark/DCGC-10232/2020 | 2020-11-09 | NA |
| Portugal/PT1836/2020 | 2020-11-09 | NA |
| England/CAMC-B540F3/2020 | 2020-11-09 | NA |
| England/NORT-1B61728/2020 | 2020-11-09 | NA |
| England/ALDP-B5BBEC/2020 | 2020-11-09 | NA |
| England/ALDP-B5BBDD/2020 | 2020-11-09 | NA |
| England/NORT-1B70CEB/2020 | 2020-11-09 | NA |
| England/NORT-1B70902/2020 | 2020-11-09 | NA |
| England/NORT-1B7094E/2020 | 2020-11-09 | NA |
| England/WSFT-25C6108/2020 | 2020-11-09 | NA |
| England/ALDP-B62942/2020 | 2020-11-09 | NA |
| England/LOND-1287330/2020 | 2020-11-09 | NA |
| England/BIRM-6A24A/2020 | 2020-11-09 | NA |
| England/BIRM-6A2FF/2020 | 2020-11-09 | NA |
| England/CAMC-B53030/2020 | 2020-11-09 | NA |
| England/CAMC-B5308B/2020 | 2020-11-09 | NA |
| England/CAMC-B5499E/2020 | 2020-11-09 | NA |
| England/ALDP-B5E6BB/2020 | 2020-11-09 | NA |
| England/CAMC-B52A7F/2020 | 2020-11-09 | NA |
| England/CAMC-B54907/2020 | 2020-11-09 | NA |
| England/CAMC-B549E9/2020 | 2020-11-09 | NA |
| England/CAMC-B54952/2020 | 2020-11-09 | NA |
| England/CAMC-B549AD/2020 | 2020-11-09 | NA |
| England/ALDP-B5C626/2020 | 2020-11-09 | NA |
| England/ALDP-B5B742/2020 | 2020-11-09 | NA |
| England/QEUH-B677F8/2020 | 2020-11-09 | NA |
| England/ALDP-B5AF3C/2020 | 2020-11-09 | NA |
| Wales/PHWC-490FAA/2020 | 2020-11-09 | NA |
| England/ALDP-B5BEF2/2020 | 2020-11-09 | NA |
| England/ALDP-B5BE10/2020 | 2020-11-09 | NA |
| Wales/PHWC-49B2E2/2020 | 2020-11-09 | NA |
| England/ALDP-B5E17E/2020 | 2020-11-09 | NA |
| England/ALDP-B629F7/2020 | 2020-11-09 | NA |
| England/ALDP-B62353/2020 | 2020-11-09 | NA |
| England/ALDP-B5B4A5/2020 | 2020-11-09 | NA |
| England/ALDP-B5E132/2020 | 2020-11-09 | NA |
| England/CAMB-1BAECE/2020 | 2020-11-10 | NA |
| England/QEUH-B662E9/2020 | 2020-11-10 | NA |
| England/QEUH-B662DA/2020 | 2020-11-10 | NA |
| England/CAMC-B79993/2020 | 2020-11-10 | NA |
| England/NORT-1B7093F/2020 | 2020-11-10 | NA |
| England/NORT-1B70A69/2020 | 2020-11-10 | NA |
| England/CAMB-1BA884/2020 | 2020-11-10 | NA |
| Scotland/CVR5786/2020 | 2020-11-10 | NA |
| England/NORT-287CB0/2020 | 2020-11-10 | NA |
| England/CAMC-B7B102/2020 | 2020-11-10 | NA |
| England/LIVE-DC479D/2020 | 2020-11-10 | NA |
| England/LIVE-DC47E8/2020 | 2020-11-10 | NA |
| England/LIVE-DC46CD/2020 | 2020-11-10 | NA |
| England/QEUH-B67822/2020 | 2020-11-10 | NA |
| Portugal/PT1776/2020 | 2020-11-11 | NA |
| England/CAMB-1BAABB/2020 | 2020-11-11 | NA |
| England/BIRM-6AA33/2020 | 2020-11-11 | NA |
| England/QEUH-B69F94/2020 | 2020-11-11 | NA |
| England/CAMC-B79A54/2020 | 2020-11-11 | NA |
| England/QEUH-B6895B/2020 | 2020-11-11 | NA |
| England/QEUH-B66410/2020 | 2020-11-11 | NA |
| England/QEUH-B6760A/2020 | 2020-11-11 | NA |
| England/QEUH-B6751C/2020 | 2020-11-11 | NA |
| England/QEUH-B6C975/2020 | 2020-11-11 | NA |
| England/QEUH-B66A1E/2020 | 2020-11-11 | NA |
| England/QEUH-B69E5B/2020 | 2020-11-11 | NA |
| England/QEUH-B665B3/2020 | 2020-11-11 | NA |
| England/CAMC-B9A46E/2020 | 2020-11-11 | NA |
| England/QEUH-B66586/2020 | 2020-11-11 | NA |
| Wales/PHWC-48FC6B/2020 | 2020-11-11 | NA |
| England/QEUH-B66F97/2020 | 2020-11-11 | NA |
| Wales/QEUH-B6C140/2020 | 2020-11-11 | NA |
| Scotland/QEUH-B6A661/2020 | 2020-11-11 | NA |
| England/QEUH-B68E4D/2020 | 2020-11-11 | NA |
| England/QEUH-B691F8/2020 | 2020-11-11 | NA |
| England/QEUH-B691DA/2020 | 2020-11-11 | NA |
| England/QEUH-B6C957/2020 | 2020-11-11 | NA |
| England/QEUH-B6C92A/2020 | 2020-11-11 | NA |
| England/QEUH-B6A1F7/2020 | 2020-11-11 | NA |
| England/QEUH-B6B6E8/2020 | 2020-11-11 | NA |
| England/QEUH-B67A86/2020 | 2020-11-11 | NA |
| England/QEUH-B679F2/2020 | 2020-11-11 | NA |
| England/QEUH-B6B68E/2020 | 2020-11-11 | NA |
| England/QEUH-B6992D/2020 | 2020-11-11 | NA |
| England/QEUH-B699A5/2020 | 2020-11-11 | NA |
| England/QEUH-B67567/2020 | 2020-11-11 | NA |
| Scotland/QEUH-B675A3/2020 | 2020-11-11 | NA |
| England/QEUH-B698B7/2020 | 2020-11-11 | NA |
| England/QEUH-B69987/2020 | 2020-11-11 | NA |
| England/QEUH-B6C535/2020 | 2020-11-11 | NA |
| England/QEUH-B6A564/2020 | 2020-11-11 | NA |
| England/QEUH-B675B2/2020 | 2020-11-11 | NA |
| England/CAMC-B9AE06/2020 | 2020-11-11 | NA |
| England/QEUH-B66A5A/2020 | 2020-11-11 | NA |
| England/QEUH-B66AA5/2020 | 2020-11-11 | NA |
| England/QEUH-B6A115/2020 | 2020-11-11 | NA |
| England/QEUH-B6A151/2020 | 2020-11-11 | NA |
| England/QEUH-B6A106/2020 | 2020-11-11 | NA |
| England/QEUH-B6AE1E/2020 | 2020-11-11 | NA |
| England/QEUH-B6643E/2020 | 2020-11-11 | NA |
| England/QEUH-B6894C/2020 | 2020-11-11 | NA |
| England/NORW-F28EB/2020 | 2020-11-11 | NA |
| England/QEUH-B6702A/2020 | 2020-11-11 | NA |
| England/QEUH-B6686F/2020 | 2020-11-11 | NA |
| England/QEUH-B668F6/2020 | 2020-11-11 | NA |
| England/QEUH-B668D8/2020 | 2020-11-11 | NA |
| England/QEUH-B697D8/2020 | 2020-11-11 | NA |
| England/QEUH-B677DA/2020 | 2020-11-11 | NA |
| England/QEUH-B67761/2020 | 2020-11-11 | NA |
| England/QEUH-B677BC/2020 | 2020-11-11 | NA |
| England/QEUH-B6A555/2020 | 2020-11-11 | NA |
| England/QEUH-B6A50A/2020 | 2020-11-11 | NA |
| England/QEUH-B676CE/2020 | 2020-11-11 | NA |
| England/QEUH-B6B9B2/2020 | 2020-11-11 | NA |
| England/QEUH-B6BE2C/2020 | 2020-11-11 | NA |
| England/QEUH-B6BE59/2020 | 2020-11-11 | NA |
| England/QEUH-B6B09F/2020 | 2020-11-11 | NA |
| England/QEUH-B69EA6/2020 | 2020-11-11 | NA |
| England/QEUH-B69C60/2020 | 2020-11-11 | NA |
| England/QEUH-B6752B/2020 | 2020-11-11 | NA |
| England/QEUH-B67CBD/2020 | 2020-11-11 | NA |
| England/QEUH-B6C0AD/2020 | 2020-11-11 | NA |
| England/QEUH-B684C3/2020 | 2020-11-11 | NA |
| Wales/PHWC-48FCC5/2020 | 2020-11-11 | NA |
| Portugal/PT1668/2020 | 2020-11-12 | NA |
| England/MILK-B8C64B/2020 | 2020-11-12 | NA |
| England/PORT-2EA34D/2020 | 2020-11-12 | NA |
| England/CAMC-B7769E/2020 | 2020-11-12 | NA |
| England/CAMC-B7A033/2020 | 2020-11-12 | NA |
| England/ALDP-B735B3/2020 | 2020-11-12 | NA |
| Wales/PHWC-48D535/2020 | 2020-11-12 | NA |
| England/CAMC-B7B515/2020 | 2020-11-12 | NA |
| England/CAMC-B790CB/2020 | 2020-11-12 | NA |
| England/ALDP-B746DD/2020 | 2020-11-12 | NA |
| England/ALDP-B73717/2020 | 2020-11-12 | NA |
| England/ALDP-B75548/2020 | 2020-11-12 | NA |
| England/ALDP-B73AC3/2020 | 2020-11-12 | NA |
| England/ALDP-B73B57/2020 | 2020-11-12 | NA |
| England/CAMC-B77D3F/2020 | 2020-11-12 | NA |
| England/CAMC-B7AC5D/2020 | 2020-11-12 | NA |
| England/CAMC-B79F73/2020 | 2020-11-12 | NA |
| England/CAMC-B7B5CA/2020 | 2020-11-12 | NA |
| England/ALDP-B751DB/2020 | 2020-11-12 | NA |
| England/CAMC-B7981E/2020 | 2020-11-12 | NA |
| England/ALDP-B74CAE/2020 | 2020-11-12 | NA |
| England/ALDP-B768C6/2020 | 2020-11-12 | NA |
| England/ALDP-B74CCC/2020 | 2020-11-12 | NA |
| England/ALDP-B7543C/2020 | 2020-11-12 | NA |
| England/ALDP-B75F95/2020 | 2020-11-12 | NA |
| England/ALDP-B761DA/2020 | 2020-11-12 | NA |
| England/ALDP-B742D9/2020 | 2020-11-12 | NA |
| England/ALDP-B749A7/2020 | 2020-11-12 | NA |
| England/ALDP-B73C27/2020 | 2020-11-12 | NA |
| England/ALDP-B7478F/2020 | 2020-11-12 | NA |
| England/NOTT-11B7DF/2020 | 2020-11-12 | NA |
| Wales/PHWC-48E7C5/2020 | 2020-11-12 | NA |
| England/ALDP-B7695A/2020 | 2020-11-12 | NA |
| England/ALDP-B75645/2020 | 2020-11-12 | NA |
| England/CAMC-B7A552/2020 | 2020-11-12 | NA |
| England/ALDP-B768E4/2020 | 2020-11-12 | NA |
| England/ALDP-B7690F/2020 | 2020-11-12 | NA |
| England/ALDP-B75056/2020 | 2020-11-12 | NA |
| England/ALDP-B758E5/2020 | 2020-11-12 | NA |
| England/ALDP-B73AA5/2020 | 2020-11-12 | NA |
| England/LIVE-DC4CF8/2020 | 2020-11-12 | NA |
| England/ALDP-B762F5/2020 | 2020-11-12 | NA |
| England/ALDP-B73D8E/2020 | 2020-11-12 | NA |
| England/ALDP-B76A1B/2020 | 2020-11-12 | NA |
| England/ALDP-B735A4/2020 | 2020-11-12 | NA |
| England/ALDP-B7360B/2020 | 2020-11-12 | NA |
| England/ALDP-B73C81/2020 | 2020-11-12 | NA |
| England/ALDP-B75575/2020 | 2020-11-12 | NA |
| Portugal/PT2050/2020 | 2020-11-13 | NA |
| England/MILK-B9518A/2020 | 2020-11-13 | NA |
| England/MILK-B8F8E8/2020 | 2020-11-13 | NA |
| England/MILK-B8F8D9/2020 | 2020-11-13 | NA |
| England/MILK-B8D2BF/2020 | 2020-11-13 | NA |
| England/MILK-B8D2CE/2020 | 2020-11-13 | NA |
| England/CAMB-1BBD84/2020 | 2020-11-13 | NA |
| England/MILK-B8EBA4/2020 | 2020-11-13 | NA |
| England/MILK-B8EEC9/2020 | 2020-11-13 | NA |
| England/ALDP-B8432E/2020 | 2020-11-13 | NA |
| England/CAMC-B7AD96/2020 | 2020-11-13 | NA |
| England/PORT-2EA3A7/2020 | 2020-11-13 | NA |
| England/MILK-B86BAC/2020 | 2020-11-13 | NA |
| England/MILK-B8D2FB/2020 | 2020-11-13 | NA |
| England/MILK-B8D343/2020 | 2020-11-13 | NA |
| England/MILK-B85DB6/2020 | 2020-11-13 | NA |
| England/MILK-B85DA7/2020 | 2020-11-13 | NA |
| England/MILK-B8F88E/2020 | 2020-11-13 | NA |
| Wales/PHWC-48D553/2020 | 2020-11-13 | NA |
| Wales/LIVE-DBB210/2020 | 2020-11-13 | NA |
| England/PHEC-14D2D3/2020 | 2020-11-13 | NA |
| England/MILK-B8CA7C/2020 | 2020-11-13 | NA |
| England/QEUH-B7DF06/2020 | 2020-11-13 | NA |
| England/MILK-B8F217/2020 | 2020-11-13 | NA |
| England/MILK-B89DEF/2020 | 2020-11-13 | NA |
| England/MILK-B8F3AB/2020 | 2020-11-13 | NA |
| England/QEUH-B2ED28/2020 | 2020-11-13 | NA |
| England/MILK-B9620E/2020 | 2020-11-13 | NA |
| England/ALDP-B85897/2020 | 2020-11-13 | NA |
| England/CAMC-B78ED1/2020 | 2020-11-13 | NA |
| England/MILK-B952C3/2020 | 2020-11-13 | NA |
| England/MILK-B95B40/2020 | 2020-11-13 | NA |
| England/MILK-B88CA7/2020 | 2020-11-13 | NA |
| England/MILK-B88C98/2020 | 2020-11-13 | NA |
| England/MILK-B88C5C/2020 | 2020-11-13 | NA |
| England/MILK-B8E6C1/2020 | 2020-11-13 | NA |
| England/CAMC-B79984/2020 | 2020-11-13 | NA |
| England/CAMC-B79966/2020 | 2020-11-13 | NA |
| England/MILK-B8ECB0/2020 | 2020-11-13 | NA |
| England/MILK-B8D3DA/2020 | 2020-11-13 | NA |
| England/MILK-B8D5E3/2020 | 2020-11-13 | NA |
| England/MILK-B8C502/2020 | 2020-11-13 | NA |
| England/MILK-B8CA9A/2020 | 2020-11-13 | NA |
| England/CAMB-1BBA9C/2020 | 2020-11-13 | NA |
| England/QEUH-B7C383/2020 | 2020-11-13 | NA |
| England/MILK-B8D307/2020 | 2020-11-13 | NA |
| England/MILK-B959A0/2020 | 2020-11-13 | NA |
| England/MILK-B8CB5B/2020 | 2020-11-13 | NA |
| England/MILK-B8EBB3/2020 | 2020-11-13 | NA |
| England/MILK-B8EC0B/2020 | 2020-11-13 | NA |
| England/NORT-1B710EE/2020 | 2020-11-13 | NA |
| England/MILK-B95515/2020 | 2020-11-13 | NA |
| England/MILK-B974F8/2020 | 2020-11-14 | NA |
| England/ALDP-B839E1/2020 | 2020-11-14 | NA |
| England/CAMB-1BA4EA/2020 | 2020-11-14 | NA |
| England/PORT-2EE68C/2020 | 2020-11-14 | NA |
| England/ALDP-B8427C/2020 | 2020-11-14 | NA |
| England/QEUH-B7DAE7/2020 | 2020-11-14 | NA |
| England/QEUH-B7D67A/2020 | 2020-11-14 | NA |
| England/QEUH-B7C15C/2020 | 2020-11-14 | NA |
| England/MILK-B9612F/2020 | 2020-11-14 | NA |
| England/QEUH-B7FD37/2020 | 2020-11-14 | NA |
| England/QEUH-B7D6A7/2020 | 2020-11-14 | NA |
| England/ALDP-B858A6/2020 | 2020-11-14 | NA |
| England/QEUH-B7EF6F/2020 | 2020-11-14 | NA |
| England/QEUH-B7D337/2020 | 2020-11-14 | NA |
| England/QEUH-B7D768/2020 | 2020-11-14 | NA |
| England/QEUH-B7EA22/2020 | 2020-11-14 | NA |
| England/QEUH-B7E9E9/2020 | 2020-11-14 | NA |
| England/QEUH-B7EA31/2020 | 2020-11-14 | NA |
| England/QEUH-B7D07C/2020 | 2020-11-14 | NA |
| England/QEUH-B7FDFB/2020 | 2020-11-14 | NA |
| England/QEUH-B7E8A0/2020 | 2020-11-14 | NA |
| England/QEUH-B7DC2D/2020 | 2020-11-14 | NA |
| England/QEUH-B7D513/2020 | 2020-11-14 | NA |
| England/QEUH-B7DC5A/2020 | 2020-11-14 | NA |
| England/QEUH-B7C63F/2020 | 2020-11-14 | NA |
| England/QEUH-B7C07D/2020 | 2020-11-14 | NA |
| England/QEUH-B7C936/2020 | 2020-11-14 | NA |
| England/QEUH-B7F3CC/2020 | 2020-11-14 | NA |
| England/ALDP-B85A73/2020 | 2020-11-14 | NA |
| England/ALDP-B85AA0/2020 | 2020-11-14 | NA |
| England/QEUH-B7D6F2/2020 | 2020-11-14 | NA |
| England/QEUH-B7D65C/2020 | 2020-11-14 | NA |
| England/QEUH-B7D71D/2020 | 2020-11-14 | NA |
| England/QEUH-B7C110/2020 | 2020-11-14 | NA |
| England/QEUH-B7F4C9/2020 | 2020-11-14 | NA |
| England/QEUH-B7EB10/2020 | 2020-11-14 | NA |
| England/QEUH-B80C09/2020 | 2020-11-14 | NA |
| England/ALDP-B83D30/2020 | 2020-11-14 | NA |
| England/QEUH-B7ECA4/2020 | 2020-11-14 | NA |
| England/ALDP-B83B45/2020 | 2020-11-14 | NA |
| England/ALDP-B842C7/2020 | 2020-11-14 | NA |
| England/QEUH-B7F20B/2020 | 2020-11-14 | NA |
| England/QEUH-B7FE07/2020 | 2020-11-14 | NA |
| England/ALDP-B8387B/2020 | 2020-11-14 | NA |
| England/QEUH-B809A8/2020 | 2020-11-14 | NA |
| England/QEUH-B7C6B7/2020 | 2020-11-14 | NA |
| England/ALDP-B83A1B/2020 | 2020-11-14 | NA |
| England/ALDP-B839D2/2020 | 2020-11-14 | NA |
| England/ALDP-B839F0/2020 | 2020-11-14 | NA |
| England/MILK-B97373/2020 | 2020-11-14 | NA |
| England/ALDP-B84E4B/2020 | 2020-11-14 | NA |
| England/LIVE-DC437B/2020 | 2020-11-14 | NA |
| England/QEUH-B7C909/2020 | 2020-11-14 | NA |
| England/CAMC-B9A54D/2020 | 2020-11-15 | NA |
| England/NORT-28D27C/2020 | 2020-11-15 | NA |
| England/NORT-1B71321/2020 | 2020-11-15 | NA |
| England/ALDP-B84722/2020 | 2020-11-15 | NA |
| England/ALDP-B851BA/2020 | 2020-11-15 | NA |
| England/NORT-1B71190/2020 | 2020-11-15 | NA |
| England/CAMC-B9AEAC/2020 | 2020-11-15 | NA |
| England/ALDP-B8525D/2020 | 2020-11-15 | NA |
| England/NORT-28D230/2020 | 2020-11-15 | NA |
| England/CAMC-B98776/2020 | 2020-11-15 | NA |
| England/CAMC-B9870D/2020 | 2020-11-15 | NA |
| England/CAMC-B99EAD/2020 | 2020-11-15 | NA |
| England/CAMC-B9871C/2020 | 2020-11-15 | NA |
| England/CAMC-B9A6D1/2020 | 2020-11-15 | NA |
| England/CAMC-B982B1/2020 | 2020-11-15 | NA |
| England/ALDP-B85536/2020 | 2020-11-15 | NA |
| England/ALDP-B84A0B/2020 | 2020-11-15 | NA |
| England/ALDP-B84F2A/2020 | 2020-11-15 | NA |
| Denmark/DCGC-12576/2020 | 2020-11-16 | NA |
| Portugal/PT1779/2020 | 2020-11-16 | NA |
| England/CAMB-1BBC69/2020 | 2020-11-16 | NA |
| England/CAMB-1BBB3F/2020 | 2020-11-16 | NA |
| England/NORT-28D31F/2020 | 2020-11-16 | NA |
| England/NORT-1B71AD1/2020 | 2020-11-16 | NA |
| England/NORT-1B71AE0/2020 | 2020-11-16 | NA |
| England/NORT-1B71AC2/2020 | 2020-11-16 | NA |
| England/NORT-1B71A95/2020 | 2020-11-16 | NA |
| England/NORT-1B71AA4/2020 | 2020-11-16 | NA |
| England/NORT-28D2E5/2020 | 2020-11-16 | NA |
| England/NORT-28D2D6/2020 | 2020-11-16 | NA |
| England/NORT-28D35B/2020 | 2020-11-16 | NA |
| England/MILK-BA9B0D/2020 | 2020-11-17 | NA |
| England/MILK-BA9B1C/2020 | 2020-11-17 | NA |
| England/NORT-28D546/2020 | 2020-11-17 | NA |
| England/PHEC-14D9A1/2020 | 2020-11-17 | NA |
| England/MILK-BAA340/2020 | 2020-11-17 | NA |
| England/MILK-BB04EA/2020 | 2020-11-17 | NA |
| England/MILK-BB105E/2020 | 2020-11-17 | NA |
| England/MILK-BABC62/2020 | 2020-11-18 | NA |
| England/QEUH-B9FB91/2020 | 2020-11-18 | NA |
| England/QEUH-BA26E5/2020 | 2020-11-18 | NA |
| England/NORT-28D5FB/2020 | 2020-11-18 | NA |
| England/QEUH-B9FD7D/2020 | 2020-11-18 | NA |
| England/NORT-28D625/2020 | 2020-11-18 | NA |
| England/QEUH-BA27F1/2020 | 2020-11-18 | NA |
| England/NORT-1B719F2/2020 | 2020-11-18 | NA |
| England/QEUH-BA019B/2020 | 2020-11-18 | NA |
| England/MILK-BB265B/2020 | 2020-11-18 | NA |
| England/QEUH-B9EC35/2020 | 2020-11-18 | NA |
| England/QEUH-B9EFE1/2020 | 2020-11-18 | NA |
| England/QEUH-B9E44C/2020 | 2020-11-18 | NA |
| England/QEUH-BA3365/2020 | 2020-11-18 | NA |
| England/QEUH-B9F548/2020 | 2020-11-18 | NA |
| England/QEUH-B9F557/2020 | 2020-11-18 | NA |
| England/QEUH-BA1042/2020 | 2020-11-18 | NA |
| England/QEUH-B9E0FD/2020 | 2020-11-18 | NA |
| Wales/PHWC-494331/2020 | 2020-11-18 | NA |
| England/QEUH-BA175C/2020 | 2020-11-18 | NA |
| England/QEUH-B9F9C4/2020 | 2020-11-18 | NA |
| England/QEUH-B9F95B/2020 | 2020-11-18 | NA |
| England/QEUH-BA0113/2020 | 2020-11-18 | NA |
| England/QEUH-BA13A3/2020 | 2020-11-18 | NA |
| England/QEUH-B9F742/2020 | 2020-11-18 | NA |
| England/QEUH-B9E85F/2020 | 2020-11-18 | NA |
| England/QEUH-B9EA68/2020 | 2020-11-18 | NA |
| England/CAMB-1BB8ED/2020 | 2020-11-18 | NA |
| England/MILK-BB0040/2020 | 2020-11-18 | NA |
| England/QEUH-B9F2E7/2020 | 2020-11-18 | NA |
| England/NORT-28D670/2020 | 2020-11-18 | NA |
| England/QEUH-BA32D1/2020 | 2020-11-18 | NA |
| England/QEUH-BA3426/2020 | 2020-11-18 | NA |
| Scotland/QEUH-BA1491/2020 | 2020-11-18 | NA |
| England/QEUH-B9F083/2020 | 2020-11-18 | NA |
| England/NORT-28D643/2020 | 2020-11-18 | NA |
| England/QEUH-BA3392/2020 | 2020-11-18 | NA |
| England/QEUH-B9E770/2020 | 2020-11-18 | NA |
| England/MILK-BAB479/2020 | 2020-11-18 | NA |
| England/MILK-BAC645/2020 | 2020-11-18 | NA |
| England/QEUH-BA2700/2020 | 2020-11-18 | NA |
| England/QEUH-BA17C5/2020 | 2020-11-18 | NA |
| England/QEUH-BA1983/2020 | 2020-11-18 | NA |
| England/QEUH-B9E7AD/2020 | 2020-11-18 | NA |
| England/MILK-BAB51C/2020 | 2020-11-18 | NA |
| England/QEUH-B9E743/2020 | 2020-11-18 | NA |
| England/MILK-BAA182/2020 | 2020-11-18 | NA |
| England/QEUH-B9EA2C/2020 | 2020-11-18 | NA |
| England/QEUH-B9EB83/2020 | 2020-11-18 | NA |
| England/QEUH-B9E725/2020 | 2020-11-18 | NA |
| England/QEUH-B9F4F0/2020 | 2020-11-18 | NA |
| England/QEUH-BA27D3/2020 | 2020-11-18 | NA |
| England/MILK-BAA595/2020 | 2020-11-18 | NA |
| England/MILK-BAA5C2/2020 | 2020-11-18 | NA |
| England/MILK-BB215A/2020 | 2020-11-18 | NA |
| England/MILK-BAB7E9/2020 | 2020-11-18 | NA |
| England/QEUH-BA359C/2020 | 2020-11-18 | NA |
| England/QEUH-BA1C7B/2020 | 2020-11-18 | NA |
| England/QEUH-B9F320/2020 | 2020-11-18 | NA |
| England/QEUH-B9E057/2020 | 2020-11-18 | NA |
| England/MILK-BAA401/2020 | 2020-11-18 | NA |
| England/QEUH-B9F575/2020 | 2020-11-18 | NA |
| England/ALDP-BC64D2/2020 | 2020-11-19 | NA |
| Wales/LIVE-DC1B67/2020 | 2020-11-19 | NA |
| England/LIVE-DC199A/2020 | 2020-11-19 | NA |
| England/LIVE-DC1BEF/2020 | 2020-11-19 | NA |
| England/ALDP-BB5324/2020 | 2020-11-19 | NA |
| Wales/PHWC-49C991/2020 | 2020-11-19 | NA |
| England/LOND-12F098B/2020 | 2020-11-20 | NA |
| England/ALDP-BB2AC8/2020 | 2020-11-20 | NA |
| England/LIVE-DC30DF/2020 | 2020-11-20 | NA |
| Wales/LIVE-DC1C64/2020 | 2020-11-20 | NA |
| England/ALDP-BBB519/2020 | 2020-11-20 | NA |
| England/CAMC-BC0292/2020 | 2020-11-20 | NA |
| England/ALDP-BBB221/2020 | 2020-11-20 | NA |
| England/ALDP-BBB0AF/2020 | 2020-11-20 | NA |
| England/ALDP-BB56C1/2020 | 2020-11-20 | NA |
| Wales/PHWC-4945D1/2020 | 2020-11-20 | NA |
| England/ALDP-BB595F/2020 | 2020-11-20 | NA |
| England/CAMC-BBDCC7/2020 | 2020-11-20 | NA |
| England/ALDP-BBAC9D/2020 | 2020-11-20 | NA |
| England/ALDP-BB4E51/2020 | 2020-11-20 | NA |
| Taiwan/NTU34/2020 | 2020-11-20 | NA |
| England/ALDP-BC6609/2020 | 2020-11-20 | NA |
| Wales/PHWC-493D9E/2020 | 2020-11-20 | NA |
| England/ALDP-BB3D46/2020 | 2020-11-20 | NA |
| England/CAMC-BBCB70/2020 | 2020-11-21 | NA |
| England/CAMC-BBC5EB/2020 | 2020-11-21 | NA |
| England/ALDP-BB9B73/2020 | 2020-11-21 | NA |
| England/ALDP-BB9C34/2020 | 2020-11-21 | NA |
| England/ALDP-BC7978/2020 | 2020-11-21 | NA |
| England/ALDP-BB92AB/2020 | 2020-11-21 | NA |
| England/ALDP-BBB4B2/2020 | 2020-11-21 | NA |
| England/CAMC-BBC9A3/2020 | 2020-11-21 | NA |
| England/NORW-F5183/2020 | 2020-11-21 | NA |
| Wales/PHWC-495330/2020 | 2020-11-21 | NA |
| England/ALDP-BBB50A/2020 | 2020-11-21 | NA |
| England/ALDP-BBB397/2020 | 2020-11-21 | NA |
| England/ALDP-BB512A/2020 | 2020-11-21 | NA |
| Wales/PHWC-494E8B/2020 | 2020-11-21 | NA |
| Wales/PHWC-495400/2020 | 2020-11-22 | NA |
| England/CAMC-BBB90E/2020 | 2020-11-22 | NA |
| England/CAMC-BC2306/2020 | 2020-11-22 | NA |
| England/ALDP-BC74FF/2020 | 2020-11-22 | NA |
| England/CAMC-BC1D90/2020 | 2020-11-22 | NA |
| England/CAMC-BC338D/2020 | 2020-11-22 | NA |
| England/CAMC-BBDE67/2020 | 2020-11-22 | NA |
| Wales/PHWC-495497/2020 | 2020-11-22 | NA |
| Wales/CAMC-BC09E8/2020 | 2020-11-22 | NA |
| Wales/PHWC-4954A6/2020 | 2020-11-22 | NA |
| Wales/CAMC-BC0951/2020 | 2020-11-22 | NA |
| England/LIVE-DCBE82/2020 | 2020-11-22 | NA |
| England/LIVE-DCC021/2020 | 2020-11-22 | NA |
| Denmark/DCGC-15662/2020 | 2020-11-23 | NA |
| Denmark/DCGC-15379/2020 | 2020-11-23 | NA |
| Denmark/DCGC-15659/2020 | 2020-11-23 | NA |
| England/CAMB-1BC1B4/2020 | 2020-11-23 | NA |
| England/NORT-1B72074/2020 | 2020-11-23 | NA |
| England/NORT-1B72047/2020 | 2020-11-23 | NA |
| England/NORT-1B71EB7/2020 | 2020-11-23 | NA |
| England/NORT-1B72065/2020 | 2020-11-23 | NA |
| England/NORT-1B71E5D/2020 | 2020-11-23 | NA |
| England/NORT-1B71F5A/2020 | 2020-11-23 | NA |
| England/ALDP-BCAA18/2020 | 2020-11-23 | NA |
| England/ALDP-BC7626/2020 | 2020-11-23 | NA |
| England/CAMC-BC1F03/2020 | 2020-11-23 | NA |
| England/NORT-1B71F69/2020 | 2020-11-23 | NA |
| England/NORT-1B71E7B/2020 | 2020-11-23 | NA |
| England/ALDP-BCA2F2/2020 | 2020-11-23 | NA |
| England/ALDP-BCA26B/2020 | 2020-11-23 | NA |
| Wales/ALDP-BCAACD/2020 | 2020-11-23 | NA |
| England/CAMC-BC0F9B/2020 | 2020-11-23 | NA |
| England/ALDP-BCA4A1/2020 | 2020-11-23 | NA |
| Wales/PHWC-4956BF/2020 | 2020-11-23 | NA |
| Wales/PHWC-495691/2020 | 2020-11-23 | NA |
| England/ALDP-BC7BBE/2020 | 2020-11-23 | NA |
| England/ALDP-BC7BDC/2020 | 2020-11-23 | NA |
| England/ALDP-BC7C15/2020 | 2020-11-23 | NA |
| England/NORT-1B7226F/2020 | 2020-11-24 | NA |
| England/LIVE-DC298A/2020 | 2020-11-24 | NA |
| England/LIVE-DC2F4C/2020 | 2020-11-24 | NA |
| Wales/PHWC-49585F/2020 | 2020-11-24 | NA |
| England/LIVE-DC2814/2020 | 2020-11-24 | NA |
| England/SHEF-D0FEE/2020 | 2020-11-24 | NA |
| Wales/PHWC-495AFF/2020 | 2020-11-24 | NA |
| Wales/PHWC-49597A/2020 | 2020-11-24 | NA |
| Wales/PHWC-496CBC/2020 | 2020-11-24 | NA |
| England/LIVE-DC6DF3/2020 | 2020-11-24 | NA |
| England/QEUH-BCCAF8/2020 | 2020-11-25 | NA |
| England/QEUH-BC3D07/2020 | 2020-11-25 | NA |
| England/QEUH-BC3D34/2020 | 2020-11-25 | NA |
| England/QEUH-BC4BC0/2020 | 2020-11-25 | NA |
| England/LIVE-DC3057/2020 | 2020-11-25 | NA |
| England/LIVE-DC2EC7/2020 | 2020-11-25 | NA |
| England/QEUH-BC4762/2020 | 2020-11-25 | NA |
| England/QEUH-BC4A0F/2020 | 2020-11-25 | NA |
| England/QEUH-BC3709/2020 | 2020-11-25 | NA |
| England/NORT-1B72223/2020 | 2020-11-25 | NA |
| England/QEUH-BC3AA6/2020 | 2020-11-25 | NA |
| England/QEUH-BC3A00/2020 | 2020-11-25 | NA |
| England/QEUH-BC3A2E/2020 | 2020-11-25 | NA |
| Wales/PHWC-495BA1/2020 | 2020-11-25 | NA |
| Wales/PHWC-495BED/2020 | 2020-11-25 | NA |
| Wales/PHWC-496D9B/2020 | 2020-11-25 | NA |
| Wales/PHWC-496D7D/2020 | 2020-11-25 | NA |
| England/QEUH-BC3736/2020 | 2020-11-25 | NA |
| England/QEUH-BCD620/2020 | 2020-11-26 | NA |
| England/QEUH-BCD4BD/2020 | 2020-11-26 | NA |
| England/QEUH-BCE522/2020 | 2020-11-26 | NA |
| England/QEUH-BCCE56/2020 | 2020-11-26 | NA |
| England/QEUH-BCCFCC/2020 | 2020-11-26 | NA |
| England/NORT-1B722AB/2020 | 2020-11-26 | NA |
| England/QEUH-BCE59B/2020 | 2020-11-26 | NA |
| England/QEUH-BCCF80/2020 | 2020-11-26 | NA |
| England/QEUH-BCD73C/2020 | 2020-11-26 | NA |
| England/QEUH-BCE258/2020 | 2020-11-26 | NA |
| England/CAMC-BD36BE/2020 | 2020-11-27 | NA |
| England/QEUH-BCBBE7/2020 | 2020-11-27 | NA |
| England/QEUH-BCBA35/2020 | 2020-11-27 | NA |
| England/QEUH-BCC09C/2020 | 2020-11-27 | NA |
| England/CAMC-BD48C6/2020 | 2020-11-27 | NA |
| England/CAMC-BD20DF/2020 | 2020-11-27 | NA |
| England/CAMC-BD435C/2020 | 2020-11-27 | NA |
| England/CAMC-BD186F/2020 | 2020-11-27 | NA |
| England/CAMC-BD20EE/2020 | 2020-11-27 | NA |
| England/CAMC-BD46DB/2020 | 2020-11-27 | NA |
| England/QEUH-BCBA80/2020 | 2020-11-27 | NA |
| England/QEUH-BCBDF0/2020 | 2020-11-27 | NA |
| England/QEUH-BCBA44/2020 | 2020-11-27 | NA |
| England/QEUH-BCBC11/2020 | 2020-11-27 | NA |
| England/QEUH-BCB9FC/2020 | 2020-11-27 | NA |
| England/QEUH-BCBB8D/2020 | 2020-11-27 | NA |
| England/NORT-2884E4/2020 | 2020-11-27 | NA |
| England/QEUH-BCB929/2020 | 2020-11-27 | NA |
| England/QEUH-BCB9B0/2020 | 2020-11-27 | NA |
| England/QEUH-BCBB6F/2020 | 2020-11-27 | NA |
| England/NOTT-11F7F9/2020 | 2020-11-27 | NA |
| England/NOTT-11F96C/2020 | 2020-11-27 | NA |
| England/NOTT-11F7EA/2020 | 2020-11-27 | NA |
| England/CAMC-BD1BFD/2020 | 2020-11-27 | NA |
| England/QEUH-BCBB23/2020 | 2020-11-27 | NA |
| Wales/PHWC-492F20/2020 | 2020-11-28 | NA |
| England/LIVE-DCC5AA/2020 | 2020-11-28 | NA |
| England/LIVE-DC678C/2020 | 2020-11-29 | NA |
| Denmark/DCGC-13058/2020 | 2020-11-30 | NA |
| Denmark/DCGC-13842/2020 | 2020-11-30 | NA |
| Denmark/DCGC-13634/2020 | 2020-11-30 | NA |
| Denmark/DCGC-14085/2020 | 2020-11-30 | NA |
| Denmark/DCGC-13108/2020 | 2020-11-30 | NA |
| Denmark/DCGC-13196/2020 | 2020-11-30 | NA |
| Wales/PHWC-4981E8/2020 | 2020-11-30 | NA |
| England/QEUH-BE4113/2020 | 2020-11-30 | NA |
| England/QEUH-BE415F/2020 | 2020-11-30 | NA |
| England/QEUH-BE4061/2020 | 2020-11-30 | NA |
| England/NORT-1B63225/2020 | 2020-12-01 | NA |
| England/MILK-BFCF96/2020 | 2020-12-01 | NA |
| England/NORT-1B62DBC/2020 | 2020-12-01 | NA |
| England/PHEC-14E7B5/2020 | 2020-12-01 | NA |
| England/NORT-1B62E31/2020 | 2020-12-01 | NA |

*University of Glasgow student cases are indicated with ‘H’ and ‘Private’ denoting students residing in university halls and private accommodation respectively. NA indicates cases deemed to be non-students.
